# Large scale deletion and rebalancing within the *k1C* kafirin family in sorghum

**DOI:** 10.3389/fpls.2025.1686027

**Published:** 2025-10-23

**Authors:** Tyler W. Ferris, Preston J. Hurst, Abou Yobi, Bara Alartouri, Aixia Li, Ruthie Angelovici, Thomas E. Clemente, David R. Holding

**Affiliations:** ^1^ Center for Plant Science Innovation, Department of Agronomy and Horticulture, University of Nebraska-Lincoln, Lincoln, NE, United States; ^2^ Bond Life Sciences Center, University of Missouri, Columbia, MO, United States; ^3^ Morrison Microscopy Core Research Facility, Nebraska Center for Biotechnology, University of Nebraska-Lincoln, Lincoln, NE, United States

**Keywords:** proteome rebalancing, prolamin, kafirin, protein bodies, lysine, seed protein, grain quality

## Abstract

*Sorghum bicolor* (L.) Moench (sorghum) is cultivated as food for humans and livestock and is valued for its low input requirements. However, sorghum grain protein is deficient in the essential amino acid lysine and has poor protein digestibility. This is because highly abundant proline-rich kafirins constitute >70% of proteins and form low-digestibility protein bodies. To reduce kafirin expression in the endosperm and elicit wholesale proteome rebalancing to increase non-kafirin proteins, a single guide RNA (sgRNA) Clustered Regularly Interspaced Short Palindromic Repeats (CRISPR/Cas9) construct was previously used to target members of the highly repetitive alpha-kafirin family, *k1C*, in RTx430 sorghum. The current study was conceived to evaluate the nutritional and biophysical characteristics of two sorghum lines with *k1C* family deletions compared to unedited RTx430. Despite confirming a ~400-kb deletion in the *k1C* family of both edited lines encompassing seven active genes, no significant decrease was observed in *k1C* expression or compensatory increase in non-kafirin proteins. A significant increase was observed in protein-bound amino acids in both *k1C*-deleted lines and in both edited lines relative to unedited RTx430, which increased total seed protein in one line. Protein bodies were observed to be more irregularly shaped, with seeds retaining wild-type levels of vitreous endosperm. RNA Isoform Sequencing (Iso-Seq) was performed to measure *k1C* family gene expression and capture differentially expressed non-kafirin genes. Five *k1C* members were observed to have biologically significant expression, with only two of the residual, non-deleted *k1C* genes having elevated expression in lines with the deletion. The results of this study demonstrate the ability of CRISPR/Cas9 editing of kafirins to elicit changes to the amino acid profile of sorghum and increase protein. Moreover, the results demonstrate an extreme example of the ability of highly repetitive genome regions such as the *k1C* subfamily to compensate for the effects of CRISPR-induced multigene deletions.

## Introduction

Sorghum is similar in nutritional composition to maize and yields higher than maize on marginal land where maize production is <95 bushels per acre. Sorghum is particularly resistant to heat stress and water stress (Staggenborg et al., 2008; Abreha et al., 2021). Like maize, sorghum grain is deficient in the essential amino acid lysine and has low protein digestibility (45.3%–56.7% in cooked, whole grain flour) (Singh and Axtell, 1973; Anglani, 1998; Duodu et al., 2003; Jin et al., 2003; Wang et al., 2003; Duressa et al., 2018). This combination of low protein digestibility and sub-optimal amino acid profile makes sorghum an incomplete source of protein for humans and monogastric livestock. The deficiency of sorghum in lysine and poor protein digestibility are attributed to the composition and accumulation of prolamin storage proteins in the endosperm.

Prolamins, known as kafirins in sorghum, account for 70% of total seed protein content and contain virtually no lysine (Oria et al., 1995). Kafirins are categorized as α, β, γ, and δ based on molecular weight and solubility (Shull et al., 1991). α-Kafirins, sub-categorized by polypeptide size, are transcribed from several gene families (Xu and Messing, 2008). Two *k1A* genes and one *k1D* gene encode the 19-kDa kafirins, while the 22-kDa α-kafirins are transcribed from a family of 20 tandem-duplicated *k1C* genes. These 19-kDa and 22-kDa α-kafirins account for almost 80% of the kafirin proteins (Shull et al., 1992; Belton et al., 2006). The kafirins coalesce into protein bodies that accumulate in the rough endoplasmic reticulum (Kumar et al., 2012). These protein bodies contain a thin outer layer of β- and γ-kafirins that engage in extensive disulfide bonding. α- and δ-Kafirins comprise the majority of the protein body and are tightly packed within it (De Mesa-Stonestreet et al., 2010). The cross-linked shell of β- and γ-kafirins and the organization of α- and δ-kafirins within the shell make these protein bodies highly resistant to protease digestion (Oria et al., 2000). The resistance of these protein bodies to digestion results in low levels of protein digestibility for the whole sorghum seed. However, the modulation of prolamin expression and accumulation can increase both protein digestibility and the proportion of lysine within the seed’s endosperm.

When prolamins are modified or their expression is reduced, the proteome of the grain compensates by increasing the expression of non-prolamins (Wu and Messing, 2014). This compensatory increase results in a greater proportion of lysine-containing proteins, thereby improving the seed amino acid profile (Zhao et al., 2023). Furthermore, the modification in the expression of prolamins can affect the protein body morphology such that the spherical bodies become invaginated. This change in morphology is thought to allow digestive gastric proteases easier access to proteins of the body, which improves protein digestibility in modified grain (Oria et al., 2000). However, modulating prolamin expression can have deleterious effects on grain quality, as is observed in *Opaque2* maize.

*Opaque2* (*o2*) (Mertz et al., 1964) is a mutation in the O2 b-ZIP transcription factor, which regulates 19- and 22-kDa zeins. The *o2* mutation leads to a decrease in the expression of zein genes and proteins and compensatory increases in non-zein genes. The resulting non-zein protein increase raises lysine content in the seed at the cost of reduced kernel hardness and vitreousness. In sorghum, Oria et al. (2000) generated a mutant line (P721Q) with a high-digestibility, high-lysine (hdhl) phenotype. The molecular basis of the mutant was characterized by Wu et al. (2013) and results from a single base-pair substitution in a single *k1C* gene. This mutation caused an alanine-to-valine substitution signal peptide cleavage site. This substitution causes an uncleaved kafirin protein, which causes a dominant negative phenotype, including an unfolded protein response (UPR) and a global decrease in kafirin proteins. Although high in digestibility and lysine, mutant seeds were low in vitreousness and floury, with increased susceptibility to breakage as well as pest and pathogen damage. Thus, it is desirable to achieve a proteome-rebalancing phenomenon in a manner without accruing deleterious end-use quality phenotypes for improving the nutritional profiles of crop species such as sorghum.

To effectively harness proteome rebalancing for improving amino acid profile and protein digestibility without sacrificing end-use quality, Li et al. used a Clustered Regularly Interspaced Short Palindromic Repeats (CRISPR/Cas9) genome editing approach to reduce kafirin expression (2018). A consensus sequence in the *k1C* subfamily genes in lines 19Q4–77 *wx* and 19Q4–94 *Wx* was targeted with a single guide (sg) RNA (sgRNA) construct to decrease total *k1C* expression. Results showed increased protein-bound and free lysine, protein content, protein digestibility, and non-kafirin expression in many of the initial transgenic lines.

One of the initial edited lines, 19Q4-13, is described by Hurst et al. (2023). The F_2_:F_3_ generation was sequenced using PacBio to generate long-read sequences that better align repetitive genomic regions such as the *k1C* genes. 19Q4–13 exhibited reduced 22-kDa alpha-kafirin and increased non-kafirin proteins, a shift in amino acid composition including a significant increase in lysine, as well as the maintenance of a vitreous endosperm phenotype. Contig alignment of the *k1C* region showed modifications in eight of 16 *k1C* genes, including a 1.35-kb deletion, a 1.35-kb inversion, and an insertion of this inverted segment into another *k1C* gene. These modifications elicited a reduction in overall *k1C* expression and an increase in non-kafirin protein expression, resulting in an *hdhl*-like phenotype.

Having produced sorghum with the desired *hdhl*-like phenotypes, introgressions of the *waxya* (*wx*) mutation into these *k1C*, hdhl lines were then performed for the purpose of investigating potential novel biophysical and nutritional seed traits. The *waxya* mutation in the *Waxy1* gene results in a loss of function for the granule-bound starch synthase (GBSS) enzyme in sorghum, resulting in high-amylopectin (>90%) starch (Sattler et al., 2009). Previous end-use characterization of high-protein digestibility, high-amylopectin sorghum revealed that the combination of these two traits resulted in flour with a greater potential for dough-based food applications (Elhassan et al., 2015). It was our goal to determine the effects of combining the milder *hdhl* phenotypes from *k1C* edited lines with the high-amylopectin phenotype conferred by *waxya* on dough-related end-use quality traits. *Hdhl* lines with seeds displaying near wild-type levels of vitreous-to-floury endosperm ratios were selected for such advancement to retain favorable seed disease and breakage resistance.

In this paper, we present data on the nutritional and biophysical evaluation of two highly inbred CRISPR/Cas9-edited sorghum lines, which are different from 19Q4–13 described above. The sgRNA binding site of the CRISPR Cas9 construct (based on an imperfect consensus target site across all *k1C* genes) was found to have a perfect consensus at two target sites in *k1C* genes that flank six additional *k1C* genes, causing a ~400-kb deletion that spans eight genes. These were lines 19Q4–77 and 19Q4-94, which were both derived from CRISPR Event 6 as described by Li et al. (2018), with “*wx*” and “*Wx*” denoting the homozygous *waxya* mutation and wild-type allele presence, respectively. 19Q4–77 *wx* was the result of the introgression of the *waxya* locus, and 19Q4–94 *Wx* is wild type for *WAXY*. Surprisingly, both 19Q4–77 *wx* and 19Q4–94 *Wx* exhibited no evidence of non-kafirin protein rebalancing and no reduction in 22-kDa kafirin protein. These results, in addition to observing marginal protein body morphology alteration, indicate that a non-kafirin-based shift toward an increase in elevated essential amino acids does not appear to be occurring in these lines. This prompted us to characterize lines with long-read PacBio whole-genome sequencing and RNA Iso-Seq to determine the potential cause of this discrepancy. We discovered that the *k1C* deletion contained seven active kafirin genes, of which only three were transcribed substantially. Of the remaining *k1C* genes not deleted, only two are substantially transcribed. This genotype resulted in increased protein-bound and free amino acid profiles in both lines. A greater total protein content and a reduction in total starch were observed in 19Q4–77 *wx*, while 19Q4–94 was near wild type in total starch and protein contents. The *waxya* mutation conferred increased amylopectin but did not affect protein body morphology or kafirin expression. Although lysine was not drastically increased as it was in 19Q4-13, a modest increase in lysine was observed in 19Q4–77 *wx*.

## Materials and methods

### Germplasm, DNA extraction, *waxya* introgression, and agarose gel electrophoresis

**Figure 1:**
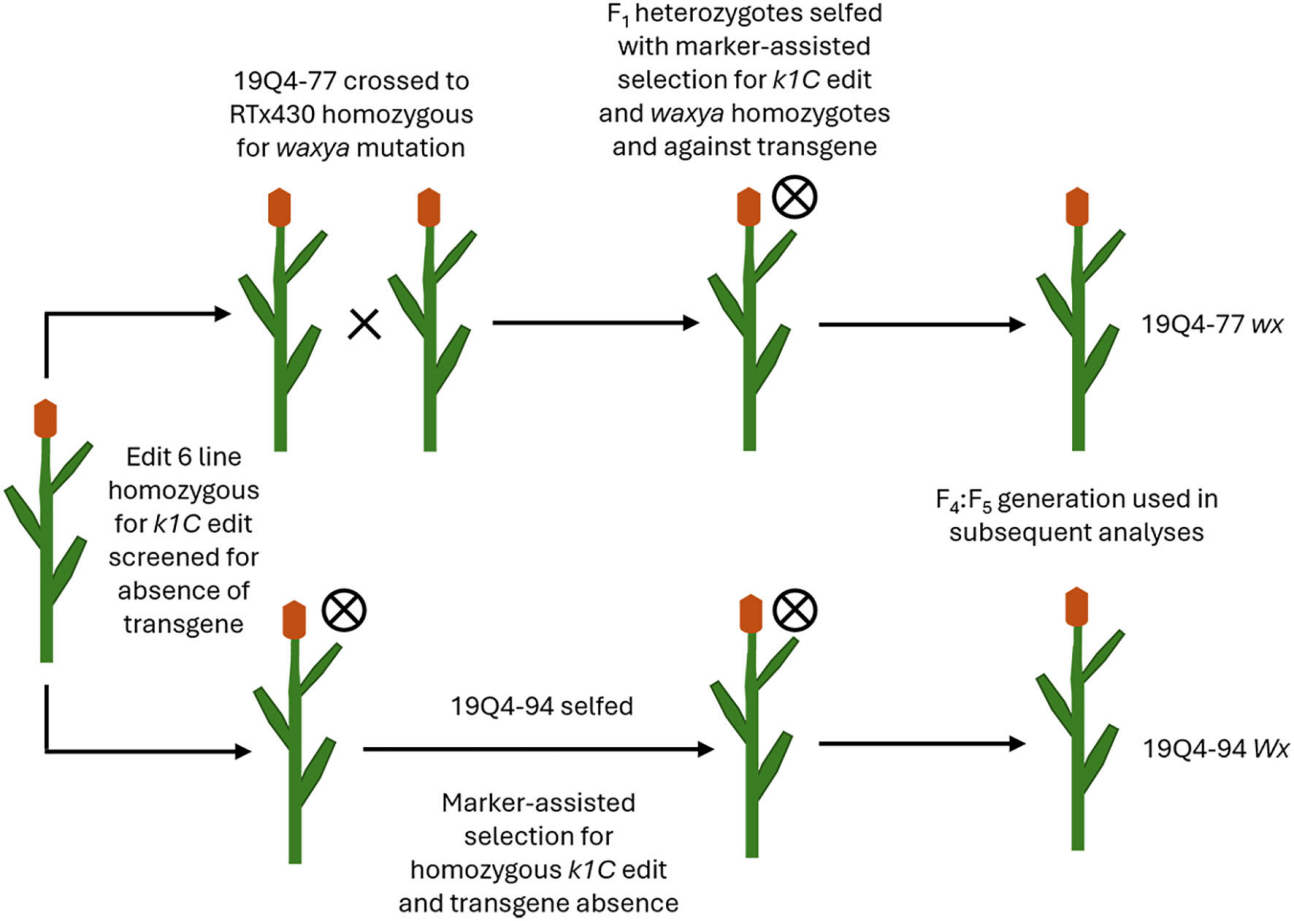
Visualization schematic for lines 19Q4–77 wx and 19Q4–94 Wx as derived from CRISPR Event 6 germplasm.

**Figure 2:**
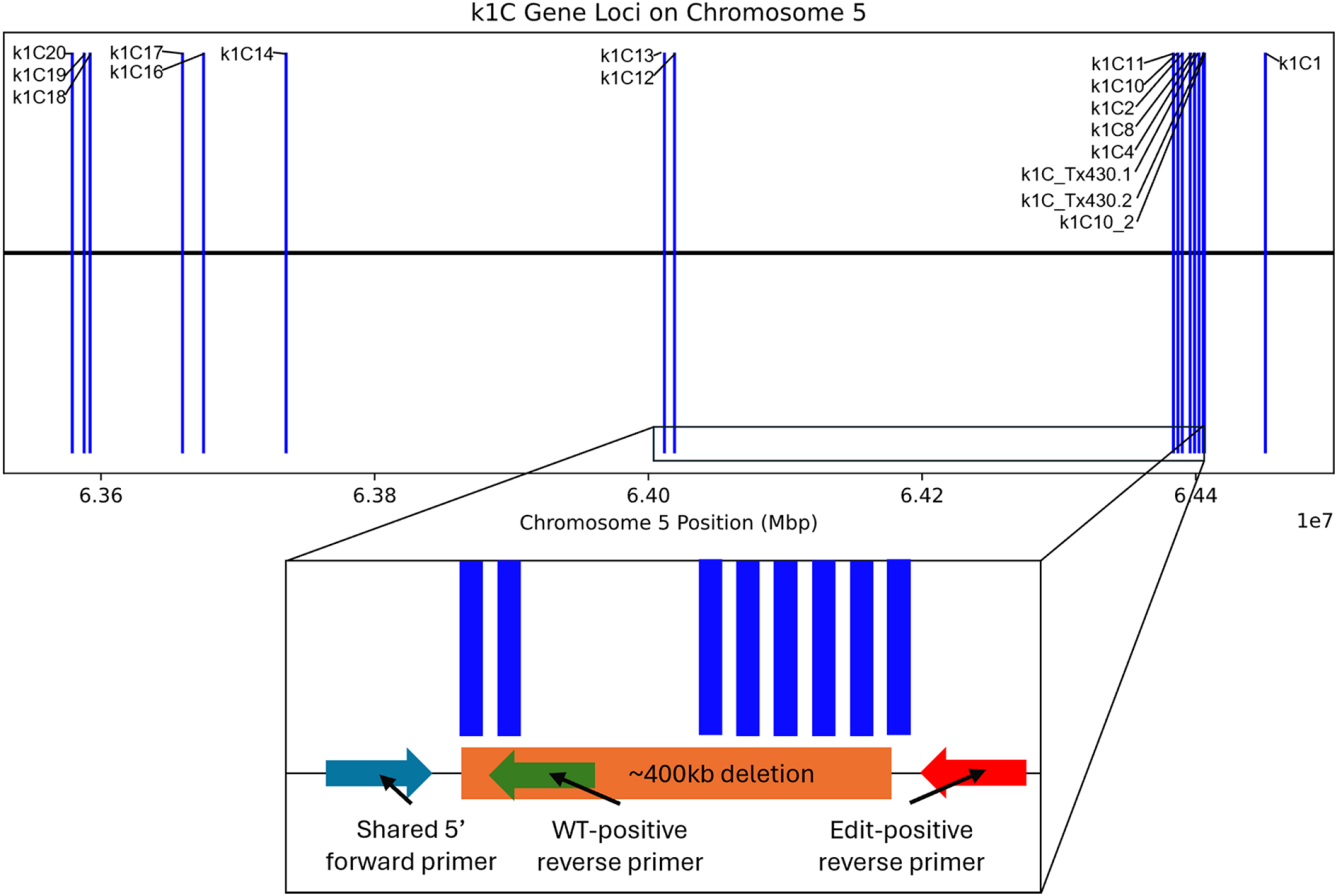
*k1C* loci as indicated by BLAST alignment of extracted *k1C* sequences against BTx642 annotated *k1C* genes; ~400-kb deletion (not to scale) is depicted in the *k1C* family in an inset below, with the primer design scheme for the marker-assisted selection of the ~400-kb deletion also depicted in the inset.

Both 19Q4–77 *wx* and 19Q4–94 *Wx* CRISPR-edited sorghum were developed from a CRISPR-edited line (Event 6) in wild-type RTx430 sorghum produced at the University of Nebraska-Lincoln Plant Transformation Core Research Facility. The details of the original vector design, transformation, and phenotyping are previously described (Li et al., 2018). RTx430 stock homozygous for the *waxya* mutation was crossed with 19Q4-77, with F_1_ plants screened for the presence of *wx* alleles using markers positive for the *waxya* mutation. The Event 6 germplasm was screened for the absence of the CRISPR Cas9 transgene prior to crossing with *waxya* RTx430 and again at the F_2_F_3_ generation. A schematic of the breeding scheme used in generating 19Q4–77 *wx* and 19Q4–94 *Wx* is shown in **Figure 1**. 19Q4–77 *wx*, 19Q4–94 *Wx*, and RTx430 plants were grown for experimentation in greenhouse conditions and were self-pollinated. To select for the homozygosity of the CRISPR deletion, markers were designed to detect the junction sites of the deletion. Total DNA was extracted and purified using the BioSprint DNA Plant Kit (Qiagen; Catalog no. 941557: Limburg, Netherlands). PCR using pairs of degenerate primers was performed to generate amplicons characterizing the CRISPR deletion site. Primers 5′-TGGATTGGCAACTACCAGTG-3′ with 5′-ATTGTGAGTGCCCTGATGTG-3′ were used to generate amplicons 780 bp in length in edited lines. One primer target site was located upstream of the 5′ breakpoint of the deletion, with the other near the 3′ end. Amplification can only occur if the ~400-kb deletion is present, as the primer positions are too far apart to amplify if the deletion is not present. Primers 5′-TGGATTGGCAACTACCAGTG-3′ with 5′-CGAACTAGCAACGTCCTTCC-3′ were used to generate amplicons 716 bp in unedited lines. One primer target site was located upstream of the 5′ breakpoint of the deletion, with the other target site located downstream within and adjacent to the 5′ breakpoint. Amplification is only possible in unedited lines, as the target site for the 3′ primer does not exist in an edited line. A visual representation of the primer design and the ~400-kb deletion in the *k1C* family is shown in **Figure 2**.

Amplicons were visualized using agarose gel electrophoresis. PCR products were combined with Orange DNA Loading Dye (Thermo Fisher; Catalog no. R0631: Massachusetts, United States) and loaded onto 1%–2% agarose gels stained with ethidium bromide. Gel image analysis was performed using the Bio-Rad ChemiDoc Imaging System with ethidium bromide fluorescence at 590/110 nm (Bio-Rad; Catalog no. 12003153).

### Protein extraction and SDS–PAGE

For whole-seed protein extraction, four to five mature seeds each from a different biological replicate were ground using a Wig-L-Bug dental amalgamator (Sigma-Aldrich; Catalog no. Z111392: Massachusetts, United States) for 1–2 minutes until a uniform fine texture was achieved. Fifty milligrams of flour was suspended with 1 mL of borate extraction buffer before performing seed protein extraction as previously described (Wallace et al., 1990). Samples were incubated at room temperature with gentle shaking for 2 hours. After extraction, solutions were centrifuged at 12,400 *g* for 15 minutes at room temperature to pellet kafirins. Thirty microliters of deionized water plus 10 μL of 5× sodium dodecyl sulfate loading buffer was used to resuspend the kafirin pellets for gel loading. The non-kafirin-containing pellets were resuspended in 200 μL of deionized water using gentle pipetting and stirring. Fifty microliters of the non-kafirin solution was added to a new tube with 10 μL sodium dodecyl sulfate loading buffer for gel loading. Three kafirin and three non-kafirin fraction gels were generated from three separate whole-seed protein extractions. All samples were stored with loading buffer at −20 °C.

Sodium dodecyl sulfate–polyacrylamide gel electrophoresis (SDS–PAGE) was performed via separation of both kafirin and non-kafirin fraction solutions on 12% (w/v) gels stained with Coomassie Brilliant Blue R-250 dye (Sigma-Aldrich; Catalog no.112553: Massachusetts, United States). Five microliters of kafirin loading solution and 10 μL of non-kafirin loading solution were loaded and run at 75 V for approximately 3.5 hours. Gel image analysis was performed using the Bio-Rad ChemiDoc Imaging System (Bio-Rad; Catalog no. 12003153).

### Transmission electron microscopy of protein bodies

Sorghum seeds were harvested 25 days after pollination, and a ~2-mm transverse slice was cut from each seed using a double-sided razor blade. Tissue was fixed in 2% paraformaldehyde/2% glutaraldehyde (vol/vol) in 0.1 M sodium cacodylate buffer (pH 7.4) for 1 hour at room temperature and up to 24 hours at 4°C. Small wedges from these slices were then made using a scalpel blade, which were then post-fixed with 1% osmium tetroxide for 1 hour, followed by rinses. Samples were dehydrated with graded ethanol to 100%, infiltrated and embedded in Spurr’s resin, and polymerized at 60 °C–65 °C for 24–36 hours. Ultrathin sections (70–80 nm) were stained with uranyl acetate and lead citrate and imaged on a Hitachi HT7800 TEM at 80 kV; images were acquired from the third sub-aleurone cell layer.

### Protein-bound and free amino acid profiling

Protein-bound and free amino acids were measured as described by Fountoulakis and Lahm (1998). Fifty milligram whole-inflorescence seed pools of flour from nine biological replicates per line (*n* = 27) were prepared using a Wig-L-Bug grinder/mixer (Sigma-Aldrich; Catalog no. Z111392: Massachusetts, United States) and run for 1–2 minutes until a uniform flour texture was achieved. Three technical replications of each biological replication were performed for each line (*n* = 81). Approximately 5 mg of flour was homogenized and incubated in an aqueous solution and filtered through polytetrafluoroethylene via centrifugation.

Protein-bound amino acids were extracted from whole-seed flour using acid hydrolysis, with free amino acids extracted with an aqueous extraction. Filtered samples were then diluted with the standard buffer and analyzed using ultra-performance liquid chromatography–tandem mass spectrometry (UPLC–MS/MS) described by Angelovici et al. (2013). Sample means of 19Q4–77 *wx* and 19Q4–94 *Wx* were then compared to unedited RTx430 using a Student’s *t*-test (two samples assuming equal variances) (*n* = 27).

### Total starch and amylose/amylopectin determination

Total seed starch content was determined using the Megazyme Total Starch (α-Amylase/Amyloglucosidase) Assay Protocol (SKU: 700004351 K-TSTA-100A) with amylose/amylopectin determination performed using the Megazyme Amylose/Amylopectin Assay Kit (SKU: 700004262 K-AMYL). Flour was prepared following the aforementioned Wig-L-Bug methodology. Included protocol steps for the removal of d-glucose, maltodextrins, and total starch were performed on flour samples from whole-inflorescence flour pools from three biological replicates for all three lines prior to total starch determination for each line in two technical replications (*n* = 18). Sample absorbance was measured at 505/630 nm using a MegaQuant™ Wave Spectrophotometer with 3.0 mL of the included GOPOD™ reagent incubated with a final sample extract of 0.1 mL against sample blanks. d-Glucose standards were prepared by incubating 1.0 mL of included d-glucose with the GOPOD™ reagent for standard generation. Sample means of 19Q4–77 *wx* and 19Q4–94 *Wx* were then compared to unedited RTx430 using a Student’s *t*-test (two samples assuming equal variances) (*n* = 6).

### 100-seed weight measurement

A total of 100 mature seeds from 10 19Q4–77 *wx*, 19Q4–94 *Wx*, and RTx430 plants (*n* = 30) were manually separated from the panicle and weighed to obtain 100-seed weight values in grams. Average values from each line were calculated from the 10 replicate seed pools and were then compared to unedited RTx430 using a Student’s *t*-test (two samples assuming equal variances) (*n* = 10).

### Total protein determination

Total seed protein quantification was performed using the Thermo Scientific BCA™ Protein Assay Kit for protein assay using bicinchoninic acid (Prod # 23225). Whole-inflorescence flour pools from nine biological replicates per line (19Q4–77 *wx*, 19Q4–94 *Wx*, and RTx430) (*n* = 27) were assembled into three technical replications per line (*n* = 81). Flour was prepared following the aforementioned Wig-L-Bug methodology. BCA working reagent was prepared in accordance with the microplate procedure, with diluted bovine serum albumin (BSA) standards prepared in 1,000, 500, 125, and 25 μg/mL concentrations. Sample absorbance was measured at 562 nm against average blank measurements with a standard curve generated from BSA standards for total protein concentration determination using a microplate reader. Sample means of 19Q4–77 *wx* and 19Q4–94 *Wx* were then compared to unedited RTx430 using a Student’s *t*-test (two samples assuming equal variances) (*n* = 27).

### PacBio whole genome sequencing (WGS)

High-molecular-weight genomic DNA was extracted from leaf tissue samples from two 19Q4–77 *wx*, 19Q4–94 *Wx*, and RTx430 plants each using the NucleoBond HMW DNA kit (Macherey-Nagel^®^). HiFi libraries were generated using the SMRTbell^®^ Prep Kit 3.0 (PacBio^®^) and sequenced on the Revio in WGS whole genome sequencing. HiFi Reads mode. Genome assembly was performed using minimap2 read alignment and the Phytozome *Sorghum bicolor* RTx430 v2.1, DOE-JGI reference. These sequence data were produced by the US Department of Energy Joint Genome Institute. The ~400-kb deletion region was identified using variant calling performed with Sniffles2 filtered for maximum quality score, which was then extracted and assembled into contigs along with 100 Mb upstream and downstream from the deletion using Canu.

### RNA extraction and PacBio Iso-Seq

Five seeds harvested 20 days after pollination were frozen in liquid nitrogen and pulverized for crude RNA extraction and purification for each line. The Plant RNA Protocol I procedure from the E.Z.N.A™ Plant RNA Mini Kit was followed in accordance with the manufacturer’s guidelines. Two biological replicates’ purified RNA per line was subjected to cDNA synthesis and barcoded amplification using the Iso-Seq Express 2.0 kit (PacBio^®^). Barcoded products were then multiplexed and arrayed using the Kinnex Full-Length RNA kit (PacBio^®^) and sequenced using the Revio in Kinnex Full-Length RNA mode. Reads were then demultiplexed, and adapters were trimmed. After Iso-Seq pre-processing, transcriptome alignment and quantification were performed using the Phytozome *S. bicolor* RTx430 v2.1 reference with Salmon (Sorghum bicolor RTx430 v2.1, DOE-JGI). Differential gene expression was then performed using DESeq2 with a false discovery rate cutoff of 0.05. Differentially expressed genes (DEGs) can be found in **Supplementary 1**. DEG sequences were then obtained by extracting associated FASTA sequences from the reference genome and exon GFF3 files. These extracted sequences then underwent BLAST analysis with 100% alignment length parameters against the annotated *k1C* sequences of *S. bicolor* BTx642 v1.1, DOE-JGI (Sorghum bicolor BTx642 v1.1, DOE-JGI), produced by the US Department of Energy Joint Genome Institute to identify RTx430 *k1C* sequences. **Figure 2** denotes the loci of each *k1C* member identified using BLAST of the extracted *k1C* sequences. The top 10 most abundant gene ontology (GO) terms by the presence of differentially expressed genes of 19Q4–77 *wx* and 19Q4–94 *Wx* can be viewed in **Table 1**. Differential *k1C* expression for the two edited lines is listed in **Tables 2A** and **Tables 2B** in heatmap format. Conditional formatting in Microsoft Excel was used to format each column’s range of values with a green to orange to red color gradient. The gradient’s midpoint corresponds with the 50th percentile value of each range, with either end of the gradient based on the maximum (red) and minimum (green) values and scaled based on the relative magnitude of each range of data.

**Table 1:** Table of the top 10 most abundant GO terms by presence of differentially expressed genes for 19Q4–77 *wx* and 19Q4–94 *Wx*.

19Q4–77 *wx*		19Q4–94 *Wx*	
GO term	# of genes	GO term	# of genes
GO:0005840	38	GO:0005515	143
GO:0003735	37	GO:0055114	129
GO:0006412	37	GO:0003677	86
GO:0005622	31	GO:0016021	82
GO:0003677	22	GO:0003824	79
GO:0016020	19	GO:0016491	71
GO:0016021	16	GO:0006355	61
GO:0005515	14	GO:0005524	61
GO:0003676	12	GO:0016020	57
GO:0005634	11	GO:0008152	56

GO, gene ontology.

**Table 2A:** Heatmap of differentially expressed *k1C* genes for 19Q4–77 *wx* relative to unedited RTx430.

geneID	baseMean	log2FoldChange	lfcSE	stat	pvalue	padj
SbiRTX430.05G198600	83,162.04238	−9.405351175	0.448145533	−20.98726973	8.57E−98	2.12E−94
SbiRTX430.05G198800	181,003.9472	−9.206208588	0.435936457	−21.11823509	5.41E−99	1.56E−95
SbiRTX430.05G202500	348,131.9315	−8.781618235	0.811221405	−10.82518063	2.62E−27	4.11E−24
SbiRTX430.05G202600	5,415.534359	−9.528698804	0.41295823	−23.07424365	8.40E−118	2.91E−114
SbiRTX430.05G202700	8,373.934697	−8.869342835	0.375793414	−23.60164522	3.71E−123	2.14E−119
SbiRTX430.05G202800	6,666.119449	−9.480606047	0.402458168	−23.55674902	1.07E−122	4.63E−119
SbiRTX430.05G203000	5,933.239827	−9.705211273	0.406779755	−23.85863886	8.24E−126	7.12E−122
SbiRTX430.05G203100	226.5376623	−9.164308915	0.948676485	−9.66009916	4.45E−22	6.42E−19

A three-color scale was used based on a continuous gradient (red to orange to green), based on the relevant magnitude of the data in each column, to format each range of data. The gradient’s midpoint corresponds to the 50th percentile of the data’s range.

**Table 2B:** Heatmap of differentially expressed *k1C* genes for 19Q4–94 *Wx* relative to unedited RTx430.

geneID	baseMean	log2FoldChange	lfcSE	stat	pvalue	padj
SbiRTX430.05G198600	83,162.04238	−8.672381975	0.448321473	−19.34411466	2.28E−83	6.76E−80
SbiRTX430.05G198800	181,003.9472	−8.621458383	0.436127533	−19.76820479	5.59E−87	1.99E−83
SbiRTX430.05G202500	348,131.9315	−8.262423844	0.811273531	−10.18451056	2.33E−24	4.59E−21
SbiRTX430.05G202600	5,415.534359	−8.946934971	0.421399486	−21.23148051	4.89E−100	2.17E−96
SbiRTX430.05G202700	8,373.934697	−8.236680668	0.378788651	−21.74479264	7.74E−105	4.58E−101
SbiRTX430.05G202800	6,666.119449	−9.052172159	0.414018582	−21.86416881	5.70E−106	5.06E−102
SbiRTX430.05G203000	5,933.239827	−9.240280334	0.420220402	−21.98912831	3.66E−107	6.50E−103
SbiRTX430.05G203100	226.5376623	−9.920125505	1.503914618	−6.596202594	4.22E−11	1.92E−08

A three-color scale was used based on a continuous gradient (red to orange to green), based on the relevant magnitude of the data in each column, to format each range of data. The gradient’s midpoint corresponds to the 50th percentile of the data’s range.

## Results

### Kafirin and non-kafirin seed protein fractions exhibited similar abundance in edited and non-edited lines

**Figure 3:**
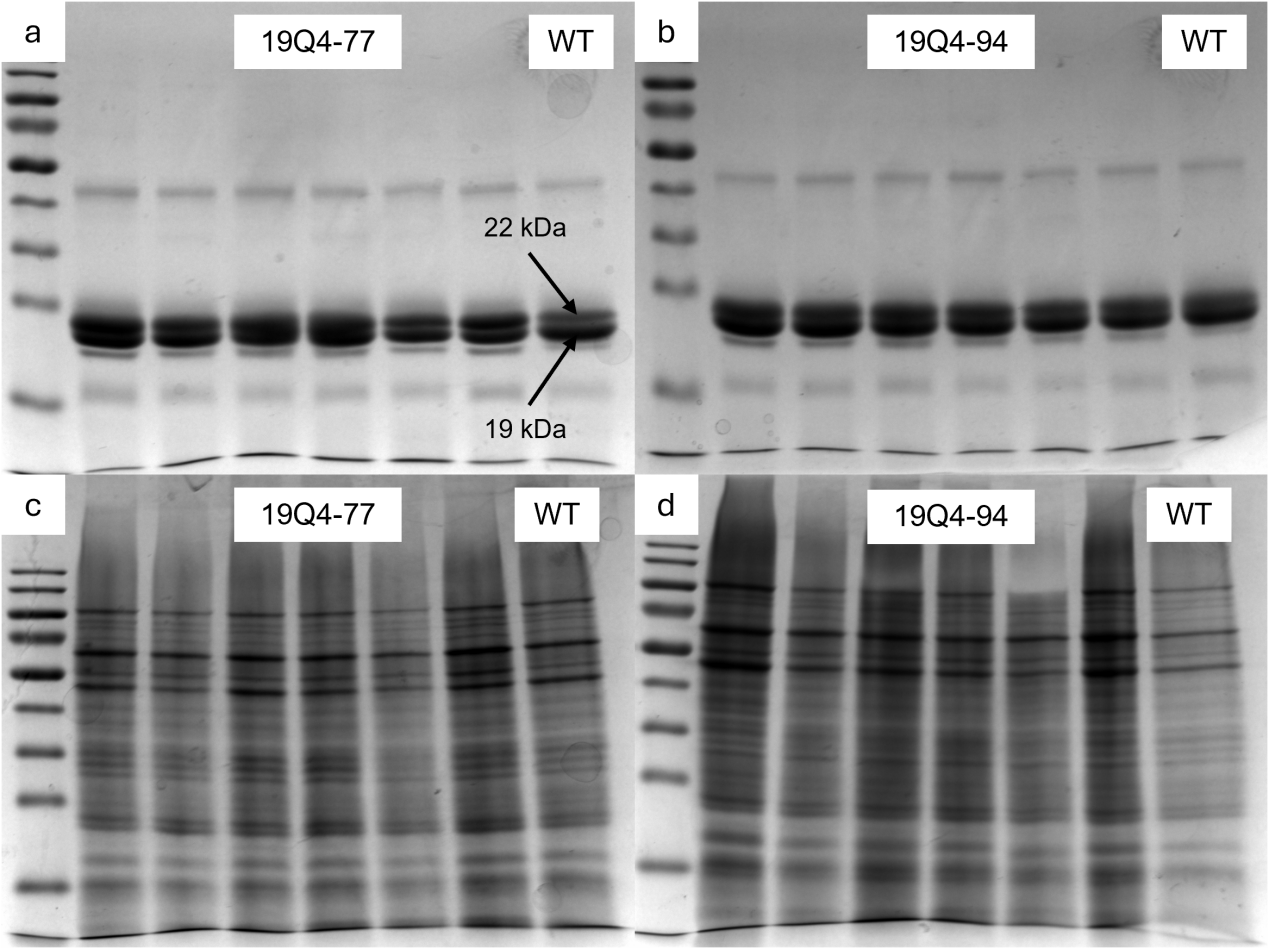
Sodium dodecyl sulfate–polyacrylamide gel electrophoresis of kafirin protein fraction **(a, b)** and non-kafirin protein fraction **(c, d)** from 19Q4–77 w*x* **(a, c)** and 19Q4–94 W*x* **(b, d)**. First lane contains 50-kDa ladder, lanes 2–7 contain loading solution from three to five seeds of identical plant, and lane 8 contains RTx430 [wild type (WT)] flour pooled from three separate plants.

To separate kafirins and non-kafirins for comparison across edited and unedited lines, pools of each fraction were analyzed using SDS–PAGE. Kafirin (**Figures 3a, b**) and non-kafirin (**Figures 3c, d**) fractions were prepared from 19Q4–77 *wx* and 19Q4–94 *Wx* derived from single-plant pools of seeds. The wild-type kafirin and non-kafirin fraction composition was characterized by pooling three kafirin/non-kafirin extractions from three pools of seeds from single-plant RTx430 plants. Based on the magnitude of protein resolution at 22 kDa, kafirin proteins had similar abundances in edited lines 19Q4–77 *wx* and 19Q4–94 *Wx* compared to unedited wild-type RTx430 (**Figures 1b**, **3a**). Non-kafirin abundance based on protein resolution excluding 22 kDa between 19Q4–77 *wx* and wild type (**Figure 3c**) is similar, with little evidence of an increase in non-kafirin protein abundance in 19Q4–94 *Wx* (**Figures 3c, d**). The presence of the waxy mutation did not obviously affect kafirin and non-kafirin abundance.

### Protein body morphology, endosperm texture, and 100-seed weight differed little between edited and non-edited lines

**Figure 4:**
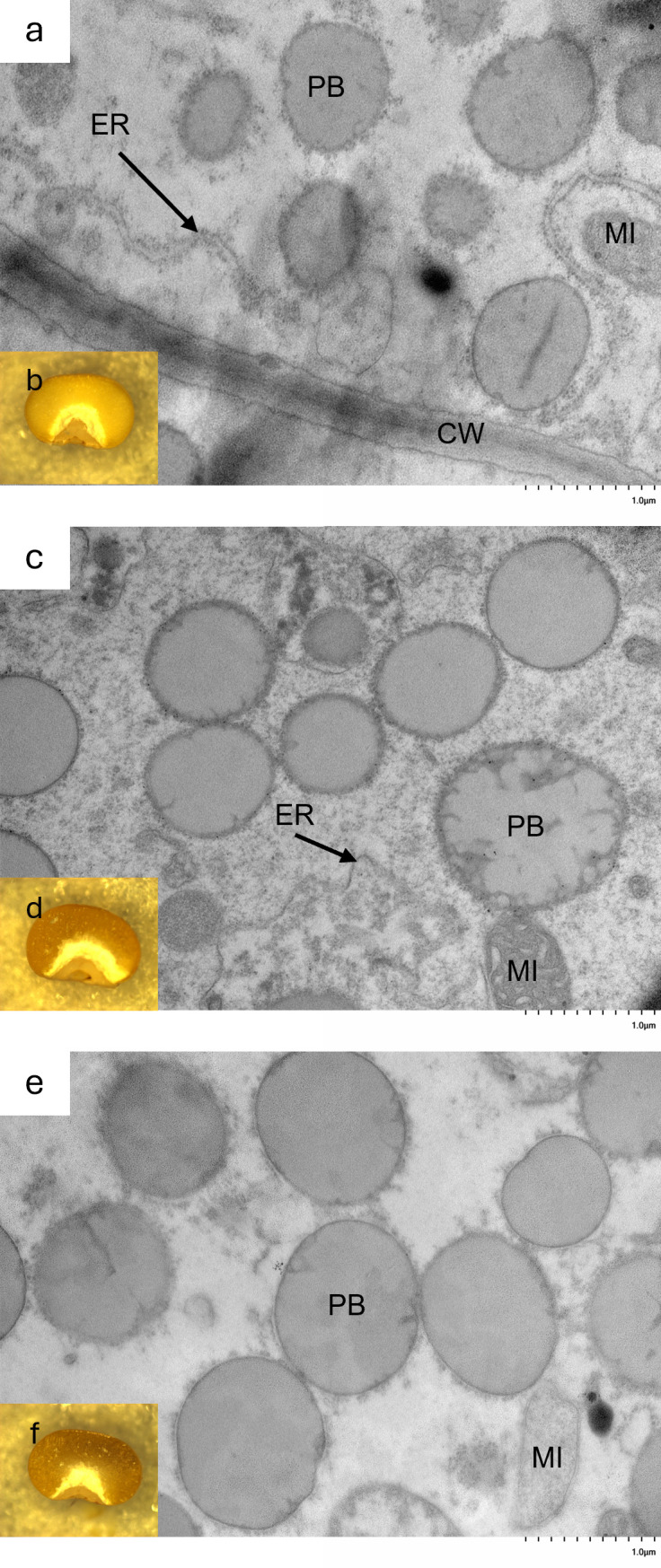
Morphology of endosperm’s third cell layer from aleurone layer of **(a)** 19Q4–77 *wx*, **(c)** 19Q4–94 *Wx*, and **(e)** RTx430 by transmission electron microscopy and dissecting microscope image of basal half of 19Q4–77 *wx* **(b)**, 19Q4–94 *Wx* **(d)**, and RTx430 **(f)** seed representative of endosperm texture. PB, protein body; ER, rough endoplasmic reticulum; MI, mitochondria; CW, cell wall.

Oria et al. (2000) described the protein bodies of hdhl P721Q as irregular in shape, which is thought to facilitate accessibility to digestive enzymes, resulting in increased protein digestibility. TEM was used to visualize 20 days after pollination, developing kernels of 19Q4–77 *wx*, 19Q4–94 *Wx*, and RTx430 to determine if and how the *k1C* deletion affected protein body morphology. The surfaces of protein bodies from 19Q4–77 *wx* and 19Q4–94 *Wx* displayed a greater degree of irregularities characterized by irregular margins and invaginations, although not nearly as severe as the original HDHL (**Figures 4a, c**). Additionally, these protein bodies were generally smaller compared to the unedited RTx430 line (**Figure 4e**), indicating the potential for increased protein digestibility in those lines.

**Figure 5:**
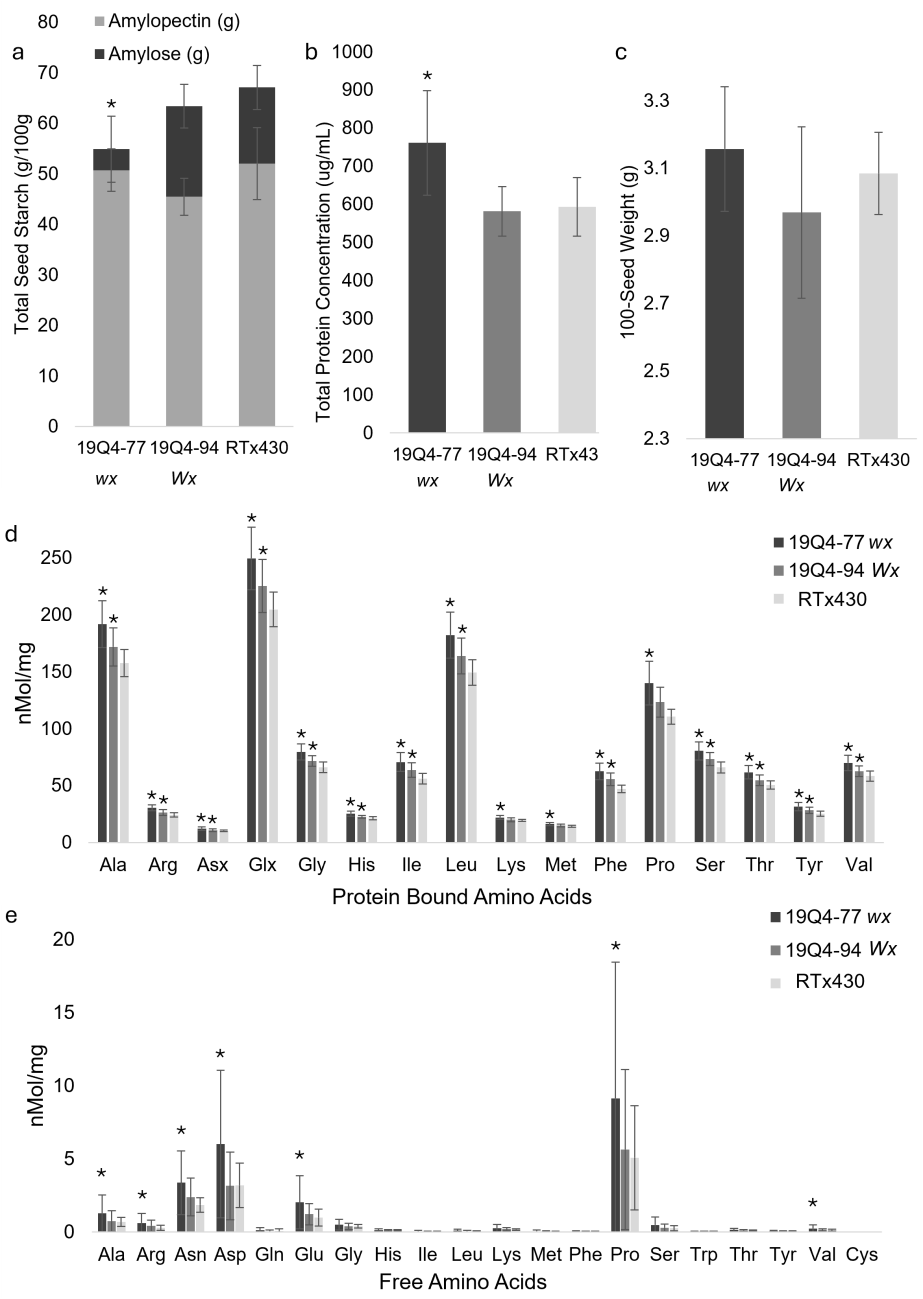
Starch content (g/100 g) and corresponding amylose/amylopectin content **(A)**, protein concentration (µg/mL) **(B)**, nanomolar concentration of protein-bound **(D)** and free **(E)** amino acids per milligram, and 100-seed weight g **(C)** of 19Q4–77 *wx*, 19Q4–94 *Wx*, and RTx430 [wild type (WT)] seeds. Error bars (*n* = 6 for total starch content, *n* = 10 for 100-seed weight, and *n* = 27 for protein concentration, free amino acids, and protein-bound amino acids) represent standard deviation. Bars with asterisks denote significant differences between that sorghum line and unedited RTx430. The asterisk for 19Q4–77 *wx* denotes significant differences between said line and unedited RTx430’s total starch content as well as amylose/amylopectin ratio (Student’s *t*-test, α = 0.05).

The approximate relative amounts of the vitreous and floury endosperm of 19Q4–77 *wx*, 19Q4–94 *Wx*, and RTx430 were visualized to determine end-use quality. Largely comparable proportions of the vitreous and floury endosperm were observed in 19Q4–77 *wx* and 19Q4–94 *Wx*, likely indicating favorable seed strength traits. The marginal thickening of the floury layer in edited lines (**Figures 4b, d**) relative to RTx430 (**Figure 4f**) at the distal edges of the floury layer in 19Q4–77 *wx* and 19Q4–94 *Wx* suggests that the modification of kafirins is occurring without affecting these end-use traits. A reduced 100-seed weight was not observed in either 19Q4–77 *wx* or 19Q4–94 *Wx*, which had 100-seed weights of 3.16 and 2.97 g, respectively, compared to the 3.08-g 100-seed weight of RTx430 (**Figure 5c**). Despite the similarities in vitreousness, the characteristic waxy phenotype is apparent in **Figure 4b**. The seed characteristics of protein body morphology, endosperm vitreousness, and 100-seed weight appeared to be highly similar between edited and unedited lines.

### 19Q4–77 *wx* had higher total seed protein and lower total starch than 19Q4–94 *Wx* and RTx430

Amino acid content, total starch, amylose/amylopectin, and total protein contents were measured from mature seeds to capture differences in the nutritional profiles of edited lines (**Figure 5**). All protein-bound seed amino acids are significantly increased in 19Q4–77 *wx* relative to unedited RTx430 (p < 0.05) (**Figure 5d**). 19Q4–94 *Wx* contained more protein-bound amino acids relative to RTx430 except for Asx, Lys, and Met (p < 0.05). Free amino acids were slightly increased in 19Q4–77 *wx* and to a lesser extent in 19Q4–94 *Wx*, but not as consistently as protein-bound amino acids (**Figure 5e**). This increase in protein-bound amino acids was greater in 19Q4–77 *wx*, with this line containing a statistically increased total seed protein content relative to RTx430 (p = 9.7E−17). 19Q4–94 *Wx* showed no statistical difference in total protein content (p = 0.30772). 19Q4–77 *wx* also contained statistically lower total seed starch content relative to RTx430 (p < 0.00257), with no significant difference observed in 19Q4–94 *Wx* compared to RTx430.

The difference in total protein and total starch between 19Q4–77 *wx* and RTx430 was an increase and a decrease of ~20%, respectively (**Figures 5a, b**). 19Q4–77 *wx* starch was composed of 7.53% amylose and subsequently 92.47% amylopectin, which is characteristic of the *waxya* mutation. This difference in starch composition was significant in both 19Q4–94 *Wx* (p = 0.00025) with an amylose percentage of 28.25% and RTx430 (p = 0.00067) with an amylose percentage of 22.48%. This shift in favor of protein at the expense of starch in 19Q4–77 *wx* was further supported by the absolute increase in protein-bound amino acid and the general increase in free amino acids. Average protein-bound lysine was increased at 11.60% (22.04 nMol/mg), with protein-bound methionine increased to 12.22% (16.32 nMol/mg) in 19Q4–77 wx (**Figure 5d**). Proline, an amino acid of high abundance in prolamins, was increased in the protein-bound fraction for both 19Q4–77 *wx* (+20.85%, 139.92 nMol/mg) and 19Q4–94 *Wx* (+10.22%, 123.36 nMol/mg) (**Figure 5d**) and in the free fraction for 19Q4–77 *wx* (+44.39%, 9.14 nMol/mg) (**Figure 5e**). In summary, 19Q4–77 *wx* grain contained a greater increase in amino acids and a subsequent increase in total protein at the expense of starch than unedited RTx430.

### PacBio WGS and junction fragment PCR revealed a ~400-kb deletion in *k1C* family

PacBio WGS was performed to characterize the deletion region in the highly repetitive *k1C* family with long-read resolution and to identify other structural variants not captured with the short read-based Illumina MiSeq sequencing used in the initial transformant lines. Barcoded library sizes were quantified fluorometrically and analyzed on TapeStation (Agilent^®^) with an average library size of 16.5 kb. Circular consensus sequencing (CCS) total yield was 157.21 Gb and 15.4 M HiFi reads; >99% of HiFi reads contained barcodes with sample sizes between 12 and 43 Gb. Genome mapping rates were >99% for each input file. The 19Q4–77 *wx* assembly contained 16 contigs with an N50 value >3,000,000.

**Figure 6:**
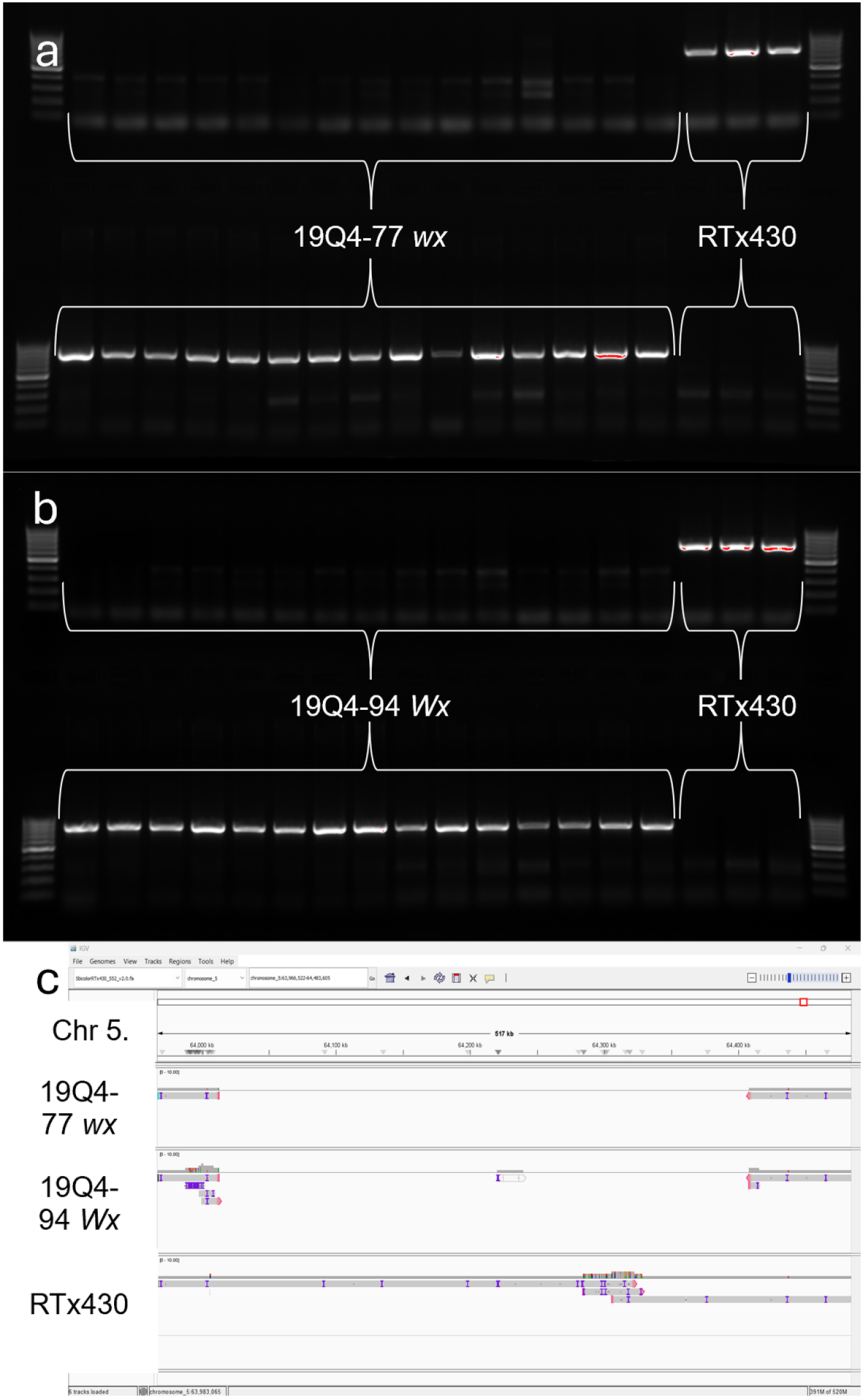
Polymerase chain reaction detection of breakpoint region in **(A)** 19Q4–77 *wx* and RTx430 and **(B)** 19Q4–94 *Wx* and RTx430 and Integrative Genome Viewer screenshot of contiguous assemblies of 19Q4–77 *wx*, 19Q4–94 *Wx*, and RTx430 reads at and around the ~400-kb deletion in *k1C* family **(C)**. Top row depicts amplification of unedited genome using primer set in deleted region (716 bp), and bottom row depicts amplification of junction fragment from edited genome using primer set that spans deletion (780 bp). First and last lanes of each row contain a 1-kb ladder. Lanes 2–14 for top and bottom rows contain DNA from 19Q4–77 *wx* **(A)** and 19Q4–94 *Wx* **(B)** plants, with lanes 17–19 containing DNA from unedited RTx430 plants (both).

Sniffles2 identified a deletion with maximum quality score on Chromosome 5 beginning at position 64012564 and ending at 64407899. Contig assembly of reads aligning between 63.0 and 65.4 Mb on Chromosome 5 at a coverage of 30× was performed (**Figure 6c**). The 19Q4–94 *Wx* assembly contained 16 contigs with an N50 value >1,900,000. Finally, the RTx430 assembly contained 12 contigs with an N50 value >2,500,000. Based on these findings, the deletion detected via short read MiSeq was verified, and the absence of any additional *k1C* modifications, including duplicated and/or translocated *k1C* material, was confirmed.

Junction fragment amplification and sequencing were used to characterize the presence or absence of the CRISPR deletion in 19Q4–77 *wx* and 19Q4–94 *Wx*. The amplification of the homozygous, deletion-free, wild-type genome can be observed in the wild-type (WT) labeled lanes with resolution at 716 bp and a lack of amplicon banding at 780 bp (**Figures 6a, b**). The presence of the deletion is denoted by an amplicon resolving at 780 bp using deletion-present designed primers. The lack of a band resolving at 716 bp for these plants indicates the lack of heterozygosity at the deletion site and, thus, a homozygous status for the deletion in these plants. These results support the proposed existence of a 400-kb deletion in 19Q4–77 *wx* and 19Q4–94 *Wx* in the *k1C* family as initially characterized by short-read sequencing.

### RNA Iso-Seq and differential gene expression analysis of *k1C* genes and non-kafirin genes

RNA Iso-Seq was conducted to obtain reliable and specific expression data of *k1C* genes and identify differentially expressed non-kafirin genes. A total of 9.3 M HiFi reads were generated with a mean read length of 9.19 kb, a mean polymerase read length of 79.9 kb, and a median Phred score of Q36. Of the reads, 96.68% contained full arrays with an average array size of 7.93. Demultiplexing analysis revealed Iso-Seq primer barcodes present in >98% of reads, with primer quality ranging from 99.6 to 99.7. Transcriptome alignment and transcript quantification were performed using Salmon, with mapping rates for transcriptome alignment >80% for each sample. A total of 46,750 transcripts were aligned to the reference transcriptome. Transcripts containing >1 transcript per million (TPM) were 14,405 to 17,929, and transcripts with a read number (NumRead) >0 were 26,535 to 29,114 across all samples. Differential gene expression was performed using DESeq2 with a false discovery rate-corrected p-value (padj) threshold of <0.05. A total of 26 genes were upregulated with 279 downregulated in 19Q4–77 *wx*, and 775 were upregulated with 251 downregulated in 19Q4–94 *Wx* (**Supplementary 1**).

**Figure 7:**
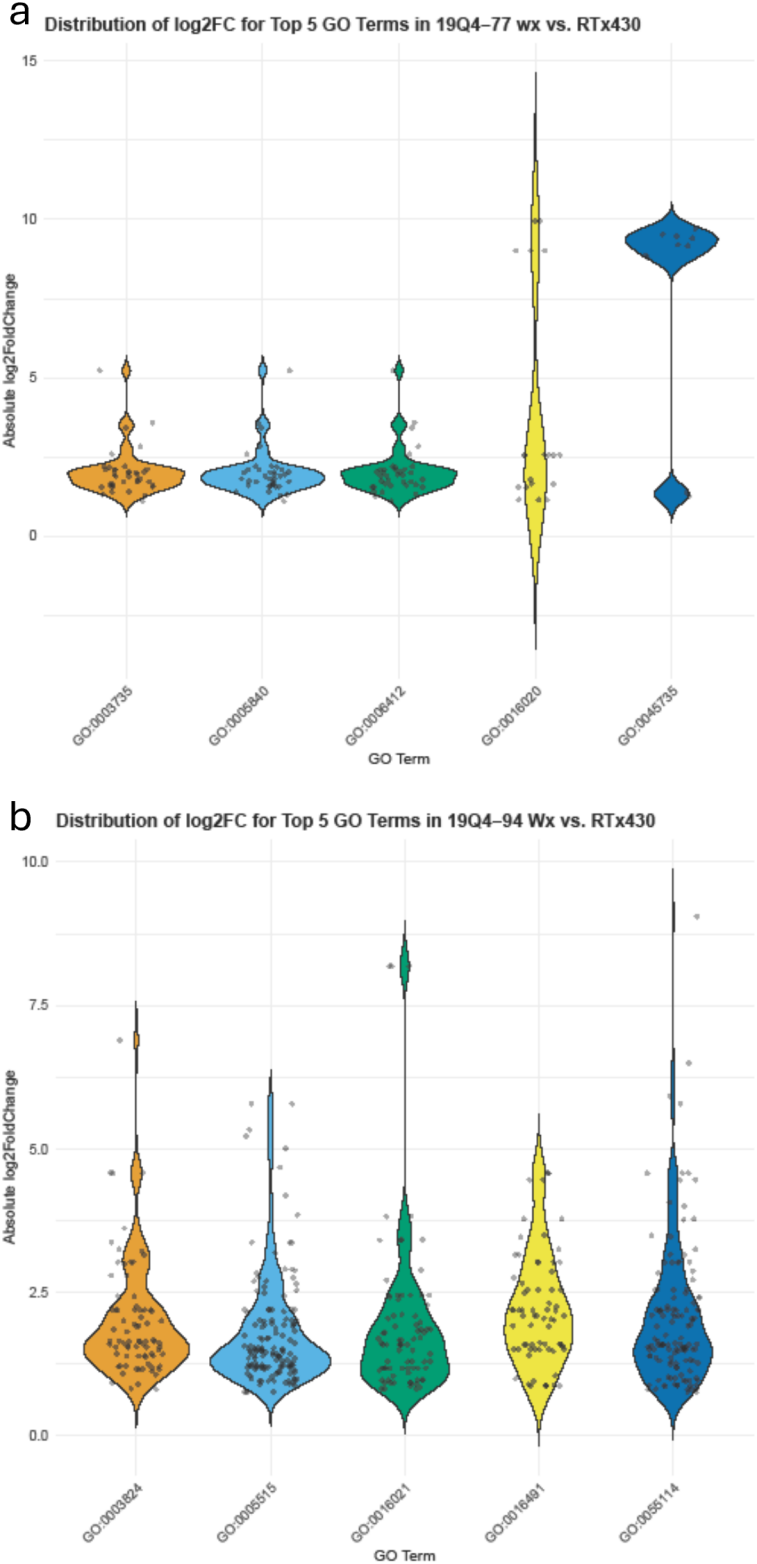
Log2FoldChange in expression of differentially expressed genes in 19Q4–77 *wx* **(A)** and 19Q4–94 *Wx* **(B)** relative to RTx430 [wild type (WT)]. X-axis depicts associated gene ontology (GO) terms.

Non-kafirin DEGs were organized via GO to characterize the shift in transcription activity in edited lines. DEGs in 19Q4–77 *wx* contained GO-tagged terms corresponding to GO:0005840, GO:0003735, GO:0006412, GO:0016020, and GO:0045735 (listed by abundance and degree of log2FoldChange of tagged genes in descending order) in greater abundance than any other GO terms. GO:0005840, GO:0003735, and GO:0006412 all had almost exclusively DEGs clustered <2.5 log2FoldChange, with GO:0016020 and GO:0045735 containing bimodally clustering DEGs approximately 2.5 and 9 absolute log2FoldChange. Additionally, granule-bound starch synthase (SbiRTX430.10G024600) downregulated −7.92 log2FoldChange in 19Q4–77 *wx* due to the *waxya* mutation and, as expected, was not differentially expressed in 19Q4–94 *Wx*. In 19Q4–94 *Wx*, these terms were GO:0005515, GO:0055114, GO:0003677, GO:00016021, and GO:0003824. The expression of these terms is depicted in **Figure 7**, with the majority of the differential expression of these GO terms changing by 1 and 2 absolute log2FoldChange, with notable exceptions in GO:0003735 and GO:0016020 in 19Q4–77 *wx* (**Figure 7a**), which were tagged to several genes near an absolute log2FoldChange value of 10. In 19Q4–77 *wx*, the top three most represented GO terms in DEGs were related to the ribosome (GO:0005840), ribosomal subunits (GO:0003735), and translation (GO:0006412), with the remaining GO terms primarily denoting molecular function. In 19Q4–94 *Wx*, the most genes differentially regulated were tagged with GO:0005515, followed by GO:0055114, and then finally by GO:0003677, which correspond to protein binding, obsolete oxidation–reduction process, and DNA binding (**Figure 7b**). The DEGs tagged with the most represented GO terms by abundance and log2FoldChange were primarily clustered <2.5 absolute log2FoldChange. The top 10 represented GO terms can be viewed in **Table 1**.

**Figure 8:**
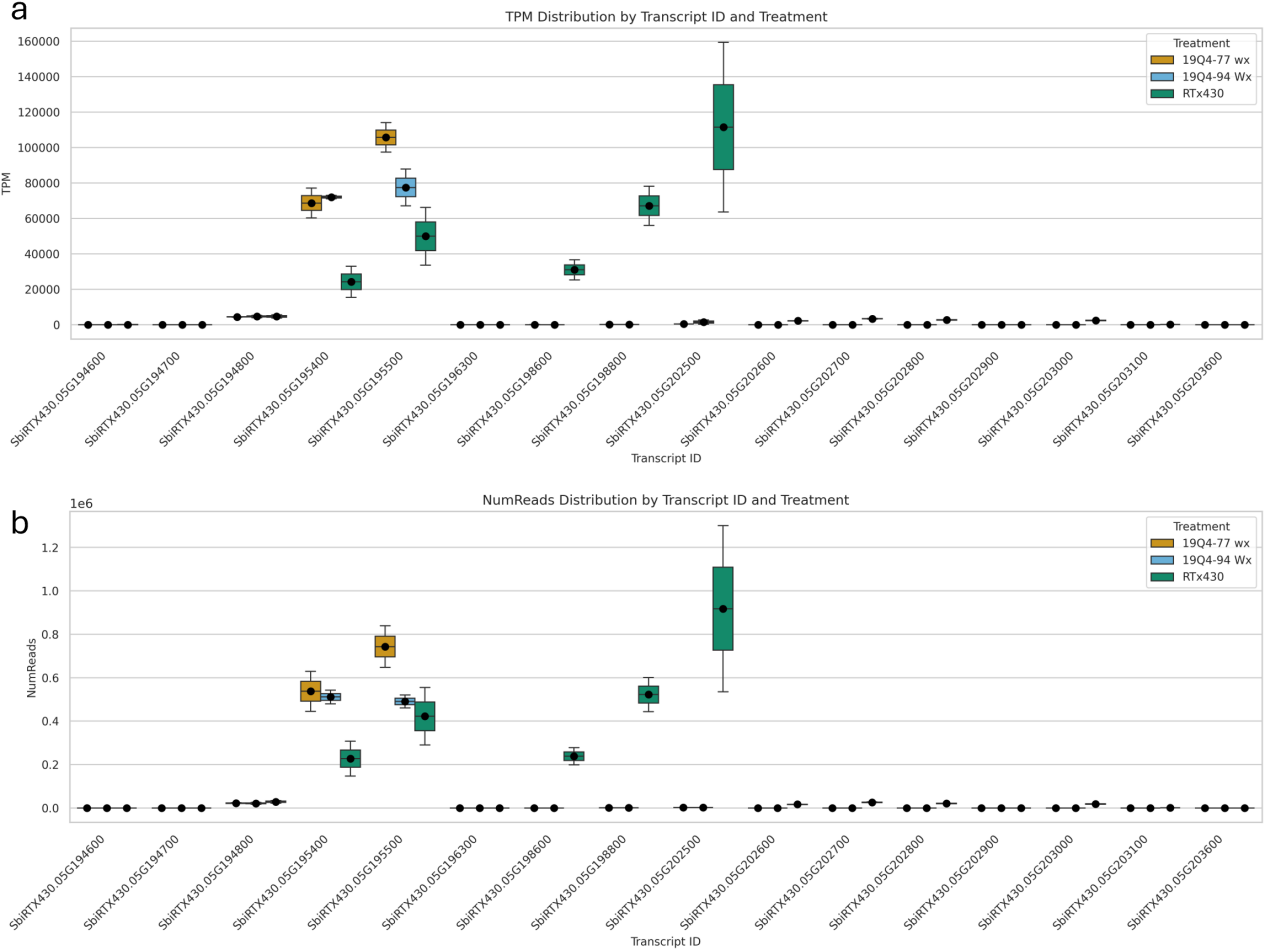
Expression of *k1C* genes by transcript per million **(A)** and read number **(B)** in 19Q4–77 *wx*, 19Q4–94 *Wx*, and RTx430 [wild type (WT)].

A total of 16 *k1C* genes were identified using BLAST of annotated FASTA sequences identified from differential gene expression to *S. bicolor* BTx642 v1.1 with 100% alignment lengths. Of these 16 genes, nine were downregulated in 19Q4–77 *wx*, with eight downregulated in 19Q4–94 *Wx*. The expression of these *k1C* genes is depicted in **Figure 8**. Five *k1C* genes (SbiRTX430.05G195400, SbiRTX430.05G195500, SbiRTX430.05G198600, SbiRTX430.05G198800, and SbiRTX430.05G202500) had much greater expression abundance than the remaining *k1C* genes in RTx430. SbiRTX430.05G195400 and SbiRTX430.05G195500 had modest increases in expression in edited 19Q4–77 *wx* and 19Q4–94 *Wx*. Six of the *k1C* genes identified had no expression, with the remaining five having very low expression in RTx430. All eight of the downregulated *k1C* genes in 19Q4–94 *Wx* were contained within the Chromosome 5 genomic deletion and had >−8 log2FoldChange reduction in expression (**Table 2B**). The same genes and downregulation were observed in 19Q4–77 *wx* in addition to a *k1C* gene outside of the deletion with a −1.38 log2FoldChange (**Table 2A**).

## Discussion

### The k1C deletion lines had near-normal alpha-kafirin abundance and vitreous endosperm

Lines 19Q4–77 *wx* and 19Q4–94 *Wx*, with the ~400 kb of *k1C* region deleted, exhibited little evidence of decreased kafirin expression or proteome rebalancing based on SDS–PAGE analysis. Additionally, both 19Q4–77 *wx* and 19Q4–94 *Wx* had a significantly higher level of protein-bound proline, which is not indicative of a decrease in kafirins. However, a significant increase in protein-bound lysine was found in 19Q4–77 *wx*. The greater increase in protein-bound amino acids in 19Q4–77 *wx* appeared to be due in large part to the increase in total seed protein and subsequent decrease in total seed starch, with the more modest increases in protein-bound amino acids in 19Q4–94 *Wx* reflective of a seed protein content comparable to unedited RTx430.

Kafirin protein content is associated with the hardening of the endosperm. We observed a marginal increase in the proportion of floury endosperm relative to vitreous endosperm in edited lines compared to unedited RTx430 (**Figure 4**). Kafirin expression is approximately 20% greater in the vitreous endosperm of sorghum compared to the floury endosperm (Shull et al., 1992). The increase in floury endosperm suggests that there is some modification in the kafirin expression of 19Q4–77 *wx* and 19Q4–94 *Wx*. However, a decrease in grain test weight was not observed, as is observed in *floury2* maize grain, which is associated with an increase in floury endosperm and a reduction in prolamin expression (**Figure 5c**) (Paez and Zuber, 1973). Furthermore, we did not identify a reduction in the corresponding 22-kDa kafirin proteins in either edited line compared to wild-type RTx430 (**Figures 3a, b**). Finally, there was no obvious increase in the non-kafirin proteins of 19Q4–77 *wx* or 19Q4–94 *Wx* compared to RTx430, which would accompany a reduction of kafirins (**Figures 3c, d**).

The greater degree of irregularities and invaginations observed in 19Q4–77 *wx* and 19Q4–94 *Wx* suggest that the penetration of these protein bodies by gastric proteases may be more successful, thus facilitating increased protein digestibility compared to RTx430 (**Figure 4**). The degree to which this morphological change in protein bodies will increase protein digestibility was not experimentally validated in our study, but prior publications have demonstrated this link between protein body structure and protein digestibility. For example, Oria et al. reported on the extreme change in protein body morphology in the hdhl sorghum line P721Q (2000). This extreme modification to P721Q protein bodies was attributed to an extreme reduction in *k1C* subfamily expression (Wu et al., 2013). The protein bodies of 19Q4–77 *wx* and 19Q4–94 *Wx* did not exhibit an extreme change in morphology, nor were they visually identical to the protein bodies of unedited RTx430. This moderate change in protein body morphology was expected to be greater due to the large proportion of *k1C* genes deleted in edited lines. While not a direct comparison due to the different microscopy techniques employed, protein body morphology appeared more extreme in initial transformants than in the edited lines reported here. Li et al. reported increased protein digestibility with these highly modified protein bodies, and, while not experimentally validated in this study, we would expect a less severe increase in protein digestibility in 19Q4–77 *wx* and 19Q4–94 *Wx*.

Our SDS–PAGE and protein body morphology results were not aligned with our expectations for 19Q4–77 *wx* or 19Q4–94 *Wx*. The presence of such a large deletion in the *k1C* family was expected to produce a drastic reduction in 22-kDa kafirin protein abundance and a dramatic increase in the lysine-containing non-kafirin protein fraction. The reduction in alpha-kafirins would cause malformed protein bodies to form, which would indicate increased protein digestibility as described by Oria et al. (2000). Upon discovering that both 19Q4–77 *wx* and 19Q4–94 *Wx* display near wild-type kafirin and non-kafirin protein contents and protein body morphology phenotypes, we determined that long-read sequencing was needed to ensure that our characterization of the edited *k1C* family was accurate before proceeding with biophysical dough characterization. Since the *k1C* family contains several tandem duplications, the sequence is highly repetitive for both intergenic regions and for gene members. This increases the likelihood of read misalignments and poor read coverage when employing short-read genomic sequencing techniques, which had been preliminarily used to characterize CRISPR edits in these lines.

### 19Q4–77 *wx* protein–starch tradeoff and the resulting amino acid profile resulted in increased total protein in sorghum

We expected a drastic shift in the proteome, favoring increased expression of non-kafirins and a decrease in 22-kDa kafirin expression. A reduction in protein-bound proline in edited lines would be indicative of this phenomenon. Sorghum grain is typically high in proline due to the abundance of prolamin storage proteins in seed endosperm with high proportions of non-polar amino acids, including proline (De Mesa-Stonestreet et al., 2010). If a reduction in kafirin protein was achieved, we would expect to observe a decrease in proline, which we did not observe in either edited line. The increase in protein-bound proline in both edited lines suggests that the *k1C* family is somehow able to compensate for the CRISPR/Cas9 editing. However, we observed not just an increase in protein-bound proline but also an increase in every amino acid in the protein-bound fraction of each line’s flour in 19Q4–77 *wx* and for all except for Asx, Lys, and Met in 19Q4–94 *Wx*. 19Q4–77 *wx* and 19Q4-13 (Hurst et al., 2023) both contained similar levels of protein-bound lysine (22.04 and 21.00 nMol/mg, respectively). Both edited lines described in this study contained free lysine in no greater quantity than wild-type RTx430, unlike 19Q4-13 (Hurst et al., 2023). While a few protein-bound amino acids in 19Q4–94 *Wx* were not increased, and no free amino acids were increased, none were lower than those in RTx430. In 19Q4–77 *wx*, all protein-bound and some free amino acids were increased with no reduction in any amino acids. SbiRTX430.04G339300 is a lysine-ketoglutarate reductase/saccharopine dehydrogenase bifunctional enzyme that catabolizes free lysine (Kemper et al., 1999). This enzyme, whose maize orthologue is regulated by the *Opaque2* transcription factor, was not differentially expressed in 19Q4–77 *wx* and was downregulated in 19Q4–94 *Wx*, with both edited lines having no difference in free lysine content. This may suggest a lack of increased lysine catabolism in 19Q4–77 *wx* and 19Q4–94 *Wx*, which likely contributed to the wild-type levels of free lysine in these edited lines. The increase in amino acids and protein and the reduction in total starch with a greater proportion of amylopectin from the *waxya* line 19Q4–77 *wx* were the only differing macronutrient phenotypes observed compared to 19Q4–94 *Wx*. We observed no differences in kafirin and non-kafirin protein fractions, 100-seed weight, or protein body morphology between these two lines. The increased protein-bound and free amino acids (particularly lysine) as well as the increased total seed protein in 19Q4–77 *wx* were consistent with the increased amino acids and total seed protein observed in initial lines as reported by Li et al. (2018). In 19Q4-13, total seed protein was not measured, but the increase in amino acids (again, particularly for lysine) was also observed (Hurst et al., 2023).

### *k1C* transcription was primarily attributed to five genes

With WGS and Iso-Seq confirming the deletion of seven active *k1C* genes, it was evident that the *k1C* subfamily is compensating for kafirin expression. At the transcriptional level, the five most significantly expressed *k1C* genes in unedited RTx430 were SbiRTX430.05G195400, SbiRTX430.05G195500, SbiRTX430.05G198600, SbiRTX430.05G198800, and SbiRTX430.05G202500 with average NumRead values of 2 × 10^5^ > 8 × 10^5^ and average TPM values of 20,000 > 100,000. Three of these highly expressed *k1C* genes were deleted in 19Q4–77 *wx* and 19Q4–94 *Wx*, with only SbiRTX430.05G195400 and SbiRTX430.05G195500 as substantially transcriptionally active *k1C* genes in these lines. Only five of the remaining 11 (four of which were deleted in 19Q4–77 *wx* and 19Q4–94 *Wx*) had any, albeit low, expression in RTx430, with the remaining six *k1C* genes identified via alignment with *S. bicolor* BTx642 v1.1 being non-expressed. Although the remaining two substantially transcriptionally active *k1C* genes not deleted in 19Q4–77 *wx* and 19Q4–94 *Wx* did not have significantly increased NumRead or TPM values, the low proportion of *k1C* genes with substantial transcription suggests that a consistent and balanced transcription rate across *k1C* members was not necessary for normal *k1C* expression. Of the differentially expressed genes in edited lines, the most common GO slim category in 19Q4–77 *wx* was for cellular component, and that in 19Q4–94 *Wx* was for molecular function. Ribosome and membrane-associated proteins were the most represented cellular component-tagged GO terms in 19Q4–77 *wx*; in 19Q4–94 *Wx*, binding and catalytic activity were the most represented molecular function-tagged GO terms.

### Discrepancies in 22-kDa kafirin expression between initial transformants and inbred lines

Although the initial transformants exhibited reduced 22-kDa kafirin protein with compensatory increases in non-kafirin protein according to SDS–PAGE (Li et al., 2018), we did not observe this in either 19Q4–77 *wx* or 19Q4–94 *Wx*. However, we did observe some phenotypes shared by the inbreds from this study and the initial transformants, such as an increase in essential amino acids and an increase in total seed protein. Hurst et al. described a 1.35-kb duplicated translocation of the *k1C* sequence in 19Q4–13 that reinserted at the gRNA target site (Hurst et al., 2023). The potential for this phenomenon to explain the lack of hdhl-like proteome rebalancing in 19Q4–77 *wx* in 19Q4–94 *Wx* prompted the use of PacBio long-read sequencing. Having ruled out the possibility that duplicated translocations caused by non-homologous end joining repair from CRISPR and the high sequence similarity of the *k1C* family could have generated novel, active alpha-kafirin genes, we can only speculate as to why these phenotypes differ.

One such explanation for this difference in phenotypes is that a dominant negative phenotype was generated in one or multiple *k1C* members in the initial transformants not fully characterized by the short-read genomic sequencing performed. This mutation could have, in a similar fashion to the P721Q mutant, drastically affected the expression of the *k1C* family, resulting in the non-kafirin proteome rebalancing seen in initial transformants. Selection for the ~400kb deletion could have selected against this dominant negative mutation, resulting in lines where the deletion did not result in a classical hdhl-like non-kafirin proteome rebalance.

## Conclusion

In conclusion, the use of a consensus-based targeting strategy using CRISPR/Cas9 may not produce consistently predictable proteome rebalancing in a gene family such as *k1C* with a high degree of gene sequence and intergenic sequence similarity. Although we demonstrated the ability of this strategy in yielding high-lysine, high-protein sorghum, the simplicity of this expression may need to be traded for a more complex editing strategy with more individualized targets for increased consistency. Additionally, we demonstrate the resilience of the *k1C* subfamily in its ability to withstand the induction of large structural variations. Finally, these results suggest that continued investigation into proteome rebalancing in cereal grains is necessary for functional improvement in the nutritional and biophysical traits of these crop species without sacrificing important end-use traits.

## Data Availability

Any and all data reported in this publication can be accessed by contacting the corresponding author, David R. Holding. The sequencing reads used in this study are deposited in NCBI, accession PRJNA1285952.
