# Supplementary material for: Large scale deletion and rebalancing within the k1C kafirin family in sorghum
Source: Front Plant Sci. 2025 Oct 23;16:1686027. doi: 10.3389/fpls.2025.1686027 (PMC12588965; doi:10.3389/fpls.2025.1686027)
Supplement: Supplementary file 1 [file DataSheet1.pdf]

#pacID	locusName	transcriptN	peptideNa	Pfam	Panther	ec	KOG	KO	GO
PAC:43581	SbiRTX430	SbiRTX430	SbiRTX430.01G023400.1.p						
PAC:43580	SbiRTX430	SbiRTX430	SbiRTX430.01G02610		PTHR31184	PTHR3118	KOG3450		
PAC:43581	SbiRTX430	SbiRTX430	SbiRTX430.01G04190		PTHR31704	PTHR31704:SF25			
PAC:43578	SbiRTX430	SbiRTX430	SbiRTX430	PF00444	PTHR18804	PTHR1880	KOG4122	K02919	GO:00037
PAC:43581	SbiRTX430	SbiRTX430	SbiRTX430.01G04650		PTHR36360	PTHR36360:SF1			
PAC:43583	SbiRTX430	SbiRTX430	SbiRTX430	PF03169	PTHR22601	PTHR22601:SF2			GO:00550
PAC:43580	SbiRTX430	SbiRTX430	SbiRTX430	PF04241	PTHR10584		KOG3472		
PAC:43577	SbiRTX430	SbiRTX430	SbiRTX430.01G091300.1.p						
PAC:43582	SbiRTX430	SbiRTX430	SbiRTX430	PF00125 P	PTHR23430		KOG1757	K11251	GO:00007
PAC:43577	SbiRTX430	SbiRTX430	SbiRTX430.01G10370		PTHR33832	PTHR33832:SF5			
PAC:43578	SbiRTX430	SbiRTX430	SbiRTX430.01G128200.1.p						
PAC:43578	SbiRTX430	SbiRTX430	SbiRTX430.01G128200.2.p						
PAC:43579	SbiRTX430	SbiRTX430	SbiRTX430	PF01165	PTHR37228	PTHR37228:SF1		K02970	GO:00037
PAC:43577	SbiRTX430	SbiRTX430	SbiRTX430	PF01249	PTHR10442		KOG3486	K02971	GO:00037
PAC:43579	SbiRTX430	SbiRTX430	SbiRTX430.01G16810		PTHR35999				
PAC:43579	SbiRTX430	SbiRTX430	SbiRTX430.01G16810		PTHR35999				
PAC:43579	SbiRTX430	SbiRTX430	SbiRTX430	PF03171 P	PTHR10209	PTHR1020	KOG0143		GO:00055
PAC:43579	SbiRTX430	SbiRTX430	SbiRTX430	PF00173	PTHR19359	PTHR1935	KOG0537		
PAC:43579	SbiRTX430	SbiRTX430	SbiRTX430	PF00173	PTHR19359	PTHR1935	KOG0537		
PAC:43577	SbiRTX430	SbiRTX430	SbiRTX430.01G22350		PTHR37720	PTHR37720:SF1			
PAC:43577	SbiRTX430	SbiRTX430	SbiRTX430.01G22350		PTHR37720	PTHR37720:SF1			
PAC:43580	SbiRTX430	SbiRTX430	SbiRTX430	PF01157	PTHR20981		KOG1732	K02889	GO:00037
PAC:43583	SbiRTX430	SbiRTX430	SbiRTX430.01G242000.1.p						
PAC:43584	SbiRTX430	SbiRTX430	SbiRTX430	PF08783 P	PTHR15439	PTHR1543	KOG0314		GO:00036
PAC:43584	SbiRTX430	SbiRTX430	SbiRTX430	PF08783 P	PTHR15439	PTHR1543	KOG0314	K10624	GO:00036
PAC:43584	SbiRTX430	SbiRTX430	SbiRTX430	PF08783 P	PTHR15439	PTHR1543	KOG0314	K10624	GO:00036
PAC:43583	SbiRTX430	SbiRTX430	SbiRTX430	PF03018	PTHR21495	PTHR21495:SF46			
PAC:43578	SbiRTX430	SbiRTX430	SbiRTX430	PF04504					
PAC:43583	SbiRTX430	SbiRTX430	SbiRTX430	PF12874	PTHR23067	PTHR23067:SF41			GO:00036
PAC:43581	SbiRTX430	SbiRTX430	SbiRTX430.01G288800.1.p						
PAC:43583	SbiRTX430	SbiRTX430	SbiRTX430	PF05899	PTHR33271	PTHR33271:SF7			GO:00036
PAC:43577	SbiRTX430	SbiRTX430	SbiRTX430.01G30020		PTHR37387	PTHR37387:SF1			
PAC:43577	SbiRTX430	SbiRTX430	SbiRTX430.01G30020		PTHR37387	PTHR37387:SF1			
PAC:43578	SbiRTX430	SbiRTX430	SbiRTX430	PF13639	PTHR14155	PTHR14155:SF187			GO:00052
PAC:43581	SbiRTX430	SbiRTX430	SbiRTX430	PF15511	PTHR10484		KOG3467	K11254	GO:00007
PAC:43584	SbiRTX430	SbiRTX430	SbiRTX430	PF04043					GO:00048
PAC:43578	SbiRTX430	SbiRTX430	SbiRTX430.01G35390		PTHR33403			K18635	
PAC:43578	SbiRTX430	SbiRTX430	SbiRTX430.01G35390		PTHR33403			K18635	
PAC:43580	SbiRTX430	SbiRTX430	SbiRTX430.01G35530		PTHR37760				GO:00310
PAC:43582	SbiRTX430	SbiRTX430	SbiRTX430	PF01907	PTHR10768		KOG3475	K02922	GO:00037
PAC:43581	SbiRTX430	SbiRTX430	SbiRTX430	PF01249	PTHR10442		KOG3486	K02971	GO:00037
PAC:43581	SbiRTX430	SbiRTX430	SbiRTX430	PF01249	PTHR10442		KOG3486	K02971	GO:00037
PAC:43577	SbiRTX430	SbiRTX430	SbiRTX430.01G394200.1.p						

PAC:43577	SbiRTX430	SbiRTX430	SbiRTX430.01G394200.2.p						
PAC:43579	SbiRTX430	SbiRTX430	SbiRTX430	PF11969	PTHR23089	PTHR2308	KOG3275	K02503	GO:000382
PAC:43578	SbiRTX430	SbiRTX430	SbiRTX430.01G413500.1.p						GO:001602
PAC:43578	SbiRTX430	SbiRTX430	SbiRTX430.01G413500.2.p						GO:001602
PAC:43577	SbiRTX430	SbiRTX430	SbiRTX430	PF04667	PTHR34804	PTHR34804:SF1			
PAC:43576	SbiRTX430	SbiRTX430	SbiRTX430	PF00407	PTHR31213	PTHR31213:SF20			GO:000695
PAC:43578	SbiRTX430	SbiRTX430	SbiRTX430	PF01200	PTHR10769		KOG3502	K02979	GO:000373
PAC:43578	SbiRTX430	SbiRTX430	SbiRTX430	PF01200	PTHR10769		KOG3502		GO:000373
PAC:43578	SbiRTX430	SbiRTX430	SbiRTX430	PF00076	PTHR24012				GO:000016
PAC:43581	SbiRTX430	SbiRTX430	SbiRTX430	PF06127	PTHR34205	PTHR34205:SF2			GO:001649
PAC:43577	SbiRTX430	SbiRTX430	SbiRTX430	PF05347	PTHR13166	PTHR1316	KOG3801		
PAC:43577	SbiRTX430	SbiRTX430	SbiRTX430	PF00241	PTHR11913	PTHR1191	KOG1735	K05765	GO:000377
PAC:43577	SbiRTX430	SbiRTX430	SbiRTX430	PF00241	PTHR11913	PTHR11913:SF16			GO:000377
PAC:43580	SbiRTX430	SbiRTX430	SbiRTX430	PF02574	PTHR2109	EC:2.1.1.1	KOG1579	K00547	
PAC:43583	SbiRTX430	SbiRTX430	SbiRTX430	PF00190	PTHR31189	PTHR31189:SF7			GO:004573
PAC:43583	SbiRTX430	SbiRTX430	SbiRTX430	PF00916 P	PTHR11814	PTHR11814:SF101		K17470	GO:000827
PAC:43583	SbiRTX430	SbiRTX430	SbiRTX430	PF00916 P	PTHR11814	PTHR11814:SF101		K17470	GO:000827
PAC:43583	SbiRTX430	SbiRTX430	SbiRTX430	PF00916 P	PTHR11814	PTHR11814:SF101		K17470	GO:000827
PAC:43583	SbiRTX430	SbiRTX430	SbiRTX430	PF00916 P	PTHR11814	PTHR11814:SF101		K17470	GO:000827
PAC:43583	SbiRTX430	SbiRTX430	SbiRTX430	PF00916 P	PTHR11814	PTHR11814:SF101		K17470	GO:000827
PAC:43583	SbiRTX430	SbiRTX430	SbiRTX430	PF00916 P	PTHR11814	PTHR11814:SF101		K17470	GO:000827
PAC:43583	SbiRTX430	SbiRTX430	SbiRTX430.01G50420		PTHR35548	PTHR35548:SF1			
PAC:43583	SbiRTX430	SbiRTX430	SbiRTX430.01G50420		PTHR35548				
PAC:43580	SbiRTX430	SbiRTX430	SbiRTX430	PF12697	PTHR1099	EC:3.1.1.3	KOG1454		
PAC:43579	SbiRTX430	SbiRTX430	SbiRTX430	PF01200	PTHR10769		KOG3502	K02979	GO:000373
PAC:43581	SbiRTX430	SbiRTX430	SbiRTX430	PF00515	PTHR36326	PTHR36326:SF1			GO:000551
PAC:43581	SbiRTX430	SbiRTX430	SbiRTX430	PF00304	PTHR33147	PTHR33147:SF9			GO:000695
PAC:43579	SbiRTX430	SbiRTX430	SbiRTX430.01G56520		PTHR33214	PTHR33214:SF1			
PAC:43577	SbiRTX430	SbiRTX430	SbiRTX430	PF00234	PTHR31731	PTHR31731:SF5			
PAC:43577	SbiRTX430	SbiRTX430	SbiRTX430	PF00234	PTHR31731	PTHR31731:SF5			
PAC:43577	SbiRTX430	SbiRTX430	SbiRTX430	PF00234	PTHR31731	PTHR31731:SF5			
PAC:43559	SbiRTX430	SbiRTX430	SbiRTX430.02G03410		PTHR14154	PTHR14154:SF20			
PAC:43559	SbiRTX430	SbiRTX430	SbiRTX430.02G03410		PTHR14154	PTHR14154:SF20			
PAC:43555	SbiRTX430	SbiRTX430	SbiRTX430.02G069200.1.p						
PAC:43558	SbiRTX430	SbiRTX430	SbiRTX430	PF03179	PTHR12713		KOG1772	K02152	GO:000372
PAC:43558	SbiRTX430	SbiRTX430	SbiRTX430	PF03179	PTHR12713		KOG1772	K02152	GO:000372
PAC:43558	SbiRTX430	SbiRTX430	SbiRTX430	PF03179	PTHR12713		KOG1772	K02152	GO:000372
PAC:43556	SbiRTX430	SbiRTX430	SbiRTX430	PF13415 P	PTHR23244	PTHR23244:SF276			GO:000551
PAC:43558	SbiRTX430	SbiRTX430	SbiRTX430	PF00161 PF00652		EC:3.2.2.22			GO:001714
PAC:43558	SbiRTX430	SbiRTX430	SbiRTX430	PF00161 PF00652		EC:3.2.2.22			GO:001714
PAC:43555	SbiRTX430	SbiRTX430	SbiRTX430	PF00304	PTHR33147	PTHR33147:SF13			GO:000695
PAC:43556	SbiRTX430	SbiRTX430	SbiRTX430.02G114600.1.p						
PAC:43561	SbiRTX430	SbiRTX430	SbiRTX430	PF03604	PTHR12056		KOG3507	K03009	GO:000367
PAC:43556	SbiRTX430	SbiRTX430	SbiRTX430.02G13850		PTHR36856	PTHR36856:SF1			

PAC:43559	SbiRTX430	SbiRTX430	SbiRTX430.02G185000.1.p						
PAC:43557	SbiRTX430	SbiRTX430	SbiRTX430	PF01247	PTHR10902		KOG0887	K02917	GO:000373
PAC:43557	SbiRTX430	SbiRTX430	SbiRTX430	PF01247	PTHR10902		KOG0887	K02917	GO:000373
PAC:43559	SbiRTX430	SbiRTX430	SbiRTX430	PF12937					GO:000551
PAC:43556	SbiRTX430	SbiRTX430	SbiRTX430.02G190500.1.p						
PAC:43556	SbiRTX430	SbiRTX430	SbiRTX430.02G190500.2.p						
PAC:43556	SbiRTX430	SbiRTX430	SbiRTX430.02G190500.3.p						
PAC:43556	SbiRTX430	SbiRTX430	SbiRTX430	PF04588	PTHR12297		KOG4431		
PAC:43558	SbiRTX430	SbiRTX430	SbiRTX430.02G19370		PTHR33124				GO:004698
PAC:43559	SbiRTX430	SbiRTX430	SbiRTX430.02G19670		PTHR35987	PTHR35987:SF2			
PAC:43561	SbiRTX430	SbiRTX430	SbiRTX430	PF04828	PTHR28620	PTHR2862	KOG4192		GO:000815
PAC:43556	SbiRTX430	SbiRTX430	SbiRTX430	PF02704	PTHR23201	PTHR23201:SF14			
PAC:43558	SbiRTX430	SbiRTX430	SbiRTX430	PF00847	PTHR31190	PTHR31190:SF8		K09286	GO:000367
PAC:43558	SbiRTX430	SbiRTX430	SbiRTX430	PF00847	PTHR31190	PTHR31190:SF8		K09286	GO:000367
PAC:43558	SbiRTX430	SbiRTX430	SbiRTX430	PF00847	PTHR31190	PTHR31190:SF8			GO:000367
PAC:43556	SbiRTX430	SbiRTX430	SbiRTX430.02G22470		PTHR37207				
PAC:43557	SbiRTX430	SbiRTX430	SbiRTX430.02G24340		PTHR35420				
PAC:43561	SbiRTX430	SbiRTX430	SbiRTX430	PF01241	PTHR34195	PTHR34195:SF1		K08905	GO:000952
PAC:43558	SbiRTX430	SbiRTX430	SbiRTX430	PF00067	PTHR2429	EC:1.14.13	KOG0156		GO:000449
PAC:43558	SbiRTX430	SbiRTX430	SbiRTX430.02G32380		PTHR35687				
PAC:43560	SbiRTX430	SbiRTX430	SbiRTX430	PF01253	PTHR10388	PTHR1038	KOG1770	K03113	GO:000374
PAC:43560	SbiRTX430	SbiRTX430	SbiRTX430	PF01253	PTHR10388	PTHR1038	KOG1770	K03113	GO:000374
PAC:43560	SbiRTX430	SbiRTX430	SbiRTX430	PF01253	PTHR10388	PTHR1038	KOG1770	K03113	GO:000374
PAC:43560	SbiRTX430	SbiRTX430	SbiRTX430	PF01253	PTHR10388	PTHR1038	KOG1770		GO:000374
PAC:43560	SbiRTX430	SbiRTX430	SbiRTX430	PF01253	PTHR10388	PTHR10388:SF14			GO:000374
PAC:43556	SbiRTX430	SbiRTX430	SbiRTX430	PF01781	PTHR10965	PTHR1096	KOG3499		GO:000373
PAC:43556	SbiRTX430	SbiRTX430	SbiRTX430	PF01781	PTHR10965	PTHR1096	KOG3499	K02923	GO:000373
PAC:43558	SbiRTX430	SbiRTX430	SbiRTX430.02G34440		PTHR36385				
PAC:43556	SbiRTX430	SbiRTX430	SbiRTX430	PF00304	PTHR33147	PTHR33147:SF7			GO:000695
PAC:43559	SbiRTX430	SbiRTX430	SbiRTX430.02G35440		PTHR34114	PTHR34114:SF2			
PAC:43559	SbiRTX430	SbiRTX430	SbiRTX430.02G35440		PTHR34114				
PAC:43558	SbiRTX430	SbiRTX430	SbiRTX430.02G365800.1.p						
PAC:43558	SbiRTX430	SbiRTX430	SbiRTX430.02G365800.2.p						
PAC:43558	SbiRTX430	SbiRTX430	SbiRTX430.02G365800.3.p						
PAC:43555	SbiRTX430	SbiRTX430	SbiRTX430.02G37240		PTHR36724				
PAC:43560	SbiRTX430	SbiRTX430	SbiRTX430	PF13202	PTHR10891	PTHR10891:SF597			GO:000550
PAC:43555	SbiRTX430	SbiRTX430	SbiRTX430	PF10215	PTHR28677				
PAC:43555	SbiRTX430	SbiRTX430	SbiRTX430	PF10215	PTHR28677				
PAC:43558	SbiRTX430	SbiRTX430	SbiRTX430	PF00403	PTHR2409	EC:3.6.3.4	EC:3.6.3.54		GO:003000
PAC:43556	SbiRTX430	SbiRTX430	SbiRTX430.02G39130		PTHR34776	PTHR34776:SF1			
PAC:43557	SbiRTX430	SbiRTX430	SbiRTX430.02G39460		PTHR34565	PTHR34565:SF1			
PAC:43560	SbiRTX430	SbiRTX430	SbiRTX430	PF05839	PTHR28672	PTHR28672:SF1		K12456	
PAC:43560	SbiRTX430	SbiRTX430	SbiRTX430	PF05839	PTHR28672	PTHR28672:SF1		K12456	
PAC:43560	SbiRTX430	SbiRTX430	SbiRTX430.02G401300.1.p						

PAC:43558	SbiRTX430	SbiRTX430	SbiRTX430.02G40520(		PTHR33976	PTHR33976:SF1			
PAC:43544	SbiRTX430	SbiRTX430	SbiRTX430.03G027100.1.p						
PAC:43543	SbiRTX430	SbiRTX430	SbiRTX430	PF06596	PTHR34455	PTHR34455:SF2			GO:000952
PAC:43543	SbiRTX430	SbiRTX430	SbiRTX430	PF15511	PTHR10484		KOG3467	K11254	GO:000078
PAC:43544	SbiRTX430	SbiRTX430	SbiRTX430.03G09170(		PTHR37378	PTHR37378:SF2			GO:000486
PAC:43541	SbiRTX430	SbiRTX430	SbiRTX430.03G096700.1.p						
PAC:43538	SbiRTX430	SbiRTX430	SbiRTX430.03G113500.1.p						
PAC:43541	SbiRTX430	SbiRTX430	SbiRTX430.03G11360(		PTHR31257	PTHR31257:SF5			
PAC:43541	SbiRTX430	SbiRTX430	SbiRTX430.03G11360(		PTHR31257				
PAC:43542	SbiRTX430	SbiRTX430	SbiRTX430	PF01221	PTHR1188(	EC:3.6.4.2			GO:000587
PAC:43542	SbiRTX430	SbiRTX430	SbiRTX430	PF01221	PTHR1188(	EC:3.6.4.2	KOG3430	K10418	GO:000587
PAC:43540	SbiRTX430	SbiRTX430	SbiRTX430	PF05160	PTHR16771	PTHR16771:SF2		K10881	
PAC:43540	SbiRTX430	SbiRTX430	SbiRTX430	PF05160	PTHR16771	PTHR16771:SF2		K10881	
PAC:43544	SbiRTX430	SbiRTX430	SbiRTX430	PF01679	PTHR21659	PTHR2165	KOG1773		GO:001602
PAC:43544	SbiRTX430	SbiRTX430	SbiRTX430	PF01679	PTHR21659	PTHR2165	KOG1773		GO:001602
PAC:43543	SbiRTX430	SbiRTX430	SbiRTX430.03G164000.1.p						GO:000551
PAC:43539	SbiRTX430	SbiRTX430	SbiRTX430.03G183200.1.p						
PAC:43542	SbiRTX430	SbiRTX430	SbiRTX430	PF00507	PTHR11058	PTHR1105	KOG4662	K05574	GO:000813
PAC:43538	SbiRTX430	SbiRTX430	SbiRTX430	PF03911	PTHR13509	PTHR13509:SF2		K09481	GO:000578
PAC:43540	SbiRTX430	SbiRTX430	SbiRTX430	PF00468	PTHR14503	PTHR14503:SF0		K02914	GO:000373
PAC:43541	SbiRTX430	SbiRTX430	SbiRTX430.03G21730(		PTHR34970	PTHR34970:SF1			
PAC:43544	SbiRTX430	SbiRTX430	SbiRTX430	PF08038	PTHR34944	PTHR3494	KOG4449	K17771	GO:000574
PAC:43540	SbiRTX430	SbiRTX430	SbiRTX430	PF01197	PTHR33280	PTHR33280:SF1		K02909	GO:000373
PAC:43540	SbiRTX430	SbiRTX430	SbiRTX430	PF01197	PTHR33280	PTHR33280:SF1		K02909	GO:000373
PAC:43538	SbiRTX430	SbiRTX430	SbiRTX430.03G256100.1.p						
PAC:43541	SbiRTX430	SbiRTX430	SbiRTX430	PF00179	PTHR2406	EC:6.3.2.1	KOG0417	K06689	
PAC:43544	SbiRTX430	SbiRTX430	SbiRTX430.03G29380(		PTHR36484	PTHR36484:SF1			
PAC:43540	SbiRTX430	SbiRTX430	SbiRTX430.03G30700(		PTHR36896				
PAC:43538	SbiRTX430	SbiRTX430	SbiRTX430.03G31320(		PTHR33156	PTHR33156:SF11			
PAC:43538	SbiRTX430	SbiRTX430	SbiRTX430.03G32100(		PTHR3547	EC:1.6.5.3			
PAC:43543	SbiRTX430	SbiRTX430	SbiRTX430	PF12095	PTHR36803				
PAC:43541	SbiRTX430	SbiRTX430	SbiRTX430	PF07123	PTHR34552				GO:000950
PAC:43541	SbiRTX430	SbiRTX430	SbiRTX430	PF07123	PTHR34552			K02721	GO:000950
PAC:43542	SbiRTX430	SbiRTX430	SbiRTX430.03G341500.1.p						
PAC:43543	SbiRTX430	SbiRTX430	SbiRTX430	PF16845	PTHR11413	PTHR11413:SF56			GO:000486
PAC:43543	SbiRTX430	SbiRTX430	SbiRTX430	PF16845	PTHR11413	PTHR11413:SF56			GO:000486
PAC:43542	SbiRTX430	SbiRTX430	SbiRTX430	PF00234	PTHR33076	PTHR33076:SF1			GO:000686
PAC:43538	SbiRTX430	SbiRTX430	SbiRTX430	PF03222	PTHR22950	PTHR2295	KOG1305		GO:000333
PAC:43538	SbiRTX430	SbiRTX430	SbiRTX430	PF03222	PTHR22950	PTHR2295	KOG1305		GO:000333
PAC:43538	SbiRTX430	SbiRTX430	SbiRTX430	PF01490	PTHR22950	PTHR2295	KOG1305		
PAC:43539	SbiRTX430	SbiRTX430	SbiRTX430	PF10604	PTHR31213	PTHR31213:SF21		K14496	
PAC:43539	SbiRTX430	SbiRTX430	SbiRTX430	PF00125	PTHR23428		KOG1744	K11252	GO:000078
PAC:43542	SbiRTX430	SbiRTX430	SbiRTX430	PF00228	PTHR33479				GO:000486
PAC:43541	SbiRTX430	SbiRTX430	SbiRTX430	PF01423	PTHR13110		KOG3460	K12622	

PAC:43538	SbiRTX430	SbiRTX430	SbiRTX430	PF17067	PTHR34550	PTHR34550:SF1		K19033	GO:000584
PAC:43538	SbiRTX430	SbiRTX430	SbiRTX430	PF17067	PTHR34550	PTHR34550:SF1		K19033	GO:000584
PAC:43541	SbiRTX430	SbiRTX430	SbiRTX430	PF11976	PTHR10562	PTHR1056	KOG1769	K12160	GO:000551
PAC:43538	SbiRTX430	SbiRTX430	SbiRTX430.03G44560		PTHR36399				
PAC:43542	SbiRTX430	SbiRTX430	SbiRTX430	PF14541 P	PTHR1368	EC:3.4.23.1	KOG1339		GO:000419
PAC:43543	SbiRTX430	SbiRTX430	SbiRTX430.03G457200.1.p						
PAC:43543	SbiRTX430	SbiRTX430	SbiRTX430	PF12734	PTHR31568				
PAC:43543	SbiRTX430	SbiRTX430	SbiRTX430	PF04133	PTHR12050		KOG2174		
PAC:43543	SbiRTX430	SbiRTX430	SbiRTX430	PF04133	PTHR12050		KOG2174		
PAC:43544	SbiRTX430	SbiRTX430	SbiRTX430	PF00076	PTHR24012			K13195	GO:000016
PAC:43565	SbiRTX430	SbiRTX430	SbiRTX430	PF04043	PTHR31707	PTHR31707:SF53			GO:000485
PAC:43567	SbiRTX430	SbiRTX430	SbiRTX430	PF00251 P	PTHR3195	EC:3.2.1.26		K01193	GO:000455
PAC:43567	SbiRTX430	SbiRTX430	SbiRTX430	PF00251 P	PTHR3195	EC:3.2.1.2	KOG0228	K01193	GO:000455
PAC:43567	SbiRTX430	SbiRTX430	SbiRTX430.04G01560		PTHR36483	PTHR36483:SF1			
PAC:43569	SbiRTX430	SbiRTX430	SbiRTX430.04G03010		PTHR34283				
PAC:43569	SbiRTX430	SbiRTX430	SbiRTX430	PF01780			KOG0402	K02921	GO:000373
PAC:43568	SbiRTX430	SbiRTX430	SbiRTX430.04G05100		PTHR33157	PTHR33157:SF2			
PAC:43569	SbiRTX430	SbiRTX430	SbiRTX430	PF00584	PTHR12309		KOG3498	K07342	GO:000660
PAC:43566	SbiRTX430	SbiRTX430	SbiRTX430	PF00006 P	PTHR1518	EC:3.6.3.14		K02112	GO:000552
PAC:43569	SbiRTX430	SbiRTX430	SbiRTX430	PF04535	PTHR11615	PTHR11615:SF143			
PAC:43569	SbiRTX430	SbiRTX430	SbiRTX430	PF01282	PTHR10496		KOG3424	K02974	GO:000016
PAC:43567	SbiRTX430	SbiRTX430	SbiRTX430.04G11920		PTHR23241	PTHR2324	KOG4744		
PAC:43567	SbiRTX430	SbiRTX430	SbiRTX430.04G11920		PTHR23241	PTHR23241:SF54			
PAC:43567	SbiRTX430	SbiRTX430	SbiRTX430.04G12030		PTHR35704	PTHR35704:SF2			
PAC:43567	SbiRTX430	SbiRTX430	SbiRTX430.04G15860		PTHR36338				
PAC:43565	SbiRTX430	SbiRTX430	SbiRTX430	PF11820	PTHR33128	PTHR33128:SF13			
PAC:43568	SbiRTX430	SbiRTX430	SbiRTX430	PF03009	PTHR2295	EC:3.1.4.4	KOG2421		GO:000662
PAC:43568	SbiRTX430	SbiRTX430	SbiRTX430	PF03009	PTHR2295	EC:3.1.4.46			GO:000662
PAC:43568	SbiRTX430	SbiRTX430	SbiRTX430	PF03009	PTHR2295	EC:3.1.4.4	KOG2421		GO:000662
PAC:43569	SbiRTX430	SbiRTX430	SbiRTX430	PF01428 P	PTHR10634		KOG3173		GO:000367
PAC:43567	SbiRTX430	SbiRTX430	SbiRTX430.04G177700.1.p						
PAC:43567	SbiRTX430	SbiRTX430	SbiRTX430.04G17770		PTHR33177	PTHR33177:SF4			
PAC:43567	SbiRTX430	SbiRTX430	SbiRTX430.04G17770		PTHR33177	PTHR33177:SF4			
PAC:43569	SbiRTX430	SbiRTX430	SbiRTX430	PF02597	PTHR3335	EC:2.8.1.1	KOG3474	K03635	GO:000677
PAC:43565	SbiRTX430	SbiRTX430	SbiRTX430.04G193800.1.p			EC:5.2.1.8			
PAC:43566	SbiRTX430	SbiRTX430	SbiRTX430.04G193900.1.p			EC:5.2.1.8			
PAC:43569	SbiRTX430	SbiRTX430	SbiRTX430	PF04588	PTHR28018	PTHR28018:SF1			
PAC:43569	SbiRTX430	SbiRTX430	SbiRTX430.04G20880		PTHR28018	PTHR28018:SF1			
PAC:43564	SbiRTX430	SbiRTX430	SbiRTX430.04G21780		PTHR36709	PTHR36709:SF1			
PAC:43564	SbiRTX430	SbiRTX430	SbiRTX430.04G21780		PTHR36709				
PAC:43567	SbiRTX430	SbiRTX430	SbiRTX430.04G233600.1.p						
PAC:43567	SbiRTX430	SbiRTX430	SbiRTX430.04G25840		PTHR12701	PTHR12701:SF12			GO:000578
PAC:43567	SbiRTX430	SbiRTX430	SbiRTX430.04G25840		PTHR12701	PTHR12701:SF12			GO:000578
PAC:43566	SbiRTX430	SbiRTX430	SbiRTX430	PF01248	PTHR23105	PTHR2310	KOG3167	K11129	GO:000372

PAC:43568	SbiRTX430	SbiRTX430	SbiRTX430	PF04720	PTHR31579	PTHR31579:SF15			
PAC:43569	SbiRTX430	SbiRTX430	SbiRTX430	PF00462	PTHR1016	EC:1.8.1.9	KOG1752	K03676	GO:000905
PAC:43566	SbiRTX430	SbiRTX430	SbiRTX430	PF00010	PTHR16223	PTHR16223:SF42			GO:004698
PAC:43566	SbiRTX430	SbiRTX430	SbiRTX430	PF00010	PTHR16223	PTHR16223:SF42			GO:004698
PAC:43567	SbiRTX430	SbiRTX430	SbiRTX430	PF00832	PTHR19970		KOG0002	K02924	GO:000373
PAC:43566	SbiRTX430	SbiRTX430	SbiRTX430.04G35050		PTHR33132				
PAC:43569	SbiRTX430	SbiRTX430	SbiRTX430	PF05348	PTHR12828	PTHR1282	KOG3061	K11599	
PAC:43569	SbiRTX430	SbiRTX430	SbiRTX430	PF00571	PTHR11911	PTHR11911:SF51			
PAC:43575	SbiRTX430	SbiRTX430	SbiRTX430	PF00487 P	PTHR32100	PTHR32100:SF14		K10257	GO:000662
PAC:43575	SbiRTX430	SbiRTX430	SbiRTX430	PF04770	PTHR31948				
PAC:43575	SbiRTX430	SbiRTX430	SbiRTX430	PF00240	PTHR13042		KOG3493	K13113	GO:000551
PAC:43575	SbiRTX430	SbiRTX430	SbiRTX430	PF00240	PTHR13042		KOG3493	K13113	GO:000551
PAC:43573	SbiRTX430	SbiRTX430	SbiRTX430.05G066800.1.p						
PAC:43576	SbiRTX430	SbiRTX430	SbiRTX430.05G073500.1.p						
PAC:43576	SbiRTX430	SbiRTX430	SbiRTX430	PF03936	PTHR3122	EC:4.2.3.22	EC:4.2.3.75		GO:000028
PAC:43575	SbiRTX430	SbiRTX430	SbiRTX430.05G09650		PTHR33157				
PAC:43575	SbiRTX430	SbiRTX430	SbiRTX430	PF00177	PTHR11205	PTHR1120	KOG3291	K02992	GO:000373
PAC:43576	SbiRTX430	SbiRTX430	SbiRTX430	PF00304					GO:000695
PAC:43575	SbiRTX430	SbiRTX430	SbiRTX430	PF06404	PTHR33285	PTHR33285:SF1			GO:000557
PAC:43576	SbiRTX430	SbiRTX430	SbiRTX430	PF01559					GO:004573
PAC:43575	SbiRTX430	SbiRTX430	SbiRTX430	PF01559					GO:004573
PAC:43575	SbiRTX430	SbiRTX430	SbiRTX430	PF01559					GO:004573
PAC:43576	SbiRTX430	SbiRTX430	SbiRTX430	PF00717	PTHR1238	EC:3.4.21.	KOG0171	K09647	GO:000650
PAC:43576	SbiRTX430	SbiRTX430	SbiRTX430	PF00717	PTHR1238	EC:3.4.21.	KOG0171	K09647	GO:000650
PAC:43573	SbiRTX430	SbiRTX430	SbiRTX430	PF00076	PTHR24012	PTHR2401	KOG0148	K13201	GO:000016
PAC:43574	SbiRTX430	SbiRTX430	SbiRTX430.05G200800.1.p						
PAC:43574	SbiRTX430	SbiRTX430	SbiRTX430	PF00083 P	PTHR23500	PTHR2350	KOG0254		GO:001602
PAC:43574	SbiRTX430	SbiRTX430	SbiRTX430	PF00083 P	PTHR23500	PTHR2350	KOG0254		GO:001602
PAC:43574	SbiRTX430	SbiRTX430	SbiRTX430	PF00083 P	PTHR23500	PTHR2350	KOG0254		GO:001602
PAC:43576	SbiRTX430	SbiRTX430	SbiRTX430.05G20150		PTHR11926	PTHR11926:SF341			GO:000815
PAC:43575	SbiRTX430	SbiRTX430	SbiRTX430	PF01559					GO:004573
PAC:43575	SbiRTX430	SbiRTX430	SbiRTX430	PF01559					GO:004573
PAC:43574	SbiRTX430	SbiRTX430	SbiRTX430	PF01559					GO:004573
PAC:43574	SbiRTX430	SbiRTX430	SbiRTX430	PF01559					GO:000815
PAC:43575	SbiRTX430	SbiRTX430	SbiRTX430	PF01559					GO:004573
PAC:43576	SbiRTX430	SbiRTX430	SbiRTX430	PF01559					GO:004573
PAC:43574	SbiRTX430	SbiRTX430	SbiRTX430.05G20480		PTHR33403	PTHR33403:SF1		K18635	
PAC:43575	SbiRTX430	SbiRTX430	SbiRTX430.05G22710		PTHR33479				GO:000486
PAC:43576	SbiRTX430	SbiRTX430	SbiRTX430	PF04578 P	PTHR31325	PTHR31325:SF23			
PAC:43576	SbiRTX430	SbiRTX430	SbiRTX430	PF01439	PTHR33543	PTHR33543:SF2			GO:001714
PAC:43573	SbiRTX430	SbiRTX430	SbiRTX430.05G24700		PTHR2166	EC:3.1.2.2	KOG3328	K17362	GO:000367
PAC:43551	SbiRTX430	SbiRTX430	SbiRTX430	PF00092 P	PTHR10579	PTHR10579:SF64			
PAC:43550	SbiRTX430	SbiRTX430	SbiRTX430.06G05880		PTHR33184	PTHR33184:SF4			
PAC:43552	SbiRTX430	SbiRTX430	SbiRTX430	PF04689	PTHR35298				GO:000367

PAC:43552	SbiRTX430	SbiRTX430	SbiRTX430	PF04689	PTHR35298				GO:000367
PAC:43550	SbiRTX430	SbiRTX430	SbiRTX430.06G07810		PTHR3631	EC:1.97.1.12		K14332	
PAC:43552	SbiRTX430	SbiRTX430	SbiRTX430	PF14368	PTHR33122	PTHR33122:SF6			
PAC:43552	SbiRTX430	SbiRTX430	SbiRTX430	PF14368	PTHR33122	PTHR33122:SF6			
PAC:43550	SbiRTX430	SbiRTX430	SbiRTX430	PF04398	PTHR31676	PTHR31676:SF18			
PAC:43551	SbiRTX430	SbiRTX430	SbiRTX430	PF00076	PTHR24012	PTHR2401	KOG0148		GO:000016
PAC:43550	SbiRTX430	SbiRTX430	SbiRTX430.06G109600.1.p						
PAC:43552	SbiRTX430	SbiRTX430	SbiRTX430.06G112200.1.p						
PAC:43549	SbiRTX430	SbiRTX430	SbiRTX430.06G14040		PTHR36743				
PAC:43549	SbiRTX430	SbiRTX430	SbiRTX430	PF05680	PTHR36028				GO:000027
PAC:43548	SbiRTX430	SbiRTX430	SbiRTX430	PF00462	PTHR1016	EC:1.8.1.9	KOG1752	K03676	GO:000905
PAC:43550	SbiRTX430	SbiRTX430	SbiRTX430	PF11779	PTHR33727	PTHR33727:SF2			
PAC:43550	SbiRTX430	SbiRTX430	SbiRTX430	PF11779	PTHR33727	PTHR33727:SF2			
PAC:43551	SbiRTX430	SbiRTX430	SbiRTX430	PF01423	PTHR12777		KOG3459	K11096	GO:000838
PAC:43551	SbiRTX430	SbiRTX430	SbiRTX430	PF01423	PTHR12777		KOG3459	K11096	GO:000838
PAC:43551	SbiRTX430	SbiRTX430	SbiRTX430	PF01423	PTHR12777		KOG3459	K11096	GO:000838
PAC:43550	SbiRTX430	SbiRTX430	SbiRTX430	PF04520	PTHR33083	PTHR33083:SF17			
PAC:43551	SbiRTX430	SbiRTX430	SbiRTX430.06G165400.1.p						
PAC:43549	SbiRTX430	SbiRTX430	SbiRTX430	PF00097	PTHR12313	PTHR1231	KOG0823	K10666	GO:000551
PAC:43549	SbiRTX430	SbiRTX430	SbiRTX430	PF00097	PTHR12313	PTHR1231	KOG0823	K10666	GO:000551
PAC:43549	SbiRTX430	SbiRTX430	SbiRTX430	PF00097	PTHR12313	PTHR1231	KOG0823		GO:000551
PAC:43551	SbiRTX430	SbiRTX430	SbiRTX430	PF02892 P	PTHR23272	PTHR2327	KOG1121		GO:000367
PAC:43551	SbiRTX430	SbiRTX430	SbiRTX430	PF15511	PTHR10484		KOG3467	K11254	GO:000078
PAC:43550	SbiRTX430	SbiRTX430	SbiRTX430	PF04570	PTHR33059	PTHR33059:SF7			
PAC:43550	SbiRTX430	SbiRTX430	SbiRTX430.06G22260		PTHR36320				GO:001676
PAC:43551	SbiRTX430	SbiRTX430	SbiRTX430	PF12609	PTHR33090	PTHR33090:SF2			
PAC:43552	SbiRTX430	SbiRTX430	SbiRTX430	PF12609	PTHR33090				
PAC:43552	SbiRTX430	SbiRTX430	SbiRTX430	PF12609	PTHR33090				
PAC:43549	SbiRTX430	SbiRTX430	SbiRTX430	PF12609	PTHR33090				
PAC:43550	SbiRTX430	SbiRTX430	SbiRTX430	PF01423	PTHR10553		KOG1781	K12626	GO:000039
PAC:43551	SbiRTX430	SbiRTX430	SbiRTX430.06G257700.1.p						
PAC:43550	SbiRTX430	SbiRTX430	SbiRTX430.06G27270		PTHR21588		KOG4618		
PAC:43549	SbiRTX430	SbiRTX430	SbiRTX430.06G27950		PTHR33156	PTHR33156:SF8			
PAC:43549	SbiRTX430	SbiRTX430	SbiRTX430.06G27950		PTHR33156	PTHR33156:SF8			
PAC:43549	SbiRTX430	SbiRTX430	SbiRTX430.06G27950		PTHR33156	PTHR33156:SF8			
PAC:43550	SbiRTX430	SbiRTX430	SbiRTX430.06G27990		PTHR33156	PTHR33156:SF8			
PAC:43549	SbiRTX430	SbiRTX430	SbiRTX430	PF00366	PTHR10744	PTHR1074	KOG1740	K02961	GO:000373
PAC:43564	SbiRTX430	SbiRTX430	SbiRTX430	PF00111	PTHR19370	PTHR19370:SF110		K02639	GO:000905
PAC:43562	SbiRTX430	SbiRTX430	SbiRTX430	PF00428	PTHR21141		KOG1762	K02942	GO:000373
PAC:43562	SbiRTX430	SbiRTX430	SbiRTX430	PF00428	PTHR21141		KOG1762	K02942	GO:000373
PAC:43562	SbiRTX430	SbiRTX430	SbiRTX430	PF00428	PTHR21141		KOG1762	K02942	GO:000373
PAC:43562	SbiRTX430	SbiRTX430	SbiRTX430.07G029600.1.p						
PAC:43562	SbiRTX430	SbiRTX430	SbiRTX430	PF00234	PTHR33076	PTHR33076:SF12			GO:000686
PAC:43562	SbiRTX430	SbiRTX430	SbiRTX430.07G03140		PTHR33076	PTHR33076:SF12			GO:000686

PAC:43561	SbiRTX430	SbiRTX430	SbiRTX430	PF00335	PTHR19282	PTHR19282:SF262			GO:001602
PAC:43563	SbiRTX430	SbiRTX430	SbiRTX430	PF00264 P	PTHR1147	EC:1.10.3.1		K00422	GO:000409
PAC:43561	SbiRTX430	SbiRTX430	SbiRTX430	PF03604	PTHR12056	PTHR1205	KOG3507	K03009	GO:000367
PAC:43563	SbiRTX430	SbiRTX430	SbiRTX430	PF04725	PTHR34369			K03541	GO:000952
PAC:43562	SbiRTX430	SbiRTX430	SbiRTX430	PF02671					GO:000635
PAC:43562	SbiRTX430	SbiRTX430	SbiRTX430	PF02671	PTHR12346				GO:000635
PAC:43562	SbiRTX430	SbiRTX430	SbiRTX430	PF02671	PTHR12346				GO:000635
PAC:43564	SbiRTX430	SbiRTX430	SbiRTX430.07G10200		PTHR18901	PTHR18901:SF28			
PAC:43564	SbiRTX430	SbiRTX430	SbiRTX430.07G10200		PTHR18901	PTHR18901:SF28			
PAC:43564	SbiRTX430	SbiRTX430	SbiRTX430.07G10200		PTHR18901	PTHR18901:SF28			
PAC:43561	SbiRTX430	SbiRTX430	SbiRTX430.07G10730		PTHR35990	PTHR35990:SF1			
PAC:43564	SbiRTX430	SbiRTX430	SbiRTX430.07G12160		PTHR35106	PTHR35106:SF2			
PAC:43563	SbiRTX430	SbiRTX430	SbiRTX430	PF01217	PTHR11753		KOG0934	K12403	GO:000681
PAC:43562	SbiRTX430	SbiRTX430	SbiRTX430	PF02297	PTHR11387			K18179	GO:000412
PAC:43562	SbiRTX430	SbiRTX430	SbiRTX430	PF01781	PTHR10965	PTHR1096	KOG3499	K02923	GO:000373
PAC:43563	SbiRTX430	SbiRTX430	SbiRTX430.07G20530		PTHR31568				
PAC:43563	SbiRTX430	SbiRTX430	SbiRTX430.07G205300.2.p						
PAC:43562	SbiRTX430	SbiRTX430	SbiRTX430	PF02953			KOG3489	K17780	
PAC:43554	SbiRTX430	SbiRTX430	SbiRTX430	PF01187	PTHR1195	EC:5.3.2.1	KOG1759		
PAC:43554	SbiRTX430	SbiRTX430	SbiRTX430	PF01187	PTHR1195	EC:5.3.2.1	KOG1759	K07253	
PAC:43553	SbiRTX430	SbiRTX430	SbiRTX430.08G09500		PTHR38398				
PAC:43552	SbiRTX430	SbiRTX430	SbiRTX430.08G12110		PTHR33167	PTHR33167:SF6			
PAC:43554	SbiRTX430	SbiRTX430	SbiRTX430	PF03171 P	PTHR1020	EC:1.14.20	KOG0143		GO:001649
PAC:43553	SbiRTX430	SbiRTX430	SbiRTX430.08G137200.1.p						
PAC:43554	SbiRTX430	SbiRTX430	SbiRTX430.08G148300.1.p						
PAC:43552	SbiRTX430	SbiRTX430	SbiRTX430.08G161000.1.p						
PAC:43555	SbiRTX430	SbiRTX430	SbiRTX430	PF03638	PTHR12446	PTHR12446:SF25			
PAC:43554	SbiRTX430	SbiRTX430	SbiRTX430	PF07847	PTHR2296	EC:1.13.11	KOG4281	K10712	GO:001670
PAC:43570	SbiRTX430	SbiRTX430	SbiRTX430	PF00280	PTHR33091	PTHR33091:SF8			GO:000486
PAC:43569	SbiRTX430	SbiRTX430	SbiRTX430	PF00280	PTHR33091	PTHR33091:SF10			GO:000486
PAC:43572	SbiRTX430	SbiRTX430	SbiRTX430	PF00280	PTHR33091	PTHR33091:SF10			GO:000486
PAC:43572	SbiRTX430	SbiRTX430	SbiRTX430	PF00280	PTHR33091	PTHR33091:SF10			GO:000486
PAC:43573	SbiRTX430	SbiRTX430	SbiRTX430	PF00280	PTHR33091	PTHR33091:SF10			GO:000486
PAC:43571	SbiRTX430	SbiRTX430	SbiRTX430.09G013900.1.p						
PAC:43573	SbiRTX430	SbiRTX430	SbiRTX430.09G01570		PTHR34565				
PAC:43570	SbiRTX430	SbiRTX430	SbiRTX430	PF00125 P	PTHR23430		KOG1756	K11251	GO:000078
PAC:43571	SbiRTX430	SbiRTX430	SbiRTX430	PF15054	PTHR33528	PTHR33528:SF5			
PAC:43571	SbiRTX430	SbiRTX430	SbiRTX430	PF15054	PTHR33528	PTHR33528:SF5			
PAC:43570	SbiRTX430	SbiRTX430	SbiRTX430	PF01679	PTHR21659	PTHR2165	KOG1773		GO:001602
PAC:43571	SbiRTX430	SbiRTX430	SbiRTX430	PF01599 P	PTHR10666		KOG0004	K02977	GO:000373
PAC:43570	SbiRTX430	SbiRTX430	SbiRTX430.09G05650		PTHR14154	PTHR14154:SF18			
PAC:43571	SbiRTX430	SbiRTX430	SbiRTX430	PF05047	PTHR12878		KOG3446	K03946	
PAC:43570	SbiRTX430	SbiRTX430	SbiRTX430.09G06460		PTHR34198	PTHR34198:SF3			
PAC:43571	SbiRTX430	SbiRTX430	SbiRTX430.09G07880		PTHR33834				

PAC:43570	SbiRTX430	SbiRTX430	SbiRTX430	PF06376	PTHR33374	PTHR33374:SF6			
PAC:43570	SbiRTX430	SbiRTX430	SbiRTX430	PF03145	PTHR1031	EC:6.3.2.1	KOG3002	K08742	GO:000484
PAC:43573	SbiRTX430	SbiRTX430	SbiRTX430	PF00189	PTHR11760	PTHR11760:SF19		K02982	GO:000372
PAC:43573	SbiRTX430	SbiRTX430	SbiRTX430	PF00189	PTHR11760	PTHR11760:SF19			GO:000372
PAC:43572	SbiRTX430	SbiRTX430	SbiRTX430	PF00410	PTHR11758		KOG1754	K02994	GO:000373
PAC:43572	SbiRTX430	SbiRTX430	SbiRTX430	PF00411	PTHR11759		KOG0407	K02948	GO:000373
PAC:43571	SbiRTX430	SbiRTX430	SbiRTX430	PF01000 P	PTHR3210	EC:2.7.7.6		K03040	GO:000367
PAC:43570	SbiRTX430	SbiRTX430	SbiRTX430	PF01084	PTHR13479			K02963	GO:000373
PAC:43571	SbiRTX430	SbiRTX430	SbiRTX430	PF01779	PTHR12884		KOG3504	K02905	GO:000373
PAC:43571	SbiRTX430	SbiRTX430	SbiRTX430	PF01779	PTHR12884		KOG3504	K02905	GO:000373
PAC:43570	SbiRTX430	SbiRTX430	SbiRTX430.09G169400.1.p						
PAC:43570	SbiRTX430	SbiRTX430	SbiRTX430	PF01158	PTHR10114		KOG3452	K02920	GO:000373
PAC:43570	SbiRTX430	SbiRTX430	SbiRTX430	PF01158	PTHR10114		KOG3452	K02920	GO:000373
PAC:43573	SbiRTX430	SbiRTX430	SbiRTX430	PF13499	PTHR23050	PTHR2305	KOG0027	K02183	GO:000550
PAC:43573	SbiRTX430	SbiRTX430	SbiRTX430	PF13499 P	PTHR23050	PTHR2305	KOG0027	K02183	GO:000550
PAC:43572	SbiRTX430	SbiRTX430	SbiRTX430.09G20010		PTHR34114	PTHR34114:SF1			GO:000425
PAC:43572	SbiRTX430	SbiRTX430	SbiRTX430	PF13414	PTHR36326	PTHR36326:SF1			GO:000551
PAC:43569	SbiRTX430	SbiRTX430	SbiRTX430.09G20780		PTHR33869	PTHR33869:SF2			
PAC:43573	SbiRTX430	SbiRTX430	SbiRTX430.09G21010		PTHR33782	PTHR33782:SF3			
PAC:43573	SbiRTX430	SbiRTX430	SbiRTX430.09G21010		PTHR33782	PTHR33782:SF3			
PAC:43573	SbiRTX430	SbiRTX430	SbiRTX430.09G21480		PTHR36744				
PAC:43572	SbiRTX430	SbiRTX430	SbiRTX430.09G22470		PTHR15223				GO:000574
PAC:43570	SbiRTX430	SbiRTX430	SbiRTX430	PF00428	PTHR21141	PTHR2114	KOG3449	K02943	
PAC:43547	SbiRTX430	SbiRTX430	SbiRTX430.10G01520		PTHR33876	PTHR33876:SF1			
PAC:43547	SbiRTX430	SbiRTX430	SbiRTX430	PF00125	PTHR11426		KOG1745	K11253	GO:000078
PAC:43546	SbiRTX430	SbiRTX430	SbiRTX430	PF00534 P	PTHR1252	EC:2.4.1.242		K13679	GO:000437
PAC:43544	SbiRTX430	SbiRTX430	SbiRTX430	PF02892 P	PTHR23272	PTHR2327	KOG1121		GO:000367
PAC:43544	SbiRTX430	SbiRTX430	SbiRTX430	PF05699 P	PTHR23272	PTHR2327	KOG1121		GO:000367
PAC:43544	SbiRTX430	SbiRTX430	SbiRTX430	PF05699 P	PTHR23272	PTHR2327	KOG1121		GO:000367
PAC:43546	SbiRTX430	SbiRTX430	SbiRTX430	PF12734	PTHR35470	PTHR35470:SF2			
PAC:43546	SbiRTX430	SbiRTX430	SbiRTX430	PF12734	PTHR35470	PTHR35470:SF2			
PAC:43546	SbiRTX430	SbiRTX430	SbiRTX430.10G03680		PTHR33474	PTHR33474:SF5			
PAC:43547	SbiRTX430	SbiRTX430	SbiRTX430.10G03790		PTHR33120				
PAC:43548	SbiRTX430	SbiRTX430	SbiRTX430.10G04110		PTHR36755				
PAC:43547	SbiRTX430	SbiRTX430	SbiRTX430.10G08360		PTHR34268	PTHR34268:SF3			
PAC:43547	SbiRTX430	SbiRTX430	SbiRTX430	PF06522	PTHR33417				
PAC:43547	SbiRTX430	SbiRTX430	SbiRTX430	PF06522	PTHR33417				
PAC:43545	SbiRTX430	SbiRTX430	SbiRTX430	PF05486	PTHR12834	PTHR1283	KOG3465	K03109	GO:000661
PAC:43546	SbiRTX430	SbiRTX430	SbiRTX430.10G139900.1.p						
PAC:43546	SbiRTX430	SbiRTX430	SbiRTX430.10G142700.1.p						
PAC:43545	SbiRTX430	SbiRTX430	SbiRTX430.10G144900.1.p						
PAC:43545	SbiRTX430	SbiRTX430	SbiRTX430	PF00411	PTHR11759	PTHR1175	KOG0407	K02955	GO:000373
PAC:43546	SbiRTX430	SbiRTX430	SbiRTX430	PF05207	PTHR21454		KOG2923	K15455	
PAC:43546	SbiRTX430	SbiRTX430	SbiRTX430	PF00170	PTHR22952	PTHR22952:SF127			GO:000370

PAC:4354[illegible]	SbiRTX430	SbiRTX430	SbiRTX430	PF00170	PTHR22952	PTHR22952:SF127			GO:00037[illegible]
PAC:43547	SbiRTX430	SbiRTX430	SbiRTX430	PF12609	PTHR33090	PTHR33090:SF3			
PAC:4354[illegible]	SbiRTX430	SbiRTX430	SbiRTX430	PF06293	PTHR2405[illegible]	EC:2.7.11.24		K04371	GO:00046[illegible]
PAC:4354[illegible]	SbiRTX430	SbiRTX430	SbiRTX430	PF06293	PTHR2405[illegible]	EC:2.7.11.24		K04371	GO:00046[illegible]
PAC:4354[illegible]	SbiRTX430	SbiRTX430	SbiRTX430	PF06293	PTHR2405[illegible]	EC:2.7.11.24		K04371	GO:00046[illegible]
PAC:4354[illegible]	SbiRTX430	SbiRTX430	SbiRTX430	PF01187	PTHR1195[illegible]	EC:5.3.2.1	KOG1759		

Best-hit-ara	Best-hit-ara	Best-hit-cla	Best-hit-cla	Best-hit-ric	Best-hit-rice-defline				
				LOC_Os03	expressed protein				
AT3G0661(	DNA-bindir	Cre01.g04:	(1 of 1) KO(	LOC_Os03	huntingtin-	putative	expressed		
AT5G2018(	Ribosomal	Cre09.g38(	Mitochondi	LOC_Os03	ribosomal		expressed			
AT3G09860				LOC_Os03	expressed protein				
AT4G1637(	oligopeptide transporter			LOC_Os03	oligopeptid	putative	expressed		
AT2G0490(	(1 of 1) KO(	Cre12.g54:	(1 of 1) KOG3472 - Predicted small membrane protein						
AT3G5456(	histone H2	Cre13.g56:	Histone H2	LOC_Os03	Core histor	putative	expressed		
				LOC_Os03	PIII5 - Prote	expressed			
AT3G2636(	Ribosomal protein S21 family prot			LOC_Os03	ribosomal		putative	expressed		
AT5G2770(	Ribosomal	Cre03.g20:	Cytosolic 8	LOC_Os03	40S ribosor	putative	expressed		
AT1G4941(	translocase of the outer mitochon			LOC_Os03	expressed protein				
AT1G4941(	translocase of the outer mitochon			LOC_Os03	expressed protein				
AT3G1900(	2-oxoglutarate (2OG) and Fe(II)-de			LOC_Os03	gibberellin	putative	expressed		
AT5G5356(	cytochrom	Cre16.g65!	Cytochrom	LOC_Os10	cytochrom	expressed			
AT5G5356(	cytochrom	Cre16.g65!	Cytochrom	LOC_Os10	cytochrom	expressed			
AT2G28430									
AT2G28430									
AT1G5766(	Translation	Cre06.g27(	Cytosolic 8	LOC_Os10	60S ribosor	putative	expressed		
AT5G4743(	DWNN don	a CCHC-ty	Cre12.g54:	conserved	LOC_Os10	transposor	putative	CACTA	En/Spm su
AT5G4743(	DWNN don	a CCHC-ty	Cre12.g54:	conserved	LOC_Os10	transposor	putative	CACTA	En/Spm su
AT5G4743(	DWNN don	a CCHC-ty	Cre12.g54:	conserved	LOC_Os10	transposor	putative	CACTA	En/Spm su
				LOC_Os10	dirigent	putative	expressed		
				LOC_Os09	expressed protein				
76 GO:0008270				LOC_Os10	ZOS10-02 ·	expressed			
AT2G3218(	plastid transcriptionally active 18			LOC_Os02	enzyme of	putative	expressed		
AT1G32310									
AT1G32310									
AT1G7641(	RING/U-box superfamily protein			LOC_Os10	RING-H2 fi	putative	expressed		
AT1G0766(	Histone su	Cre06.g27(	Histone H4	LOC_Os10	Core histor	putative	expressed		
57									
AT1G2635!	SPIRAL1-like1			LOC_Os03	nitrilase-as	putative	expressed		
AT1G2635!	SPIRAL1-like1			LOC_Os03	nitrilase-as	putative	expressed		
AT5G15802				LOC_Os03	expressed protein				
AT3G1608(	Zinc-bindir	Cre10.g43(	Cytosolic 8	LOC_Os02	ribosomal		putative	expressed		
AT5G2770(	Ribosomal	Cre03.g20:	Cytosolic 8	LOC_Os03	40S ribosor	putative	expressed		
AT5G2770(	Ribosomal	Cre03.g20:	Cytosolic 8	LOC_Os03	40S ribosor	putative	expressed		

AT3G5649(	HIS triad family protein 3								
20 GO:0051260									
20 GO:0051260									
AT4G1614(	cAMP-regu	Cre06.g30	(1 of 1) PTH	LOC_Os12	Lg106	putative	expressed		
52 GO:0009607				LOC_Os03	pathogene	putative	expressed		
AT5G6414(	ribosomal	Cre12.g51(	Cytosolic 8	LOC_Os10	40S riboso	putative	expressed		
AT5G6414(	ribosomal	Cre12.g51(	Cytosolic 8	LOC_Os10	40S riboso	putative	expressed		
AT2G3751(	RNA-binding (RRM/RBD/RNP moti			LOC_Os03	RNA recogr	putative	expressed		
AT3G0908	Protein of u	Cre03.g18	(1 of 2) PFC	LOC_Os03	membrane	putative	expressed		
AT5G6122(	LYR family	Cre08.g38	Complex 1	LOC_Os08	LYR motif c	putative	expressed		
AT2G1670(	actin depolymerizing factor 5			LOC_Os03	actin-depo	putative	expressed		
AT2G1670(	actin depolymerizing factor 5			LOC_Os03	actin-depo	putative	expressed		
AT3G2590(	Homocyste	Cre10.g45	(1 of 1) 2.1	LOC_Os03	homocyste	putative	expressed		
AT2G1854(	RmlC-like cupins superfamily prot			LOC_Os03	cupin doma	expressed			
AT1G7800(	sulfate transporter 1;2			LOC_Os03	sulfate trar	putative	expressed		
AT1G7800(	sulfate transporter 1;2			LOC_Os03	sulfate trar	putative	expressed		
AT1G7800(	sulfate transporter 1;2			LOC_Os03	sulfate trar	putative	expressed		
AT1G7800(	sulfate transporter 1;2			LOC_Os03	sulfate trar	putative	expressed		
AT1G7800(	sulfate transporter 1;2			LOC_Os03	sulfate trar	putative	expressed		
AT1G7800(	sulfate transporter 1;2			LOC_Os03	sulfate trar	putative	expressed		
AT5G64816		Cre07.g31	Conserved	LOC_Os03	THION26 -	expressed			
AT5G64816		Cre07.g31	Conserved	LOC_Os03	THION26 -	expressed			
AT5G6414(	ribosomal	Cre12.g51(	Cytosolic 8	LOC_Os10	40S riboso	putative	expressed		
15				LOC_Os03	tetratricop	expressed			
52									
AT1G6685(	Bifunctional inhibitor/lipid-transfe			LOC_Os10	LTPL160 -	expressed			
AT4G1249(	Bifunctional inhibitor/lipid-transfe			LOC_Os03	LTPL116 -	expressed			
AT4G1249(	Bifunctional inhibitor/lipid-transfe			LOC_Os03	LTPL116 -	expressed			
AT4G1249(	Bifunctional inhibitor/lipid-transfe			LOC_Os03	LTPL116 -	expressed			
AT5G0212(	one helix p	Cre02.g10	peptidyl-pr	FKBP-type	LOC_Os05	expressed protein			
AT5G0212(	one helix p	Cre02.g10	peptidyl-pr	FKBP-type	LOC_Os05	expressed protein			
AT3G0139(	vacuolar m	Cre11.g46	Vacuolar A	LOC_Os04	vacuolar A	putative	expressed		
AT3G0139(	vacuolar m	Cre11.g46	Vacuolar A	LOC_Os04	vacuolar A	putative	expressed		
AT3G0139(	vacuolar m	Cre11.g46	Vacuolar A	LOC_Os04	vacuolar A	putative	expressed		
AT3G0772(	Galactose	Cre03.g14(	(1 of 1) PTH	LOC_Os09	kelch repea	putative	expressed		
48 GO:0030598									
48 GO:0030598									
AT2G0213(	low-molecular-weight cysteine-ric			LOC_Os03	DEF8 - Def	expressed			
AT5G4101(	DNA direct	7 kDa subu	Cre07.g34	(1 of 1) K03	II	and III sub	POLR2K)	LOC_Os01	DNA direct
				LOC_Os07	expressed protein				

AT1G0707(	Ribosomal	Cre10.g45!	Cytosolic 8	LOC_Os02	60S riboso	putative	expressed	
AT1G0707(	Ribosomal	Cre10.g45!	Cytosolic 8	LOC_Os02	60S riboso	putative	expressed	
15								
				LOC_Os09	expressed protein			
				LOC_Os09	expressed protein			
				LOC_Os09	expressed protein			
				LOC_Os09	hypoxia-re:	putative	expressed	
33								
AT1G10522				LOC_Os09	expressed protein			
AT5G1694(	carbon-sulfur lyases			LOC_Os09	proline-rich	putative	expressed	
AT3G1018!	Gibberellin-regulated family prote			LOC_Os09	GASR10 - G	expressed		
AT1G5391(	related to AP2 12			LOC_Os09	AP2 domai	expressed		
AT1G5391(	related to AP2 12			LOC_Os09	AP2 domai	expressed		
AT1G5391(	related to AP2 12			LOC_Os09	AP2 domai	expressed		
AT2G20480								
AT1G5567(	photosyste	Cre12.g56(	Photosyste	LOC_Os09	photosyste	chloroplas	putative	expressed
AT3G6188(	cytochrome p450 78a9			LOC_Os09	cytochrom	putative	expressed	
AT4G04745				LOC_Os07	expressed protein			
43 GO:0006413		Cre06.g29{	Translation initiation protein					
43 GO:0006413		Cre06.g29{	Translation initiation protein					
43 GO:0006413		Cre06.g29{	Translation initiation protein					
43 GO:0006413		Cre06.g29{	Translation initiation protein					
43 GO:0006413		Cre06.g29{	Translation initiation protein					
35 GO:0005622 GO:00(		Cre07.g32!	Ribosomal	LOC_Os11	60S riboso	putative	expressed	
35 GO:0005622 GO:00(		Cre07.g32!	Ribosomal	LOC_Os11	60S riboso	putative	expressed	
AT4G27380				LOC_Os07	expressed protein			
52								
AT1G6503:	(1 of 3) PF13232 - Complex1_LYR-			LOC_Os02	expressed protein			
09				LOC_Os03	EF hand fa	putative		
AT5G0250:	Oligosaccaryltransferase							
AT5G0250:	Oligosaccaryltransferase							
01 GO:0046872				LOC_Os07	heavy meta	expressed		
AT1G1677(	(1 of 1) PTH	Cre07.g32!	(1 of 1) PTH	LOC_Os02	expressed protein			
AT5G59613								
AT1G7317:	bonsai							
AT1G7317:	bonsai							
				LOC_Os12	expressed protein			

AT5G1925(	Glycoprotein membrane precurso			LOC_Os07	uncharacte	putative	expressed	
AT1G16916				LOC_Os01	expressed protein			
AT2G0652(	photosystem II subunit X			LOC_Os03	ultraviolet-	putative	expressed	
AT1G0766(	Histone su	Cre06.g27(	Histone H4	LOC_Os10	Core histor	putative	expressed	
57 GO:0005576				LOC_Os01	BBTI12 - Bc	expressed		
AT4G1593(	Dynein light chain type 1 family pr(			LOC_Os01	dynein ligh	expressed		
AT4G1593(	Dynein light chain type 1 family pr(			LOC_Os01	dynein ligh	expressed		
				LOC_Os01	split hand/	putative	expressed	
				LOC_Os01	split hand/	putative	expressed	
AT3G0589(	Low tempe	Cre03.g19(	(1 of 1) KOG1773 - Stress responsive protein					
AT3G0589(	Low tempe	Cre03.g19(	(1 of 1) KOG1773 - Stress responsive protein					
15								
ATCG0044	NADH:ubiq	chain 3 protein			LOC_Os10	NADPH-de	putative	expressed
AT2G4507(	Preprotein	Sec61-bet	Cre07.g31(	SEC61-bet	LOC_Os01	protein trar	putative	expressed
AT3G1388:	Ribosomal	Cre14.g60!	Putative 8C	LOC_Os01	50S riboso	putative	expressed	
AT5G53650				LOC_Os01	expressed protein			
AT5G4168!	Mitochond	subunit Tom7			LOC_Os05	mitochond	putative	expressed
AT1G7535(	Ribosomal	Cre08.g36!	Chloroplas	LOC_Os01	50S riboso	putative	expressed	
AT1G7535(	Ribosomal	Cre08.g36!	Chloroplas	LOC_Os01	50S riboso	putative	expressed	
				LOC_Os01	ubiquitin-c	putative	expressed	
				LOC_Os01	expressed protein			
				LOC_Os01	expressed protein			
AT1G70350				LOC_Os01	expressed protein			
AT2G3149(	(1 of 54) 1.6.5.3 - NADH:ubiquinone reductase (H(+)-translocating) / Ubiquinone reductase							
AT5G3921(	chlororespiratory reduction 7							
AT2G3057(	photosystem II reaction center W			LOC_Os01	photosyste	chloroplas	putative	expressed
AT2G3057(	photosystem II reaction center W			LOC_Os01	photosyste	chloroplas	putative	expressed
AT2G4088(	cystatin A	Cre04.g22!	(1 of 1) PTH	LOC_Os01	cysteine pr	putative	expressed	
AT2G4088(	cystatin A	Cre04.g22!	(1 of 1) PTH	LOC_Os01	cysteine pr	putative	expressed	
69 GO:0008289				LOC_Os01	LTPL16 - Pr	expressed		
AT3G5620(	Transmeml	Cre02.g14!	Amino acid	LOC_Os01	transmeml	putative	expressed	
AT3G5620(	Transmeml	Cre02.g14!	Amino acid	LOC_Os01	transmeml	putative	expressed	
AT3G5620(	Transmeml	Cre02.g14!	Amino acid	LOC_Os01	transmeml	putative	expressed	
AT2G4033(	PYR1-like 6							
AT3G4598(	Histone su	Cre13.g57(	Histone H2	LOC_Os01	Core histor	putative	expressed	
57 GO:0005576								
AT1G7686(	Small nucl(	Cre16.g66:	U6 snRNA-	LOC_Os01	LSM domai	expressed		

AT2G3814(	plastid-specific ribosomal protein			LOC_Os05	30S riboso	chloroplas	putative	expressed	
AT2G3814(	plastid-specific ribosomal protein			LOC_Os05	30S riboso	chloroplas	putative	expressed	
AT4G2684(	small ubiq	Cre16.g67!	Small ubiq	LOC_Os01	ubiquitin fa	expressed			
AT5G4375(	NAD(P)H dehydrogenase 18			LOC_Os01	expressed protein				
90 GO:0006508				LOC_Os01	xylanase in	putative	expressed		
AT3G1153(	Vacuolar p	Cre12.g49:	Vacuolar tr	LOC_Os01	vacuolar pr	putative	expressed		
AT3G1153(	Vacuolar p	Cre12.g49:	Vacuolar tr	LOC_Os01	vacuolar pr	putative	expressed		
66 GO:0003676		Cre02.g07!	(1 of 90) PF	RBD	or RNP dor	LOC_Os01	RNA recogn	putative	expressed
AT5G4696(	Plant invertase/pectin methyleste			LOC_Os02	invertase/p	putative	expressed		
AT1G6266(	Glycosyl hy	Cre12.g50:	(1 of 2) K01	sacA)	LOC_Os02	glycosyl hy	putative	expressed	
AT1G6266(	Glycosyl hy	Cre12.g50:	(1 of 2) K01	sacA)	LOC_Os02	glycosyl hy	putative	expressed	
				LOC_Os02	THION20 -	putative	expressed		
AT3G4957(	response to low sulfur 3			LOC_Os02	UP-9A	putative	expressed		
AT3G1095(	Zinc-bindir	Cre06.g25:	Cytosolic 8	LOC_Os05	60S riboso	putative	expressed		
AT5G5046(	secE/sec6:	Cre16.g68(	SEC61-gan	LOC_Os02	protein trar	putative	expressed		
24 GO:0015991 GO:0016820 GO:0033178									
AT5G5498(	Uncharacterised protein family (U			LOC_Os02	integral me	putative	expressed		
AT3G0492(	Ribosomal	Cre10.g45(	Cytosolic 8	LOC_Os02	40S riboso	putative	expressed		
AT2G4256(	late embryogenesis abundant dom			LOC_Os02	late embry(	putative	expressed		
AT2G4256(	late embryogenesis abundant dom			LOC_Os02	late embry(	putative	expressed		
				LOC_Os02	expressed protein				
AT1G79390				LOC_Os02	expressed protein				
AT5G4097(	Protein of unknown function (DUF			LOC_Os02	expressed protein				
AT5G4108(	PLC-like ph	Cre03.g20:	Glycerophc	probably a	LOC_Os02	glycerophо	putative	expressed	
AT5G4108(	PLC-like ph	Cre03.g20:	Glycerophc	probably a	LOC_Os02	glycerophо	putative	expressed	
AT5G4108(	PLC-like ph	Cre03.g20:	Glycerophc	probably a	LOC_Os02	glycerophо	putative	expressed	
AT2G2758(	A20/AN1-like zinc finger family pro			LOC_Os02	zinc finger ,	putative	expressed		
AT4G1010(	co-factor fo	reductase	Cre08.g38:	(1 of 1) PTH	LOC_Os02	VP15	putative	expressed	
				LOC_Os01	expressed protein				
				LOC_Os01	expressed protein				
				LOC_Os02	hypoxia-res	putative	expressed		
				LOC_Os02	hypoxia-res	putative	expressed		
AT1G08580				LOC_Os02	expressed protein				
AT1G08580				LOC_Os02	expressed protein				
AT5G1719(	(1 of 2) PTHR12701:SF12 - GB			LOC_Os02	erwinia ind	putative	expressed		
AT5G1719(	(1 of 2) PTHR12701:SF12 - GB			LOC_Os02	erwinia ind	putative	expressed		
AT5G0818(	Ribosomal	Cre16.g69(	Nucleolar p	Small subunit of H/ACA snoRNPs					

AT1G1203(	Protein of ι	Cre03.g14ξ	PWR motif	LOC_Os02	plant-spec	expressed		
AT1G7737(	Glutaredox	Cre12.g55(	Glutaredox	CPYC type	LOC_Os02	OsGrx_C3 -	expressed	
AT2G2426(	LJRHL1-like 1			LOC_Os02	BHLH trans	putative	expressed	
AT2G2426(	LJRHL1-like 1			LOC_Os02	BHLH trans	putative	expressed	
AT4G3198!	Ribosomal protein L39 family prot			LOC_Os02	60S riboso	putative	expressed	
				LOC_Os02	expressed protein			
AT1G6725(	Proteasom	Cre16.g66!	Proteasom	LOC_Os02	proteasom	expressed		
29	GO:0016717 GO:00!	Cre01.g03ξ	Chloroplas	LOC_Os12	fatty acid d	putative	expressed	
AT1G7466(	mini zinc finger 1			LOC_Os11	ZF-HD prot	expressed		
AT3G4518(	Ubiquitin-li	Cre17.g72!	Ubiquitin-r	LOC_Os11	ubiquitin-li	putative	expressed	
AT3G4518(	Ubiquitin-li	Cre17.g72!	Ubiquitin-r	LOC_Os11	ubiquitin-li	putative	expressed	
37	GO:0010333 GO:0016829							
ATCG0090	Ribosomal protein S7p/S5e family			LOC_Os10	chloroplast	putative	expressed	
52								
76	GO:0008083 GO:0008283			LOC_Os11	phytosulfo	putative	expressed	
35								
35								
35								
AT1G5353(	Peptidase !	Cre11.g46	Mitochond	LOC_Os11	OsSigP7 - F	expressed		
AT1G5353(	Peptidase !	Cre11.g46	Mitochond	LOC_Os11	OsSigP7 - F	expressed		
AT1G1737(	oligouridylate binding protein 1B			LOC_Os11	RNA recogr	putative	expressed	
20	GO:0016021 GO:0022857 GO:0022891 GO			LOC_Os11	transporter	putative	expressed	
20	GO:0016021 GO:0022857 GO:0022891 GO			LOC_Os11	transporter	putative	expressed	
20	GO:0016021 GO:0022857 GO:0022891 GO			LOC_Os11	transporter	putative	expressed	
52	GO:0016758							
35								
35								
35								
52	GO:0016627 GO:0045735 GO:0050660 GO:0055114							
35								
35								
				LOC_Os11	nitrilase-as	putative	expressed	
57	GO:0005576							
				LOC_Os01	expressed protein			
48	GO:0030598 GO:0046872			LOC_Os11	metallothic	putative	expressed	
AT2G2959(	Thioesterase superfamily protein			LOC_Os02	thioesteras	putative	expressed	
				LOC_Os03	zinc finger	putative	expressed	
AT4G3210!	Beta-1	3-N-Acetylglucosaminyltransferas			LOC_Os02	LGC1	putative	expressed
AT3G5337(	S1FA-like DNA-binding protein			LOC_Os04	DNA-bindir	putative	expressed	

AT3G5337(	S1FA-like DNA-binding protein			LOC_Os04	DNA-bindir	putative	expressed	
AT1G0838(	photosyste	Cre07.g33	Photosyste	LOC_Os04	membrane	putative	expressed	
				LOC_Os04	LTPL102 - F	expressed		
				LOC_Os04	LTPL102 - F	expressed		
				LOC_Os04	DUF538 dc	putative	expressed	
AT1G4749(	RNA-bindir	Cre16.g65	(1 of 16) KC	subunit 4	LOC_Os04	RNA recogr	putative	expressed
AT3G29034								
AT2G43540				LOC_Os04	expressed protein			
AT5G15320				LOC_Os04	expressed protein			
AT5G6303(	Thioredoxin superfamily protein			LOC_Os04	OsGrx_C2.	expressed		
				LOC_Os04	expressed protein			
				LOC_Os04	expressed protein			
AT3G6284(	Small nucl	Cre10.g45	Small nucl	LOC_Os05	LSM domai	expressed		
AT3G6284(	Small nucl	Cre10.g45	Small nucl	LOC_Os05	LSM domai	expressed		
AT3G6284(	Small nucl	Cre10.g45	Small nucl	LOC_Os05	LSM domai	expressed		
AT5G6068(	Protein of u	DUF584			LOC_Os04	DUF584 dc	putative	expressed
				LOC_Os04	expressed protein			
AT4G0351(	RING mem	Cre12.g51	(1 of 2) PTH	LOC_Os04	zinc finger	C3HC4 typ	expressed	
AT4G0351(	RING mem	Cre12.g51	(1 of 2) PTH	LOC_Os04	zinc finger	C3HC4 typ	expressed	
AT4G0351(	RING mem	Cre12.g51	(1 of 2) PTH	LOC_Os04	zinc finger	C3HC4 typ	expressed	
76 GO:0003677 GO:0046983								
AT1G0766(	Histone su	Cre06.g27	Histone H4	LOC_Os10	Core histor	putative	expressed	
				LOC_Os04	DUF581 dc	expressed		
AT1G30880				LOC_Os04	expressed protein			
				LOC_Os04	expressed protein			
AT4G1027(	Wound-responsive family protein			LOC_Os04	wound indu	putative	expressed	
AT4G1027(	Wound-responsive family protein			LOC_Os04	wound indu	putative	expressed	
AT4G1027(	Wound-responsive family protein			LOC_Os04	wound indu	putative	expressed	
AT2G0387(	Small nucl	Cre03.g16	U6 snRNA-	LOC_Os08	LSM domai	expressed		
AT1G0216(	Cox19 family protein (CHCH motif)							
AT4G39300				LOC_Os01	expressed protein			
AT4G39300				LOC_Os01	expressed protein			
AT4G39300				LOC_Os01	expressed protein			
AT4G39300				LOC_Os01	expressed protein			
AT1G7985(	ribosomal	Cre02.g11	Chloroplast ribosomal	protein S17				
AT1G6095(	2Fe-2S ferr	Cre14.g62	Chloroplas	LOC_Os08	2Fe-2S iror	expressed		
AT1G0110(	60S acidic	Cre17.g73	Acidic ribo	LOC_Os08	60S acidic	putative	expressed	
AT1G0110(	60S acidic	Cre17.g73	Acidic ribo	LOC_Os08	60S acidic	putative	expressed	
AT1G0110(	60S acidic	Cre17.g73	Acidic ribo	LOC_Os08	60S acidic	putative	expressed	
69 GO:0008289				LOC_Os08	LTPL24 - Pr	expressed		
69 GO:0008289				LOC_Os08	LTPL24 - Pr	expressed		

21				LOC_Os08	tetraspanir	putative	expressed		
97 GO:0008152 GO:0016491 GO:0055114									
AT5G4101(	DNA direct	7 kDa subu	Cre07.g34:	(1 of 1) K03	II	and III sub	POLR2K)	LOC_Os01	DNA direct
AT1G7904(	photosyste	Cre06.g26:	Photosyste	LOC_Os08	photosyste	chloroplas	putative	expressed	
55									
55									
AT1G2725(	Paired amphipathic helix (PAH2) superfamily protein								
AT1G16000				LOC_Os08	expressed protein				
AT5G3806(	(1 of 1) PTH	Cre13.g605700							
AT2G1979(	SNARE-like	Cre07.g33(	Sigma4-Ad	LOC_Os08	clathrin ad	expressed			
AT5G5800	Cytochrom	subunit Vib family protein			LOC_Os08	expressed protein			
AT3G5954(	Ribosomal	Cre07.g32	Ribosomal protein L38						
AT5G5081(	translocase inner membrane subu			LOC_Os08	mitochond	putative	expressed		
AT5G0165(	Tautomera	Cre17.g70(	(1 of 1) 5.3	LOC_Os11	macrophag	putative	expressed		
AT5G0165(	Tautomera	Cre17.g70(	(1 of 1) 5.3	LOC_Os11	macrophag	putative	expressed		
AT5G50335				LOC_Os12	expressed protein				
				LOC_Os12	expressed protein				
91 GO:0055114				LOC_Os03	1-aminocy	putative	expressed		
				LOC_Os12	RALFL42 - l	expressed			
				LOC_Os12	expressed protein				
				LOC_Os12	tesmin/TS(	expressed			
02 GO:0055114				LOC_Os12	2-aminoeth	putative	expressed		
57 GO:0009611				LOC_Os12	inhibitor I fa	putative	expressed		
57 GO:0009611				LOC_Os12	inhibitor I fa	putative	expressed		
57 GO:0009611									
57 GO:0009611									
57 GO:0009611									
AT5G59613									
AT5G0256(	histone H2A 12								
AT3G0589(	Low tempe	Cre03.g19(	(1 of 1) KO(	LOC_Os05	OsRCI2-6 -	expressed			
AT2G4711(	ubiquitin 6	Cre06.g27:	Cytosolic 8	LOC_Os05	40S riboso	putative	expressed		
AT5G4789(	NADH-ubic	putative	Cre16.g67(	NADH:ubiq	LOC_Os04	ribosomal		putative	expressed
AT1G52720				LOC_Os05	expressed protein				
AT2G31090				LOC_Os05	expressed protein				

AT2G4633(	arabinogalactan protein 16							
AT3G6179(	Protein with RING/U-box and TRAF			LOC_Os05	seven in ab	expressed		
ATCG0080	structural constituent of ribosome			LOC_Os09	chloroplast	putative	expressed	
ATCG0080	structural constituent of ribosome			LOC_Os09	chloroplast	putative	expressed	
ATCG0077	ribosomal protein S8			LOC_Os08	chloroplast	putative	expressed	
ATCG0075	ribosomal protein S11			LOC_Os10	chloroplast	putative	expressed	
ATCG0074	RNA polymerase subunit alpha			LOC_Os08	DNA-direct	putative	expressed	
ATCG0065	ribosomal protein S18			LOC_Os08	chloroplast	putative	expressed	
AT3G0670(	Ribosomal	Cre03.g20	Cytosolic 8	LOC_Os01	60S riboso	putative	expressed	
AT3G0670(	Ribosomal	Cre03.g20	Cytosolic 8	LOC_Os01	60S riboso	putative	expressed	
AT3G5374(	Ribosomal	Cre12.g48	Cytosolic 8	LOC_Os01	60S riboso	putative	expressed	
AT3G5374(	Ribosomal	Cre12.g48	Cytosolic 8	LOC_Os01	60S riboso	putative	expressed	
AT3G4381(	calmodulir	Cre03.g17	Calmodulir	radial spok	LOC_Os01	OsCam1-3	expressed	
AT3G4381(	calmodulir	Cre03.g17	Calmodulir	radial spok	LOC_Os01	OsCam1-3	expressed	
52 GO:0006508								
AT5G4885(	Tetratricopeptide repeat (TPR)-like			LOC_Os05	tetratricop	expressed		
				LOC_Os05	expressed protein			
				LOC_Os05	expressed protein			
				LOC_Os05	expressed protein			
AT2G43780								
AT1G7620(	(1 of 1) PTHR15223 - NADH-UBIQU			LOC_Os05	expressed protein			
AT3G4459(	60S acidic	Cre02.g14	Acidic ribos	LOC_Os01	60S acidic	putative	expressed	
		Cre14.g62	nickel trans	LOC_Os06	high-affinit	putative	expressed	
AT4G4004(	Histone su	Cre06.g26	Histone H3	LOC_Os03	histone H3	putative	expressed	
73		Cre17.g72	Granule-bc	LOC_Os06	starch synt	putative	expressed	
76 GO:0003677 GO:0046983								
76 GO:0003677 GO:0046983								
76 GO:0003677 GO:0046983								
				LOC_Os06	expressed protein			
				LOC_Os06	expressed protein			
				LOC_Os06	expressed protein			
AT3G13845				LOC_Os06	expressed protein			
AT2G31945				LOC_Os06	expressed protein			
AT3G4814(	B12D protein			LOC_Os06	B12D prote	putative	expressed	
AT3G4814(	B12D protein			LOC_Os06	B12D prote	putative	expressed	
AT3G4910(	Signal reco	SRP9/SRP	Cre12.g54	Subunit of	LOC_Os06	signal reco	putative	expressed
AT3G5258(	Ribosomal	Cre11.g48	Cytosolic 8	LOC_Os02	ribosomal	putative	expressed	
		Cre16.g65	(1 of 1) K15	DPH3)	LOC_Os06	diphthamic	putative	expressed
AT5G2480(	basic leucine zipper 9			LOC_Os06	bZIP transc	expressed		

AT5G2480(	basic leucine zipper 9			LOC_Os06	bZIP transc	expressed			
				LOC_Os06	wound indu	putative	expressed		
AT1G5958(	mitogen-activated protein kinase I			LOC_Os06	CGMC_MA	MAPK	GSK3	and CLKC	expressed
AT1G5958(	mitogen-activated protein kinase I			LOC_Os06	CGMC_MA	MAPK	GSK3	and CLKC	expressed
AT1G5958(	mitogen-activated protein kinase I			LOC_Os06	CGMC_MA	MAPK	GSK3	and CLKC	expressed
AT5G5717(	Tautomera	Cre17.g70(	(1 of 1) 5.3	LOC_Os06	macrophag	putative	expressed		

expressed
expressed
expressed

7 kDa subı expressed

7 kDa subı expressed
