# Supplementary material for: Large scale deletion and rebalancing within the k1C kafirin family in sorghum
Source: Front Plant Sci. 2025 Oct 23;16:1686027. doi: 10.3389/fpls.2025.1686027 (PMC12588965; doi:10.3389/fpls.2025.1686027)
Supplement: Supplementary file 2 [file DataSheet2.pdf]

#pacID	locusName	transcriptN	peptideNa	Pfam	Panther	ec	KOG	KO	GO
PAC:43576	SbiRTX430	SbiRTX430	SbiRTX430.01G00080		PTHR33740 PTHR33740:SF3				
PAC:43576	SbiRTX430	SbiRTX430	SbiRTX430.01G00080		PTHR33740 PTHR33740:SF3				
PAC:43580	SbiRTX430	SbiRTX430	SbiRTX430.01G01130		PTHR33880 PTHR33880:SF3				
PAC:43580	SbiRTX430	SbiRTX430	SbiRTX430.01G01130		PTHR33880 PTHR33880:SF3				
PAC:43584	SbiRTX430	SbiRTX430	SbiRTX430	PF00201	PTHR1192	EC:2.4.1.85		K13030	GO:000815
PAC:43584	SbiRTX430	SbiRTX430	SbiRTX430	PF03407	PTHR24015 PTHR24015:SF903				
PAC:43578	SbiRTX430	SbiRTX430	SbiRTX430	PF13561	PTHR2432	EC:1.1.1.1	KOG1205		
PAC:43578	SbiRTX430	SbiRTX430	SbiRTX430	PF03168	PTHR31415 PTHR31415:SF6				
PAC:43581	SbiRTX430	SbiRTX430	SbiRTX430	PF00320	PTHR10071 PTHR10071:SF208				GO:00037
PAC:43581	SbiRTX430	SbiRTX430	SbiRTX430	PF00320	PTHR10071 PTHR10071:SF208				GO:00037
PAC:43578	SbiRTX430	SbiRTX430	SbiRTX430	PF12697	PTHR1099	EC:3.3.2.1	KOG4178		GO:000382
PAC:43578	SbiRTX430	SbiRTX430	SbiRTX430	PF12697	PTHR1099	EC:3.3.2.10			GO:000382
PAC:43578	SbiRTX430	SbiRTX430	SbiRTX430	PF12697	PTHR1099	EC:3.3.2.10			GO:000382
PAC:43581	SbiRTX430	SbiRTX430	SbiRTX430	PF02536	PTHR13068 PTHR13068:SF17			K15032	GO:000369
PAC:43578	SbiRTX430	SbiRTX430	SbiRTX430	PF01535 P	PTHR24015 PTHR24015:SF659				GO:00055
PAC:43578	SbiRTX430	SbiRTX430	SbiRTX430	PF01357 P	PTHR31867 PTHR31867:SF5				GO:00055
PAC:43578	SbiRTX430	SbiRTX430	SbiRTX430	PF02431	PTHR2803	EC:5.5.1.6		K01859	GO:00098
PAC:43584	SbiRTX430	SbiRTX430	SbiRTX430	PF13839 P	PTHR32285 PTHR32285:SF67				
PAC:43578	SbiRTX430	SbiRTX430	SbiRTX430	PF05691	PTHR3126	EC:2.4.1.82		K06617	GO:00038
PAC:43578	SbiRTX430	SbiRTX430	SbiRTX430	PF05691	PTHR3126	EC:2.4.1.82			GO:00038
PAC:43577	SbiRTX430	SbiRTX430	SbiRTX430	PF04862	PTHR31265 PTHR31265:SF2				
PAC:43582	SbiRTX430	SbiRTX430	SbiRTX430	PF03080 P	PTHR31589 PTHR31589:SF16				
PAC:43577	SbiRTX430	SbiRTX430	SbiRTX430.01G05010		PTHR34574 PTHR34574:SF2				GO:00055
PAC:43579	SbiRTX430	SbiRTX430	SbiRTX430.01G052500.1.p						
PAC:43578	SbiRTX430	SbiRTX430	SbiRTX430.01G058100.1.p						
PAC:43584	SbiRTX430	SbiRTX430	SbiRTX430	PF00112 P	PTHR1241	EC:3.4.22.	KOG1543		GO:00065
PAC:43578	SbiRTX430	SbiRTX430	SbiRTX430	PF00190	PTHR31189 PTHR31189:SF3				GO:00457
PAC:43578	SbiRTX430	SbiRTX430	SbiRTX430	PF00190	PTHR31189 PTHR31189:SF3				GO:00457
PAC:43580	SbiRTX430	SbiRTX430	SbiRTX430	PF00179	PTHR2406	EC:6.3.2.1	KOG0419	K10573	
PAC:43578	SbiRTX430	SbiRTX430	SbiRTX430	PF00226 P	PTHR24078 PTHR2407		KOG0712	K09503	GO:00064
PAC:43578	SbiRTX430	SbiRTX430	SbiRTX430	PF00684 P	PTHR24078 PTHR2407		KOG0712	K09503	GO:00064
PAC:43578	SbiRTX430	SbiRTX430	SbiRTX430	PF00684 P	PTHR24078 PTHR2407		KOG0712		GO:00064
PAC:43577	SbiRTX430	SbiRTX430	SbiRTX430	PF13410 P	PTHR1126	EC:2.5.1.1	KOG0406	K00799	GO:00055
PAC:43579	SbiRTX430	SbiRTX430	SbiRTX430	PF13410 P	PTHR1126	EC:2.5.1.1	KOG0406	K00799	GO:000551
PAC:43582	SbiRTX430	SbiRTX430	SbiRTX430	PF07491	PTHR20835 PTHR20835:SF0			K17553	
PAC:43580	SbiRTX430	SbiRTX430	SbiRTX430	PF02507	PTHR3493	EC:1.97.1.12		K02694	GO:00095
PAC:43580	SbiRTX430	SbiRTX430	SbiRTX430	PF02507	PTHR3493	EC:1.97.1.12		K02694	GO:00095
PAC:43580	SbiRTX430	SbiRTX430	SbiRTX430	PF02507	PTHR3493	EC:1.97.1.12		K02694	GO:00095
PAC:43584	SbiRTX430	SbiRTX430	SbiRTX430.01G07210		PTHR12763 PTHR127		KOG0723	K09539	
PAC:43578	SbiRTX430	SbiRTX430	SbiRTX430.01G07780		PTHR33526 PTHR33526:SF1				
PAC:43579	SbiRTX430	SbiRTX430	SbiRTX430.01G08230		PTHR33167 PTHR33167:SF8				
PAC:43579	SbiRTX430	SbiRTX430	SbiRTX430	PF03106	PTHR31221 PTHR31221:SF15				GO:00037
PAC:43579	SbiRTX430	SbiRTX430	SbiRTX430	PF03106	PTHR31221 PTHR31221:SF15				GO:00037

PAC:43579	SbiRTX430	SbiRTX430	SbiRTX430	PF03106	PTHR31221	PTHR31221:SF15			GO:000370
PAC:43582	SbiRTX430	SbiRTX430	SbiRTX430	PF00582	PTHR31964	PTHR31964:SF38			GO:000695
PAC:43580	SbiRTX430	SbiRTX430	SbiRTX430	PF04927	PTHR31174	PTHR31174:SF4			
PAC:43577	SbiRTX430	SbiRTX430	SbiRTX430	PF04927	PTHR31174	PTHR31174:SF2			
PAC:43584	SbiRTX430	SbiRTX430	SbiRTX430	PF01027	PTHR23291	PTHR2329	KOG2322	K06890	
PAC:43583	SbiRTX430	SbiRTX430	SbiRTX430	PF00201	PTHR1192	EC:2.4.1.128			GO:000815
PAC:43583	SbiRTX430	SbiRTX430	SbiRTX430	PF03000	PTHR32370	PTHR32370:SF35			GO:000551
PAC:43583	SbiRTX430	SbiRTX430	SbiRTX430	PF00294	PTHR1058	EC:2.7.1.6	KOG2855		GO:000552
PAC:43583	SbiRTX430	SbiRTX430	SbiRTX430	PF00294	PTHR1058	EC:2.7.1.6	KOG2855		GO:000552
PAC:43578	SbiRTX430	SbiRTX430	SbiRTX430.01G10080		PTHR35127	PTHR35127:SF1			
PAC:43581	SbiRTX430	SbiRTX430	SbiRTX430.01G10290		PTHR33983	PTHR33983:SF5			
PAC:43581	SbiRTX430	SbiRTX430	SbiRTX430.01G10290		PTHR33983	PTHR33983:SF5			
PAC:43577	SbiRTX430	SbiRTX430	SbiRTX430.01G10300		PTHR33983	PTHR33983:SF5			
PAC:43577	SbiRTX430	SbiRTX430	SbiRTX430	PF02450	PTHR1144	EC:2.3.1.4	KOG2369	K06129	GO:000662
PAC:43580	SbiRTX430	SbiRTX430	SbiRTX430.01G10740		PTHR31412	PTHR31412:SF2			
PAC:43579	SbiRTX430	SbiRTX430	SbiRTX430.01G11220		PTHR36410	PTHR36410:SF1			
PAC:43577	SbiRTX430	SbiRTX430	SbiRTX430	PF00360 P	PTHR24423	PTHR24423:SF519		K12120	GO:000015
PAC:43582	SbiRTX430	SbiRTX430	SbiRTX430	PF00360 P	PTHR24423	PTHR24423:SF519		K12120	GO:000015
PAC:43582	SbiRTX430	SbiRTX430	SbiRTX430	PF00360 P	PTHR24423	PTHR24423:SF519			GO:000551
PAC:43581	SbiRTX430	SbiRTX430	SbiRTX430.01G11750		PTHR36012	PTHR36012:SF1			
PAC:43584	SbiRTX430	SbiRTX430	SbiRTX430	PF00012	PTHR19375	PTHR19375:SF219		K09490	
PAC:43584	SbiRTX430	SbiRTX430	SbiRTX430	PF11250	PTHR33155	PTHR33155:SF1			
PAC:43577	SbiRTX430	SbiRTX430	SbiRTX430	PF02840 P	PTHR13007	PTHR1300	KOG2808	K12817	GO:000568
PAC:43577	SbiRTX430	SbiRTX430	SbiRTX430	PF02840 P	PTHR13007	PTHR1300	KOG2808	K12817	GO:000568
PAC:43577	SbiRTX430	SbiRTX430	SbiRTX430	PF02840 P	PTHR13007	PTHR13007:SF20			GO:000568
PAC:43581	SbiRTX430	SbiRTX430	SbiRTX430	PF00305 P	PTHR1177	EC:1.13.11.58		K15718	GO:000551
PAC:43577	SbiRTX430	SbiRTX430	SbiRTX430	PF00305 P	PTHR1177	EC:1.13.11.58		K15718	GO:000551
PAC:43577	SbiRTX430	SbiRTX430	SbiRTX430	PF00305	PTHR1177	EC:1.13.11.58			GO:001649
PAC:43577	SbiRTX430	SbiRTX430	SbiRTX430	PF00305	PTHR1177	EC:1.13.11.58			GO:001649
PAC:43577	SbiRTX430	SbiRTX430	SbiRTX430	PF00305	PTHR1177	EC:1.13.11.58			GO:001649
PAC:43577	SbiRTX430	SbiRTX430	SbiRTX430	PF00305	PTHR1177	EC:1.13.11.58			GO:001649
PAC:43577	SbiRTX430	SbiRTX430	SbiRTX430	PF00305	PTHR1177	EC:1.13.11.58			GO:001649
PAC:43577	SbiRTX430	SbiRTX430	SbiRTX430	PF00305	PTHR1177	EC:1.13.11.58			GO:001649
PAC:43578	SbiRTX430	SbiRTX430	SbiRTX430	PF01277	PTHR33203	PTHR33203:SF6			GO:001251
PAC:43583	SbiRTX430	SbiRTX430	SbiRTX430	PF00117	PTHR11922	PTHR1192	KOG3179		
PAC:43582	SbiRTX430	SbiRTX430	SbiRTX430.01G14200		PTHR34789				
PAC:43583	SbiRTX430	SbiRTX430	SbiRTX430	PF13920	PTHR10044	PTHR1004	KOG1100	K19042	GO:000551
PAC:43577	SbiRTX430	SbiRTX430	SbiRTX430	PF12681	PTHR1037	EC:5.1.99.	KOG2944		
PAC:43577	SbiRTX430	SbiRTX430	SbiRTX430	PF12681	PTHR1037	EC:5.1.99.	KOG2944		
PAC:43577	SbiRTX430	SbiRTX430	SbiRTX430	PF12681	PTHR1037	EC:5.1.99.	KOG2944		
PAC:43582	SbiRTX430	SbiRTX430	SbiRTX430	PF07847	PTHR2296	EC:1.13.11	KOG4281	K10712	GO:001670
PAC:43578	SbiRTX430	SbiRTX430	SbiRTX430	PF07847	PTHR2296	EC:1.13.11	KOG4281	K10712	GO:001670
PAC:43580	SbiRTX430	SbiRTX430	SbiRTX430	PF07802	PTHR34357	PTHR34357:SF1			
PAC:43577	SbiRTX430	SbiRTX430	SbiRTX430	PF03171 P	PTHR1020	EC:1.14.11	KOG0143		GO:000550

PAC:43579	SbiRTX430	SbiRTX430	SbiRTX430	PF03171 P	PTHR10209	PTHR1020	KOG0143		GO:00055(
PAC:43581	SbiRTX430	SbiRTX430	SbiRTX430	PF03478	PTHR36901	PTHR36901:SF1			GO:000551
PAC:43584	SbiRTX430	SbiRTX430	SbiRTX430	PF02466	PTHR15371	PTHR1537	KOG3324	K17794	
PAC:43581	SbiRTX430	SbiRTX430	SbiRTX430	PF01053	PTHR1180	EC:4.4.1.1	KOG0053	K01761	GO:00036
PAC:43579	SbiRTX430	SbiRTX430	SbiRTX430	PF00230	PTHR19139	PTHR1913	KOG0223	K09874	GO:00052
PAC:43581	SbiRTX430	SbiRTX430	SbiRTX430.01G20690(		PTHR33836	PTHR33836:SF1			GO:00043
PAC:43581	SbiRTX430	SbiRTX430	SbiRTX430.01G20690(		PTHR33836	PTHR33836:SF1			GO:00043
PAC:43584	SbiRTX430	SbiRTX430	SbiRTX430	PF05147	PTHR12736	PTHR12736:SF13			GO:00038
PAC:43584	SbiRTX430	SbiRTX430	SbiRTX430	PF05147	PTHR12736	PTHR12736:SF13			GO:00038
PAC:43578	SbiRTX430	SbiRTX430	SbiRTX430	PF16499	PTHR1145	EC:3.2.1.2	KOG2366	K07407	GO:000382
PAC:43580	SbiRTX430	SbiRTX430	SbiRTX430	PF00230	PTHR19139	PTHR1913	KOG0223	K09873	GO:00052
PAC:43582	SbiRTX430	SbiRTX430	SbiRTX430.01G22360(		PTHR35718	PTHR35718:SF1			
PAC:43584	SbiRTX430	SbiRTX430	SbiRTX430.01G225200.1.p						
PAC:43582	SbiRTX430	SbiRTX430	SbiRTX430	PF00249	PTHR10641	PTHR10641:SF533		K09422	GO:00036
PAC:43579	SbiRTX430	SbiRTX430	SbiRTX430	PF05903	PTHR12378	PTHR1237	KOG0324		
PAC:43579	SbiRTX430	SbiRTX430	SbiRTX430	PF05903	PTHR12378	PTHR1237	KOG0324		
PAC:43579	SbiRTX430	SbiRTX430	SbiRTX430	PF05903	PTHR12378	PTHR1237	KOG0324		
PAC:43579	SbiRTX430	SbiRTX430	SbiRTX430	PF05903	PTHR12378	PTHR1237	KOG0324		
PAC:43576	SbiRTX430	SbiRTX430	SbiRTX430	PF03016	PTHR11062	PTHR1106	KOG1021		
PAC:43582	SbiRTX430	SbiRTX430	SbiRTX430.01G23920(		PTHR33548	PTHR33548:SF4			
PAC:43580	SbiRTX430	SbiRTX430	SbiRTX430	PF02040	PTHR10283	PTHR1028	KOG2639		GO:00151(
PAC:43580	SbiRTX430	SbiRTX430	SbiRTX430	PF02040	PTHR10283	PTHR1028	KOG2639		GO:00151(
PAC:43580	SbiRTX430	SbiRTX430	SbiRTX430	PF03600	PTHR10283	PTHR1028	KOG2639		GO:00160
PAC:43580	SbiRTX430	SbiRTX430	SbiRTX430	PF02040	PTHR10283	PTHR1028	KOG2639		GO:00151(
PAC:43580	SbiRTX430	SbiRTX430	SbiRTX430	PF03600	PTHR10283	PTHR1028	KOG2639		GO:00160
PAC:43580	SbiRTX430	SbiRTX430	SbiRTX430	PF02040	PTHR10283	PTHR1028	KOG2639		GO:00151(
PAC:43584	SbiRTX430	SbiRTX430	SbiRTX430	PF07690	PTHR2406	EC:3.6.3.27		K08176	GO:00053
PAC:43584	SbiRTX430	SbiRTX430	SbiRTX430	PF13921	PTHR21717	PTHR21717:SF29			GO:00036
PAC:43583	SbiRTX430	SbiRTX430	SbiRTX430.01G242000.1.p						
PAC:43580	SbiRTX430	SbiRTX430	SbiRTX430	PF00582	PTHR31964	PTHR31964:SF15			GO:00069
PAC:43580	SbiRTX430	SbiRTX430	SbiRTX430	PF02485	PTHR19297	PTHR1929	KOG0799		GO:00083
PAC:43578	SbiRTX430	SbiRTX430	SbiRTX430.01G24790(		PTHR35986	PTHR35986:SF1			
PAC:43579	SbiRTX430	SbiRTX430	SbiRTX430	PF13417	PTHR1126	EC:2.5.1.1	KOG0406	K00799	GO:000551
PAC:43584	SbiRTX430	SbiRTX430	SbiRTX430	PF01408	PTHR2260	EC:1.1.1.1	KOG2741		GO:00164
PAC:43577	SbiRTX430	SbiRTX430	SbiRTX430.01G26480(		PTHR33541	PTHR33541:SF1			
PAC:43578	SbiRTX430	SbiRTX430	SbiRTX430	PF04674	PTHR31279	PTHR31279:SF10			
PAC:43577	SbiRTX430	SbiRTX430	SbiRTX430	PF01190	PTHR33935				
PAC:43580	SbiRTX430	SbiRTX430	SbiRTX430	PF00141	PTHR3138	EC:1.11.1.7		K00430	GO:00046(
PAC:43579	SbiRTX430	SbiRTX430	SbiRTX430	PF02458	PTHR3164	EC:2.3.1.196			GO:00167
PAC:43577	SbiRTX430	SbiRTX430	SbiRTX430	PF03405	PTHR3115	EC:1.14.19.2		K03921	GO:000663
PAC:43577	SbiRTX430	SbiRTX430	SbiRTX430	PF03405	PTHR3115	EC:1.14.19.2		K03921	GO:000663
PAC:43577	SbiRTX430	SbiRTX430	SbiRTX430	PF03405	PTHR3115	EC:1.14.19.2		K03921	GO:000663
PAC:43582	SbiRTX430	SbiRTX430	SbiRTX430	PF03141	PTHR10108	PTHR10108:SF844			GO:00081(
PAC:43582	SbiRTX430	SbiRTX430	SbiRTX430	PF03141	PTHR10108	PTHR10108:SF844			GO:00081(

PAC:43582	SbiRTX430	SbiRTX430	SbiRTX430	PF03141	PTHR10108	PTHR10108:SF844			GO:000816
PAC:43579	SbiRTX430	SbiRTX430	SbiRTX430	PF08477	PTHR11711	PTHR1171	KOG0070	K07977	GO:000552
PAC:43582	SbiRTX430	SbiRTX430	SbiRTX430	PF00153	PTHR24089	PTHR2408	KOG0758	K15109	
PAC:43582	SbiRTX430	SbiRTX430	SbiRTX430	PF00153	PTHR24089	PTHR2408	KOG0758	K15109	
PAC:43582	SbiRTX430	SbiRTX430	SbiRTX430.01G305700.1.p						
PAC:43583	SbiRTX430	SbiRTX430	SbiRTX430	PF00069 P	PTHR2435	EC:2.7.11.1			GO:000467
PAC:43579	SbiRTX430	SbiRTX430	SbiRTX430	PF00125 P	PTHR23430		KOG1756	K11251	GO:000078
PAC:43578	SbiRTX430	SbiRTX430	SbiRTX430	PF01357 P	PTHR31692	PTHR31692:SF17			GO:000557
PAC:43582	SbiRTX430	SbiRTX430	SbiRTX430	PF01357 P	PTHR31692	PTHR31692:SF17			GO:000557
PAC:43578	SbiRTX430	SbiRTX430	SbiRTX430	PF01357 P	PTHR31692	PTHR31692:SF17			GO:000557
PAC:43584	SbiRTX430	SbiRTX430	SbiRTX430.01G31820		PTHR1118	EC:2.4.1.1	KOG1950		
PAC:43584	SbiRTX430	SbiRTX430	SbiRTX430	PF01619	PTHR1391	EC:1.5.5.2	KOG0186	K00318	GO:000465
PAC:43584	SbiRTX430	SbiRTX430	SbiRTX430	PF01619	PTHR1391	EC:1.5.5.2	KOG0186	K00318	GO:000465
PAC:43584	SbiRTX430	SbiRTX430	SbiRTX430	PF01619	PTHR1391	EC:1.5.5.2	KOG0186	K00318	GO:000465
PAC:43578	SbiRTX430	SbiRTX430	SbiRTX430	PF08450	PTHR1042	EC:4.3.3.2	KOG1520		
PAC:43583	SbiRTX430	SbiRTX430	SbiRTX430.01G328500.1.p						
PAC:43584	SbiRTX430	SbiRTX430	SbiRTX430	PF06454	PTHR31142	PTHR31142:SF3			
PAC:43584	SbiRTX430	SbiRTX430	SbiRTX430	PF06454	PTHR31142	PTHR31142:SF3			
PAC:43582	SbiRTX430	SbiRTX430	SbiRTX430	PF00069 P	PTHR2700	EC:2.7.11.	KOG1187		GO:000467
PAC:43581	SbiRTX430	SbiRTX430	SbiRTX430	PF13410 P	PTHR1126	EC:2.5.1.1	KOG0406	K00799	GO:000551
PAC:43580	SbiRTX430	SbiRTX430	SbiRTX430	PF00514	PTHR23315	PTHR23315:SF97			GO:000548
PAC:43580	SbiRTX430	SbiRTX430	SbiRTX430	PF00514	PTHR23315	PTHR23315:SF97			GO:000548
PAC:43580	SbiRTX430	SbiRTX430	SbiRTX430	PF00514	PTHR23315	PTHR23315:SF97			GO:000548
PAC:43583	SbiRTX430	SbiRTX430	SbiRTX430	PF12695	PTHR1099	EC:3.7.1.1	KOG1454		GO:000372
PAC:43583	SbiRTX430	SbiRTX430	SbiRTX430	PF00646	PTHR10706	PTHR10706:SF128			GO:000551
PAC:43583	SbiRTX430	SbiRTX430	SbiRTX430	PF00646	PTHR10706	PTHR10706:SF128			GO:000551
PAC:43582	SbiRTX430	SbiRTX430	SbiRTX430.01G34370		PTHR33595	PTHR33595:SF1			
PAC:43578	SbiRTX430	SbiRTX430	SbiRTX430	PF00560 P	PTHR2700	EC:2.7.10.1	EC:2.7.11.1		GO:000551
PAC:43583	SbiRTX430	SbiRTX430	SbiRTX430	PF00188	PTHR10334	PTHR1033	KOG3017		
PAC:43584	SbiRTX430	SbiRTX430	SbiRTX430	PF02466	PTHR10485	PTHR1048	KOG1652	K17795	GO:000574
PAC:43579	SbiRTX430	SbiRTX430	SbiRTX430	PF03195	PTHR31304	PTHR31304:SF3			
PAC:43584	SbiRTX430	SbiRTX430	SbiRTX430	PF13499	PTHR1089	EC:2.7.11.1			GO:000550
PAC:43583	SbiRTX430	SbiRTX430	SbiRTX430	PF03619	PTHR23423		KOG2641		
PAC:43581	SbiRTX430	SbiRTX430	SbiRTX430	PF00069	PTHR2434	EC:2.7.11.	KOG0583	K14498	GO:000467
PAC:43581	SbiRTX430	SbiRTX430	SbiRTX430	PF07714	PTHR2434	EC:2.7.11.1			GO:000467
PAC:43581	SbiRTX430	SbiRTX430	SbiRTX430	PF13266	PTHR31132	PTHR31132:SF5			
PAC:43577	SbiRTX430	SbiRTX430	SbiRTX430	PF02358	PTHR1078	EC:3.1.3.12		K01087	GO:000382
PAC:43577	SbiRTX430	SbiRTX430	SbiRTX430	PF02358	PTHR1078	EC:3.1.3.12			GO:000382
PAC:43580	SbiRTX430	SbiRTX430	SbiRTX430	PF00400	PTHR22847	PTHR2284	KOG0771		GO:000551
PAC:43580	SbiRTX430	SbiRTX430	SbiRTX430	PF00400	PTHR22847	PTHR2284	KOG0771		GO:000551
PAC:43580	SbiRTX430	SbiRTX430	SbiRTX430	PF00400	PTHR22847	PTHR2284	KOG0771		GO:000551
PAC:43578	SbiRTX430	SbiRTX430	SbiRTX430	PF08392 P	PTHR3156	EC:2.3.1.199		K15397	GO:000382
PAC:43581	SbiRTX430	SbiRTX430	SbiRTX430.01G37260		PTHR35463	PTHR35463:SF1			
PAC:43580	SbiRTX430	SbiRTX430	SbiRTX430	PF00004 P	PTHR23070	PTHR2307	KOG0743	K08900	GO:000552

PAC:43577	SbiRTX430	SbiRTX430	SbiRTX430	PF00067	PTHR24298	PTHR2429	KOG0156		GO:000449
PAC:43582	SbiRTX430	SbiRTX430	SbiRTX430	PF13419	PTHR1272	EC:3.1.3.2	KOG3085		GO:000815
PAC:43582	SbiRTX430	SbiRTX430	SbiRTX430	PF13668	PTHR31694	PTHR31694:SF4			
PAC:43577	SbiRTX430	SbiRTX430	SbiRTX430	PF01073	PTHR1036	EC:4.1.1.35		K08678	GO:000385
PAC:43577	SbiRTX430	SbiRTX430	SbiRTX430	PF01073	PTHR1036	EC:4.1.1.3	KOG1430		GO:000385
PAC:43581	SbiRTX430	SbiRTX430	SbiRTX430	PF04844	PTHR33057	PTHR33057:SF23			GO:000551
PAC:43578	SbiRTX430	SbiRTX430	SbiRTX430	PF00190	PTHR31189	PTHR31189:SF2			GO:004573
PAC:43579	SbiRTX430	SbiRTX430	SbiRTX430.01G40010		PTHR37758	PTHR37758:SF1			
PAC:43577	SbiRTX430	SbiRTX430	SbiRTX430	PF01676 P	PTHR3163	EC:5.4.2.11	EC:5.4.2.1	K15633	GO:000382
PAC:43580	SbiRTX430	SbiRTX430	SbiRTX430	PF13639 P	PTHR2276	EC:6.3.2.19		K11982	GO:000551
PAC:43580	SbiRTX430	SbiRTX430	SbiRTX430	PF13639 P	PTHR2276	EC:6.3.2.19		K11982	GO:000551
PAC:43580	SbiRTX430	SbiRTX430	SbiRTX430	PF00226	PTHR24078				
PAC:43580	SbiRTX430	SbiRTX430	SbiRTX430	PF00226	PTHR24078				
PAC:43583	SbiRTX430	SbiRTX430	SbiRTX430	PF02987	PTHR23241	PTHR2324	KOG4744		
PAC:43580	SbiRTX430	SbiRTX430	SbiRTX430	PF00173	PTHR10281	PTHR1028	KOG1110	K17278	
PAC:43581	SbiRTX430	SbiRTX430	SbiRTX430	PF05678	PTHR33179	PTHR33179:SF2			
PAC:43583	SbiRTX430	SbiRTX430	SbiRTX430	PF01501	PTHR1118	EC:2.4.1.1	KOG1950	K18819	GO:000601
PAC:43584	SbiRTX430	SbiRTX430	SbiRTX430.01G41420		PTHR35096	PTHR35096:SF3			
PAC:43584	SbiRTX430	SbiRTX430	SbiRTX430.01G41420		PTHR35096	PTHR35096:SF3			
PAC:43582	SbiRTX430	SbiRTX430	SbiRTX430	PF00326	PTHR1173	EC:3.4.19.	KOG2100		GO:000650
PAC:43582	SbiRTX430	SbiRTX430	SbiRTX430	PF00326	PTHR1173	EC:3.4.19.	KOG2100		GO:000650
PAC:43579	SbiRTX430	SbiRTX430	SbiRTX430	PF02466	PTHR15371	PTHR15371:SF1			
PAC:43577	SbiRTX430	SbiRTX430	SbiRTX430	PF04667	PTHR34804	PTHR34804:SF1			
PAC:43583	SbiRTX430	SbiRTX430	SbiRTX430	PF00226	PTHR24078				
PAC:43583	SbiRTX430	SbiRTX430	SbiRTX430	PF00226	PTHR24078				
PAC:43583	SbiRTX430	SbiRTX430	SbiRTX430	PF00226	PTHR24078				
PAC:43577	SbiRTX430	SbiRTX430	SbiRTX430.01G424000.1.p						
PAC:43579	SbiRTX430	SbiRTX430	SbiRTX430	PF00733 P	PTHR1177	EC:6.3.5.4		K01953	GO:000406
PAC:43579	SbiRTX430	SbiRTX430	SbiRTX430	PF00733 P	PTHR1177	EC:6.3.5.4			GO:000406
PAC:43579	SbiRTX430	SbiRTX430	SbiRTX430	PF00733 P	PTHR1177	EC:6.3.5.4			GO:000406
PAC:43579	SbiRTX430	SbiRTX430	SbiRTX430	PF00733 P	PTHR1177	EC:6.3.5.4			GO:000406
PAC:43579	SbiRTX430	SbiRTX430	SbiRTX430	PF00733 P	PTHR1177	EC:6.3.5.4			GO:000406
PAC:43577	SbiRTX430	SbiRTX430	SbiRTX430.01G43360		PTHR33701	PTHR33701:SF2			
PAC:43582	SbiRTX430	SbiRTX430	SbiRTX430	PF13410 P	PTHR11260	PTHR1126	KOG0406	K00799	GO:000551
PAC:43583	SbiRTX430	SbiRTX430	SbiRTX430	PF01789	PTHR31407	PTHR31407:SF10			GO:000550
PAC:43578	SbiRTX430	SbiRTX430	SbiRTX430	PF00012	PTHR19375	PTHR19375:SF232		K03283	
PAC:43581	SbiRTX430	SbiRTX430	SbiRTX430.01G44350		PTHR1272	EC:3.1.3.5			
PAC:43581	SbiRTX430	SbiRTX430	SbiRTX430.01G44350		PTHR12725	PTHR12725:SF72			
PAC:43582	SbiRTX430	SbiRTX430	SbiRTX430	PF08609	PTHR19316	PTHR1931	KOG2160	K09562	GO:000548
PAC:43577	SbiRTX430	SbiRTX430	SbiRTX430	PF04788	PTHR31300	PTHR31300:SF2			
PAC:43578	SbiRTX430	SbiRTX430	SbiRTX430	PF00011	PTHR11527	PTHR1152	KOG0710	K13993	
PAC:43582	SbiRTX430	SbiRTX430	SbiRTX430.01G44840		PTHR34686	PTHR34686:SF1			
PAC:43583	SbiRTX430	SbiRTX430	SbiRTX430	PF00076	PTHR15241	PTHR1524	KOG0107	KOG4207	GO:000016
PAC:43583	SbiRTX430	SbiRTX430	SbiRTX430	PF00076	PTHR15241	PTHR1524	KOG0107	KOG4207	GO:000016

PAC:43583	SbiRTX430	SbiRTX430	SbiRTX430	PF00076	PTHR15241	PTHR1524	KOG0107	KOG4207	GO:000016
PAC:43578	SbiRTX430	SbiRTX430	SbiRTX430	PF02298	PTHR33021	PTHR33021:SF39			GO:000905
PAC:43580	SbiRTX430	SbiRTX430	SbiRTX430	PF07250 P	PTHR3220	EC:1.1.3.9			
PAC:43581	SbiRTX430	SbiRTX430	SbiRTX430.01G45860		PTHR34574	PTHR34574:SF2			GO:000550
PAC:43581	SbiRTX430	SbiRTX430	SbiRTX430.01G45860		PTHR34574	PTHR34574:SF2			GO:000550
PAC:43581	SbiRTX430	SbiRTX430	SbiRTX430.01G45860		PTHR34574	PTHR34574:SF2			GO:000550
PAC:43581	SbiRTX430	SbiRTX430	SbiRTX430.01G45860		PTHR34574	PTHR34574:SF2			GO:000550
PAC:43581	SbiRTX430	SbiRTX430	SbiRTX430.01G45860		PTHR34574	PTHR34574:SF2			
PAC:43580	SbiRTX430	SbiRTX430	SbiRTX430	PF00332	PTHR3222	EC:3.2.1.39			GO:000455
PAC:43581	SbiRTX430	SbiRTX430	SbiRTX430	PF00011	PTHR11527	PTHR1152	KOG0710	K13993	
PAC:43579	SbiRTX430	SbiRTX430	SbiRTX430	PF01490	PTHR22950	PTHR2295	KOG1303	K13946	
PAC:43577	SbiRTX430	SbiRTX430	SbiRTX430.01G46630		PTHR37232				
PAC:43583	SbiRTX430	SbiRTX430	SbiRTX430.01G46890		PTHR37217	PTHR37217:SF1			
PAC:43584	SbiRTX430	SbiRTX430	SbiRTX430.01G47000		PTHR10996	PTHR10996:SF136			
PAC:43584	SbiRTX430	SbiRTX430	SbiRTX430.01G47000		PTHR10996	PTHR10996:SF136			
PAC:43584	SbiRTX430	SbiRTX430	SbiRTX430.01G47000		PTHR10996	PTHR10996:SF136			
PAC:43584	SbiRTX430	SbiRTX430	SbiRTX430	PF00644 P	PTHR3226	EC:2.4.2.30			GO:000395
PAC:43584	SbiRTX430	SbiRTX430	SbiRTX430.01G47120		PTHR32263	PTHR32263:SF5			GO:000395
PAC:43581	SbiRTX430	SbiRTX430	SbiRTX430	PF04678	PTHR13462	PTHR13462:SF18			
PAC:43578	SbiRTX430	SbiRTX430	SbiRTX430	PF07690	PTHR23505	PTHR2350	KOG1330		GO:001602
PAC:43578	SbiRTX430	SbiRTX430	SbiRTX430	PF07690	PTHR23505	PTHR2350	KOG1330		GO:001602
PAC:43581	SbiRTX430	SbiRTX430	SbiRTX430	PF00012	PTHR19375	PTHR1937	KOG0101	K09489	
PAC:43577	SbiRTX430	SbiRTX430	SbiRTX430	PF00232	PTHR1035	EC:3.2.1.21		K01188	GO:000455
PAC:43577	SbiRTX430	SbiRTX430	SbiRTX430	PF00232	PTHR1035	EC:3.2.1.2	KOG0626		GO:000455
PAC:43580	SbiRTX430	SbiRTX430	SbiRTX430	PF00046	PTHR24326	PTHR24326:SF262			GO:000367
PAC:43583	SbiRTX430	SbiRTX430	SbiRTX430	PF00190	PTHR31189	PTHR31189:SF7			GO:004573
PAC:43584	SbiRTX430	SbiRTX430	SbiRTX430	PF03188 P	PTHR23130	PTHR2313	KOG4293		GO:001602
PAC:43578	SbiRTX430	SbiRTX430	SbiRTX430	PF13450	PTHR1074	EC:1.5.3.1	KOG0029	K13366	GO:001649
PAC:43578	SbiRTX430	SbiRTX430	SbiRTX430	PF13450	PTHR1074	EC:1.5.3.1	KOG0029	K13366	GO:001649
PAC:43581	SbiRTX430	SbiRTX430	SbiRTX430	PF01658 P	PTHR1151	EC:5.5.1.4		K01858	GO:000451
PAC:43581	SbiRTX430	SbiRTX430	SbiRTX430	PF01658 P	PTHR1151	EC:5.5.1.4			GO:000451
PAC:43581	SbiRTX430	SbiRTX430	SbiRTX430	PF01658 P	PTHR1151	EC:5.5.1.4		K01858	GO:000451
PAC:43581	SbiRTX430	SbiRTX430	SbiRTX430	PF01658 P	PTHR1151	EC:5.5.1.4			GO:000451
PAC:43582	SbiRTX430	SbiRTX430	SbiRTX430	PF12697	PTHR1099	EC:3.7.1.1	KOG1454		
PAC:43582	SbiRTX430	SbiRTX430	SbiRTX430	PF12697	PTHR1099	EC:3.1.1.3	KOG1454		
PAC:43580	SbiRTX430	SbiRTX430	SbiRTX430	PF12697	PTHR1099	EC:3.1.1.3	KOG1454		
PAC:43578	SbiRTX430	SbiRTX430	SbiRTX430	PF00847	PTHR32467	PTHR32467:SF5		K09285	GO:000367
PAC:43583	SbiRTX430	SbiRTX430	SbiRTX430	PF00153	PTHR24089	PTHR2408	KOG0765	K15121	GO:005508
PAC:43583	SbiRTX430	SbiRTX430	SbiRTX430	PF00153	PTHR24089	PTHR2408	KOG0765	K15121	GO:005508
PAC:43582	SbiRTX430	SbiRTX430	SbiRTX430.01G51840		PTHR23241	PTHR23241:SF63			
PAC:43581	SbiRTX430	SbiRTX430	SbiRTX430	PF00515	PTHR36326	PTHR36326:SF1			GO:000551
PAC:43581	SbiRTX430	SbiRTX430	SbiRTX430	PF11947	PTHR34575	PTHR34575:SF2			
PAC:43577	SbiRTX430	SbiRTX430	SbiRTX430	PF04927	PTHR31174	PTHR31174:SF7			
PAC:43577	SbiRTX430	SbiRTX430	SbiRTX430	PF04927	PTHR31174	PTHR31174:SF7			

PAC:43584	SbiRTX430	SbiRTX430	SbiRTX430	PF04842	PTHR31860	PTHR31860:SF5			
PAC:43584	SbiRTX430	SbiRTX430	SbiRTX430	PF04842	PTHR31860	PTHR31860:SF5			
PAC:43584	SbiRTX430	SbiRTX430	SbiRTX430	PF04842	PTHR31860	PTHR31860:SF5			
PAC:43582	SbiRTX430	SbiRTX430	SbiRTX430	PF13386	PTHR33876	PTHR33876:SF2			
PAC:43582	SbiRTX430	SbiRTX430	SbiRTX430	PF13386	PTHR33876	PTHR33876:SF2			
PAC:43582	SbiRTX430	SbiRTX430	SbiRTX430	PF00085	PTHR1043	EC:5.3.4.1	KOG0907		GO:00066
PAC:43582	SbiRTX430	SbiRTX430	SbiRTX430	PF00085	PTHR1043	EC:5.3.4.1	KOG0907		GO:00066
PAC:43583	SbiRTX430	SbiRTX430	SbiRTX430	PF00892	PTHR31218	PTHR31218:SF46			GO:001602
PAC:43578	SbiRTX430	SbiRTX430	SbiRTX430	PF00230	PTHR19139	PTHR1913	KOG0223	K09873	GO:00052
PAC:43577	SbiRTX430	SbiRTX430	SbiRTX430	PF05495 P	PTHR2131	EC:6.3.2.1	KOG1940	K10144	GO:00055
PAC:43584	SbiRTX430	SbiRTX430	SbiRTX430.01G53780		PTHR26312	PTHR26312:SF89			GO:00055
PAC:43583	SbiRTX430	SbiRTX430	SbiRTX430	PF00067	PTHR2429	EC:1.14.15	KOG0157		GO:00055
PAC:43581	SbiRTX430	SbiRTX430	SbiRTX430.01G539100.1.p						
PAC:43580	SbiRTX430	SbiRTX430	SbiRTX430	PF00043 P	PTHR1126	EC:2.5.1.1	KOG0867	K00799	GO:000551
PAC:43582	SbiRTX430	SbiRTX430	SbiRTX430	PF00182	PTHR2259	EC:3.2.1.1	KOG4742		GO:00045
PAC:43579	SbiRTX430	SbiRTX430	SbiRTX430	PF03547	PTHR31651	PTHR3165	KOG2722		GO:001602
PAC:43583	SbiRTX430	SbiRTX430	SbiRTX430	PF00298 P	PTHR11661	PTHR1166	KOG3257	K02867	GO:00037
PAC:43579	SbiRTX430	SbiRTX430	SbiRTX430	PF13813	PTHR3159	EC:2.3.1.75			GO:00471
PAC:43584	SbiRTX430	SbiRTX430	SbiRTX430	PF00141	PTHR3123	EC:1.11.1.7		K00430	GO:00046
PAC:43583	SbiRTX430	SbiRTX430	SbiRTX430.01G564700.1.p						
PAC:43580	SbiRTX430	SbiRTX430	SbiRTX430	PF00230	PTHR19139	PTHR1913	KOG0223	K09873	GO:000521
PAC:43577	SbiRTX430	SbiRTX430	SbiRTX430	PF07765	PTHR32258	PTHR32258:SF3			
PAC:43579	SbiRTX430	SbiRTX430	SbiRTX430	PF01277	PTHR33203	PTHR33203:SF1			GO:00061
PAC:43557	SbiRTX430	SbiRTX430	SbiRTX430	PF00564 P	PTHR2325	EC:2.7.11.	KOG0192		GO:000467
PAC:43557	SbiRTX430	SbiRTX430	SbiRTX430	PF00564 P	PTHR2325	EC:2.7.11.	KOG0192		GO:000467
PAC:43557	SbiRTX430	SbiRTX430	SbiRTX430	PF00564 P	PTHR2325	EC:2.7.11.	KOG0192		GO:000467
PAC:43557	SbiRTX430	SbiRTX430	SbiRTX430	PF00564 P	PTHR2325	EC:2.7.11.	KOG0192		GO:000467
PAC:43559	SbiRTX430	SbiRTX430	SbiRTX430	PF00319 P	PTHR11945	PTHR1194	KOG0014	K09264	GO:00036
PAC:43558	SbiRTX430	SbiRTX430	SbiRTX430	PF07714	PTHR2405	EC:2.7.11.1		K03097	GO:00046
PAC:43558	SbiRTX430	SbiRTX430	SbiRTX430	PF01490	PTHR22950	PTHR2295	KOG1303		
PAC:43559	SbiRTX430	SbiRTX430	SbiRTX430	PF06200 P	PTHR33077	PTHR33077:SF17			
PAC:43561	SbiRTX430	SbiRTX430	SbiRTX430	PF12695	PTHR2302	EC:3.1.1.1	KOG1515		
PAC:43560	SbiRTX430	SbiRTX430	SbiRTX430	PF01428 P	PTHR10634	PTHR1063	KOG3173		GO:000367
PAC:43560	SbiRTX430	SbiRTX430	SbiRTX430	PF01428 P	PTHR10634	PTHR1063	KOG3173		GO:000367
PAC:43555	SbiRTX430	SbiRTX430	SbiRTX430.02G04990		PTHR33059	PTHR33059:SF26			
PAC:43555	SbiRTX430	SbiRTX430	SbiRTX430.02G04990		PTHR33059	PTHR33059:SF26			
PAC:43558	SbiRTX430	SbiRTX430	SbiRTX430	PF00743	PTHR2302	EC:1.14.13	KOG1399		GO:00044
PAC:43559	SbiRTX430	SbiRTX430	SbiRTX430	PF00504	PTHR14154	PTHR14154:SF5			
PAC:43559	SbiRTX430	SbiRTX430	SbiRTX430	PF00170 P	PTHR22952	PTHR22952:SF213			GO:00037
PAC:43557	SbiRTX430	SbiRTX430	SbiRTX430	PF00704	PTHR1117	EC:3.2.1.14		K01183	GO:000455
PAC:43555	SbiRTX430	SbiRTX430	SbiRTX430	PF03016	PTHR1106	EC:2.4.2.4	KOG1021		
PAC:43559	SbiRTX430	SbiRTX430	SbiRTX430	PF00957 P	PTHR21136	PTHR2113	KOG0859		GO:000681
PAC:43559	SbiRTX430	SbiRTX430	SbiRTX430	PF00957 P	PTHR21136	PTHR2113	KOG0859	K08511	GO:000681
PAC:43559	SbiRTX430	SbiRTX430	SbiRTX430	PF00957 P	PTHR21136	PTHR2113	KOG0859		GO:000681

PAC:43559	SbiRTX430	SbiRTX430	SbiRTX430	PF00957 P	PTHR21136	PTHR2113	KOG0859		GO:000681
PAC:43559	SbiRTX430	SbiRTX430	SbiRTX430	PF00957	PTHR21136	PTHR21136:SF87			GO:001602
PAC:43559	SbiRTX430	SbiRTX430	SbiRTX430	PF02365	PTHR31719	PTHR31719:SF27			GO:000367
PAC:43556	SbiRTX430	SbiRTX430	SbiRTX430	PF13415 P	PTHR23244	PTHR23244:SF276			GO:000551
PAC:43560	SbiRTX430	SbiRTX430	SbiRTX430.02G086800.1.p						
PAC:43558	SbiRTX430	SbiRTX430	SbiRTX430	PF00161 PF00652		EC:3.2.2.22			GO:001714
PAC:43558	SbiRTX430	SbiRTX430	SbiRTX430	PF00161 PF00652		EC:3.2.2.22			GO:001714
PAC:43558	SbiRTX430	SbiRTX430	SbiRTX430	PF01419 P	PTHR21495	PTHR21495:SF81			
PAC:43560	SbiRTX430	SbiRTX430	SbiRTX430.02G094500.1.p						
PAC:43555	SbiRTX430	SbiRTX430	SbiRTX430	PF00111 P	PTHR1190	EC:1.2.3.1	EC:1.2.3.7		GO:000382
PAC:43555	SbiRTX430	SbiRTX430	SbiRTX430	PF00111 P	PTHR1190	EC:1.2.3.1	EC:1.2.3.7		GO:000382
PAC:43555	SbiRTX430	SbiRTX430	SbiRTX430	PF00111 P	PTHR1190	EC:1.2.3.1	EC:1.2.3.7		GO:000382
PAC:43555	SbiRTX430	SbiRTX430	SbiRTX430	PF00111 P	PTHR1190	EC:1.2.3.1	EC:1.2.3.7		GO:000382
PAC:43555	SbiRTX430	SbiRTX430	SbiRTX430	PF00941 P	PTHR1190	EC:1.2.3.1	EC:1.2.3.7		GO:000382
PAC:43555	SbiRTX430	SbiRTX430	SbiRTX430	PF00941 P	PTHR1190	EC:1.2.3.1	EC:1.2.3.7		GO:000382
PAC:43559	SbiRTX430	SbiRTX430	SbiRTX430.02G11300	PTHR31032	PTHR31032:SF3				
PAC:43559	SbiRTX430	SbiRTX430	SbiRTX430.02G11300	PTHR31032	PTHR31032:SF3				
PAC:43556	SbiRTX430	SbiRTX430	SbiRTX430	PF05512	PTHR33294	PTHR33294:SF5			
PAC:43556	SbiRTX430	SbiRTX430	SbiRTX430	PF05512	PTHR33294	PTHR33294:SF5			
PAC:43556	SbiRTX430	SbiRTX430	SbiRTX430	PF05512	PTHR33294	PTHR33294:SF5			
PAC:43556	SbiRTX430	SbiRTX430	SbiRTX430	PF05512	PTHR33294	PTHR33294:SF5			
PAC:43557	SbiRTX430	SbiRTX430	SbiRTX430	PF03092	PTHR31585	PTHR31585:SF12			GO:000681
PAC:43556	SbiRTX430	SbiRTX430	SbiRTX430.02G125900.1.p						
PAC:43559	SbiRTX430	SbiRTX430	SbiRTX430	PF00067	PTHR2429	EC:1.14.13	KOG0156	K00487	GO:000449
PAC:43559	SbiRTX430	SbiRTX430	SbiRTX430	PF05910	PTHR31972	PTHR31972:SF5			
PAC:43560	SbiRTX430	SbiRTX430	SbiRTX430	PF00125	PTHR23428	PTHR23428:SF50		K11252	GO:000078
PAC:43556	SbiRTX430	SbiRTX430	SbiRTX430	PF00168	PTHR32246	PTHR32246:SF17			GO:000551
PAC:43559	SbiRTX430	SbiRTX430	SbiRTX430	PF07986 P	PTHR15139		KOG2512		GO:000090
PAC:43561	SbiRTX430	SbiRTX430	SbiRTX430.02G14620	PTHR33919	PTHR33919:SF1				
PAC:43559	SbiRTX430	SbiRTX430	SbiRTX430.02G149500.1.p						
PAC:43560	SbiRTX430	SbiRTX430	SbiRTX430	PF00504	PTHR21649	PTHR21649:SF24		K14172	GO:000976
PAC:43560	SbiRTX430	SbiRTX430	SbiRTX430	PF07714 P	PTHR2700	EC:2.7.10.	KOG1187		GO:000467
PAC:43560	SbiRTX430	SbiRTX430	SbiRTX430	PF07714 P	PTHR2700	EC:2.7.10.	KOG1187		GO:000467
PAC:43561	SbiRTX430	SbiRTX430	SbiRTX430	PF01020 P	PTHR10666		KOG0003	K02927	GO:000373
PAC:43559	SbiRTX430	SbiRTX430	SbiRTX430	PF08392 P	PTHR31561	PTHR31561:SF29		K15397	GO:000382
PAC:43556	SbiRTX430	SbiRTX430	SbiRTX430	PF02358	PTHR1078	EC:3.1.3.12		K01087	GO:000382
PAC:43556	SbiRTX430	SbiRTX430	SbiRTX430.02G190500.1.p						
PAC:43556	SbiRTX430	SbiRTX430	SbiRTX430.02G190500.2.p						
PAC:43556	SbiRTX430	SbiRTX430	SbiRTX430.02G190500.3.p						
PAC:43560	SbiRTX430	SbiRTX430	SbiRTX430	PF13085 P	PTHR1192	EC:1.3.5.1	KOG3049	K00235	GO:000609
PAC:43560	SbiRTX430	SbiRTX430	SbiRTX430	PF14009	PTHR33413	PTHR33413:SF4			
PAC:43560	SbiRTX430	SbiRTX430	SbiRTX430	PF04828	PTHR28620	PTHR2862	KOG4192		GO:000815
PAC:43555	SbiRTX430	SbiRTX430	SbiRTX430	PF03106	PTHR31429	PTHR31429:SF3			GO:000370
PAC:43555	SbiRTX430	SbiRTX430	SbiRTX430	PF03106	PTHR31429	PTHR31429:SF3			GO:000370

PAC:43558	SbiRTX430	SbiRTX430	SbiRTX430	PF00155	PTHR1175	EC:2.6.1.2		K00814	GO:000382
PAC:43558	SbiRTX430	SbiRTX430	SbiRTX430	PF00155	PTHR1175	EC:2.6.1.2			GO:000382
PAC:43559	SbiRTX430	SbiRTX430	SbiRTX430	PF13016	PTHR33454				GO:004573
PAC:43558	SbiRTX430	SbiRTX430	SbiRTX430	PF00847	PTHR31190	PTHR31190:SF8		K09286	GO:000367
PAC:43558	SbiRTX430	SbiRTX430	SbiRTX430	PF00847	PTHR31190	PTHR31190:SF8		K09286	GO:000367
PAC:43558	SbiRTX430	SbiRTX430	SbiRTX430	PF00847	PTHR31190	PTHR31190:SF8			GO:000367
PAC:43558	SbiRTX430	SbiRTX430	SbiRTX430	PF00067	PTHR2429	EC:1.14.13.21			GO:000449
PAC:43555	SbiRTX430	SbiRTX430	SbiRTX430.02G22620		PTHR33237	PTHR33237:SF9			
PAC:43560	SbiRTX430	SbiRTX430	SbiRTX430	PF03999	PTHR19321	PTHR1932	KOG4302	K16732	GO:000022
PAC:43560	SbiRTX430	SbiRTX430	SbiRTX430	PF03999	PTHR19321	PTHR1932	KOG4302	K16732	GO:000022
PAC:43560	SbiRTX430	SbiRTX430	SbiRTX430	PF03999	PTHR19321	PTHR1932	KOG4302	K16732	GO:000022
PAC:43559	SbiRTX430	SbiRTX430	SbiRTX430	PF00447	PTHR10015	PTHR10015:SF204		K09419	GO:000370
PAC:43558	SbiRTX430	SbiRTX430	SbiRTX430	PF12695	PTHR2302	EC:3.1.1.1	KOG1515		
PAC:43558	SbiRTX430	SbiRTX430	SbiRTX430	PF12695	PTHR2302	EC:3.1.1.1	EC:4.2.1.105		
PAC:43560	SbiRTX430	SbiRTX430	SbiRTX430	PF12695	PTHR2302	EC:3.1.1.1	KOG1515		
PAC:43561	SbiRTX430	SbiRTX430	SbiRTX430	PF00076	PTHR24012	PTHR2401	KOG0148		GO:000016
PAC:43561	SbiRTX430	SbiRTX430	SbiRTX430	PF00076	PTHR24012	PTHR2401	KOG0131		GO:000016
PAC:43555	SbiRTX430	SbiRTX430	SbiRTX430	PF04770	PTHR31948	PTHR31948:SF20			GO:000367
PAC:43557	SbiRTX430	SbiRTX430	SbiRTX430	PF13041	PTHR24015	PTHR24015:SF591		K17710	GO:000551
PAC:43557	SbiRTX430	SbiRTX430	SbiRTX430	PF13041	PTHR24015	PTHR24015:SF591			GO:000551
PAC:43557	SbiRTX430	SbiRTX430	SbiRTX430	PF13041	PTHR24015	PTHR24015:SF591			GO:000551
PAC:43557	SbiRTX430	SbiRTX430	SbiRTX430	PF07847	PTHR2296	EC:1.13.11	KOG4281	K10712	GO:001670
PAC:43557	SbiRTX430	SbiRTX430	SbiRTX430	PF00141	PTHR3138	EC:1.11.1.7		K00430	GO:000460
PAC:43557	SbiRTX430	SbiRTX430	SbiRTX430	PF00141	PTHR3138	EC:1.11.1.7		K00430	GO:000460
PAC:43557	SbiRTX430	SbiRTX430	SbiRTX430	PF00141	PTHR3138	EC:1.11.1.7			GO:000460
PAC:43557	SbiRTX430	SbiRTX430	SbiRTX430	PF00141	PTHR3138	EC:1.11.1.7			GO:000460
PAC:43557	SbiRTX430	SbiRTX430	SbiRTX430	PF00141	PTHR3138	EC:1.11.1.7		K00430	GO:000460
PAC:43559	SbiRTX430	SbiRTX430	SbiRTX430	PF00005 P	PTHR1924	EC:3.6.3.3	KOG0061		GO:000552
PAC:43556	SbiRTX430	SbiRTX430	SbiRTX430	PF03015 P	PTHR1101	EC:1.2.1.4	KOG1221	K13356	GO:008001
PAC:43561	SbiRTX430	SbiRTX430	SbiRTX430	PF00069 P	PTHR2700	EC:2.7.11.	KOG1187		GO:000467
PAC:43561	SbiRTX430	SbiRTX430	SbiRTX430	PF13639	PTHR14155	PTHR14155:SF184		K16285	GO:000551
PAC:43558	SbiRTX430	SbiRTX430	SbiRTX430	PF13578	PTHR1050	EC:2.1.1.1	KOG1663	K00588	GO:000817
PAC:43558	SbiRTX430	SbiRTX430	SbiRTX430.02G25300		PTHR34055	PTHR34055:SF2			
PAC:43555	SbiRTX430	SbiRTX430	SbiRTX430	PF00642 P	PTHR12620	PTHR1262	KOG2202	K12836	GO:000016
PAC:43555	SbiRTX430	SbiRTX430	SbiRTX430	PF00642 P	PTHR12620	PTHR1262	KOG2202	K12836	GO:000016
PAC:43555	SbiRTX430	SbiRTX430	SbiRTX430	PF00642 P	PTHR12620	PTHR1262	KOG2202	K12836	GO:000016
PAC:43555	SbiRTX430	SbiRTX430	SbiRTX430	PF00642 P	PTHR12620	PTHR1262	KOG2202	K12836	GO:000016
PAC:43555	SbiRTX430	SbiRTX430	SbiRTX430	PF00642 P	PTHR12620	PTHR1262	KOG2202	K12836	GO:000016
PAC:43555	SbiRTX430	SbiRTX430	SbiRTX430	PF00642 P	PTHR12620	PTHR1262	KOG2202	K12836	GO:000016
PAC:43556	SbiRTX430	SbiRTX430	SbiRTX430	PF00182	PTHR22595	PTHR2259	KOG4742		GO:000456
PAC:43556	SbiRTX430	SbiRTX430	SbiRTX430	PF00182	PTHR22595	PTHR2259	KOG4742		GO:000456
PAC:43559	SbiRTX430	SbiRTX430	SbiRTX430.02G25730		PTHR35510	PTHR35510:SF1			
PAC:43561	SbiRTX430	SbiRTX430	SbiRTX430	PF04654	PTHR31881	PTHR31881:SF6			
PAC:43561	SbiRTX430	SbiRTX430	SbiRTX430	PF04654	PTHR31881	PTHR31881:SF6			

PAC:43555	SbiRTX430	SbiRTX430	SbiRTX430.02G25930(		PTHR3516:	EC:3.4.21.72			
PAC:43557	SbiRTX430	SbiRTX430	SbiRTX430	PF00332 P	PTHR3222	EC:3.2.1.39			GO:00045!
PAC:43556	SbiRTX430	SbiRTX430	SbiRTX430	PF02181	PTHR23213	PTHR23213:SF246			
PAC:43558	SbiRTX430	SbiRTX430	SbiRTX430	PF01120	PTHR1003(	EC:3.2.1.51		K01206	GO:000456
PAC:43560	SbiRTX430	SbiRTX430	SbiRTX430	PF01554	PTHR11206	PTHR1120(	KOG1347	K03327	GO:000685
PAC:43560	SbiRTX430	SbiRTX430	SbiRTX430	PF00447	PTHR10015	PTHR10015:SF194		K09419	GO:00037(
PAC:43558	SbiRTX430	SbiRTX430	SbiRTX430	PF00067	PTHR2429	EC:1.14.13	KOG0156		GO:00044!
PAC:43560	SbiRTX430	SbiRTX430	SbiRTX430.02G28730(		PTHR35167	PTHR35167:SF2			
PAC:43557	SbiRTX430	SbiRTX430	SbiRTX430	PF00278 P	PTHR1148:	EC:4.1.1.1	KOG0622	K01581	GO:000382
PAC:43557	SbiRTX430	SbiRTX430	SbiRTX430.02G29680(		PTHR34484	PTHR34484:SF1			
PAC:43560	SbiRTX430	SbiRTX430	SbiRTX430	PF16913	PTHR31376	PTHR31376:SF10			GO:00052
PAC:43559	SbiRTX430	SbiRTX430	SbiRTX430	PF00722 P	PTHR3106:	EC:2.4.1.207		K08235	GO:00045!
PAC:43555	SbiRTX430	SbiRTX430	SbiRTX430	PF00112 P	PTHR1241	EC:3.4.22.	KOG1542	K01373	GO:00065(
PAC:43557	SbiRTX430	SbiRTX430	SbiRTX430	PF00201	PTHR11926	PTHR11926:SF200			GO:00081!
PAC:43559	SbiRTX430	SbiRTX430	SbiRTX430	PF00330 P	PTHR1167(	EC:4.2.1.3		K01681	GO:000815
PAC:43559	SbiRTX430	SbiRTX430	SbiRTX430	PF00330 P	PTHR1167(	EC:4.2.1.3		K01681	GO:000815
PAC:43556	SbiRTX430	SbiRTX430	SbiRTX430	PF03151	PTHR11132	PTHR1113	KOG1441	K15283	GO:000521
PAC:43556	SbiRTX430	SbiRTX430	SbiRTX430	PF01590	PTHR2442:	EC:2.7.11.	KOG0519	K14509	GO:000016
PAC:43556	SbiRTX430	SbiRTX430	SbiRTX430	PF01590	PTHR2442:	EC:2.7.11.	KOG0519	K14509	GO:000016
PAC:43556	SbiRTX430	SbiRTX430	SbiRTX430	PF00332 P	PTHR3222	EC:3.2.1.39			GO:000455
PAC:43556	SbiRTX430	SbiRTX430	SbiRTX430	PF00332 P	PTHR3222	EC:3.2.1.39			GO:000455
PAC:43560	SbiRTX430	SbiRTX430	SbiRTX430	PF06749	PTHR31769	PTHR31769:SF13			
PAC:43561	SbiRTX430	SbiRTX430	SbiRTX430.02G33880(		PTHR33735	PTHR33735:SF6			
PAC:43557	SbiRTX430	SbiRTX430	SbiRTX430.02G34640(		PTHR33728	PTHR33728:SF1			GO:00425!
PAC:43557	SbiRTX430	SbiRTX430	SbiRTX430	PF00082 P	PTHR1079!	EC:3.4.21.25			GO:000425
PAC:43558	SbiRTX430	SbiRTX430	SbiRTX430.02G36160(		PTHR33595	PTHR33595:SF1			
PAC:43556	SbiRTX430	SbiRTX430	SbiRTX430	PF00141	PTHR3138	EC:1.11.1.7		K00430	GO:00046(
PAC:43559	SbiRTX430	SbiRTX430	SbiRTX430	PF06075	PTHR31928	PTHR31928:SF5			
PAC:43558	SbiRTX430	SbiRTX430	SbiRTX430	PF03195	PTHR31304	PTHR31304:SF4			
PAC:43556	SbiRTX430	SbiRTX430	SbiRTX430	PF13561	PTHR2432:	EC:1.1.1.1	KOG0725		GO:000866
PAC:43557	SbiRTX430	SbiRTX430	SbiRTX430	PF13943	PTHR34362	PTHR34362:SF1			
PAC:43557	SbiRTX430	SbiRTX430	SbiRTX430	PF07983	PTHR32227	PTHR32227:SF83			
PAC:43560	SbiRTX430	SbiRTX430	SbiRTX430	PF12695	PTHR2302	EC:3.1.1.1	KOG1515		
PAC:43556	SbiRTX430	SbiRTX430	SbiRTX430.02G37640(		PTHR22814	PTHR2281	KOG1603		GO:00300(
PAC:43557	SbiRTX430	SbiRTX430	SbiRTX430	PF01095	PTHR3132	EC:3.1.1.11		K01051	GO:00056
PAC:43557	SbiRTX430	SbiRTX430	SbiRTX430	PF01095	PTHR3132	EC:3.1.1.11			GO:00056
PAC:43555	SbiRTX430	SbiRTX430	SbiRTX430	PF07714	PTHR2700	EC:2.7.11.	KOG1187		GO:000467
PAC:43555	SbiRTX430	SbiRTX430	SbiRTX430.02G38110(		PTHR37256				
PAC:43556	SbiRTX430	SbiRTX430	SbiRTX430	PF01070	PTHR1057	EC:1.1.3.15		K11517	GO:00038:
PAC:43556	SbiRTX430	SbiRTX430	SbiRTX430	PF01070	PTHR1057	EC:1.1.3.15		K11517	GO:00038:
PAC:43556	SbiRTX430	SbiRTX430	SbiRTX430	PF01070	PTHR1057	EC:1.1.3.15			GO:00038:
PAC:43560	SbiRTX430	SbiRTX430	SbiRTX430	PF13202	PTHR10891	PTHR10891:SF597			GO:00055(
PAC:43557	SbiRTX430	SbiRTX430	SbiRTX430	PF00561	PTHR1099:	EC:3.1.1.3	KOG1454		GO:00038:
PAC:43558	SbiRTX430	SbiRTX430	SbiRTX430	PF13266	PTHR3113:	EC:1.14.11.33			

PAC:43558	SbiRTX430	SbiRTX430	SbiRTX430	PF13266	PTHR3113	EC:1.14.11.33			
PAC:43558	SbiRTX430	SbiRTX430	SbiRTX430	PF13266	PTHR31132	PTHR31132:SF7			
PAC:43556	SbiRTX430	SbiRTX430	SbiRTX430.02G39130		PTHR34776	PTHR34776:SF1			
PAC:43560	SbiRTX430	SbiRTX430	SbiRTX430.02G39190		PTHR35463	PTHR35463:SF2			
PAC:43560	SbiRTX430	SbiRTX430	SbiRTX430.02G39190		PTHR35463	PTHR35463:SF2			
PAC:43560	SbiRTX430	SbiRTX430	SbiRTX430.02G39190		PTHR35463	PTHR35463:SF2			
PAC:43557	SbiRTX430	SbiRTX430	SbiRTX430	PF00249	PTHR10641	PTHR10641:SF491		K09422	GO:000367
PAC:43559	SbiRTX430	SbiRTX430	SbiRTX430	PF13639	PTHR22763	PTHR22763:SF72			GO:000551
PAC:43556	SbiRTX430	SbiRTX430	SbiRTX430	PF08534 P	PTHR1068	EC:1.11.1.	KOG0854	K11188	GO:001649
PAC:43556	SbiRTX430	SbiRTX430	SbiRTX430	PF00578 P	PTHR1068	EC:1.11.1.	KOG0854		GO:001620
PAC:43556	SbiRTX430	SbiRTX430	SbiRTX430	PF00578 P	PTHR1068	EC:1.11.1.	KOG0854		GO:001620
PAC:43556	SbiRTX430	SbiRTX430	SbiRTX430	PF00578 P	PTHR1068	EC:1.11.1.	KOG0854		GO:001620
PAC:43559	SbiRTX430	SbiRTX430	SbiRTX430	PF13561	PTHR2432	EC:1.1.1.1	KOG1205		
PAC:43556	SbiRTX430	SbiRTX430	SbiRTX430	PF00170	PTHR22952	PTHR22952:SF139			GO:000370
PAC:43561	SbiRTX430	SbiRTX430	SbiRTX430	PF12681	PTHR1037	EC:4.4.1.5	KOG2944		
PAC:43560	SbiRTX430	SbiRTX430	SbiRTX430	PF13561	PTHR2432	EC:1.1.1.1	KOG0725		GO:000402
PAC:43558	SbiRTX430	SbiRTX430	SbiRTX430	PF05755	PTHR33732	PTHR33732:SF1			
PAC:43556	SbiRTX430	SbiRTX430	SbiRTX430	PF00201	PTHR11926	PTHR11926:SF205			GO:000815
PAC:43561	SbiRTX430	SbiRTX430	SbiRTX430.02G42380		PTHR35985	PTHR35985:SF1			
PAC:43560	SbiRTX430	SbiRTX430	SbiRTX430	PF04844	PTHR33057	PTHR33057:SF27			
PAC:43557	SbiRTX430	SbiRTX430	SbiRTX430	PF02365	PTHR31719	PTHR31719:SF26			GO:000367
PAC:43558	SbiRTX430	SbiRTX430	SbiRTX430	PF02365	PTHR31744	PTHR31744:SF20			GO:000367
PAC:43561	SbiRTX430	SbiRTX430	SbiRTX430	PF00046	PTHR24326	PTHR24326:SF280			GO:000367
PAC:43539	SbiRTX430	SbiRTX430	SbiRTX430	PF05558	PTHR38522	PTHR38522:SF1			GO:004665
PAC:43539	SbiRTX430	SbiRTX430	SbiRTX430	PF05558	PTHR38522	PTHR38522:SF1			GO:004665
PAC:43539	SbiRTX430	SbiRTX430	SbiRTX430	PF05558	PTHR38522	PTHR38522:SF1			GO:004665
PAC:43542	SbiRTX430	SbiRTX430	SbiRTX430	PF00067	PTHR2429	EC:1.14.13	KOG0156		GO:000449
PAC:43543	SbiRTX430	SbiRTX430	SbiRTX430.03G01180		PTHR33929	PTHR33929:SF1			
PAC:43542	SbiRTX430	SbiRTX430	SbiRTX430	PF03168	PTHR31459	PTHR31459:SF4			GO:000926
PAC:43539	SbiRTX430	SbiRTX430	SbiRTX430	PF00657	PTHR2283	EC:3.1.1.1			GO:001678
PAC:43543	SbiRTX430	SbiRTX430	SbiRTX430	PF03321	PTHR31901	PTHR31901:SF13		K14487	
PAC:43543	SbiRTX430	SbiRTX430	SbiRTX430	PF03321	PTHR31901	PTHR31901:SF13		K14487	
PAC:43538	SbiRTX430	SbiRTX430	SbiRTX430	PF01965	PTHR11019	PTHR1101	KOG2764		
PAC:43540	SbiRTX430	SbiRTX430	SbiRTX430	PF12222	PTHR3110	EC:3.5.1.52			
PAC:43544	SbiRTX430	SbiRTX430	SbiRTX430	PF00141	PTHR3138	EC:1.11.1.7		K00430	GO:000460
PAC:43538	SbiRTX430	SbiRTX430	SbiRTX430	PF00462	PTHR10168	PTHR1016	KOG1752	K03676	GO:000905
PAC:43540	SbiRTX430	SbiRTX430	SbiRTX430	PF00642	PTHR14493	PTHR14493:SF41			GO:004687
PAC:43542	SbiRTX430	SbiRTX430	SbiRTX430	PF00249	PTHR10641	PTHR10641:SF607		K09422	GO:000367
PAC:43542	SbiRTX430	SbiRTX430	SbiRTX430	PF02668	PTHR10696	PTHR10696:SF21			GO:001649
PAC:43541	SbiRTX430	SbiRTX430	SbiRTX430	PF00657	PTHR2283	EC:3.1.1.1			GO:001678
PAC:43544	SbiRTX430	SbiRTX430	SbiRTX430	PF01764	PTHR3140	EC:3.1.1.3	KOG4569		GO:000662
PAC:43542	SbiRTX430	SbiRTX430	SbiRTX430	PF00011	PTHR11527	PTHR11527:SF93		K13993	
PAC:43542	SbiRTX430	SbiRTX430	SbiRTX430.03G055100.1.p						
PAC:43542	SbiRTX430	SbiRTX430	SbiRTX430.03G055100.2.p						

PAC:43538	SbiRTX430	SbiRTX430	SbiRTX430.03G05520		PTHR34372	PTHR34372:SF2			GO:000574
PAC:43541	SbiRTX430	SbiRTX430	SbiRTX430	PF05691	PTHR3126	EC:2.4.1.82		K06617	GO:000382
PAC:43544	SbiRTX430	SbiRTX430	SbiRTX430	PF00450	PTHR1180	EC:3.4.16.6		K16297	GO:000418
PAC:43543	SbiRTX430	SbiRTX430	SbiRTX430	PF04749	PTHR15907	PTHR15907:SF58			
PAC:43543	SbiRTX430	SbiRTX430	SbiRTX430.03G072100.1.p						
PAC:43541	SbiRTX430	SbiRTX430	SbiRTX430	PF00582	PTHR31964	PTHR31964:SF59			GO:000695
PAC:43541	SbiRTX430	SbiRTX430	SbiRTX430	PF00582	PTHR31964	PTHR31964:SF59			GO:000695
PAC:43541	SbiRTX430	SbiRTX430	SbiRTX430	PF00582	PTHR31964	PTHR31964:SF59			GO:000695
PAC:43541	SbiRTX430	SbiRTX430	SbiRTX430	PF00582	PTHR31964	PTHR31964:SF59			GO:000695
PAC:43541	SbiRTX430	SbiRTX430	SbiRTX430	PF05623	PTHR31343	PTHR31343:SF8			
PAC:43538	SbiRTX430	SbiRTX430	SbiRTX430	PF00011	PTHR11527	PTHR1152	KOG0710	K13993	
PAC:43542	SbiRTX430	SbiRTX430	SbiRTX430	PF00011	PTHR11527	PTHR1152	KOG0710	K13993	
PAC:43540	SbiRTX430	SbiRTX430	SbiRTX430	PF00011	PTHR11527	PTHR11527:SF141		K13993	
PAC:43539	SbiRTX430	SbiRTX430	SbiRTX430	PF00011	PTHR11527	PTHR11527:SF123		K13993	
PAC:43539	SbiRTX430	SbiRTX430	SbiRTX430	PF15699					
PAC:43541	SbiRTX430	SbiRTX430	SbiRTX430.03G096700.1.p						
PAC:43543	SbiRTX430	SbiRTX430	SbiRTX430	PF01161	PTHR11362	PTHR1136	KOG3346		
PAC:43543	SbiRTX430	SbiRTX430	SbiRTX430	PF13855	PTHR27000	PTHR27000:SF18			GO:000551
PAC:43538	SbiRTX430	SbiRTX430	SbiRTX430	PF02671	PTHR12346				GO:000635
PAC:43544	SbiRTX430	SbiRTX430	SbiRTX430	PF01370	PTHR1036	EC:1.3.1.45			GO:000382
PAC:43538	SbiRTX430	SbiRTX430	SbiRTX430	PF00177	PTHR11205	PTHR1120	KOG3291	K02989	GO:000373
PAC:43540	SbiRTX430	SbiRTX430	SbiRTX430	PF00646 P	PTHR32212	PTHR32212:SF116			GO:000551
PAC:43540	SbiRTX430	SbiRTX430	SbiRTX430	PF00646 P	PTHR32212	PTHR32212:SF116			GO:000551
PAC:43540	SbiRTX430	SbiRTX430	SbiRTX430	PF00031 P	PTHR11413	PTHR11413:SF55			GO:000486
PAC:43539	SbiRTX430	SbiRTX430	SbiRTX430.03G135000.1.p						
PAC:43539	SbiRTX430	SbiRTX430	SbiRTX430	PF00141	PTHR3123	EC:1.11.1.7		K00430	GO:000460
PAC:43544	SbiRTX430	SbiRTX430	SbiRTX430.03G135600.1.p						
PAC:43544	SbiRTX430	SbiRTX430	SbiRTX430.03G138900.1.p						
PAC:43540	SbiRTX430	SbiRTX430	SbiRTX430.03G13990		PTHR36394	PTHR36394:SF1			
PAC:43538	SbiRTX430	SbiRTX430	SbiRTX430.03G144500.1.p						
PAC:43538	SbiRTX430	SbiRTX430	SbiRTX430	PF01694	PTHR1291	EC:3.4.21.	KOG2632	K09651	GO:000425
PAC:43541	SbiRTX430	SbiRTX430	SbiRTX430	PF00005 P	PTHR2422	EC:3.6.3.44		K05658	GO:000552
PAC:43541	SbiRTX430	SbiRTX430	SbiRTX430	PF00005 P	PTHR2422	EC:3.6.3.44		K05658	GO:000552
PAC:43541	SbiRTX430	SbiRTX430	SbiRTX430	PF00005 P	PTHR2422	EC:3.6.3.44		K05658	GO:000552
PAC:43541	SbiRTX430	SbiRTX430	SbiRTX430	PF00005 P	PTHR2422	EC:3.6.3.44			GO:000552
PAC:43541	SbiRTX430	SbiRTX430	SbiRTX430	PF00005 P	PTHR2422	EC:3.6.3.44			GO:000552
PAC:43541	SbiRTX430	SbiRTX430	SbiRTX430	PF00005 P	PTHR2422	EC:3.6.3.44		K05658	GO:000552
PAC:43541	SbiRTX430	SbiRTX430	SbiRTX430	PF00005 P	PTHR2422	EC:3.6.3.44			GO:000552
PAC:43541	SbiRTX430	SbiRTX430	SbiRTX430	PF00005 P	PTHR2422	EC:3.6.3.44			GO:000552
PAC:43540	SbiRTX430	SbiRTX430	SbiRTX430	PF02466	PTHR15371	PTHR15371:SF5			
PAC:43540	SbiRTX430	SbiRTX430	SbiRTX430	PF02466	PTHR15371	PTHR15371:SF5			
PAC:43538	SbiRTX430	SbiRTX430	SbiRTX430	PF04667	PTHR34804	PTHR34804:SF2			
PAC:43538	SbiRTX430	SbiRTX430	SbiRTX430	PF04667	PTHR34804	PTHR34804:SF2			
PAC:43543	SbiRTX430	SbiRTX430	SbiRTX430	PF00249	PTHR10641	PTHR10641:SF651		K09422	GO:000367

PAC:43542	SbiRTX430	SbiRTX430	SbiRTX430.03G156100.1.p						
PAC:43540	SbiRTX430	SbiRTX430	SbiRTX430	PF00847	PTHR31190	PTHR31190:SF14			GO:000367
PAC:43538	SbiRTX430	SbiRTX430	SbiRTX430	PF00076	PTHR24012	PTHR2401	KOG0105	K12890	GO:000016
PAC:43538	SbiRTX430	SbiRTX430	SbiRTX430	PF00076	PTHR24012	PTHR2401	KOG0105	K12890	GO:000016
PAC:43538	SbiRTX430	SbiRTX430	SbiRTX430	PF00076	PTHR24012	PTHR2401	KOG0105	K12890	GO:000016
PAC:43538	SbiRTX430	SbiRTX430	SbiRTX430	PF00076	PTHR24012	PTHR2401	KOG0105	K12890	GO:000016
PAC:43538	SbiRTX430	SbiRTX430	SbiRTX430	PF00076	PTHR24012	PTHR2401	KOG0105	K12890	GO:000016
PAC:43543	SbiRTX430	SbiRTX430	SbiRTX430	PF00141	PTHR3123	EC:1.11.1.7		K00430	GO:000460
PAC:43543	SbiRTX430	SbiRTX430	SbiRTX430.03G164000.1.p						GO:000551
PAC:43542	SbiRTX430	SbiRTX430	SbiRTX430	PF13401	PTHR23155	PTHR2315	KOG4658		
PAC:43541	SbiRTX430	SbiRTX430	SbiRTX430.03G16830		PTHR35717				
PAC:43540	SbiRTX430	SbiRTX430	SbiRTX430	PF02902	PTHR33018	PTHR33018:SF7			GO:000650
PAC:43540	SbiRTX430	SbiRTX430	SbiRTX430.03G16860		PTHR33018	PTHR33018:SF7			
PAC:43540	SbiRTX430	SbiRTX430	SbiRTX430	PF03152	PTHR12555	PTHR1255	KOG1816		GO:000484
PAC:43541	SbiRTX430	SbiRTX430	SbiRTX430.03G20350		PTHR36484	PTHR36484:SF2			
PAC:43541	SbiRTX430	SbiRTX430	SbiRTX430	PF00447	PTHR10015	PTHR1001	KOG0627	K09419	GO:000370
PAC:43541	SbiRTX430	SbiRTX430	SbiRTX430	PF00026	PTHR1368	EC:3.4.23.	KOG1339		GO:000419
PAC:43540	SbiRTX430	SbiRTX430	SbiRTX430.03G232400.1.p						
PAC:43540	SbiRTX430	SbiRTX430	SbiRTX430.03G232400.2.p						
PAC:43541	SbiRTX430	SbiRTX430	SbiRTX430	PF04862	PTHR31265	PTHR31265:SF3			
PAC:43539	SbiRTX430	SbiRTX430	SbiRTX430	PF00067	PTHR2428	EC:1.3.3.9	KOG0157		GO:000449
PAC:43539	SbiRTX430	SbiRTX430	SbiRTX430.03G24780		PTHR33640	PTHR33640:SF3			
PAC:43540	SbiRTX430	SbiRTX430	SbiRTX430	PF00067	PTHR2428	EC:1.14.13	KOG0684	K05917	GO:000449
PAC:43540	SbiRTX430	SbiRTX430	SbiRTX430	PF00067	PTHR2428	EC:1.14.13	KOG0684	K05917	GO:000449
PAC:43541	SbiRTX430	SbiRTX430	SbiRTX430	PF00561	PTHR1099	EC:3.1.1.2	KOG1454		GO:000382
PAC:43539	SbiRTX430	SbiRTX430	SbiRTX430	PF01277	PTHR33203	PTHR33203:SF2			GO:001251
PAC:43544	SbiRTX430	SbiRTX430	SbiRTX430	PF03219	PTHR31187	PTHR31187:SF3		K03301	GO:000547
PAC:43541	SbiRTX430	SbiRTX430	SbiRTX430	PF01370	PTHR1036	EC:1.3.1.45			GO:000382
PAC:43542	SbiRTX430	SbiRTX430	SbiRTX430	PF00180	PTHR1182	EC:1.1.1.42		K00031	GO:000445
PAC:43542	SbiRTX430	SbiRTX430	SbiRTX430	PF00180	PTHR1182	EC:1.1.1.42		K00031	GO:000445
PAC:43544	SbiRTX430	SbiRTX430	SbiRTX430	PF00150	PTHR3145	EC:3.2.1.78		K19355	GO:000455
PAC:43542	SbiRTX430	SbiRTX430	SbiRTX430	PF00025	PTHR24073	PTHR2407	KOG0087	K07976	GO:000392
PAC:43540	SbiRTX430	SbiRTX430	SbiRTX430	PF03188	PTHR23130	PTHR2313	KOG4293		GO:001602
PAC:43541	SbiRTX430	SbiRTX430	SbiRTX430	PF13410 P	PTHR1126	EC:2.5.1.1	KOG0406	K00799	GO:000551
PAC:43540	SbiRTX430	SbiRTX430	SbiRTX430	PF00257	PTHR33346	PTHR33346:SF1			GO:000695
PAC:43539	SbiRTX430	SbiRTX430	SbiRTX430.03G29280		PTHR23241	PTHR23241:SF44			
PAC:43539	SbiRTX430	SbiRTX430	SbiRTX430.03G29280		PTHR23241	PTHR23241:SF44			
PAC:43540	SbiRTX430	SbiRTX430	SbiRTX430	PF04863 P	PTHR11751	PTHR11751:SF126			GO:000382
PAC:43540	SbiRTX430	SbiRTX430	SbiRTX430	PF04570	PTHR33059	PTHR33059:SF13			
PAC:43540	SbiRTX430	SbiRTX430	SbiRTX430	PF04570	PTHR33059	PTHR33059:SF13			
PAC:43541	SbiRTX430	SbiRTX430	SbiRTX430.03G30490		PTHR33210	PTHR33210:SF3			
PAC:43542	SbiRTX430	SbiRTX430	SbiRTX430	PF06884	PTHR31360	PTHR31360:SF2			
PAC:43542	SbiRTX430	SbiRTX430	SbiRTX430	PF06884	PTHR31360	PTHR31360:SF2			
PAC:43541	SbiRTX430	SbiRTX430	SbiRTX430	PF00266	PTHR1160	EC:2.8.1.7			GO:000382

PAC:43540	SbiRTX430	SbiRTX430	SbiRTX430	PF00447	PTHR10015	PTHR1001	KOG0627	K09419	GO:000370
PAC:43542	SbiRTX430	SbiRTX430	SbiRTX430	PF00390 P	PTHR2340	EC:1.1.1.40		K00029	GO:000447
PAC:43541	SbiRTX430	SbiRTX430	SbiRTX430	PF04520	PTHR33083				
PAC:43540	SbiRTX430	SbiRTX430	SbiRTX430	PF06884	PTHR31360	PTHR31360:SF1			GO:001018
PAC:43543	SbiRTX430	SbiRTX430	SbiRTX430	PF00847	PTHR31190	PTHR31190:SF20			GO:000367
PAC:43538	SbiRTX430	SbiRTX430	SbiRTX430	PF10252	PTHR22055	PTHR2205	KOG3375		
PAC:43543	SbiRTX430	SbiRTX430	SbiRTX430	PF14144	PTHR22952	PTHR22952:SF176			GO:000635
PAC:43542	SbiRTX430	SbiRTX430	SbiRTX430	PF00069 P	PTHR2434	EC:2.7.11.	KOG0583	K07198	GO:000467
PAC:43541	SbiRTX430	SbiRTX430	SbiRTX430.03G33300		PTHR34454	PTHR34454:SF2			
PAC:43542	SbiRTX430	SbiRTX430	SbiRTX430	PF03007 P	PTHR3165	EC:2.3.1.20	EC:2.3.1.75		GO:000414
PAC:43538	SbiRTX430	SbiRTX430	SbiRTX430	PF02574	PTHR2109	EC:2.1.1.1	KOG1579	K00547	GO:000573
PAC:43538	SbiRTX430	SbiRTX430	SbiRTX430	PF02574	PTHR2109	EC:2.1.1.1	KOG1579		
PAC:43541	SbiRTX430	SbiRTX430	SbiRTX430	PF07123	PTHR34552				GO:000950
PAC:43541	SbiRTX430	SbiRTX430	SbiRTX430	PF07123	PTHR34552			K02721	GO:000950
PAC:43538	SbiRTX430	SbiRTX430	SbiRTX430	PF03126	PTHR13115	PTHR13115:SF9			GO:000367
PAC:43538	SbiRTX430	SbiRTX430	SbiRTX430	PF03126	PTHR13115	PTHR13115:SF9			GO:000367
PAC:43543	SbiRTX430	SbiRTX430	SbiRTX430	PF02298	PTHR33021	PTHR33021:SF29			GO:000905
PAC:43539	SbiRTX430	SbiRTX430	SbiRTX430	PF01925	PTHR14255	PTHR14255:SF10			GO:001602
PAC:43539	SbiRTX430	SbiRTX430	SbiRTX430	PF01925	PTHR14255	PTHR14255:SF10			GO:001602
PAC:43543	SbiRTX430	SbiRTX430	SbiRTX430	PF02136	PTHR12612	PTHR1261	KOG2104		GO:000562
PAC:43543	SbiRTX430	SbiRTX430	SbiRTX430	PF00013	PTHR12826	PTHR1282	KOG3273	K11884	GO:000367
PAC:43543	SbiRTX430	SbiRTX430	SbiRTX430	PF00847	PTHR31677	PTHR31677:SF19			GO:000367
PAC:43543	SbiRTX430	SbiRTX430	SbiRTX430	PF14215	PTHR13902	PTHR13902:SF3			
PAC:43544	SbiRTX430	SbiRTX430	SbiRTX430	PF12937	PTHR31348	PTHR31348:SF3			GO:000367
PAC:43539	SbiRTX430	SbiRTX430	SbiRTX430	PF01357 P	PTHR31867	PTHR31867:SF22			GO:000557
PAC:43538	SbiRTX430	SbiRTX430	SbiRTX430	PF03222	PTHR22950	PTHR2295	KOG1305		GO:000333
PAC:43538	SbiRTX430	SbiRTX430	SbiRTX430	PF03222	PTHR22950	PTHR2295	KOG1305		GO:000333
PAC:43538	SbiRTX430	SbiRTX430	SbiRTX430	PF01490	PTHR22950	PTHR2295	KOG1305		
PAC:43543	SbiRTX430	SbiRTX430	SbiRTX430.03G371600.1.p						
PAC:43540	SbiRTX430	SbiRTX430	SbiRTX430.03G37300		PTHR33674	PTHR33674:SF1			GO:000367
PAC:43538	SbiRTX430	SbiRTX430	SbiRTX430	PF00314	PTHR31048	PTHR31048:SF7			
PAC:43539	SbiRTX430	SbiRTX430	SbiRTX430	PF00012	PTHR19375	PTHR19375:SF232		K03283	
PAC:43539	SbiRTX430	SbiRTX430	SbiRTX430	PF00012	PTHR19375	PTHR19375:SF232			
PAC:43539	SbiRTX430	SbiRTX430	SbiRTX430	PF00012	PTHR19375	PTHR19375:SF232		K03283	
PAC:43541	SbiRTX430	SbiRTX430	SbiRTX430	PF00481	PTHR1383	EC:3.1.3.1	KOG0698	K14497	GO:000382
PAC:43541	SbiRTX430	SbiRTX430	SbiRTX430	PF00481	PTHR1383	EC:3.1.3.16			GO:000382
PAC:43541	SbiRTX430	SbiRTX430	SbiRTX430	PF00481	PTHR1383	EC:3.1.3.1	KOG0698		GO:000382
PAC:43541	SbiRTX430	SbiRTX430	SbiRTX430	PF00481	PTHR1383	EC:3.1.3.16			GO:000382
PAC:43541	SbiRTX430	SbiRTX430	SbiRTX430	PF13962	PTHR24177	PTHR24177:SF51			
PAC:43540	SbiRTX430	SbiRTX430	SbiRTX430.03G38440		PTHR33738	PTHR33738:SF4			
PAC:43540	SbiRTX430	SbiRTX430	SbiRTX430.03G38440		PTHR33738	PTHR33738:SF4			
PAC:43540	SbiRTX430	SbiRTX430	SbiRTX430	PF04832	PTHR11220				GO:000486
PAC:43541	SbiRTX430	SbiRTX430	SbiRTX430.03G38830		PTHR34355				
PAC:43539	SbiRTX430	SbiRTX430	SbiRTX430	PF01490	PTHR22950	PTHR2295	KOG1303		

PAC:43542	SbiRTX430	SbiRTX430	SbiRTX430	PF00067	PTHR24296	PTHR2429	KOG0157			GO:00055
PAC:43539	SbiRTX430	SbiRTX430	SbiRTX430	PF00170	PTHR22952	PTHR22952:SF187		K14432		GO:00037
PAC:43539	SbiRTX430	SbiRTX430	SbiRTX430	PF00111	PTHR19370	PTHR19370:SF103		K02639		GO:000905
PAC:43540	SbiRTX430	SbiRTX430	SbiRTX430	PF00847	PTHR31190	PTHR31190:SF24				GO:00036
PAC:43541	SbiRTX430	SbiRTX430	SbiRTX430	PF07690	PTHR11654	PTHR11654:SF108				GO:00052
PAC:43541	SbiRTX430	SbiRTX430	SbiRTX430	PF01762 P	PTHR1121	EC:2.4.1.1	KOG2288			GO:00064
PAC:43541	SbiRTX430	SbiRTX430	SbiRTX430	PF01762 P	PTHR1121	EC:2.4.1.1	KOG2288			GO:00064
PAC:43544	SbiRTX430	SbiRTX430	SbiRTX430	PF02900	PTHR3009	EC:1.13.11.29		K15777		GO:00067
PAC:43544	SbiRTX430	SbiRTX430	SbiRTX430	PF03018	PTHR21495	PTHR21495:SF60				
PAC:43539	SbiRTX430	SbiRTX430	SbiRTX430	PF03405	PTHR3115	EC:1.14.19.2		K03921		GO:00066
PAC:43541	SbiRTX430	SbiRTX430	SbiRTX430	PF03171 P	PTHR10209	PTHR102	KOG0143			GO:00055
PAC:43544	SbiRTX430	SbiRTX430	SbiRTX430.03G41320		PTHR36023	PTHR36023:SF2				
PAC:43540	SbiRTX430	SbiRTX430	SbiRTX430	PF00305 P	PTHR1177	EC:1.13.11.58		K15718		GO:00055
PAC:43540	SbiRTX430	SbiRTX430	SbiRTX430	PF00305 P	PTHR1177	EC:1.13.11.58		K15718		GO:00055
PAC:43544	SbiRTX430	SbiRTX430	SbiRTX430	PF03188	PTHR23130	PTHR2313	KOG4293			GO:00160
PAC:43542	SbiRTX430	SbiRTX430	SbiRTX430	PF02536	PTHR13068	PTHR13068:SF39		K15032		GO:00036
PAC:43538	SbiRTX430	SbiRTX430	SbiRTX430	PF13920	PTHR15315	PTHR1531	KOG1039			GO:00055
PAC:43538	SbiRTX430	SbiRTX430	SbiRTX430	PF00274	PTHR1162	EC:4.1.2.13		K01623		GO:00038
PAC:43543	SbiRTX430	SbiRTX430	SbiRTX430	PF00112 P	PTHR1241	EC:3.4.22.	KOG1543			GO:00065
PAC:43544	SbiRTX430	SbiRTX430	SbiRTX430	PF12937						GO:000551
PAC:43540	SbiRTX430	SbiRTX430	SbiRTX430	PF14368	PTHR33122	PTHR33122:SF11				
PAC:43543	SbiRTX430	SbiRTX430	SbiRTX430	PF16851	PTHR37239	PTHR37239:SF1				
PAC:43542	SbiRTX430	SbiRTX430	SbiRTX430	PF05694	PTHR23300			K17285		GO:00055
PAC:43542	SbiRTX430	SbiRTX430	SbiRTX430	PF05694	PTHR23300					GO:00055
PAC:43539	SbiRTX430	SbiRTX430	SbiRTX430	PF13639	PTHR2304	EC:6.3.2.1	KOG0320			GO:00055
PAC:43539	SbiRTX430	SbiRTX430	SbiRTX430	PF13639	PTHR2304	EC:6.3.2.1	KOG0320			GO:00055
PAC:43541	SbiRTX430	SbiRTX430	SbiRTX430	PF11976	PTHR10666		KOG0001	K04551	K0	GO:00055
PAC:43541	SbiRTX430	SbiRTX430	SbiRTX430	PF11976	PTHR10666		KOG0001	K04551	K0	GO:00055
PAC:43541	SbiRTX430	SbiRTX430	SbiRTX430	PF11976	PTHR10666		KOG0001	K04551	K0	GO:00055
PAC:43541	SbiRTX430	SbiRTX430	SbiRTX430	PF11976	PTHR10666		KOG0001	K04551	K0	GO:00055
PAC:43541	SbiRTX430	SbiRTX430	SbiRTX430	PF11976	PTHR10666		KOG0001	K04551	K0	GO:00055
PAC:43541	SbiRTX430	SbiRTX430	SbiRTX430	PF11976	PTHR10666		KOG0001	K04551	K0	GO:00055
PAC:43542	SbiRTX430	SbiRTX430	SbiRTX430.03G43850		PTHR35132	PTHR35132:SF2				
PAC:43544	SbiRTX430	SbiRTX430	SbiRTX430	PF14364	PTHR35762	PTHR35762:SF2				
PAC:43538	SbiRTX430	SbiRTX430	SbiRTX430	PF02365	PTHR31989	PTHR31989:SF28				GO:00036
PAC:43543	SbiRTX430	SbiRTX430	SbiRTX430	PF02458	PTHR3164	EC:2.3.1.188		K15400		GO:00167
PAC:43543	SbiRTX430	SbiRTX430	SbiRTX430	PF07714	PTHR2700	EC:2.7.10.1 EC:2.7.11.1				GO:00046
PAC:43541	SbiRTX430	SbiRTX430	SbiRTX430	PF14541 P	PTHR13683	PTHR1368	KOG1339			GO:00041
PAC:43542	SbiRTX430	SbiRTX430	SbiRTX430	PF14541 P	PTHR1368	EC:3.4.23.	KOG1339			GO:00041
PAC:43539	SbiRTX430	SbiRTX430	SbiRTX430	PF04852	PTHR31165					
PAC:43543	SbiRTX430	SbiRTX430	SbiRTX430	PF00043 P	PTHR1126	EC:2.5.1.1	KOG0406	K00799		GO:000551
PAC:43540	SbiRTX430	SbiRTX430	SbiRTX430.03G46410		PTHR2331	EC:6.3.2.19				GO:00054
PAC:43542	SbiRTX430	SbiRTX430	SbiRTX430	PF01453	PTHR32444	PTHR32444:SF14				
PAC:43540	SbiRTX430	SbiRTX430	SbiRTX430.03G46920		PTHR36073					

PAC:43541	SbiRTX430	SbiRTX430	SbiRTX430	PF00141	PTHR3123	EC:1.11.1.7		K00430	GO:00046
PAC:43539	SbiRTX430	SbiRTX430	SbiRTX430	PF05512	PTHR33294	PTHR33294:SF4			
PAC:43539	SbiRTX430	SbiRTX430	SbiRTX430	PF03106	PTHR31221	PTHR31221:SF42			GO:00037
PAC:43543	SbiRTX430	SbiRTX430	SbiRTX430	PF00249	PTHR10641	PTHR10641:SF553			GO:000367
PAC:43565	SbiRTX430	SbiRTX430	SbiRTX430.04G00080		PTHR33527	PTHR33527:SF3			
PAC:43566	SbiRTX430	SbiRTX430	SbiRTX430	PF01423	PTHR23338		KOG3172	K11088	GO:00063
PAC:43568	SbiRTX430	SbiRTX430	SbiRTX430.04G020900.1.p						
PAC:43569	SbiRTX430	SbiRTX430	SbiRTX430	PF13833	PTHR10891	PTHR10891:SF626			GO:00055
PAC:43568	SbiRTX430	SbiRTX430	SbiRTX430.04G02500		PTHR19308	PTHR193	KOG2761		GO:00082
PAC:43568	SbiRTX430	SbiRTX430	SbiRTX430.04G02500		PTHR19308	PTHR193	KOG2761		GO:00082
PAC:43565	SbiRTX430	SbiRTX430	SbiRTX430	PF01027	PTHR23291	PTHR232	KOG1629		
PAC:43569	SbiRTX430	SbiRTX430	SbiRTX430.04G03010		PTHR34283				
PAC:43566	SbiRTX430	SbiRTX430	SbiRTX430	PF00295	PTHR3137	EC:3.2.1.15			GO:00046
PAC:43565	SbiRTX430	SbiRTX430	SbiRTX430.04G03200		PTHR35511	PTHR35511:SF1			
PAC:43565	SbiRTX430	SbiRTX430	SbiRTX430.04G03200		PTHR35511				
PAC:43568	SbiRTX430	SbiRTX430	SbiRTX430.04G03620		PTHR12176	PTHR12176:SF29			
PAC:43568	SbiRTX430	SbiRTX430	SbiRTX430	PF00432	PTHR11764	PTHR11764:SF23			GO:000382
PAC:43568	SbiRTX430	SbiRTX430	SbiRTX430	PF00432	PTHR11764	PTHR11764:SF23			GO:000382
PAC:43568	SbiRTX430	SbiRTX430	SbiRTX430	PF00432	PTHR11764	PTHR11764:SF23			GO:000382
PAC:43567	SbiRTX430	SbiRTX430	SbiRTX430	PF02309 P	PTHR31384	PTHR31384:SF9			GO:000367
PAC:43567	SbiRTX430	SbiRTX430	SbiRTX430	PF02309 P	PTHR31384	PTHR31384:SF9			GO:000367
PAC:43567	SbiRTX430	SbiRTX430	SbiRTX430	PF00571	PTHR13780	PTHR1378	KOG1764		
PAC:43565	SbiRTX430	SbiRTX430	SbiRTX430	PF13639	PTHR14155	PTHR14155:SF152			GO:00055
PAC:43569	SbiRTX430	SbiRTX430	SbiRTX430	PF00004 P	PTHR11638	PTHR11638:SF86		K03695	GO:00055
PAC:43567	SbiRTX430	SbiRTX430	SbiRTX430.04G07330		PTHR31414	PTHR31414:SF7			
PAC:43567	SbiRTX430	SbiRTX430	SbiRTX430.04G07520		PTHR36387	PTHR36387:SF1			
PAC:43567	SbiRTX430	SbiRTX430	SbiRTX430	PF00005 P	PTHR2422	EC:3.6.3.44		K05658	GO:00055
PAC:43565	SbiRTX430	SbiRTX430	SbiRTX430	PF01428 P	PTHR10634	PTHR1063	KOG3173		GO:00036
PAC:43565	SbiRTX430	SbiRTX430	SbiRTX430	PF01428	PTHR10634	PTHR1063	KOG3173		GO:00082
PAC:43568	SbiRTX430	SbiRTX430	SbiRTX430	PF00011	PTHR11527	PTHR1152	KOG3591	K13993	
PAC:43569	SbiRTX430	SbiRTX430	SbiRTX430	PF00656	PTHR31810	PTHR3181	KOG1546		GO:00041
PAC:43569	SbiRTX430	SbiRTX430	SbiRTX430	PF00656	PTHR31810	PTHR3181	KOG1546		GO:00041
PAC:43567	SbiRTX430	SbiRTX430	SbiRTX430	PF08392 P	PTHR3156	EC:2.3.1.119	EC:2.3.1	K15397	GO:00038
PAC:43567	SbiRTX430	SbiRTX430	SbiRTX430	PF08392 P	PTHR3156	EC:2.3.1.119	EC:2.3.1	K15397	GO:00038
PAC:43565	SbiRTX430	SbiRTX430	SbiRTX430.04G09750		PTHR33207	PTHR33207:SF6			GO:000551
PAC:43565	SbiRTX430	SbiRTX430	SbiRTX430	PF00011	PTHR11527	PTHR11527:SF130		K13993	
PAC:43565	SbiRTX430	SbiRTX430	SbiRTX430	PF00011	PTHR11527	PTHR11527:SF130		K13993	
PAC:43569	SbiRTX430	SbiRTX430	SbiRTX430	PF00011	PTHR11527	PTHR11527:SF130		K13993	
PAC:43565	SbiRTX430	SbiRTX430	SbiRTX430	PF00069 P	PTHR2700	EC:2.7.11.	KOG1187		GO:00046
PAC:43566	SbiRTX430	SbiRTX430	SbiRTX430	PF00447	PTHR10015	PTHR10015:SF171		K09419	GO:00037
PAC:43567	SbiRTX430	SbiRTX430	SbiRTX430	PF00281 P	PTHR11994	PTHR1199	KOG0397	K02868	GO:00037
PAC:43568	SbiRTX430	SbiRTX430	SbiRTX430	PF13639	PTHR14155	PTHR14155:SF146			GO:000551
PAC:43567	SbiRTX430	SbiRTX430	SbiRTX430.04G11920		PTHR23241	PTHR2324	KOG4744		
PAC:43567	SbiRTX430	SbiRTX430	SbiRTX430.04G11920		PTHR23241	PTHR23241:SF54			

PAC:43564	SbiRTX430	SbiRTX430	SbiRTX430	PF10604	PTHR31213	PTHR31213:SF16		K14496	
PAC:43564	SbiRTX430	SbiRTX430	SbiRTX430	PF10604	PTHR31213	PTHR31213:SF16			
PAC:43564	SbiRTX430	SbiRTX430	SbiRTX430	PF10604	PTHR31213	PTHR31213:SF16		K14496	
PAC:43564	SbiRTX430	SbiRTX430	SbiRTX430	PF10604	PTHR31213	PTHR31213:SF16		K14496	
PAC:43564	SbiRTX430	SbiRTX430	SbiRTX430	PF10604	PTHR31213	PTHR31213:SF16			
PAC:43566	SbiRTX430	SbiRTX430	SbiRTX430.04G12210(		PTHR34967	PTHR34967:SF1			
PAC:43565	SbiRTX430	SbiRTX430	SbiRTX430	PF00067	PTHR2429	EC:1.14.13	KOG0156	K00487	GO:000449
PAC:43569	SbiRTX430	SbiRTX430	SbiRTX430	PF04927	PTHR31174	PTHR31174:SF6			
PAC:43566	SbiRTX430	SbiRTX430	SbiRTX430	PF00481	PTHR1383	EC:3.1.3.1(	KOG0698		GO:000382
PAC:43567	SbiRTX430	SbiRTX430	SbiRTX430	PF03810 P	PTHR10997	PTHR10997:SF35			GO:000548
PAC:43569	SbiRTX430	SbiRTX430	SbiRTX430.04G16410(		PTHR35280				
PAC:43569	SbiRTX430	SbiRTX430	SbiRTX430.04G16410(		PTHR35280				
PAC:43568	SbiRTX430	SbiRTX430	SbiRTX430	PF03009	PTHR2295	EC:3.1.4.4(	KOG2421		GO:000662
PAC:43568	SbiRTX430	SbiRTX430	SbiRTX430	PF03009	PTHR2295	EC:3.1.4.46			GO:000662
PAC:43568	SbiRTX430	SbiRTX430	SbiRTX430	PF03009	PTHR2295	EC:3.1.4.4(	KOG2421		GO:000662
PAC:43569	SbiRTX430	SbiRTX430	SbiRTX430	PF13920	PTHR10044	PTHR1004	KOG1100	K19042	
PAC:43568	SbiRTX430	SbiRTX430	SbiRTX430	PF00128 P	PTHR1035	EC:2.4.1.18		K00700	GO:000382
PAC:43568	SbiRTX430	SbiRTX430	SbiRTX430	PF00533 P	PTHR1045	EC:2.4.2.3(	KOG1037	K10798	GO:000395
PAC:43568	SbiRTX430	SbiRTX430	SbiRTX430	PF00533 P	PTHR1045	EC:2.4.2.3(	KOG1037		GO:000395
PAC:43565	SbiRTX430	SbiRTX430	SbiRTX430	PF04043	PTHR35357	PTHR35357:SF2			GO:000485
PAC:43565	SbiRTX430	SbiRTX430	SbiRTX430	PF04043	PTHR35357	PTHR35357:SF3			GO:000485
PAC:43565	SbiRTX430	SbiRTX430	SbiRTX430	PF00657	PTHR2283	EC:3.1.1.1			GO:001678
PAC:43565	SbiRTX430	SbiRTX430	SbiRTX430	PF00657	PTHR2283	EC:3.1.1.1			GO:001678
PAC:43568	SbiRTX430	SbiRTX430	SbiRTX430	PF01842	PTHR3109	EC:2.7.7.59			GO:000815
PAC:43568	SbiRTX430	SbiRTX430	SbiRTX430	PF01842	PTHR3109	EC:2.7.7.59			GO:000815
PAC:43568	SbiRTX430	SbiRTX430	SbiRTX430	PF01842	PTHR3109	EC:2.7.7.59			GO:000815
PAC:43566	SbiRTX430	SbiRTX430	SbiRTX430.04G19010(		PTHR33646				
PAC:43565	SbiRTX430	SbiRTX430	SbiRTX430	PF00656	PTHR31810	PTHR3181	KOG1546		GO:000419
PAC:43566	SbiRTX430	SbiRTX430	SbiRTX430	PF00083	PTHR23500	PTHR23500:SF173			GO:001602
PAC:43566	SbiRTX430	SbiRTX430	SbiRTX430	PF00083	PTHR23500	PTHR23500:SF173			GO:001602
PAC:43566	SbiRTX430	SbiRTX430	SbiRTX430	PF00083	PTHR23500	PTHR23500:SF173			GO:001602
PAC:43566	SbiRTX430	SbiRTX430	SbiRTX430	PF00083	PTHR23500	PTHR23500:SF173			GO:001602
PAC:43566	SbiRTX430	SbiRTX430	SbiRTX430	PF00083	PTHR23500	PTHR23500:SF173			GO:001602
PAC:43568	SbiRTX430	SbiRTX430	SbiRTX430	PF00956	PTHR11875	PTHR1187	KOG1508	K11290	GO:000563
PAC:43565	SbiRTX430	SbiRTX430	SbiRTX430	PF00854	PTHR11654	PTHR11654:SF154			GO:000521
PAC:43565	SbiRTX430	SbiRTX430	SbiRTX430	PF00854	PTHR1165	EC:3.6.3.26			GO:000521
PAC:43565	SbiRTX430	SbiRTX430	SbiRTX430.04G20530(		PTHR22814	PTHR22814:SF122			
PAC:43567	SbiRTX430	SbiRTX430	SbiRTX430	PF04927	PTHR31174	PTHR31174:SF5			
PAC:43565	SbiRTX430	SbiRTX430	SbiRTX430	PF04927	PTHR31174	PTHR31174:SF5			
PAC:43566	SbiRTX430	SbiRTX430	SbiRTX430	PF03009	PTHR2334	EC:3.1.4.4(	KOG2258		GO:000662
PAC:43566	SbiRTX430	SbiRTX430	SbiRTX430	PF03009	PTHR2334	EC:3.1.4.4(	KOG2258		GO:000662
PAC:43568	SbiRTX430	SbiRTX430	SbiRTX430	PF03879	PTHR13557				GO:000370
PAC:43566	SbiRTX430	SbiRTX430	SbiRTX430	PF00069	PTHR2435	EC:2.7.11.	KOG0610		GO:000467
PAC:43567	SbiRTX430	SbiRTX430	SbiRTX430	PF03151	PTHR11132	PTHR1113	KOG1444	K15281	

PAC:43567	SbiRTX430	SbiRTX430	SbiRTX430	PF00076	PTHR24012				GO:000016
PAC:43566	SbiRTX430	SbiRTX430	SbiRTX430	PF00221	PTHR1036	EC:4.3.1.24		K10775	GO:000382
PAC:43568	SbiRTX430	SbiRTX430	SbiRTX430	PF03151	PTHR11132	PTHR1113	KOG1441	K15285	
PAC:43565	SbiRTX430	SbiRTX430	SbiRTX430.04G23920		PTHR24006				
PAC:43565	SbiRTX430	SbiRTX430	SbiRTX430.04G23920		PTHR24006				
PAC:43565	SbiRTX430	SbiRTX430	SbiRTX430.04G23920		PTHR24006				
PAC:43565	SbiRTX430	SbiRTX430	SbiRTX430.04G23920		PTHR24006				
PAC:43565	SbiRTX430	SbiRTX430	SbiRTX430.04G23920		PTHR24006				
PAC:43567	SbiRTX430	SbiRTX430	SbiRTX430	PF01477	PTHR31718	PTHR31718:SF4			GO:000551
PAC:43569	SbiRTX430	SbiRTX430	SbiRTX430	PF00462	PTHR10168	PTHR1016	KOG1752	K17479	GO:000905
PAC:43566	SbiRTX430	SbiRTX430	SbiRTX430	PF01357 P	PTHR31867	PTHR31867:SF26			GO:000557
PAC:43568	SbiRTX430	SbiRTX430	SbiRTX430.04G253900.1.p						
PAC:43568	SbiRTX430	SbiRTX430	SbiRTX430	PF05183	PTHR2307	EC:2.7.7.48		K11699	GO:000016
PAC:43568	SbiRTX430	SbiRTX430	SbiRTX430	PF05183	PTHR2307	EC:2.7.7.48		K11699	GO:000016
PAC:43568	SbiRTX430	SbiRTX430	SbiRTX430	PF05183	PTHR2307	EC:2.7.7.48		K11699	GO:000016
PAC:43568	SbiRTX430	SbiRTX430	SbiRTX430	PF05183	PTHR2307	EC:2.7.7.48		K11699	GO:000016
PAC:43568	SbiRTX430	SbiRTX430	SbiRTX430	PF05183	PTHR2307	EC:2.7.7.48		K11699	GO:000016
PAC:43568	SbiRTX430	SbiRTX430	SbiRTX430	PF05183	PTHR2307	EC:2.7.7.48			GO:000396
PAC:43567	SbiRTX430	SbiRTX430	SbiRTX430	PF00249	PTHR10641	PTHR10641:SF575		K09422	GO:000367
PAC:43566	SbiRTX430	SbiRTX430	SbiRTX430	PF00071	PTHR11711	PTHR1171	KOG0075	K07955	GO:000552
PAC:43565	SbiRTX430	SbiRTX430	SbiRTX430	PF02701	PTHR31992				GO:000367
PAC:43568	SbiRTX430	SbiRTX430	SbiRTX430	PF16529	PTHR15598	PTHR15598:SF6		K12616	GO:000551
PAC:43566	SbiRTX430	SbiRTX430	SbiRTX430	PF16544	PTHR11208		KOG1588	K14945	GO:000367
PAC:43568	SbiRTX430	SbiRTX430	SbiRTX430	PF04720	PTHR31579	PTHR31579:SF15			
PAC:43568	SbiRTX430	SbiRTX430	SbiRTX430	PF00307	PTHR19961	PTHR19961:SF25			GO:000551
PAC:43568	SbiRTX430	SbiRTX430	SbiRTX430	PF00307	PTHR19961	PTHR19961:SF25			GO:000551
PAC:43568	SbiRTX430	SbiRTX430	SbiRTX430	PF00307	PTHR19961	PTHR19961:SF25			GO:000551
PAC:43569	SbiRTX430	SbiRTX430	SbiRTX430.04G271200.1.p						
PAC:43569	SbiRTX430	SbiRTX430	SbiRTX430.04G271200.2.p						
PAC:43567	SbiRTX430	SbiRTX430	SbiRTX430	PF04707	PTHR11158	PTHR1115	KOG3336		GO:001649
PAC:43565	SbiRTX430	SbiRTX430	SbiRTX430	PF00487 P	PTHR32100	PTHR32100:SF13		K10256	GO:000662
PAC:43564	SbiRTX430	SbiRTX430	SbiRTX430	PF00487 P	PTHR32100	PTHR32100:SF13		K10256	GO:000662
PAC:43564	SbiRTX430	SbiRTX430	SbiRTX430	PF00487 P	PTHR32100	PTHR32100:SF13		K10256	GO:000662
PAC:43566	SbiRTX430	SbiRTX430	SbiRTX430	PF00011	PTHR11527	PTHR11527:SF144		K13993	
PAC:43566	SbiRTX430	SbiRTX430	SbiRTX430	PF00389 P	PTHR1099	EC:1.2.1.2	KOG0069	K00122	GO:000461
PAC:43564	SbiRTX430	SbiRTX430	SbiRTX430	PF08381 P	PTHR22870				
PAC:43564	SbiRTX430	SbiRTX430	SbiRTX430	PF08381	PTHR22870				
PAC:43568	SbiRTX430	SbiRTX430	SbiRTX430	PF00324	PTHR11785	PTHR1178	KOG1287		GO:000333
PAC:43568	SbiRTX430	SbiRTX430	SbiRTX430	PF00324	PTHR11785	PTHR1178	KOG1287		GO:000333
PAC:43568	SbiRTX430	SbiRTX430	SbiRTX430	PF00324	PTHR11785	PTHR1178	KOG1287		GO:000333
PAC:43567	SbiRTX430	SbiRTX430	SbiRTX430	PF01786	PTHR3180	EC:1.10.3.11		K17893	GO:000991
PAC:43567	SbiRTX430	SbiRTX430	SbiRTX430	PF01786	PTHR3180	EC:1.10.3.11			GO:000991
PAC:43566	SbiRTX430	SbiRTX430	SbiRTX430.04G28790		PTHR34197	PTHR34197:SF1			
PAC:43569	SbiRTX430	SbiRTX430	SbiRTX430	PF16913	PTHR31376	PTHR3137	KOG3912		GO:000521

PAC:43568	SbiRTX430	SbiRTX430	SbiRTX430	PF13639	PTHR14155	PTHR1415	KOG1493		GO:000551
PAC:43569	SbiRTX430	SbiRTX430	SbiRTX430.04G29440		PTHR13734	PTHR13734:SF42			
PAC:43569	SbiRTX430	SbiRTX430	SbiRTX430.04G29440		PTHR13734	PTHR13734:SF42			
PAC:43569	SbiRTX430	SbiRTX430	SbiRTX430.04G29440		PTHR13734	PTHR13734:SF42			
PAC:43569	SbiRTX430	SbiRTX430	SbiRTX430	PF03514	PTHR31636	PTHR31636:SF29			
PAC:43568	SbiRTX430	SbiRTX430	SbiRTX430	PF06884	PTHR31360	PTHR31360:SF0			
PAC:43569	SbiRTX430	SbiRTX430	SbiRTX430	PF04844	PTHR33057	PTHR33057:SF7			
PAC:43569	SbiRTX430	SbiRTX430	SbiRTX430	PF14299	PTHR31960	PTHR31960:SF8			GO:000551
PAC:43567	SbiRTX430	SbiRTX430	SbiRTX430	PF03478	PTHR13710	PTHR13710:SF88			GO:000551
PAC:43564	SbiRTX430	SbiRTX430	SbiRTX430	PF00657	PTHR2283	EC:3.1.1.3			GO:001678
PAC:43566	SbiRTX430	SbiRTX430	SbiRTX430	PF13716	PTHR11106	PTHR1110	KOG2633		
PAC:43566	SbiRTX430	SbiRTX430	SbiRTX430	PF00230	PTHR19139	PTHR1913	KOG0223	K09872	GO:000521
PAC:43566	SbiRTX430	SbiRTX430	SbiRTX430	PF00230	PTHR19139	PTHR1913	KOG0223	K09872	GO:000521
PAC:43566	SbiRTX430	SbiRTX430	SbiRTX430	PF13912	PTHR26374	PTHR26374:SF175			GO:000367
PAC:43567	SbiRTX430	SbiRTX430	SbiRTX430	PF01357 P	PTHR31692	PTHR31692:SF8			GO:000557
PAC:43568	SbiRTX430	SbiRTX430	SbiRTX430	PF01357 P	PTHR31692	PTHR31692:SF3			GO:000557
PAC:43566	SbiRTX430	SbiRTX430	SbiRTX430	PF05703 P	PTHR31351	PTHR31351:SF2			
PAC:43565	SbiRTX430	SbiRTX430	SbiRTX430	PF00847	PTHR31985	PTHR31985:SF24			GO:000367
PAC:43565	SbiRTX430	SbiRTX430	SbiRTX430	PF00847	PTHR31985	PTHR31985:SF3			GO:000367
PAC:43567	SbiRTX430	SbiRTX430	SbiRTX430.04G315200.1.p						
PAC:43569	SbiRTX430	SbiRTX430	SbiRTX430	PF13639	PTHR14155	PTHR14155:SF74			GO:000551
PAC:43566	SbiRTX430	SbiRTX430	SbiRTX430	PF01650	PTHR1200	EC:3.4.22.34		K01369	GO:000382
PAC:43568	SbiRTX430	SbiRTX430	SbiRTX430	PF13561	PTHR2432	EC:1.1.1.2	KOG1208	K15095	GO:000815
PAC:43565	SbiRTX430	SbiRTX430	SbiRTX430	PF03171 P	PTHR1020	EC:1.14.11	KOG0143	K05278	GO:000550
PAC:43569	SbiRTX430	SbiRTX430	SbiRTX430	PF07800	PTHR31197	PTHR31197:SF12			GO:000367
PAC:43569	SbiRTX430	SbiRTX430	SbiRTX430	PF07800	PTHR31197	PTHR31197:SF12			GO:000367
PAC:43568	SbiRTX430	SbiRTX430	SbiRTX430	PF00332 P	PTHR3222	EC:3.2.1.39			GO:000455
PAC:43568	SbiRTX430	SbiRTX430	SbiRTX430	PF00332 P	PTHR3222	EC:3.2.1.39			GO:000455
PAC:43568	SbiRTX430	SbiRTX430	SbiRTX430.04G333500.1.p						GO:000367
PAC:43566	SbiRTX430	SbiRTX430	SbiRTX430.04G337900.1.p						
PAC:43566	SbiRTX430	SbiRTX430	SbiRTX430.04G337900.2.p						
PAC:43567	SbiRTX430	SbiRTX430	SbiRTX430	PF00011	PTHR11527	PTHR11527:SF106		K13993	
PAC:43566	SbiRTX430	SbiRTX430	SbiRTX430	PF03435 P	PTHR1113	EC:1.5.1.9	KOG0172	K14157	GO:001649
PAC:43566	SbiRTX430	SbiRTX430	SbiRTX430	PF03435 P	PTHR1113	EC:1.5.1.9	KOG0172		GO:001649
PAC:43566	SbiRTX430	SbiRTX430	SbiRTX430	PF03435 P	PTHR1113	EC:1.5.1.9	KOG0172		GO:001649
PAC:43566	SbiRTX430	SbiRTX430	SbiRTX430	PF03435 P	PTHR1113	EC:1.5.1.9	KOG0172	K14157	GO:001649
PAC:43566	SbiRTX430	SbiRTX430	SbiRTX430	PF03435 P	PTHR1113	EC:1.5.1.9	KOG0172	K14157	GO:001649
PAC:43566	SbiRTX430	SbiRTX430	SbiRTX430	PF00188	PTHR10334	PTHR10334:SF207			
PAC:43569	SbiRTX430	SbiRTX430	SbiRTX430	PF01073	PTHR1036	EC:5.1.3.6	KOG1371	K08679	GO:000385
PAC:43569	SbiRTX430	SbiRTX430	SbiRTX430	PF01073	PTHR1036	EC:5.1.3.6	KOG1371	K08679	GO:000385
PAC:43569	SbiRTX430	SbiRTX430	SbiRTX430	PF01073	PTHR1036	EC:5.1.3.6	KOG1371	K08679	GO:000385
PAC:43564	SbiRTX430	SbiRTX430	SbiRTX430	PF00293 P	PTHR2342	EC:5.3.3.2	KOG0142		GO:001678
PAC:43564	SbiRTX430	SbiRTX430	SbiRTX430	PF04857	PTHR1079	EC:3.1.13.	KOG0304	K12581	GO:000367
PAC:43568	SbiRTX430	SbiRTX430	SbiRTX430	PF14299	PTHR32278	PTHR32278:SF2			GO:000551

PAC:43569	SbiRTX430	SbiRTX430	SbiRTX430	PF05903	PTHR12378	PTHR1237	KOG0324		
PAC:43565	SbiRTX430	SbiRTX430	SbiRTX430	PF00571	PTHR11911	PTHR11911:SF51			
PAC:43565	SbiRTX430	SbiRTX430	SbiRTX430	PF00571	PTHR11911	PTHR11911:SF51			
PAC:43569	SbiRTX430	SbiRTX430	SbiRTX430	PF00571	PTHR11911	PTHR11911:SF51			
PAC:43565	SbiRTX430	SbiRTX430	SbiRTX430	PF01554	PTHR11206	PTHR1120	KOG1347	K03327	GO:000685
PAC:43569	SbiRTX430	SbiRTX430	SbiRTX430	PF03600	PTHR10283	PTHR1028	KOG2639		GO:001602
PAC:43569	SbiRTX430	SbiRTX430	SbiRTX430	PF03600	PTHR10283	PTHR1028	KOG2639		GO:001602
PAC:43564	SbiRTX430	SbiRTX430	SbiRTX430.04G37340		PTHR37391	PTHR37391:SF1			GO:000551
PAC:43575	SbiRTX430	SbiRTX430	SbiRTX430	PF00487 P	PTHR32100	PTHR32100:SF14		K10257	GO:000662
PAC:43574	SbiRTX430	SbiRTX430	SbiRTX430.05G00850		PTHR35740				
PAC:43575	SbiRTX430	SbiRTX430	SbiRTX430	PF03000	PTHR32370	PTHR32370:SF17			GO:000487
PAC:43575	SbiRTX430	SbiRTX430	SbiRTX430	PF03000	PTHR32370	PTHR32370:SF17			GO:000487
PAC:43575	SbiRTX430	SbiRTX430	SbiRTX430	PF03000	PTHR32370	PTHR32370:SF17			GO:000487
PAC:43575	SbiRTX430	SbiRTX430	SbiRTX430	PF03000	PTHR32370	PTHR32370:SF17			GO:000487
PAC:43575	SbiRTX430	SbiRTX430	SbiRTX430	PF03000	PTHR32370	PTHR32370:SF17			GO:000487
PAC:43575	SbiRTX430	SbiRTX430	SbiRTX430	PF03000	PTHR32370	PTHR32370:SF17			GO:000487
PAC:43575	SbiRTX430	SbiRTX430	SbiRTX430	PF03000	PTHR32370	PTHR32370:SF17			GO:000487
PAC:43575	SbiRTX430	SbiRTX430	SbiRTX430	PF03000	PTHR32370	PTHR32370:SF17			GO:000487
PAC:43575	SbiRTX430	SbiRTX430	SbiRTX430	PF03000	PTHR32370	PTHR32370:SF17			GO:000487
PAC:43574	SbiRTX430	SbiRTX430	SbiRTX430	PF00847	PTHR32467	PTHR32467:SF21		K09285	GO:000367
PAC:43575	SbiRTX430	SbiRTX430	SbiRTX430	PF00122 P	PTHR2409	EC:3.6.3.8		K01537	GO:000016
PAC:43575	SbiRTX430	SbiRTX430	SbiRTX430	PF00122 P	PTHR2409	EC:3.6.3.8			GO:000016
PAC:43575	SbiRTX430	SbiRTX430	SbiRTX430	PF13537	PTHR11772	PTHR11772:SF18			
PAC:43575	SbiRTX430	SbiRTX430	SbiRTX430	PF06775	PTHR21212	PTHR21212:SF0			
PAC:43576	SbiRTX430	SbiRTX430	SbiRTX430	PF07690	PTHR23504	PTHR2350	KOG2816		GO:001602
PAC:43575	SbiRTX430	SbiRTX430	SbiRTX430	PF02496	PTHR33801	PTHR33801:SF7			GO:000695
PAC:43575	SbiRTX430	SbiRTX430	SbiRTX430	PF13561	PTHR2432	EC:1.1.1.2	KOG0725	K08081	GO:000815
PAC:43575	SbiRTX430	SbiRTX430	SbiRTX430	PF13561	PTHR2432	EC:1.1.1.2	KOG0725		GO:000815
PAC:43574	SbiRTX430	SbiRTX430	SbiRTX430	PF01490	PTHR22950	PTHR2295	KOG1303		
PAC:43576	SbiRTX430	SbiRTX430	SbiRTX430.05G07180		PTHR32054	PTHR32054:SF11			
PAC:43574	SbiRTX430	SbiRTX430	SbiRTX430	PF00011	PTHR11527	PTHR1152	KOG0710	K13993	
PAC:43574	SbiRTX430	SbiRTX430	SbiRTX430	PF00432	PTHR1176	EC:5.4.99.47		K19010	GO:000382
PAC:43574	SbiRTX430	SbiRTX430	SbiRTX430	PF00931	PTHR2315	EC:2.3.1.156		K13457	GO:004353
PAC:43574	SbiRTX430	SbiRTX430	SbiRTX430	PF00931	PTHR23155	PTHR2315	KOG4658	K13457	GO:004353
PAC:43574	SbiRTX430	SbiRTX430	SbiRTX430	PF00107 P	PTHR1169	EC:1.1.1.1			GO:000827
PAC:43575	SbiRTX430	SbiRTX430	SbiRTX430	PF02179	PTHR33322	PTHR33322:SF1			GO:000551
PAC:43576	SbiRTX430	SbiRTX430	SbiRTX430	PF01397 P	PTHR3122	EC:4.2.3.104		K15803	GO:000028
PAC:43576	SbiRTX430	SbiRTX430	SbiRTX430	PF01397 P	PTHR3122	EC:4.2.3.104		K15803	GO:000028
PAC:43576	SbiRTX430	SbiRTX430	SbiRTX430	PF01397	PTHR3122	EC:4.2.3.123			GO:000815
PAC:43576	SbiRTX430	SbiRTX430	SbiRTX430	PF01397 P	PTHR3122	EC:4.2.3.104		K15803	GO:000028
PAC:43575	SbiRTX430	SbiRTX430	SbiRTX430	PF01074 P	PTHR1160	EC:3.2.1.24		K01191	GO:000382
PAC:43574	SbiRTX430	SbiRTX430	SbiRTX430.05G15320		PTHR33919	PTHR33919:SF2			
PAC:43575	SbiRTX430	SbiRTX430	SbiRTX430	PF13920	PTHR10044	PTHR1004	KOG1100	K19042	GO:000551
PAC:43574	SbiRTX430	SbiRTX430	SbiRTX430	PF00244	PTHR18860	PTHR1886	KOG0841	K06630	GO:001990

PAC:43574	SbiRTX430	SbiRTX430	SbiRTX430	PF00244	PTHR18860	PTHR1886	KOG0841	K06630	GO:001990
PAC:43576	SbiRTX430	SbiRTX430	SbiRTX430.05G160100.1.p						
PAC:43575	SbiRTX430	SbiRTX430	SbiRTX430	PF06404	PTHR33285	PTHR33285:SF1			GO:000557
PAC:43573	SbiRTX430	SbiRTX430	SbiRTX430	PF06886	PTHR31358	PTHR31358:SF1			
PAC:43576	SbiRTX430	SbiRTX430	SbiRTX430	PF01027	PTHR23291	PTHR2329	KOG2322	K06890	
PAC:43576	SbiRTX430	SbiRTX430	SbiRTX430	PF04185	PTHR3195	EC:3.1.4.3		K01114	GO:000382
PAC:43573	SbiRTX430	SbiRTX430	SbiRTX430	PF01734	PTHR32176	PTHR3217	KOG0513		GO:000662
PAC:43573	SbiRTX430	SbiRTX430	SbiRTX430	PF01734	PTHR32176	PTHR3217	KOG0513		GO:000662
PAC:43573	SbiRTX430	SbiRTX430	SbiRTX430	PF01734	PTHR3217	EC:3.1.1.26			GO:000662
PAC:43576	SbiRTX430	SbiRTX430	SbiRTX430	PF01734	PTHR32176	PTHR3217	KOG0513		GO:000662
PAC:43574	SbiRTX430	SbiRTX430	SbiRTX430	PF02234	PTHR10265	PTHR10265:SF27			GO:000486
PAC:43575	SbiRTX430	SbiRTX430	SbiRTX430	PF01559					GO:004573
PAC:43575	SbiRTX430	SbiRTX430	SbiRTX430	PF01559					GO:004573
PAC:43576	SbiRTX430	SbiRTX430	SbiRTX430	PF00717	PTHR1238	EC:3.4.21.	KOG0171	K09647	GO:00065
PAC:43576	SbiRTX430	SbiRTX430	SbiRTX430	PF00717	PTHR1238	EC:3.4.21.	KOG0171	K09647	GO:00065
PAC:43573	SbiRTX430	SbiRTX430	SbiRTX430	PF00076	PTHR24012	PTHR2401	KOG0148	K13201	GO:00001
PAC:43574	SbiRTX430	SbiRTX430	SbiRTX430.05G200800.1.p						
PAC:43574	SbiRTX430	SbiRTX430	SbiRTX430	PF00083 P	PTHR23500	PTHR235	KOG0254		GO:001602
PAC:43574	SbiRTX430	SbiRTX430	SbiRTX430	PF00083 P	PTHR23500	PTHR235	KOG0254		GO:001602
PAC:43574	SbiRTX430	SbiRTX430	SbiRTX430	PF00083 P	PTHR23500	PTHR235	KOG0254		GO:001602
PAC:43576	SbiRTX430	SbiRTX430	SbiRTX430.05G20150		PTHR11926	PTHR11926:SF341			GO:000815
PAC:43575	SbiRTX430	SbiRTX430	SbiRTX430	PF01559					GO:004573
PAC:43575	SbiRTX430	SbiRTX430	SbiRTX430	PF01559					GO:004573
PAC:43574	SbiRTX430	SbiRTX430	SbiRTX430	PF01559					GO:004573
PAC:43574	SbiRTX430	SbiRTX430	SbiRTX430	PF01559					GO:000815
PAC:43575	SbiRTX430	SbiRTX430	SbiRTX430	PF01559					GO:004573
PAC:43576	SbiRTX430	SbiRTX430	SbiRTX430	PF01559					GO:004573
PAC:43575	SbiRTX430	SbiRTX430	SbiRTX430	PF07173	PTHR34365	PTHR34365:SF2			GO:00055
PAC:43575	SbiRTX430	SbiRTX430	SbiRTX430	PF07173	PTHR34365	PTHR34365:SF2			GO:00055
PAC:43575	SbiRTX430	SbiRTX430	SbiRTX430	PF07173	PTHR34365	PTHR34365:SF2			GO:00055
PAC:43575	SbiRTX430	SbiRTX430	SbiRTX430	PF07173	PTHR34365	PTHR34365:SF2			GO:00055
PAC:43575	SbiRTX430	SbiRTX430	SbiRTX430	PF07173	PTHR34365	PTHR34365:SF2			GO:00055
PAC:43575	SbiRTX430	SbiRTX430	SbiRTX430	PF07173	PTHR34365	PTHR34365:SF2			GO:00055
PAC:43575	SbiRTX430	SbiRTX430	SbiRTX430	PF07173	PTHR34365	PTHR34365:SF2			GO:00055
PAC:43575	SbiRTX430	SbiRTX430	SbiRTX430.05G20510		PTHR33167	PTHR33167:SF12			
PAC:43574	SbiRTX430	SbiRTX430	SbiRTX430	PF11250	PTHR33155	PTHR33155:SF7			
PAC:43575	SbiRTX430	SbiRTX430	SbiRTX430	PF04665	PTHR23155	PTHR2315	KOG4658		
PAC:43575	SbiRTX430	SbiRTX430	SbiRTX430	PF04665	PTHR23155	PTHR2315	KOG4658		
PAC:43574	SbiRTX430	SbiRTX430	SbiRTX430	PF03018	PTHR21495	PTHR21495:SF81			
PAC:43576	SbiRTX430	SbiRTX430	SbiRTX430	PF04578 P	PTHR31325	PTHR31325:SF23			
PAC:43576	SbiRTX430	SbiRTX430	SbiRTX430	PF00931	PTHR23155	PTHR2315	KOG4658		GO:004353
PAC:43576	SbiRTX430	SbiRTX430	SbiRTX430	PF00931	PTHR23155	PTHR2315	KOG4658		GO:004353
PAC:43576	SbiRTX430	SbiRTX430	SbiRTX430	PF00931	PTHR23155	PTHR2315	KOG4658		GO:004353
PAC:43576	SbiRTX430	SbiRTX430	SbiRTX430.05G23680		PTHR23155	PTHR23155:SF484			

PAC:43574	SbiRTX430	SbiRTX430	SbiRTX430	PF00069 P	PTHR2700:	EC:2.7.11.1	KOG1187		GO:000467
PAC:43551	SbiRTX430	SbiRTX430	SbiRTX430	PF00067	PTHR24298	PTHR2429	KOG0156		GO:00044
PAC:43552	SbiRTX430	SbiRTX430	SbiRTX430	PF00183 P	PTHR11528	PTHR11528:SF53		K04079	GO:00055
PAC:43552	SbiRTX430	SbiRTX430	SbiRTX430	PF00183 P	PTHR11528	PTHR11528:SF53			GO:00055
PAC:43551	SbiRTX430	SbiRTX430	SbiRTX430	PF00092 P	PTHR10579	PTHR10579:SF64			
PAC:43550	SbiRTX430	SbiRTX430	SbiRTX430	PF07676	PTHR32161	PTHR32161:SF6			
PAC:43549	SbiRTX430	SbiRTX430	SbiRTX430	PF00314	PTHR31048	PTHR31048:SF33			
PAC:43551	SbiRTX430	SbiRTX430	SbiRTX430	PF00069 P	PTHR2700:	EC:2.7.11.1	KOG1187		GO:000467
PAC:43551	SbiRTX430	SbiRTX430	SbiRTX430.06G037200.1.p						
PAC:43551	SbiRTX430	SbiRTX430	SbiRTX430.06G037200.2.p						
PAC:43551	SbiRTX430	SbiRTX430	SbiRTX430.06G037200.3.p						
PAC:43552	SbiRTX430	SbiRTX430	SbiRTX430.06G05550		PTHR33829	PTHR33829:SF2			
PAC:43551	SbiRTX430	SbiRTX430	SbiRTX430	PF00676 P	PTHR2315	EC:1.2.4.2		K00164	GO:00045
PAC:43551	SbiRTX430	SbiRTX430	SbiRTX430	PF00676 P	PTHR2315	EC:1.2.4.2		K00164	GO:00045
PAC:43551	SbiRTX430	SbiRTX430	SbiRTX430	PF00676 P	PTHR2315	EC:1.2.4.2		K00164	GO:00045
PAC:43551	SbiRTX430	SbiRTX430	SbiRTX430	PF00676 P	PTHR2315	EC:1.2.4.2		K00164	GO:00045
PAC:43550	SbiRTX430	SbiRTX430	SbiRTX430.06G057900.1.p						
PAC:43550	SbiRTX430	SbiRTX430	SbiRTX430.06G057900.2.p						
PAC:43549	SbiRTX430	SbiRTX430	SbiRTX430	PF08659	PTHR2432	EC:1.1.1.1	KOG1205		GO:00081
PAC:43550	SbiRTX430	SbiRTX430	SbiRTX430	PF13561	PTHR2432	EC:1.1.1.1	KOG1205		GO:00081
PAC:43552	SbiRTX430	SbiRTX430	SbiRTX430	PF00462	PTHR1016	EC:1.8.1.9	KOG1752	K03676	GO:00090
PAC:43552	SbiRTX430	SbiRTX430	SbiRTX430.06G061500.1.p						
PAC:43549	SbiRTX430	SbiRTX430	SbiRTX430	PF01565 P	PTHR3244	EC:1.3.3.8			GO:00038
PAC:43551	SbiRTX430	SbiRTX430	SbiRTX430.06G06590		PTHR22814	PTHR22814:SF135			
PAC:43551	SbiRTX430	SbiRTX430	SbiRTX430.06G06590		PTHR22814	PTHR22814:SF135			
PAC:43548	SbiRTX430	SbiRTX430	SbiRTX430.06G06620		PTHR22814	PTHR2281	KOG1603		GO:00300
PAC:43548	SbiRTX430	SbiRTX430	SbiRTX430.06G06620		PTHR22814	PTHR2281	KOG1603		
PAC:43550	SbiRTX430	SbiRTX430	SbiRTX430.06G06630		PTHR22814	PTHR22814:SF135			
PAC:43549	SbiRTX430	SbiRTX430	SbiRTX430	PF13668	PTHR31694				
PAC:43552	SbiRTX430	SbiRTX430	SbiRTX430	PF00149 P	PTHR2295	EC:3.1.3.2	KOG1378		GO:00039
PAC:43551	SbiRTX430	SbiRTX430	SbiRTX430.06G07840		PTHR33177	PTHR33177:SF3			
PAC:43551	SbiRTX430	SbiRTX430	SbiRTX430	PF02496	PTHR33801	PTHR33801:SF6			GO:000695
PAC:43551	SbiRTX430	SbiRTX430	SbiRTX430.06G083600.2.p						
PAC:43551	SbiRTX430	SbiRTX430	SbiRTX430.06G083600.3.p						
PAC:43551	SbiRTX430	SbiRTX430	SbiRTX430.06G08650		PTHR13593	PTHR13593:SF52			GO:00066
PAC:43551	SbiRTX430	SbiRTX430	SbiRTX430	PF01025	PTHR21237	PTHR2123	KOG3003		GO:00007
PAC:43550	SbiRTX430	SbiRTX430	SbiRTX430	PF13664	PTHR23241	PTHR2324	KOG2886		
PAC:43550	SbiRTX430	SbiRTX430	SbiRTX430	PF00724	PTHR2289	EC:1.3.1.4	KOG0134	K05894	GO:000382
PAC:43550	SbiRTX430	SbiRTX430	SbiRTX430	PF00724	PTHR2289	EC:1.3.1.4	KOG0134		GO:000382
PAC:43550	SbiRTX430	SbiRTX430	SbiRTX430	PF00724	PTHR2289	EC:1.3.1.4	KOG0134		GO:000382
PAC:43551	SbiRTX430	SbiRTX430	SbiRTX430	PF00011	PTHR11527	PTHR1152	KOG3591	K13993	
PAC:43552	SbiRTX430	SbiRTX430	SbiRTX430	PF04043	PTHR31080	PTHR31080:SF18			GO:00048
PAC:43550	SbiRTX430	SbiRTX430	SbiRTX430	PF04844	PTHR33057	PTHR33057:SF13			
PAC:43551	SbiRTX430	SbiRTX430	SbiRTX430	PF00076	PTHR24012	PTHR2401	KOG0148		GO:00001

PAC:43550	SbiRTX430	SbiRTX430	SbiRTX430	PF02298	PTHR33021	PTHR33021:SF38			GO:00090!
PAC:43551	SbiRTX430	SbiRTX430	SbiRTX430	PF01477	PTHR31718	PTHR31718:SF0			GO:000551
PAC:43549	SbiRTX430	SbiRTX430	SbiRTX430	PF04873	PTHR33305	PTHR33305:SF4		K14514	GO:000370
PAC:43549	SbiRTX430	SbiRTX430	SbiRTX430	PF04873	PTHR33305	PTHR33305:SF4			GO:000370
PAC:43550	SbiRTX430	SbiRTX430	SbiRTX430	PF01263	PTHR1009	EC:5.1.3.3	KOG1604	K01785	GO:000382
PAC:43551	SbiRTX430	SbiRTX430	SbiRTX430	PF00244	PTHR18860	PTHR1886	KOG0841	K06630	GO:001990
PAC:43551	SbiRTX430	SbiRTX430	SbiRTX430	PF00244	PTHR18860	PTHR1886	KOG0841	K06630	GO:001990
PAC:43551	SbiRTX430	SbiRTX430	SbiRTX430	PF00244	PTHR18860	PTHR1886	KOG0841	K06630	GO:001990
PAC:43552	SbiRTX430	SbiRTX430	SbiRTX430	PF01145	PTHR23222	PTHR2322	KOG3083	K17080	GO:001602
PAC:43550	SbiRTX430	SbiRTX430	SbiRTX430	PF14570	PTHR12603				GO:000551
PAC:43549	SbiRTX430	SbiRTX430	SbiRTX430	PF02704	PTHR23201	PTHR23201:SF18			
PAC:43549	SbiRTX430	SbiRTX430	SbiRTX430.06G11950		PTHR2281	EC:3.4.21.72			
PAC:43549	SbiRTX430	SbiRTX430	SbiRTX430.06G11950		PTHR2281	EC:3.4.21.72			
PAC:43549	SbiRTX430	SbiRTX430	SbiRTX430.06G11950		PTHR2281	EC:3.4.21.72			
PAC:43548	SbiRTX430	SbiRTX430	SbiRTX430	PF00150	PTHR3126	EC:3.2.1.4			GO:00045!
PAC:43548	SbiRTX430	SbiRTX430	SbiRTX430.06G128600.2.p						
PAC:43549	SbiRTX430	SbiRTX430	SbiRTX430	PF08241	PTHR1010	EC:2.1.1.41			GO:00081!
PAC:43548	SbiRTX430	SbiRTX430	SbiRTX430	PF00400	PTHR2284	EC:2.7.11.	KOG0313		GO:000551
PAC:43551	SbiRTX430	SbiRTX430	SbiRTX430	PF01625	PTHR1017	EC:1.8.4.1	KOG1635	K07304	GO:000697
PAC:43552	SbiRTX430	SbiRTX430	SbiRTX430	PF00201	PTHR11926	PTHR11926:SF399			GO:00081!
PAC:43550	SbiRTX430	SbiRTX430	SbiRTX430	PF00069	PTHR2435	EC:2.7.11.	KOG0610		GO:000467
PAC:43552	SbiRTX430	SbiRTX430	SbiRTX430.06G13870		PTHR16223	PTHR16223:SF44			GO:004698
PAC:43552	SbiRTX430	SbiRTX430	SbiRTX430.06G13870		PTHR16223	PTHR16223:SF44			GO:004698
PAC:43552	SbiRTX430	SbiRTX430	SbiRTX430.06G13870		PTHR16223	PTHR16223:SF44			GO:004698
PAC:43549	SbiRTX430	SbiRTX430	SbiRTX430	PF00182	PTHR2259	EC:3.2.1.1	KOG4742	K01183	GO:000456
PAC:43550	SbiRTX430	SbiRTX430	SbiRTX430	PF00182 P	PTHR2259	EC:3.2.1.1	KOG4742	K01183	GO:000456
PAC:43550	SbiRTX430	SbiRTX430	SbiRTX430	PF04862	PTHR31265	PTHR31265:SF11			
PAC:43552	SbiRTX430	SbiRTX430	SbiRTX430	PF00141	PTHR3123	EC:1.11.1.7		K00430	GO:000460
PAC:43551	SbiRTX430	SbiRTX430	SbiRTX430	PF03140	PTHR31549	PTHR31549:SF25			
PAC:43549	SbiRTX430	SbiRTX430	SbiRTX430	PF05042	PTHR3149	EC:1.11.2.3		K17991	
PAC:43549	SbiRTX430	SbiRTX430	SbiRTX430	PF05042	PTHR3149	EC:1.11.2.3			
PAC:43549	SbiRTX430	SbiRTX430	SbiRTX430	PF05042	PTHR3149	EC:1.11.2.3		K17991	
PAC:43552	SbiRTX430	SbiRTX430	SbiRTX430	PF05757	PTHR33399	PTHR33399:SF4			GO:000550
PAC:43549	SbiRTX430	SbiRTX430	SbiRTX430	PF00230	PTHR19139	PTHR1913	KOG0223	K09873	GO:000521
PAC:43549	SbiRTX430	SbiRTX430	SbiRTX430	PF00230	PTHR19139	PTHR1913	KOG0223		GO:000521
PAC:43550	SbiRTX430	SbiRTX430	SbiRTX430	PF00487 P	PTHR12879			K04712	GO:000662
PAC:43551	SbiRTX430	SbiRTX430	SbiRTX430	PF03188	PTHR1010	EC:1.16.5.	KOG1619	K08360	GO:001602
PAC:43551	SbiRTX430	SbiRTX430	SbiRTX430	PF00538	PTHR11467	PTHR1146	KOG4012		GO:000078
PAC:43549	SbiRTX430	SbiRTX430	SbiRTX430.06G175600.1.p						
PAC:43552	SbiRTX430	SbiRTX430	SbiRTX430	PF01277	PTHR33203	PTHR33203:SF7			GO:001251
PAC:43552	SbiRTX430	SbiRTX430	SbiRTX430	PF01277	PTHR33203	PTHR33203:SF7			GO:001251
PAC:43552	SbiRTX430	SbiRTX430	SbiRTX430	PF01277	PTHR33203	PTHR33203:SF7			GO:001251
PAC:43551	SbiRTX430	SbiRTX430	SbiRTX430	PF00847	PTHR31190	PTHR31190:SF29		K09286	GO:000367
PAC:43550	SbiRTX430	SbiRTX430	SbiRTX430	PF00847	PTHR31985	PTHR31985:SF24			GO:000367

PAC:43551	SbiRTX430	SbiRTX430	SbiRTX430	PF02892 P	PTHR23272	PTHR2327	KOG1121		GO:000367
PAC:43551	SbiRTX430	SbiRTX430	SbiRTX430	PF08613	PTHR15615	PTHR1561	KOG1674		GO:000007
PAC:43552	SbiRTX430	SbiRTX430	SbiRTX430	PF02460 P	PTHR10796	PTHR10796:SF117		K12385	GO:000815
PAC:43549	SbiRTX430	SbiRTX430	SbiRTX430	PF00447	PTHR10015	PTHR10015:SF203		K09419	GO:000370
PAC:43548	SbiRTX430	SbiRTX430	SbiRTX430	PF00612 P	PTHR32295	PTHR32295:SF11			GO:000551
PAC:43549	SbiRTX430	SbiRTX430	SbiRTX430.06G198500.1.p						
PAC:43550	SbiRTX430	SbiRTX430	SbiRTX430	PF13639	PTHR14155	PTHR14155:SF2			GO:000551
PAC:43549	SbiRTX430	SbiRTX430	SbiRTX430	PF00005 P	PTHR2422	EC:3.6.3.44		K05665	GO:000552
PAC:43549	SbiRTX430	SbiRTX430	SbiRTX430	PF00005 P	PTHR2422	EC:3.6.3.44		K05665	GO:000552
PAC:43549	SbiRTX430	SbiRTX430	SbiRTX430.06G21870		PTHR33972	PTHR33972:SF2			
PAC:43549	SbiRTX430	SbiRTX430	SbiRTX430	PF12708	PTHR3392	EC:3.2.1.15			
PAC:43549	SbiRTX430	SbiRTX430	SbiRTX430	PF12708	PTHR3392	EC:3.2.1.15			
PAC:43552	SbiRTX430	SbiRTX430	SbiRTX430	PF01344	PTHR24413	PTHR24413:SF123			GO:000551
PAC:43551	SbiRTX430	SbiRTX430	SbiRTX430	PF00005 P	PTHR2422	EC:3.6.3.44			GO:000552
PAC:43551	SbiRTX430	SbiRTX430	SbiRTX430	PF00005 P	PTHR2422	EC:3.6.3.44			GO:000552
PAC:43551	SbiRTX430	SbiRTX430	SbiRTX430	PF00005 P	PTHR2422	EC:3.6.3.44			GO:000552
PAC:43551	SbiRTX430	SbiRTX430	SbiRTX430	PF00005 P	PTHR2422	EC:3.6.3.44			GO:000552
PAC:43551	SbiRTX430	SbiRTX430	SbiRTX430	PF00005 P	PTHR2422	EC:3.6.3.44			GO:000552
PAC:43549	SbiRTX430	SbiRTX430	SbiRTX430	PF08212	PTHR1143	EC:1.14.13	KOG4824		
PAC:43549	SbiRTX430	SbiRTX430	SbiRTX430	PF08212	PTHR1143	EC:1.14.13	KOG4824		
PAC:43550	SbiRTX430	SbiRTX430	SbiRTX430	PF00722 P	PTHR3106	EC:2.4.1.207	EC:3.2.1	K08235	GO:000455
PAC:43552	SbiRTX430	SbiRTX430	SbiRTX430.06G24860		PTHR33109	PTHR33109:SF6			
PAC:43551	SbiRTX430	SbiRTX430	SbiRTX430	PF03171 P	PTHR1020	EC:1.14.11	KOG0143		GO:001649
PAC:43550	SbiRTX430	SbiRTX430	SbiRTX430	PF05678	PTHR33402				
PAC:43549	SbiRTX430	SbiRTX430	SbiRTX430.06G257600.1.p						
PAC:43551	SbiRTX430	SbiRTX430	SbiRTX430.06G257700.1.p						
PAC:43551	SbiRTX430	SbiRTX430	SbiRTX430	PF04669	PTHR31444	PTHR31444:SF3			
PAC:43550	SbiRTX430	SbiRTX430	SbiRTX430	PF02769 P	PTHR1009	EC:6.3.5.3		K01952	GO:000464
PAC:43548	SbiRTX430	SbiRTX430	SbiRTX430	PF00251 P	PTHR3195	EC:3.2.1.26		K01193	GO:000455
PAC:43548	SbiRTX430	SbiRTX430	SbiRTX430	PF00251 P	PTHR3195	EC:3.2.1.26			GO:000455
PAC:43550	SbiRTX430	SbiRTX430	SbiRTX430	PF00403	PTHR22814	PTHR2281	KOG1603		GO:003000
PAC:43550	SbiRTX430	SbiRTX430	SbiRTX430	PF00403	PTHR22814	PTHR2281	KOG1603		GO:003000
PAC:43549	SbiRTX430	SbiRTX430	SbiRTX430	PF00067	PTHR24298	PTHR2429	KOG0156		GO:000449
PAC:43551	SbiRTX430	SbiRTX430	SbiRTX430	PF00571	PTHR13780	PTHR13780:SF55			
PAC:43550	SbiRTX430	SbiRTX430	SbiRTX430.06G28750		PTHR36053	PTHR36053:SF1			
PAC:43550	SbiRTX430	SbiRTX430	SbiRTX430	PF04844	PTHR33057	PTHR33057:SF26			
PAC:43551	SbiRTX430	SbiRTX430	SbiRTX430	PF00141	PTHR3123	EC:1.11.1.7		K00430	GO:000460
PAC:43562	SbiRTX430	SbiRTX430	SbiRTX430	PF04535	PTHR11615	PTHR11615:SF116			
PAC:43564	SbiRTX430	SbiRTX430	SbiRTX430.07G00510		PTHR37732				
PAC:43561	SbiRTX430	SbiRTX430	SbiRTX430	PF01357 P	PTHR31867	PTHR31867:SF10			GO:000557
PAC:43562	SbiRTX430	SbiRTX430	SbiRTX430	PF00076 P	PTHR10352		KOG0122	K03248	GO:000016
PAC:43564	SbiRTX430	SbiRTX430	SbiRTX430	PF02847	PTHR12626	PTHR12626:SF0			GO:000524
PAC:43564	SbiRTX430	SbiRTX430	SbiRTX430	PF02847	PTHR12626	PTHR12626:SF0			GO:000524
PAC:43563	SbiRTX430	SbiRTX430	SbiRTX430	PF00044 P	PTHR1083	EC:1.2.1.1	KOG0657	K00134	GO:000600

PAC:43563	SbiRTX430	SbiRTX430	SbiRTX430	PF00044 P	PTHR1083(	EC:1.2.1.1:	KOG0657	K00134	GO:00060(
PAC:43564	SbiRTX430	SbiRTX430	SbiRTX430	PF03222	PTHR22950	PTHR2295	KOG1303		GO:00033:
PAC:43564	SbiRTX430	SbiRTX430	SbiRTX430	PF00400	PTHR22838	PTHR22838:SF3			GO:00055:
PAC:43564	SbiRTX430	SbiRTX430	SbiRTX430	PF00400	PTHR22838	PTHR22838:SF3			GO:00055:
PAC:43564	SbiRTX430	SbiRTX430	SbiRTX430	PF00400	PTHR22838	PTHR22838:SF3			GO:00055:
PAC:43564	SbiRTX430	SbiRTX430	SbiRTX430	PF07059	PTHR12136	PTHR12136:SF61			
PAC:43564	SbiRTX430	SbiRTX430	SbiRTX430	PF07059	PTHR12136	PTHR12136:SF61			
PAC:43564	SbiRTX430	SbiRTX430	SbiRTX430	PF07059	PTHR12136	PTHR12136:SF61			
PAC:43563	SbiRTX430	SbiRTX430	SbiRTX430	PF00249	PTHR12802	PTHR12802:SF43		K12133	GO:00036
PAC:43563	SbiRTX430	SbiRTX430	SbiRTX430	PF00249	PTHR12802	PTHR12802:SF43		K12133	GO:00036
PAC:43563	SbiRTX430	SbiRTX430	SbiRTX430	PF00249	PTHR12802	PTHR12802:SF43		K12133	GO:00036
PAC:43563	SbiRTX430	SbiRTX430	SbiRTX430	PF00249	PTHR12802	PTHR12802:SF43		K12133	GO:00036
PAC:43563	SbiRTX430	SbiRTX430	SbiRTX430	PF00249	PTHR12802	PTHR12802:SF43		K12133	GO:00036
PAC:43563	SbiRTX430	SbiRTX430	SbiRTX430	PF00249	PTHR12802	PTHR12802:SF43		K12133	GO:00036
PAC:43563	SbiRTX430	SbiRTX430	SbiRTX430	PF00249	PTHR12802	PTHR12802:SF43		K12133	GO:00036
PAC:43563	SbiRTX430	SbiRTX430	SbiRTX430	PF00249	PTHR12802	PTHR12802:SF43			GO:00036
PAC:43563	SbiRTX430	SbiRTX430	SbiRTX430	PF00249	PTHR12802	PTHR12802:SF43		K12133	GO:00036
PAC:43563	SbiRTX430	SbiRTX430	SbiRTX430	PF00249	PTHR12802	PTHR12802:SF43		K12133	GO:00036
PAC:43563	SbiRTX430	SbiRTX430	SbiRTX430	PF00249	PTHR12802	PTHR12802:SF43		K12133	GO:00036
PAC:43563	SbiRTX430	SbiRTX430	SbiRTX430	PF00249	PTHR12802	PTHR12802:SF43		K12133	GO:00036
PAC:43563	SbiRTX430	SbiRTX430	SbiRTX430	PF00249	PTHR12802	PTHR12802:SF43		K12133	GO:00036
PAC:43563	SbiRTX430	SbiRTX430	SbiRTX430	PF00249	PTHR12802	PTHR12802:SF43		K12133	GO:00036
PAC:43563	SbiRTX430	SbiRTX430	SbiRTX430	PF00249	PTHR12802	PTHR12802:SF43		K12133	GO:00036
PAC:43563	SbiRTX430	SbiRTX430	SbiRTX430	PF00249	PTHR12802	PTHR12802:SF43		K12133	GO:00036
PAC:43563	SbiRTX430	SbiRTX430	SbiRTX430	PF00249	PTHR12802	PTHR12802:SF43		K12133	GO:00036
PAC:43563	SbiRTX430	SbiRTX430	SbiRTX430	PF00249	PTHR12802	PTHR12802:SF43		K12133	GO:00036
PAC:43563	SbiRTX430	SbiRTX430	SbiRTX430.07G04860(		PTHR12802	PTHR12802:SF43			
PAC:43561	SbiRTX430	SbiRTX430	SbiRTX430	PF00335	PTHR19282	PTHR19282:SF262			GO:001602
PAC:43561	SbiRTX430	SbiRTX430	SbiRTX430	PF00324	PTHR11785	PTHR1178	KOG1287		GO:00033:
PAC:43561	SbiRTX430	SbiRTX430	SbiRTX430	PF00324	PTHR11785	PTHR1178	KOG1287		GO:00033:
PAC:43561	SbiRTX430	SbiRTX430	SbiRTX430	PF13520	PTHR11785	PTHR1178	KOG1287		GO:00033:
PAC:43561	SbiRTX430	SbiRTX430	SbiRTX430	PF00324	PTHR11785	PTHR1178	KOG1287		GO:00033:
PAC:43562	SbiRTX430	SbiRTX430	SbiRTX430	PF00562 P	PTHR2085(	EC:2.7.7.6		K16252	GO:00036
PAC:43561	SbiRTX430	SbiRTX430	SbiRTX430	PF00564 P	PTHR22904	PTHR229(	KOG4151		GO:000551
PAC:43561	SbiRTX430	SbiRTX430	SbiRTX430	PF00564 P	PTHR22904	PTHR229(	KOG4151		GO:000551
PAC:43563	SbiRTX430	SbiRTX430	SbiRTX430.07G061700.1.p						GO:004687
PAC:43563	SbiRTX430	SbiRTX430	SbiRTX430	PF00264 P	PTHR1147	EC:1.10.3.1		K00422	GO:00040
PAC:43562	SbiRTX430	SbiRTX430	SbiRTX430.07G07260(		PTHR24006	PTHR24006:SF122			GO:000551
PAC:43562	SbiRTX430	SbiRTX430	SbiRTX430.07G07260(		PTHR24006	PTHR24006:SF122			GO:000551
PAC:43562	SbiRTX430	SbiRTX430	SbiRTX430.07G07260(		PTHR24006	PTHR24006:SF122			GO:000551
PAC:43562	SbiRTX430	SbiRTX430	SbiRTX430.07G07260(		PTHR24006	PTHR24006:SF122			GO:000551
PAC:43563	SbiRTX430	SbiRTX430	SbiRTX430	PF03405	PTHR3115	EC:1.14.19.2		K03921	GO:000663
PAC:43563	SbiRTX430	SbiRTX430	SbiRTX430	PF03405	PTHR3115	EC:1.14.19.2		K03921	GO:000663
PAC:43564	SbiRTX430	SbiRTX430	SbiRTX430.07G077500.1.p						

PAC:43564	SbiRTX430	SbiRTX430	SbiRTX430	PF00304	PTHR33147	PTHR33147:SF12			GO:000695
PAC:43562	SbiRTX430	SbiRTX430	SbiRTX430	PF00107 P	PTHR1169	EC:1.1.1.1	KOG0023	K00083	GO:000827
PAC:43563	SbiRTX430	SbiRTX430	SbiRTX430	PF00847	PTHR31677	PTHR31677:SF21			GO:000367
PAC:43563	SbiRTX430	SbiRTX430	SbiRTX430	PF07714	PTHR2325	EC:2.7.12.	KOG0192		GO:000467
PAC:43562	SbiRTX430	SbiRTX430	SbiRTX430	PF03760	PTHR33493	PTHR33493:SF5			GO:000979
PAC:43562	SbiRTX430	SbiRTX430	SbiRTX430	PF03760	PTHR33493	PTHR33493:SF5			GO:000979
PAC:43563	SbiRTX430	SbiRTX430	SbiRTX430.07G09800		PTHR33228	PTHR33228:SF8			
PAC:43563	SbiRTX430	SbiRTX430	SbiRTX430	PF00722 P	PTHR3106	EC:2.4.1.207		K08235	GO:000455
PAC:43564	SbiRTX430	SbiRTX430	SbiRTX430.07G10200		PTHR18901	PTHR18901:SF28			
PAC:43564	SbiRTX430	SbiRTX430	SbiRTX430.07G10200		PTHR18901	PTHR18901:SF28			
PAC:43564	SbiRTX430	SbiRTX430	SbiRTX430.07G10200		PTHR18901	PTHR18901:SF28			
PAC:43562	SbiRTX430	SbiRTX430	SbiRTX430	PF04927	PTHR31174	PTHR31174:SF6			
PAC:43562	SbiRTX430	SbiRTX430	SbiRTX430	PF13869	PTHR13047	PTHR1304	KOG1689	K14397	GO:000372
PAC:43561	SbiRTX430	SbiRTX430	SbiRTX430	PF00982 P	PTHR1078	EC:2.4.1.15	EC:3.1.3.	K16055	GO:000382
PAC:43561	SbiRTX430	SbiRTX430	SbiRTX430	PF00982 P	PTHR1078	EC:2.4.1.15	EC:3.1.3.	K16055	GO:000382
PAC:43561	SbiRTX430	SbiRTX430	SbiRTX430	PF00982 P	PTHR1078	EC:2.4.1.15			GO:000382
PAC:43561	SbiRTX430	SbiRTX430	SbiRTX430	PF00982 P	PTHR1078	EC:2.4.1.15			GO:000382
PAC:43562	SbiRTX430	SbiRTX430	SbiRTX430	PF00046 P	PTHR24326	PTHR24326:SF188		K09338	GO:000367
PAC:43564	SbiRTX430	SbiRTX430	SbiRTX430	PF00201	PTHR1192	EC:2.4.1.324			GO:000815
PAC:43563	SbiRTX430	SbiRTX430	SbiRTX430	PF00445	PTHR1124	EC:3.1.27.	KOG1642	K01166	GO:000372
PAC:43561	SbiRTX430	SbiRTX430	SbiRTX430	PF00061	PTHR1061	EC:1.14.1	KOG4824	K03098	
PAC:43562	SbiRTX430	SbiRTX430	SbiRTX430	PF01370	PTHR1036	EC:1.2.1.4	KOG1502	K09753	GO:000382
PAC:43561	SbiRTX430	SbiRTX430	SbiRTX430	PF02458	PTHR3164	EC:2.3.1.99		K13065	GO:001674
PAC:43561	SbiRTX430	SbiRTX430	SbiRTX430	PF02458	PTHR3164	EC:2.3.1.133			GO:001674
PAC:43563	SbiRTX430	SbiRTX430	SbiRTX430	PF02458	PTHR31642	PTHR31642:SF25		K13065	GO:001674
PAC:43564	SbiRTX430	SbiRTX430	SbiRTX430	PF00335	PTHR32191	PTHR32191:SF18			GO:001602
PAC:43561	SbiRTX430	SbiRTX430	SbiRTX430	PF00282	PTHR1199	EC:4.1.1.15		K01580	GO:000382
PAC:43561	SbiRTX430	SbiRTX430	SbiRTX430	PF00282	PTHR1199	EC:4.1.1.15		K01580	GO:000382
PAC:43561	SbiRTX430	SbiRTX430	SbiRTX430	PF00282	PTHR1199	EC:4.1.1.15		K01580	GO:000382
PAC:43562	SbiRTX430	SbiRTX430	SbiRTX430	PF03134	PTHR12300	PTHR12300:SF58		K17279	
PAC:43564	SbiRTX430	SbiRTX430	SbiRTX430	PF00067	PTHR2428	EC:1.14.1	KOG0157		GO:000449
PAC:43561	SbiRTX430	SbiRTX430	SbiRTX430	PF00128 P	PTHR1035	EC:3.2.1.1	KOG0471	K01176	GO:000382
PAC:43563	SbiRTX430	SbiRTX430	SbiRTX430	PF12695	PTHR2302	EC:3.1.1.1	KOG1515		
PAC:43561	SbiRTX430	SbiRTX430	SbiRTX430	PF00847	PTHR31241	PTHR31241:SF8			GO:000367
PAC:43564	SbiRTX430	SbiRTX430	SbiRTX430.07G18210		PTHR37696				
PAC:43562	SbiRTX430	SbiRTX430	SbiRTX430	PF06045 P	PTHR3201	EC:4.2.2.23		K18195	GO:000382
PAC:43562	SbiRTX430	SbiRTX430	SbiRTX430	PF06045 P	PTHR3201	EC:4.2.2.23		K18195	GO:000382
PAC:43562	SbiRTX430	SbiRTX430	SbiRTX430	PF06045 P	PTHR3201	EC:4.2.2.23		K18195	GO:000382
PAC:43562	SbiRTX430	SbiRTX430	SbiRTX430.07G191900.1.p						
PAC:43562	SbiRTX430	SbiRTX430	SbiRTX430.07G191900.2.p						
PAC:43564	SbiRTX430	SbiRTX430	SbiRTX430	PF13639	PTHR14155	PTHR1415	KOG1493	K19045	GO:000551
PAC:43561	SbiRTX430	SbiRTX430	SbiRTX430	PF00179	PTHR2406	EC:6.3.2.1	KOG0421	K06688	
PAC:43562	SbiRTX430	SbiRTX430	SbiRTX430	PF00847	PTHR31190				GO:000367
PAC:43562	SbiRTX430	SbiRTX430	SbiRTX430.07G207100.1.p						GO:000367

PAC:43562	SbiRTX430	SbiRTX430	SbiRTX430.07G207100.2.p						
PAC:43563	SbiRTX430	SbiRTX430	SbiRTX430	PF00646	PTHR31264	PTHR31264:SF7			GO:00055
PAC:43562	SbiRTX430	SbiRTX430	SbiRTX430	PF13920	PTHR12183	PTHR1218	KOG4172		GO:000551
PAC:43561	SbiRTX430	SbiRTX430	SbiRTX430	PF00010	PTHR12565	PTHR12565:SF169			GO:004698
PAC:43562	SbiRTX430	SbiRTX430	SbiRTX430.07G220600.1.p						
PAC:43563	SbiRTX430	SbiRTX430	SbiRTX430	PF00128 P	PTHR1035	EC:3.2.1.68		K01214	GO:00038
PAC:43562	SbiRTX430	SbiRTX430	SbiRTX430.07G22620		PTHR34055	PTHR34055:SF2			
PAC:43564	SbiRTX430	SbiRTX430	SbiRTX430	PF03600	PTHR10283	PTHR1028	KOG1281	K14445	GO:00052
PAC:43564	SbiRTX430	SbiRTX430	SbiRTX430	PF03600	PTHR10283	PTHR1028	KOG1281	K14445	GO:00052
PAC:43562	SbiRTX430	SbiRTX430	SbiRTX430	PF00155	PTHR2115	EC:2.6.1.4	KOG2862	K00830	GO:00038
PAC:43561	SbiRTX430	SbiRTX430	SbiRTX430	PF00011	PTHR11527	PTHR11527:SF137		K13993	
PAC:43564	SbiRTX430	SbiRTX430	SbiRTX430	PF13578	PTHR1050	EC:2.1.1.1	KOG1663	K18883	GO:000817
PAC:43564	SbiRTX430	SbiRTX430	SbiRTX430	PF13578	PTHR1050	EC:2.1.1.1	KOG1663		GO:000817
PAC:43562	SbiRTX430	SbiRTX430	SbiRTX430	PF13639	PTHR14155	PTHR14155:SF184		K16285	GO:00055
PAC:43561	SbiRTX430	SbiRTX430	SbiRTX430	PF02469	PTHR32382	PTHR32382:SF12			
PAC:43553	SbiRTX430	SbiRTX430	SbiRTX430	PF07714	PTHR2700	EC:2.7.11.	KOG1187	K04730	GO:000467
PAC:43553	SbiRTX430	SbiRTX430	SbiRTX430	PF01150	PTHR1178	EC:3.6.1.5	KOG1385	K14641	GO:001678
PAC:43553	SbiRTX430	SbiRTX430	SbiRTX430	PF07714	PTHR2700	EC:2.7.11.	KOG1187		GO:000467
PAC:43553	SbiRTX430	SbiRTX430	SbiRTX430	PF07714	PTHR2700	EC:2.7.11.	KOG1187		GO:000467
PAC:43553	SbiRTX430	SbiRTX430	SbiRTX430	PF04525	PTHR31087	PTHR31087:SF23			
PAC:43553	SbiRTX430	SbiRTX430	SbiRTX430.08G02960		PTHR34998	PTHR34998:SF1			
PAC:43553	SbiRTX430	SbiRTX430	SbiRTX430	PF14368	PTHR33076	PTHR33076:SF3			GO:000686
PAC:43553	SbiRTX430	SbiRTX430	SbiRTX430	PF14368	PTHR33076	PTHR33076:SF3			GO:000686
PAC:43555	SbiRTX430	SbiRTX430	SbiRTX430	PF00403	PTHR22814	PTHR2281	KOG1603		GO:00300
PAC:43555	SbiRTX430	SbiRTX430	SbiRTX430	PF00403	PTHR22814	PTHR2281	KOG1603		GO:00300
PAC:43555	SbiRTX430	SbiRTX430	SbiRTX430	PF00403	PTHR22814	PTHR2281	KOG1603		GO:00300
PAC:43554	SbiRTX430	SbiRTX430	SbiRTX430.08G03720		PTHR12626	PTHR12626:SF7			
PAC:43552	SbiRTX430	SbiRTX430	SbiRTX430	PF06404	PTHR33285	PTHR33285:SF2			GO:000557
PAC:43553	SbiRTX430	SbiRTX430	SbiRTX430	PF00149 P	PTHR2295	EC:3.1.3.2	KOG1378		GO:00039
PAC:43553	SbiRTX430	SbiRTX430	SbiRTX430	PF00149 P	PTHR2295	EC:3.1.3.2	KOG1378		GO:00039
PAC:43552	SbiRTX430	SbiRTX430	SbiRTX430	PF00067	PTHR2429	EC:1.14.13	KOG0156		GO:00044
PAC:43553	SbiRTX430	SbiRTX430	SbiRTX430	PF08787	PTHR33681	PTHR33681:SF2			
PAC:43554	SbiRTX430	SbiRTX430	SbiRTX430	PF01301 P	PTHR2342	EC:3.2.1.23			GO:00045
PAC:43552	SbiRTX430	SbiRTX430	SbiRTX430	PF03106	PTHR31282	PTHR31282:SF14			GO:00036
PAC:43553	SbiRTX430	SbiRTX430	SbiRTX430.08G06420		PTHR34666	PTHR34666:SF1			
PAC:43552	SbiRTX430	SbiRTX430	SbiRTX430.08G06570		PTHR34808	PTHR34808:SF2			
PAC:43554	SbiRTX430	SbiRTX430	SbiRTX430	PF00804	PTHR19957	PTHR1995	KOG0810	K08486	GO:000551
PAC:43553	SbiRTX430	SbiRTX430	SbiRTX430	PF00657	PTHR2283	EC:3.1.1.3			GO:001678
PAC:43553	SbiRTX430	SbiRTX430	SbiRTX430	PF00657	PTHR2283	EC:3.1.1.3			GO:001678
PAC:43553	SbiRTX430	SbiRTX430	SbiRTX430	PF00107 P	PTHR1169	EC:1.1.1.54	EC:1.3.1.	K07119	GO:00082
PAC:43554	SbiRTX430	SbiRTX430	SbiRTX430	PF00043 P	PTHR1126	EC:2.5.1.1	KOG0867	K00799	GO:00055
PAC:43553	SbiRTX430	SbiRTX430	SbiRTX430	PF00724	PTHR2289	EC:1.3.1.4	KOG0134	K05894	GO:000382
PAC:43555	SbiRTX430	SbiRTX430	SbiRTX430.08G08620		PTHR24106	PTHR24106:SF146			
PAC:43555	SbiRTX430	SbiRTX430	SbiRTX430	PF00012	PTHR19375	PTHR19375:SF211			GO:000552

PAC:43553	SbiRTX430	SbiRTX430	SbiRTX430.08G10200(		PTHR33133	PTHR33133:SF7			
PAC:43553	SbiRTX430	SbiRTX430	SbiRTX430	PF03083	PTHR10791	PTHR1079	KOG1623	K15382	
PAC:43555	SbiRTX430	SbiRTX430	SbiRTX430	PF00076	PTHR24012			K12741	GO:00001(
PAC:43555	SbiRTX430	SbiRTX430	SbiRTX430	PF00076	PTHR24012			K12741	GO:00001(
PAC:43553	SbiRTX430	SbiRTX430	SbiRTX430	PF03242	PTHR35109	PTHR35109:SF1			GO:000695
PAC:43553	SbiRTX430	SbiRTX430	SbiRTX430	PF03242	PTHR35109	PTHR35109:SF1			GO:000695
PAC:43552	SbiRTX430	SbiRTX430	SbiRTX430.08G12110(		PTHR33167	PTHR33167:SF6			
PAC:43553	SbiRTX430	SbiRTX430	SbiRTX430	PF04535	PTHR11615	PTHR11615:SF156			
PAC:43553	SbiRTX430	SbiRTX430	SbiRTX430.08G146800.1.p						GO:000382
PAC:43553	SbiRTX430	SbiRTX430	SbiRTX430	PF05904	PTHR33167	PTHR33167:SF3			
PAC:43553	SbiRTX430	SbiRTX430	SbiRTX430	PF05904	PTHR33167	PTHR33167:SF3			
PAC:43553	SbiRTX430	SbiRTX430	SbiRTX430	PF05904	PTHR33167	PTHR33167:SF3			
PAC:43553	SbiRTX430	SbiRTX430	SbiRTX430.08G155600.1.p						GO:000367
PAC:43554	SbiRTX430	SbiRTX430	SbiRTX430	PF02577	PTHR15160	PTHR15160:SF7			GO:000451
PAC:43552	SbiRTX430	SbiRTX430	SbiRTX430.08G161000.1.p						
PAC:43554	SbiRTX430	SbiRTX430	SbiRTX430.08G161900.1.p						GO:001714
PAC:43553	SbiRTX430	SbiRTX430	SbiRTX430	PF04043					GO:000485
PAC:43555	SbiRTX430	SbiRTX430	SbiRTX430	PF02309	PTHR31734	PTHR31734:SF13		K14484	GO:00056:
PAC:43553	SbiRTX430	SbiRTX430	SbiRTX430	PF00704	PTHR1117	EC:3.2.1.14			GO:000455
PAC:43554	SbiRTX430	SbiRTX430	SbiRTX430	PF01399	PTHR10678	PTHR10678:SF5		K03036	GO:00055:
PAC:43554	SbiRTX430	SbiRTX430	SbiRTX430	PF01399	PTHR10678	PTHR10678:SF5		K03036	GO:00055:
PAC:43554	SbiRTX430	SbiRTX430	SbiRTX430.08G17900(		PTHR33157	PTHR33157:SF2			
PAC:43554	SbiRTX430	SbiRTX430	SbiRTX430.08G18890(		PTHR33564	PTHR33564:SF3			
PAC:43554	SbiRTX430	SbiRTX430	SbiRTX430	PF05920 P	PTHR11850	PTHR1185	KOG0773		GO:00036
PAC:43554	SbiRTX430	SbiRTX430	SbiRTX430	PF05920 P	PTHR11850	PTHR1185	KOG0773		GO:00036
PAC:43554	SbiRTX430	SbiRTX430	SbiRTX430	PF00004 P	PTHR23070	PTHR23070:SF5			GO:00055
PAC:43554	SbiRTX430	SbiRTX430	SbiRTX430	PF00004 P	PTHR23070	PTHR23070:SF5			GO:00055
PAC:43552	SbiRTX430	SbiRTX430	SbiRTX430.08G203100.1.p						
PAC:43554	SbiRTX430	SbiRTX430	SbiRTX430.08G20360(		PTHR34190				
PAC:43554	SbiRTX430	SbiRTX430	SbiRTX430	PF01277	PTHR33203	PTHR33203:SF6			GO:001251
PAC:43573	SbiRTX430	SbiRTX430	SbiRTX430	PF00025	PTHR24073	PTHR2407	KOG0087	K07904	GO:00039
PAC:43570	SbiRTX430	SbiRTX430	SbiRTX430	PF00280	PTHR33091	PTHR33091:SF8			GO:000486
PAC:43572	SbiRTX430	SbiRTX430	SbiRTX430	PF00280	PTHR33091	PTHR33091:SF8			GO:000486
PAC:43572	SbiRTX430	SbiRTX430	SbiRTX430	PF00280	PTHR33091	PTHR33091:SF8			GO:000486
PAC:43571	SbiRTX430	SbiRTX430	SbiRTX430.09G013900.1.p						
PAC:43570	SbiRTX430	SbiRTX430	SbiRTX430.09G01520(		PTHR36064				
PAC:43572	SbiRTX430	SbiRTX430	SbiRTX430	PF01764	PTHR3182	EC:3.1.1.3	KOG4569		GO:00066
PAC:43570	SbiRTX430	SbiRTX430	SbiRTX430	PF05056	PTHR33103	PTHR33103:SF1			
PAC:43570	SbiRTX430	SbiRTX430	SbiRTX430.09G02180(		PTHR33312	PTHR33312:SF6			
PAC:43572	SbiRTX430	SbiRTX430	SbiRTX430.09G02260(		PTHR35304				
PAC:43571	SbiRTX430	SbiRTX430	SbiRTX430.09G02380(		PTHR31248				
PAC:43571	SbiRTX430	SbiRTX430	SbiRTX430.09G02380(		PTHR31248				
PAC:43573	SbiRTX430	SbiRTX430	SbiRTX430	PF03649	PTHR30028			K02069	GO:00068:
PAC:43571	SbiRTX430	SbiRTX430	SbiRTX430	PF00466	PTHR11560	PTHR11560:SF9		K02864	GO:000562

PAC:43570	SbiRTX430	SbiRTX430	SbiRTX430.09G029500		PTHR37196	PTHR37196:SF1			
PAC:43573	SbiRTX430	SbiRTX430	SbiRTX430	PF00642	PTHR14493	PTHR1449	KOG1595		GO:000551
PAC:43571	SbiRTX430	SbiRTX430	SbiRTX430	PF00141	PTHR3123	EC:1.11.1.7		K00430	GO:000460
PAC:43572	SbiRTX430	SbiRTX430	SbiRTX430	PF00106	PTHR24322	PTHR2432	KOG0725		GO:000402
PAC:43570	SbiRTX430	SbiRTX430	SbiRTX430.09G042800.1.p						
PAC:43570	SbiRTX430	SbiRTX430	SbiRTX430	PF03073	PTHR10057				GO:001602
PAC:43572	SbiRTX430	SbiRTX430	SbiRTX430	PF01764	PTHR2149	EC:3.1.1.3			GO:000662
PAC:43573	SbiRTX430	SbiRTX430	SbiRTX430	PF03876	PTHR1270	EC:2.7.7.6	KOG3298	K16253	GO:000389
PAC:43573	SbiRTX430	SbiRTX430	SbiRTX430	PF03876	PTHR1270	EC:2.7.7.6	KOG3298	K16253	GO:000389
PAC:43570	SbiRTX430	SbiRTX430	SbiRTX430	PF00141	PTHR3123	EC:1.11.1.7		K00430	GO:000460
PAC:43573	SbiRTX430	SbiRTX430	SbiRTX430	PF12734	PTHR35470	PTHR35470:SF2			
PAC:43573	SbiRTX430	SbiRTX430	SbiRTX430	PF03767	PTHR3128	EC:3.1.3.2			GO:000399
PAC:43573	SbiRTX430	SbiRTX430	SbiRTX430	PF03767	PTHR3128	EC:3.1.3.2			GO:000399
PAC:43571	SbiRTX430	SbiRTX430	SbiRTX430	PF00642	PTHR14493	PTHR14493:SF41			GO:004687
PAC:43571	SbiRTX430	SbiRTX430	SbiRTX430	PF00642	PTHR14493	PTHR14493:SF41			GO:004687
PAC:43572	SbiRTX430	SbiRTX430	SbiRTX430	PF00249	PTHR12374	PTHR12374:SF24			GO:000367
PAC:43572	SbiRTX430	SbiRTX430	SbiRTX430	PF07883	PTHR31238	PTHR31238:SF40			GO:003014
PAC:43572	SbiRTX430	SbiRTX430	SbiRTX430	PF02535	PTHR11040	PTHR1104	KOG1558	K14709	GO:000538
PAC:43571	SbiRTX430	SbiRTX430	SbiRTX430	PF03171 P	PTHR1020	EC:1.14.11	KOG0143	K04125	GO:000550
PAC:43571	SbiRTX430	SbiRTX430	SbiRTX430	PF03171 P	PTHR1020	EC:1.14.11	KOG0143		GO:001649
PAC:43571	SbiRTX430	SbiRTX430	SbiRTX430	PF03171 P	PTHR1020	EC:1.14.11	KOG0143		GO:000550
PAC:43571	SbiRTX430	SbiRTX430	SbiRTX430.09G078800		PTHR33834				
PAC:43572	SbiRTX430	SbiRTX430	SbiRTX430	PF03083	PTHR10791	PTHR1079	KOG1623	K15382	
PAC:43570	SbiRTX430	SbiRTX430	SbiRTX430	PF00141	PTHR3123	EC:1.11.1.7		K00430	GO:000460
PAC:43572	SbiRTX430	SbiRTX430	SbiRTX430	PF00650	PTHR10174	PTHR1017	KOG1470		
PAC:43572	SbiRTX430	SbiRTX430	SbiRTX430.09G092400		PTHR10174	PTHR1017	KOG1470		
PAC:43572	SbiRTX430	SbiRTX430	SbiRTX430	PF00650	PTHR10174	PTHR1017	KOG1470		
PAC:43571	SbiRTX430	SbiRTX430	SbiRTX430.09G100500		PTHR31945	PTHR31945:SF7			GO:004698
PAC:43572	SbiRTX430	SbiRTX430	SbiRTX430	PF07107	PTHR33703	PTHR33703:SF3			
PAC:43571	SbiRTX430	SbiRTX430	SbiRTX430	PF00477	PTHR34671	PTHR34671:SF1			
PAC:43571	SbiRTX430	SbiRTX430	SbiRTX430	PF00477	PTHR34671	PTHR34671:SF1			
PAC:43572	SbiRTX430	SbiRTX430	SbiRTX430	PF13839 P	PTHR32285	PTHR32285:SF37			
PAC:43571	SbiRTX430	SbiRTX430	SbiRTX430	PF00847	PTHR31190	PTHR31190:SF21			GO:000367
PAC:43572	SbiRTX430	SbiRTX430	SbiRTX430	PF00141	PTHR3123	EC:1.11.1.7		K00430	GO:000460
PAC:43571	SbiRTX430	SbiRTX430	SbiRTX430.09G119100		PTHR31415	PTHR31415:SF9			
PAC:43570	SbiRTX430	SbiRTX430	SbiRTX430	PF12697	PTHR10992	PTHR1099	KOG1454		
PAC:43570	SbiRTX430	SbiRTX430	SbiRTX430	PF12697	PTHR10992	PTHR1099	KOG1454		
PAC:43570	SbiRTX430	SbiRTX430	SbiRTX430	PF00257	PTHR33346				GO:000695
PAC:43572	SbiRTX430	SbiRTX430	SbiRTX430	PF00332	PTHR3222	EC:3.2.1.73			GO:000455
PAC:43572	SbiRTX430	SbiRTX430	SbiRTX430	PF01027	PTHR23291	PTHR2329	KOG2322	K06890	
PAC:43571	SbiRTX430	SbiRTX430	SbiRTX430	PF14541 P	PTHR1368	EC:3.4.23.	KOG1339		GO:000419
PAC:43573	SbiRTX430	SbiRTX430	SbiRTX430	PF12695	PTHR2302	EC:3.1.1.1	KOG1515		
PAC:43571	SbiRTX430	SbiRTX430	SbiRTX430	PF13410 P	PTHR1126	EC:2.5.1.1	KOG0406	K00799	GO:000551
PAC:43570	SbiRTX430	SbiRTX430	SbiRTX430	PF07714 P	PTHR2700	EC:2.7.11.	KOG1187		GO:000467

PAC:43570	SbiRTX430	SbiRTX430	SbiRTX430	PF07714 P	PTHR27000	EC:2.7.11.1	KOG1187		GO:000467
PAC:43570	SbiRTX430	SbiRTX430	SbiRTX430	PF07714 P	PTHR27000	EC:2.7.11.1	KOG1187		GO:000467
PAC:43572	SbiRTX430	SbiRTX430	SbiRTX430	PF01490	PTHR22950	PTHR22950	KOG1303		
PAC:43572	SbiRTX430	SbiRTX430	SbiRTX430	PF01490	PTHR22950	PTHR22950	KOG1303		
PAC:43572	SbiRTX430	SbiRTX430	SbiRTX430	PF01490	PTHR22950	PTHR22950	KOG1303		
PAC:43571	SbiRTX430	SbiRTX430	SbiRTX430.09G151600		PTHR33728	PTHR33728:SF2			
PAC:43570	SbiRTX430	SbiRTX430	SbiRTX430	PF00650 P	PTHR23324	PTHR23324	KOG1471		GO:000681
PAC:43570	SbiRTX430	SbiRTX430	SbiRTX430	PF00650 P	PTHR23324	PTHR23324	KOG1471		GO:000681
PAC:43570	SbiRTX430	SbiRTX430	SbiRTX430	PF00650 P	PTHR23324	PTHR23324	KOG1471		GO:000681
PAC:43570	SbiRTX430	SbiRTX430	SbiRTX430	PF00249	PTHR10641	PTHR10641:SF617		K09422	GO:000367
PAC:43573	SbiRTX430	SbiRTX430	SbiRTX430	PF00847	PTHR31190	PTHR31190:SF24			GO:000367
PAC:43572	SbiRTX430	SbiRTX430	SbiRTX430	PF00249	PTHR24078	PTHR24078:SF279			GO:000367
PAC:43572	SbiRTX430	SbiRTX430	SbiRTX430.09G161700		PTHR24078	PTHR24078:SF261			GO:000367
PAC:43570	SbiRTX430	SbiRTX430	SbiRTX430	PF01490	PTHR22950	PTHR22950	KOG1303	K13946	
PAC:43573	SbiRTX430	SbiRTX430	SbiRTX430	PF13432	PTHR26312	PTHR26312:SF83			GO:000551
PAC:43573	SbiRTX430	SbiRTX430	SbiRTX430	PF13432	PTHR26312	PTHR26312:SF83			GO:000551
PAC:43571	SbiRTX430	SbiRTX430	SbiRTX430	PF02238	PTHR35308	PTHR35308:SF2			
PAC:43573	SbiRTX430	SbiRTX430	SbiRTX430	PF00481	PTHR13832	EC:3.1.3.16	KOG0698	K14497	GO:000382
PAC:43573	SbiRTX430	SbiRTX430	SbiRTX430	PF00481	PTHR13832	EC:3.1.3.16	KOG0698	K14497	GO:000382
PAC:43572	SbiRTX430	SbiRTX430	SbiRTX430	PF00012	PTHR19375	PTHR19375:SF232		K03283	
PAC:43572	SbiRTX430	SbiRTX430	SbiRTX430	PF01161	PTHR30289			K06910	
PAC:43572	SbiRTX430	SbiRTX430	SbiRTX430	PF01161	PTHR30289				
PAC:43571	SbiRTX430	SbiRTX430	SbiRTX430	PF00847	PTHR31241	PTHR31241:SF2			GO:000367
PAC:43571	SbiRTX430	SbiRTX430	SbiRTX430	PF00248	PTHR11732	EC:1.1.1.2	KOG1577		GO:001649
PAC:43570	SbiRTX430	SbiRTX430	SbiRTX430.09G182600.1.p						
PAC:43570	SbiRTX430	SbiRTX430	SbiRTX430	PF04844 P	PTHR33057	PTHR33057:SF11			GO:000367
PAC:43571	SbiRTX430	SbiRTX430	SbiRTX430	PF14368	PTHR33044	PTHR33044:SF4			
PAC:43572	SbiRTX430	SbiRTX430	SbiRTX430	PF12937	PTHR31348	PTHR31348:SF3			GO:000367
PAC:43571	SbiRTX430	SbiRTX430	SbiRTX430	PF00847	PTHR31677	PTHR31677:SF19			GO:000367
PAC:43571	SbiRTX430	SbiRTX430	SbiRTX430	PF00582	PTHR31966	PTHR31966:SF7			GO:000695
PAC:43572	SbiRTX430	SbiRTX430	SbiRTX430	PF13414	PTHR36326	PTHR36326:SF1			GO:000551
PAC:43570	SbiRTX430	SbiRTX430	SbiRTX430	PF00448 P	PTHR11564	EC:3.6.5.4		K03106	GO:000392
PAC:43570	SbiRTX430	SbiRTX430	SbiRTX430	PF00448 P	PTHR11564	EC:3.6.5.4		K03106	GO:000392
PAC:43572	SbiRTX430	SbiRTX430	SbiRTX430.09G202400.1.p						
PAC:43570	SbiRTX430	SbiRTX430	SbiRTX430	PF04525	PTHR31087	PTHR31087:SF8			
PAC:43570	SbiRTX430	SbiRTX430	SbiRTX430	PF14234	PTHR33835	PTHR33835:SF2			
PAC:43570	SbiRTX430	SbiRTX430	SbiRTX430	PF14234	PTHR33835	PTHR33835:SF2			
PAC:43571	SbiRTX430	SbiRTX430	SbiRTX430	PF07983	PTHR32227	PTHR32227:SF98			
PAC:43571	SbiRTX430	SbiRTX430	SbiRTX430	PF07983	PTHR32227	PTHR32227:SF98			
PAC:43571	SbiRTX430	SbiRTX430	SbiRTX430	PF05097	PTHR33671	PTHR33671:SF4			
PAC:43569	SbiRTX430	SbiRTX430	SbiRTX430	PF03195	PTHR31304	PTHR31304:SF3			
PAC:43573	SbiRTX430	SbiRTX430	SbiRTX430	PF07823	PTHR28141	PTHR28141:SF1			GO:000411
PAC:43570	SbiRTX430	SbiRTX430	SbiRTX430	PF00657	PTHR22835	EC:3.1.1.3			GO:001678
PAC:43572	SbiRTX430	SbiRTX430	SbiRTX430	PF00004 P	PTHR11638	PTHR11638:SF116		K03695	GO:000552

PAC:43571	SbiRTX430	SbiRTX430	SbiRTX430	PF00642	PTHR14493	PTHR14493:SF44			GO:004687
PAC:43571	SbiRTX430	SbiRTX430	SbiRTX430	PF00642	PTHR14493	PTHR14493:SF44			GO:004687
PAC:43572	SbiRTX430	SbiRTX430	SbiRTX430	PF00201	PTHR11926	PTHR11926:SF358			GO:000815
PAC:43570	SbiRTX430	SbiRTX430	SbiRTX430	PF00956	PTHR11875	PTHR1187	KOG1507	K11279	GO:000563
PAC:43570	SbiRTX430	SbiRTX430	SbiRTX430	PF00956	PTHR11875	PTHR1187	KOG1507	K11279	GO:000563
PAC:43571	SbiRTX430	SbiRTX430	SbiRTX430	PF06799	PTHR34214	PTHR34214:SF1			
PAC:43571	SbiRTX430	SbiRTX430	SbiRTX430	PF03641	PTHR31223	PTHR31223:SF16			
PAC:43571	SbiRTX430	SbiRTX430	SbiRTX430	PF03641	PTHR31223	PTHR31223:SF16			
PAC:43572	SbiRTX430	SbiRTX430	SbiRTX430.09G22880		PTHR23241				
PAC:43572	SbiRTX430	SbiRTX430	SbiRTX430.09G228800.2.p						
PAC:43572	SbiRTX430	SbiRTX430	SbiRTX430.09G22880		PTHR23241	PTHR23241:SF44			
PAC:43572	SbiRTX430	SbiRTX430	SbiRTX430.09G22880		PTHR23241				
PAC:43572	SbiRTX430	SbiRTX430	SbiRTX430.09G22880		PTHR23241				
PAC:43572	SbiRTX430	SbiRTX430	SbiRTX430.09G22880		PTHR23241	PTHR23241:SF44			
PAC:43571	SbiRTX430	SbiRTX430	SbiRTX430	PF00650 P	PTHR23324	PTHR2332	KOG1471		GO:000521
PAC:43571	SbiRTX430	SbiRTX430	SbiRTX430	PF00650 P	PTHR23324	PTHR2332	KOG1471		GO:000521
PAC:43571	SbiRTX430	SbiRTX430	SbiRTX430	PF00650 P	PTHR23324	PTHR2332	KOG1471		GO:000521
PAC:43571	SbiRTX430	SbiRTX430	SbiRTX430	PF00650 P	PTHR23324	PTHR2332	KOG1471		GO:000521
PAC:43571	SbiRTX430	SbiRTX430	SbiRTX430	PF00650 P	PTHR23324	PTHR2332	KOG1471		GO:000521
PAC:43570	SbiRTX430	SbiRTX430	SbiRTX430	PF00847 P	PTHR31140	PTHR31140:SF3		K09287	GO:000367
PAC:43570	SbiRTX430	SbiRTX430	SbiRTX430	PF07797	PTHR33130	PTHR33130:SF13			
PAC:43570	SbiRTX430	SbiRTX430	SbiRTX430	PF07797	PTHR33130	PTHR33130:SF13			
PAC:43573	SbiRTX430	SbiRTX430	SbiRTX430	PF14144	PTHR22952	PTHR22952:SF210			GO:000635
PAC:43572	SbiRTX430	SbiRTX430	SbiRTX430	PF00226 P	PTHR24078	PTHR2407	KOG0714	K09510	GO:000645
PAC:43572	SbiRTX430	SbiRTX430	SbiRTX430	PF05078	PTHR31621	PTHR31621:SF0			
PAC:43573	SbiRTX430	SbiRTX430	SbiRTX430	PF00025	PTHR24073	PTHR2407	KOG0087	K07904	GO:000392
PAC:43571	SbiRTX430	SbiRTX430	SbiRTX430.09G25150		PTHR33264	PTHR33264:SF6			
PAC:43573	SbiRTX430	SbiRTX430	SbiRTX430	PF13672	PTHR1383	EC:3.1.3.1	KOG0698	K14497	GO:000382
PAC:43570	SbiRTX430	SbiRTX430	SbiRTX430	PF01277	PTHR33203	PTHR33203:SF4			GO:001251
PAC:43571	SbiRTX430	SbiRTX430	SbiRTX430	PF07816	PTHR33095	PTHR33095:SF3			
PAC:43571	SbiRTX430	SbiRTX430	SbiRTX430	PF00249	PTHR24078	PTHR24078:SF177			GO:000367
PAC:43572	SbiRTX430	SbiRTX430	SbiRTX430	PF04844	PTHR33057	PTHR33057:SF25			
PAC:43571	SbiRTX430	SbiRTX430	SbiRTX430.09G26650		PTHR34967	PTHR34967:SF2			
PAC:43570	SbiRTX430	SbiRTX430	SbiRTX430	PF00026	PTHR1368	EC:3.4.23.	KOG1339		GO:000419
PAC:43570	SbiRTX430	SbiRTX430	SbiRTX430	PF02453	PTHR10994	PTHR1099	KOG1792		
PAC:43546	SbiRTX430	SbiRTX430	SbiRTX430.10G00270		PTHR31851	PTHR31851:SF16			
PAC:43546	SbiRTX430	SbiRTX430	SbiRTX430.10G00270		PTHR31851	PTHR31851:SF16			
PAC:43546	SbiRTX430	SbiRTX430	SbiRTX430.10G00270		PTHR31851	PTHR31851:SF16			
PAC:43547	SbiRTX430	SbiRTX430	SbiRTX430	PF13238	PTHR2335	EC:2.7.4.1	KOG3079	K13800	GO:000412
PAC:43548	SbiRTX430	SbiRTX430	SbiRTX430	PF03760	PTHR33493	PTHR33493:SF6			GO:000979
PAC:43545	SbiRTX430	SbiRTX430	SbiRTX430.10G011600.1.p						GO:000367
PAC:43545	SbiRTX430	SbiRTX430	SbiRTX430	PF06592	PTHR34267	PTHR34267:SF1			
PAC:43546	SbiRTX430	SbiRTX430	SbiRTX430.10G01430		PTHR3510	EC:5.99.1.3			
PAC:43546	SbiRTX430	SbiRTX430	SbiRTX430	PF00538	PTHR11467	PTHR11467:SF50		K11275	GO:000078

PAC:43546	SbiRTX430	SbiRTX430	SbiRTX430	PF13768	PTHR10579	PTHR10579:SF57			
PAC:43546	SbiRTX430	SbiRTX430	SbiRTX430	PF01176	PTHR21668	PTHR2166	KOG3403	K03236	GO:000372
PAC:43546	SbiRTX430	SbiRTX430	SbiRTX430	PF00657	PTHR2283	EC:3.1.1.3			GO:001678
PAC:43548	SbiRTX430	SbiRTX430	SbiRTX430.10G04210		PTHR33673	PTHR33673:SF2			
PAC:43545	SbiRTX430	SbiRTX430	SbiRTX430	PF00257	PTHR33346	PTHR33346:SF3			GO:000695
PAC:43546	SbiRTX430	SbiRTX430	SbiRTX430.10G04550		PTHR24006	PTHR2400	KOG1947		GO:000551
PAC:43546	SbiRTX430	SbiRTX430	SbiRTX430	PF00657	PTHR2283	EC:3.1.1.1			GO:001678
PAC:43545	SbiRTX430	SbiRTX430	SbiRTX430	PF00657	PTHR2283	EC:3.1.1.1			GO:001678
PAC:43548	SbiRTX430	SbiRTX430	SbiRTX430	PF03168	PTHR31852	PTHR31852:SF25			
PAC:43548	SbiRTX430	SbiRTX430	SbiRTX430	PF03168	PTHR31852	PTHR31852:SF25			
PAC:43545	SbiRTX430	SbiRTX430	SbiRTX430	PF14547	PTHR31731	PTHR31731:SF9			
PAC:43548	SbiRTX430	SbiRTX430	SbiRTX430	PF03030	PTHR3199	EC:3.6.1.1		K01507	GO:000442
PAC:43548	SbiRTX430	SbiRTX430	SbiRTX430	PF03030	PTHR3199	EC:3.6.1.1		K01507	GO:000442
PAC:43548	SbiRTX430	SbiRTX430	SbiRTX430	PF03030	PTHR3199	EC:3.6.1.1			GO:000442
PAC:43548	SbiRTX430	SbiRTX430	SbiRTX430	PF03030	PTHR3199	EC:3.6.1.1			GO:000442
PAC:43548	SbiRTX430	SbiRTX430	SbiRTX430	PF03030	PTHR3199	EC:3.6.1.1			GO:000442
PAC:43548	SbiRTX430	SbiRTX430	SbiRTX430	PF03030	PTHR3199	EC:3.6.1.1			GO:000442
PAC:43545	SbiRTX430	SbiRTX430	SbiRTX430.10G06460		PTHR33915	PTHR33915:SF1			
PAC:43545	SbiRTX430	SbiRTX430	SbiRTX430	PF00271 P	PTHR1079	EC:3.6.4.12		K11643	GO:000367
PAC:43547	SbiRTX430	SbiRTX430	SbiRTX430	PF00226	PTHR24078				GO:000814
PAC:43548	SbiRTX430	SbiRTX430	SbiRTX430.10G076600.1.p						
PAC:43545	SbiRTX430	SbiRTX430	SbiRTX430	PF03171 P	PTHR1020	EC:1.14.20	KOG0143		GO:001649
PAC:43544	SbiRTX430	SbiRTX430	SbiRTX430	PF00201	PTHR11926	PTHR11926:SF406			GO:000815
PAC:43546	SbiRTX430	SbiRTX430	SbiRTX430	PF03254	PTHR3188	EC:2.4.1.69		K13681	GO:000810
PAC:43544	SbiRTX430	SbiRTX430	SbiRTX430	PF02672	PTHR33921	PTHR33921:SF1			GO:000554
PAC:43544	SbiRTX430	SbiRTX430	SbiRTX430	PF02672	PTHR33921	PTHR33921:SF1			GO:000554
PAC:43545	SbiRTX430	SbiRTX430	SbiRTX430	PF12695	PTHR2302	EC:3.1.1.1	KOG1515		
PAC:43545	SbiRTX430	SbiRTX430	SbiRTX430	PF00135	PTHR2302	EC:3.1.1.1	KOG1515		
PAC:43548	SbiRTX430	SbiRTX430	SbiRTX430.10G09260		PTHR34046	PTHR34046:SF3			
PAC:43544	SbiRTX430	SbiRTX430	SbiRTX430	PF00011	PTHR11527	PTHR11527:SF166		K13993	
PAC:43545	SbiRTX430	SbiRTX430	SbiRTX430	PF04674	PTHR31279	PTHR31279:SF15			
PAC:43546	SbiRTX430	SbiRTX430	SbiRTX430	PF00676	PTHR1151	EC:1.2.4.1		K00161	GO:000473
PAC:43546	SbiRTX430	SbiRTX430	SbiRTX430	PF00011	PTHR11527	PTHR1152	KOG3591	K13993	
PAC:43545	SbiRTX430	SbiRTX430	SbiRTX430	PF00249	PTHR10641	PTHR10641:SF627		K09422	GO:000367
PAC:43548	SbiRTX430	SbiRTX430	SbiRTX430	PF08392 P	PTHR3156	EC:2.3.1.199		K15397	GO:000382
PAC:43547	SbiRTX430	SbiRTX430	SbiRTX430	PF13962	PTHR24177	PTHR24177:SF43			
PAC:43547	SbiRTX430	SbiRTX430	SbiRTX430	PF13962	PTHR24177	PTHR24177:SF43			
PAC:43545	SbiRTX430	SbiRTX430	SbiRTX430.10G13280		PTHR31133	PTHR31133:SF2			GO:000635
PAC:43545	SbiRTX430	SbiRTX430	SbiRTX430.10G13280		PTHR31133	PTHR31133:SF2			
PAC:43546	SbiRTX430	SbiRTX430	SbiRTX430	PF03760	PTHR33493	PTHR33493:SF4			GO:000979
PAC:43546	SbiRTX430	SbiRTX430	SbiRTX430.10G142700.1.p						
PAC:43545	SbiRTX430	SbiRTX430	SbiRTX430	PF00254 P	PTHR1051	EC:5.2.1.8	KOG0543	K09571	GO:000551
PAC:43547	SbiRTX430	SbiRTX430	SbiRTX430	PF03479	PTHR31100	PTHR31100:SF5			
PAC:43545	SbiRTX430	SbiRTX430	SbiRTX430.10G144900.1.p						

PAC:43548	SbiRTX430	SbiRTX430	SbiRTX430	PF00389 P	PTHR1099	EC:1.2.1.2	KOG0069	K00122	GO:000815
PAC:43546	SbiRTX430	SbiRTX430	SbiRTX430	PF02453	PTHR10994	PTHR1099	KOG1792		
PAC:43545	SbiRTX430	SbiRTX430	SbiRTX430.10G16030		PTHR33513	PTHR33513:SF3			
PAC:43547	SbiRTX430	SbiRTX430	SbiRTX430.10G169400.1.p						
PAC:43546	SbiRTX430	SbiRTX430	SbiRTX430	PF10494	PTHR1524	EC:2.7.11.1		K08880	
PAC:43546	SbiRTX430	SbiRTX430	SbiRTX430	PF10494	PTHR1524	EC:2.7.11.1		K08880	
PAC:43546	SbiRTX430	SbiRTX430	SbiRTX430	PF00447	PTHR10015	PTHR10015:SF152			GO:000370
PAC:43546	SbiRTX430	SbiRTX430	SbiRTX430	PF00447	PTHR10015	PTHR10015:SF152		K09419	GO:000370
PAC:43546	SbiRTX430	SbiRTX430	SbiRTX430.10G177700.1.p						
PAC:43546	SbiRTX430	SbiRTX430	SbiRTX430	PF00314	PTHR31048	PTHR31048:SF3			
PAC:43547	SbiRTX430	SbiRTX430	SbiRTX430	PF00232	PTHR1035	EC:3.2.1.21		K01188	GO:000455
PAC:43546	SbiRTX430	SbiRTX430	SbiRTX430	PF02535	PTHR11040	PTHR1104	KOG1558	K14709	GO:000538
PAC:43544	SbiRTX430	SbiRTX430	SbiRTX430	PF01301	PTHR2342	EC:3.2.1.23			GO:000455
PAC:43544	SbiRTX430	SbiRTX430	SbiRTX430	PF01301	PTHR2342	EC:3.2.1.23			GO:000455
PAC:43544	SbiRTX430	SbiRTX430	SbiRTX430	PF13410 P	PTHR1126	EC:2.5.1.1	KOG0406	K00799	GO:000551
PAC:43544	SbiRTX430	SbiRTX430	SbiRTX430.10G188100.1.p						
PAC:43544	SbiRTX430	SbiRTX430	SbiRTX430.10G188100.2.p						
PAC:43545	SbiRTX430	SbiRTX430	SbiRTX430	PF00171	PTHR1169	EC:1.2.1.5		K12355	GO:000815
PAC:43545	SbiRTX430	SbiRTX430	SbiRTX430	PF00201	PTHR1192	EC:2.4.1.271			GO:000815
PAC:43545	SbiRTX430	SbiRTX430	SbiRTX430	PF00201	PTHR1192	EC:2.4.1.271			GO:000815
PAC:43546	SbiRTX430	SbiRTX430	SbiRTX430.10G191100.1.p						
PAC:43547	SbiRTX430	SbiRTX430	SbiRTX430	PF13639	PTHR14155	PTHR14155:SF175		K16282	GO:000551
PAC:43547	SbiRTX430	SbiRTX430	SbiRTX430	PF00578	PTHR1043	EC:1.11.1.	KOG0541		GO:001620
PAC:43546	SbiRTX430	SbiRTX430	SbiRTX430	PF14547	PTHR31731	PTHR31731:SF1			GO:000519
PAC:43546	SbiRTX430	SbiRTX430	SbiRTX430	PF14547	PTHR31731	PTHR31731:SF1			GO:000519
PAC:43546	SbiRTX430	SbiRTX430	SbiRTX430	PF14547	PTHR31731	PTHR31731:SF1			GO:000519
PAC:43545	SbiRTX430	SbiRTX430	SbiRTX430	PF00411	PTHR11759	PTHR1175	KOG0407	K02955	GO:000373
PAC:43548	SbiRTX430	SbiRTX430	SbiRTX430.10G223500.1.p						
PAC:43545	SbiRTX430	SbiRTX430	SbiRTX430	PF03006	PTHR20855	PTHR2085	KOG0748	K07297	GO:001602
PAC:43544	SbiRTX430	SbiRTX430	SbiRTX430	PF00335	PTHR32191	PTHR32191:SF21			GO:001602
PAC:43544	SbiRTX430	SbiRTX430	SbiRTX430	PF00162	PTHR1140	EC:2.7.2.3		K00927	GO:000461
PAC:43544	SbiRTX430	SbiRTX430	SbiRTX430	PF00162	PTHR1140	EC:2.7.2.3		K00927	GO:000461
PAC:43544	SbiRTX430	SbiRTX430	SbiRTX430	PF00162	PTHR1140	EC:2.7.2.3		K00927	GO:000461
PAC:43548	SbiRTX430	SbiRTX430	SbiRTX430	PF11976	PTHR10666	PTHR1066	KOG0001 K	K08770	GO:000551
PAC:43548	SbiRTX430	SbiRTX430	SbiRTX430	PF11976	PTHR10666	PTHR1066	KOG0001 K	K08770	GO:000551
PAC:43548	SbiRTX430	SbiRTX430	SbiRTX430	PF11976	PTHR10666		KOG0001	K04551 K0	GO:000551
PAC:43546	SbiRTX430	SbiRTX430	SbiRTX430	PF11976	PTHR10666		KOG0001 K	K04551 K0	GO:000551
PAC:43546	SbiRTX430	SbiRTX430	SbiRTX430	PF11976	PTHR10666		KOG0001 K	K02927 K0	GO:000551
PAC:43546	SbiRTX430	SbiRTX430	SbiRTX430	PF02298	PTHR33021	PTHR33021:SF60			GO:000905
PAC:43544	SbiRTX430	SbiRTX430	SbiRTX430	PF02679		EC:4.4.1.19			GO:000382
PAC:43547	SbiRTX430	SbiRTX430	SbiRTX430.10G249200.1.p						
PAC:43547	SbiRTX430	SbiRTX430	SbiRTX430.10G249200.2.p						
PAC:43547	SbiRTX430	SbiRTX430	SbiRTX430	PF13499 P	PTHR10891		KOG0027		GO:000550
PAC:43545	SbiRTX430	SbiRTX430	SbiRTX430	PF00005	PTHR2422	EC:3.6.3.2	KOG2355		GO:000552

PAC:43545	SbiRTX430	SbiRTX430	SbiRTX430	PF00005	PTHR2422(	EC:3.6.3.27			GO:000552
PAC:43546	SbiRTX430	SbiRTX430	SbiRTX430	PF00722 P	PTHR3106	EC:2.4.1.207	EC:3.2.1	K14504	GO:000455
PAC:43545	SbiRTX430	SbiRTX430	SbiRTX430	PF00481	PTHR1383	EC:3.1.3.1(	KOG0698	K17506	GO:000382
PAC:43545	SbiRTX430	SbiRTX430	SbiRTX430	PF00481	PTHR1383	EC:3.1.3.1(	KOG0698		GO:000382
PAC:43546	SbiRTX430	SbiRTX430	SbiRTX430.10G26770(		PTHR34278	PTHR34278:SF1			GO:000905
PAC:43545	SbiRTX430	SbiRTX430	SbiRTX430	PF04937	PTHR32166	PTHR32166:SF21			GO:000367
PAC:43545	SbiRTX430	SbiRTX430	SbiRTX430	PF04937	PTHR32166	PTHR32166:SF21			GO:000367
PAC:43545	SbiRTX430	SbiRTX430	SbiRTX430	PF04937	PTHR32166	PTHR32166:SF21			GO:000367
PAC:43548	SbiRTX430	SbiRTX430	SbiRTX430	PF04043	PTHR3108(	EC:3.1.1.11			GO:000485
PAC:43545	SbiRTX430	SbiRTX430	SbiRTX430	PF04043	PTHR3108(	EC:3.1.1.11			GO:000485
PAC:43546	SbiRTX430	SbiRTX430	SbiRTX430	PF00076	PTHR15241		KOG0107 K	K12897	GO:000016
PAC:43546	SbiRTX430	SbiRTX430	SbiRTX430	PF00076	PTHR15241			K12897	GO:000016
PAC:43546	SbiRTX430	SbiRTX430	SbiRTX430	PF00076	PTHR15241		KOG0107 K	K12897	GO:000016
PAC:43546	SbiRTX430	SbiRTX430	SbiRTX430	PF03087	PTHR33070	PTHR33070:SF21			
PAC:43564	SbiRTX430	SbiRTX430	SbiRTX430.K007600.1		PTHR33187	PTHR33187:SF1			

Best-hit-ar	Best-hit-ar	Best-hit-cl	Best-hit-cl	Best-hit-ric	Best-hit-ric	putative	expressed		
AT5G23890				LOC_Os03	oxidoreduc	putative	expressed		
AT5G23890				LOC_Os03	oxidoreduc	expressed protein			
AT4G1410(	transferase	transferring glycosyl groups			LOC_Os03	expressed protein			
AT4G1410(	transferase	transferring glycosyl groups			LOC_Os03g63380				
52 GO:0016758		Cre07.g32:	(1 of 1) PTHR11926//PTHR11926::			LOC_Os03	regulatory (	putative	expressed
		Cre02.g08:	(1 of 3) K15	member 1,	ENT1_2_3)	short chair	expressed		
AT4G1002(	hydroxysteroid dehydrogenase 5			LOC_Os03	oxidoreduc	expressed			
				LOC_Os03	harpin-indu	expressed			
AT3G1687(	GATA transcription factor 17			LOC_Os01	GATA zinc f	expressed			
AT3G1687(	GATA transcription factor 17			LOC_Os01	GATA zinc f	alpha/beta	expressed		
24		Cre12.g54(	(1 of 3) 3.1	LOC_Os03	hydrolase	alpha/beta	expressed		
24		Cre12.g54(	(1 of 3) 3.1	LOC_Os03	hydrolase	alpha/beta	expressed		
24		Cre12.g54(	(1 of 3) 3.1	LOC_Os03	hydrolase	expressed			
90 GO:0005739 GO:0006355				LOC_Os11	mTERF fam	putative	expressed		
AT1G7485(	plastid transcriptionally active 2			LOC_Os03	PPR repeat	putative	expressed		
AT2G3970(	expansin A	Cre17.g72:	(1 of 11) PT	LOC_Os03	expansin precursor				
AT3G5512(	Chalcone-flavanone isomerase family protein					putative	expressed		
				LOC_Os03	leaf senescence related protein				
AT3G5752(	seed imbib	Cre16.g66(	putative raffinose synthase						
AT3G5752(	seed imbib	Cre16.g66(	putative raffinose synthase			expressed protein			
AT3G0803(	Protein of u	DUF642			LOC_Os03	putative	expressed		
AT1G5536(	Protein of Unknown Function (DUF			LOC_Os03	carboxyl-te	putative	expressed		
AT4G3881(	Calcium-binding EF-hand family p			LOC_Os10	EF hand fa	putative	expressed		
				LOC_Os03	uncharacte	putative	expressed		
				LOC_Os03	basic proli	putative	expressed		
08 GO:0008234				LOC_Os01	cysteine pr	expressed			
AT3G2264(	cupin family protein			LOC_Os03	cupin doma	expressed			
AT3G2264(	cupin family protein			LOC_Os03	cupin domain containing protein				
AT2G0276(	ubiquiting-	Cre06.g29	Ubiquitin-conjugating enzyme E2			putative	expressed		
AT3G4411(	DNAJ homo	Cre17.g70:	DnaJ-like p	LOC_Os03	chaperone	putative	expressed		
AT3G4411(	DNAJ homo	Cre17.g70:	DnaJ-like p	LOC_Os03	chaperone	putative	expressed		
AT3G4411(	DNAJ homo	Cre17.g70:	DnaJ-like p	LOC_Os03	chaperone	putative	expressed		
AT1G1718(	glutathione	Cre11.g46:	(1 of 15) 2.	LOC_Os03	glutathione S-transferase				
15		Cre11.g46:	(1 of 15) 2.5.1.18 - Glutathione tra			putative	expressed		
AT2G3130!	inhibitor-3			LOC_Os05	protein pho	chloroplas	putative	expressed	
AT1G3133(	photosyste	Cre09.g41:	Photosyste	LOC_Os03	photosyste	chloroplas	putative	expressed	
AT1G3133(	photosyste	Cre09.g41:	Photosyste	LOC_Os03	photosyste	chloroplas	putative	expressed	
AT1G3133(	photosyste	Cre09.g41:	Photosyste	LOC_Os03	photosyste	putative	expressed		
		Cre09.g39(	Presequen	LOC_Os03	heat shock protein DnaJ				
AT1G1014(	Uncharacterised conserved protei			LOC_Os03	expressed protein				
				LOC_Os03	expressed	expressed			
00 GO:0006355 GO:0043565				LOC_Os03	WRKY3	expressed			
00 GO:0006355 GO:0043565				LOC_Os03	WRKY3	expressed			

)0 GO:0006355 GO:0043565				LOC_Os03	WRKY3	putative	expressed	
50		Cre16.g64!	Universal s	LOC_Os03	universal s	putative	expressed	
				LOC_Os03	late embry	putative	expressed	
AT3G2249(	Seed maturation protein			LOC_Os03	late embry	transmemb	putative	expressed
AT1G0307(	Bax inhibit	Cre01.g03!	(1 of 1) PTH	ISOFORM I	LOC_Os03	putative	expressed	
52 GO:0016758		Cre07.g32:	(1 of 1) PTH	LOC_Os03	anthocyani	Tramtrack	Broad Con	expressed
15				LOC_Os03	BTBN9 - Bri	pfkB family	putative	expressed
AT5G5873(	pfkB-like carbohydrate kinase fam			LOC_Os03	kinase	pfkB family	putative	expressed
AT5G5873(	pfkB-like carbohydrate kinase fam			LOC_Os03	kinase			
AT5G05250				LOC_Os03	expressed protein			
				LOC_Os03	expressed protein			
				LOC_Os03	expressed protein			
				LOC_Os03	expressed	putative	expressed	
AT1G2748(	alpha/beta	Cre12.g55!	(1 of 5) 2.3	LOC_Os03	lecithin ch	M50 family	putative	expressed
AT1G1787(	ethylene-dependent gravitropism-			LOC_Os03	peptidase			
				LOC_Os03	expressed	putative	expressed	
AT1G0957(	phytochrome A			LOC_Os03	phytochror	putative	expressed	
AT1G0957(	phytochrome A			LOC_Os03	phytochror	putative	expressed	
AT1G0957(	phytochrome A			LOC_Os03	phytochror	putative	expressed	
				LOC_Os08	seed matur	putative	expressed	
				LOC_Os03	DnaK family protein			
AT5G2209(	Protein of unknown function (DUF			LOC_Os03	expressed	putative	expressed	
31 GO:0008380		Cre13.g56	Pre-mRNA	LOC_Os03	pre-mRNA-	putative	expressed	
31 GO:0008380		Cre13.g56	Pre-mRNA	LOC_Os03	pre-mRNA-	putative	expressed	
31 GO:0008380		Cre13.g56	Pre-mRNA	LOC_Os03	pre-mRNA-	putative	expressed	
AT1G5502(	lipoxygena:	Cre12.g51:	(1 of 1) 1.1:	LOC_Os03	lipoxygena:	putative	expressed	
AT1G5502(	lipoxygena:	Cre12.g51:	(1 of 1) 1.1:	LOC_Os03	lipoxygena:	putative	expressed	
AT1G5502(	lipoxygena:	Cre12.g51:	(1 of 1) 1.1:	LOC_Os03	lipoxygena:	putative	expressed	
AT1G5502(	lipoxygena:	Cre12.g51:	(1 of 1) 1.1:	LOC_Os03	lipoxygena:	putative	expressed	
AT1G5502(	lipoxygena:	Cre12.g51:	(1 of 1) 1.1:	LOC_Os03	lipoxygena:	putative	expressed	
AT1G5502(	lipoxygena:	Cre12.g51:	(1 of 1) 1.1:	LOC_Os03	lipoxygena:	putative	expressed	
AT1G5502(	lipoxygena:	Cre12.g51:	(1 of 1) 1.1:	LOC_Os03	lipoxygena:	putative	expressed	
AT1G5502(	lipoxygena:	Cre12.g51:	(1 of 1) 1.1:	LOC_Os03	lipoxygena:	putative	expressed	
AT3G0157(	Oleosin family protein			LOC_Os03	oleosin	putative	expressed	
		Cre07.g34(	(1 of 1) PTH	LOC_Os03	class I glut	expressed		
AT4G2162(	glycine-rich protein			LOC_Os03	GCRP4 - Gl	putative	expressed	
AT3G1292(	SBP (S-ribonuclease binding prote			LOC_Os03	protein bin	putative	expressed	
AT2G3209(	Lactoylglutathione lyase / glyoxala			LOC_Os03	glyoxalase	putative	expressed	
AT2G3209(	Lactoylglutathione lyase / glyoxala			LOC_Os03	glyoxalase	putative	expressed	
AT2G3209(	Lactoylglutathione lyase / glyoxala			LOC_Os03	glyoxalase	putative	expressed	
)2 GO:0055114				LOC_Os03	2-aminoetl	putative	expressed	
)2 GO:0055114				LOC_Os03	2-aminoethanethiol dioxygenase			
		Cre15.g64:	(1 of 3) PF(	LOC_Os03	expressed	putative	expressed	
AT3G1900(	2-oxoglutarate (2OG) and Fe(II)-de			LOC_Os03	gibberellin	putative	expressed	

AT3G1900(	2-oxoglutarate (2OG) and Fe(II)-de			LOC_Os03	gibberellin	expressed	
15				LOC_Os10	OsFBDUF4	putative	expressed
AT1G7275(	translocas	Cre10.g43	(1 of 1) PTH	LOC_Os10	mitochond	putative	expressed
AT1G6466(	methionine	Cre10.g42(	Methionine	LOC_Os10	cystathioni	putative	expressed
AT4G1038(	NOD26-like intrinsic protein 5;1			LOC_Os10	aquaporin protein		
AT4G2558(	CAP160 protein			LOC_Os10	expressed protein		
AT4G2558(	CAP160 protein			LOC_Os10	expressed	putative	expressed
AT1G5292(	G protein c	Cre03.g15	LanC lantib	LOC_Os10	lanC-like p	putative	expressed
AT1G5292(	G protein c	Cre03.g15	LanC lantib	LOC_Os10	lanC-like p	putative	expressed
24 GO:0004553 GO:00(		Cre01.g02(	Alpha-gala	LOC_Os10	alpha-gala	putative	expressed
AT1G1781(	beta-tonop	Cre12.g54	Aquaporin	LOC_Os10	aquaporin protein		
				LOC_Os10	expressed protein		
						putative	expressed
77		Cre02.g11(	(1 of 23) PT	LOC_Os10	myb-relate	putative	expressed
		Cre12.g55	(1 of 1) KO(	LOC_Os10	endo-beta-	putative	expressed
		Cre12.g55	(1 of 1) KO(	LOC_Os10	endo-beta-	putative	expressed
		Cre12.g55	(1 of 1) KO(	LOC_Os10	endo-beta-	putative	expressed
		Cre12.g55	(1 of 1) KO(	LOC_Os10	endo-beta-	expressed	
				LOC_Os10	exostosin family domain containing protein		
						putative	expressed
AT1G0226(	Divalent io	Cre09.g39	(1 of 2) PTH	LOC_Os10	Citrate trar	putative	expressed
AT1G0226(	Divalent io	Cre09.g39	(1 of 2) PTH	LOC_Os10	Citrate trar	putative	expressed
AT1G0226(	Divalent io	Cre09.g39	(1 of 2) PTH	LOC_Os10	Citrate trar	putative	expressed
AT1G0226(	Divalent io	Cre09.g39	(1 of 2) PTH	LOC_Os10	Citrate trar	putative	expressed
AT1G0226(	Divalent io	Cre09.g39	(1 of 2) PTH	LOC_Os10	Citrate trar	putative	expressed
AT1G0226(	Divalent io	Cre09.g39	(1 of 2) PTH	LOC_Os10	Citrate trar	putative	expressed
AT2G3894(	phosphate	Cre02.g07	Proton/pho	LOC_Os10	inorganic p	putative	expressed
77				LOC_Os10	MYB family transcription factor		
						putative	expressed
AT3G1193(	Adenine nu	Cre16.g64	Universal s	LOC_Os10	universal s	putative	expressed
75 GO:0016020				LOC_Os10	xylosyltransferase		
AT1G05430				LOC_Os10	expressed	putative	expressed
15		Cre16.g68	Glutathion	LOC_Os10	glutathione	putative	expressed
AT4G0967(	Oxidoreductase family protein			LOC_Os10	uncharacte	LOC_Os10	expressed protein
AT3G1398(	(1 of 4) PTH	CHROMOS	P1 CLONE:	MDC16-RELATED		putative	expressed
				LOC_Os10	phi-1-like p	expressed	
				LOC_Os10	POEI3 - Pol	putative	expressed
01 GO:0006979 GO:0020037 GO:0055114				LOC_Os10	peroxidase precursor		
47							
31 GO:0016491 GO:0045300 GO:0055114							
31 GO:0016491 GO:0045300 GO:0055114							
31 GO:0016491 GO:0045300 GO:0055114							
AT4G1912(	S-adenosyl-L-methionine-dependent methyltransferases superfamily protein						
AT4G1912(	S-adenosyl-L-methionine-dependent methyltransferases superfamily protein						

AT4G1912(	S-adenosyl-L-methionine-dependent methyltransferases					putative	expressed	
AT5G1706(	ADP-ribosy	Cre12.g48	ARF/SAR su	LOC_Os10	miro	putative	expressed	
AT5G4680(	Mitochond	Cre04.g22	Low-CO2-i	LOC_Os10	mitochond	putative	expressed	
AT5G4680(	Mitochond	Cre04.g22	Low-CO2-i	LOC_Os10	mitochond	expressed		
				LOC_Os10	RALFL26 - I	PKG and P	expressed	
72 GO:0004713 GO:0005524 GO:0006468				LOC_Os10	AGC_PVPK	putative	expressed	
AT1G5469(	gamma his	Cre17.g70	Histone H2	LOC_Os12	Core histor	putative	expressed	
76 GO:0019953		Cre03.g17	(1 of 11) PT	LOC_Os10	expansin p	putative	expressed	
76 GO:0019953		Cre03.g17	(1 of 11) PT	LOC_Os10	expansin precursor			
76 GO:0019953		Cre03.g17	(1 of 11) PTHR31867//PTHR31867			putative	expressed	
AT2G3571(	Nucleotide-diphospho-sugar trans			LOC_Os10	glycosyl tra	mitochonc	putative	expressed
AT5G3871(	Methylenet	Cre16.g64	Proline deh	LOC_Os10	proline oxi	mitochonc	putative	expressed
AT5G3871(	Methylenet	Cre16.g64	Proline deh	LOC_Os10	proline oxi	mitochonc	putative	expressed
AT5G3871(	Methylenet	Cre16.g64	Proline deh	LOC_Os10	proline oxi	putative	expressed	
AT3G5143(	Calcium-dependent phosphotries			LOC_Os10	strictosidine synthase			
						putative	expressed	
AT2G0218(	tobamoviru	Cre12.g48	(1 of 1) PF0	LOC_Os10	tobamoviru	putative	expressed	
AT2G0218(	tobamoviru	Cre12.g48	(1 of 1) PF0	LOC_Os10	tobamovirus multiplication protein			
72 GO:0004713 GO:00(		Cre07.g34	(1 of 2) PF00069//PF07714//PF13			putative	expressed	
AT1G1036(	glutathione	Cre11.g46	(1 of 15) 2.	LOC_Os10	glutathione	putative	expressed	
AT2G4572(	ARM repeat superfamily protein			LOC_Os10	armadillo	putative	expressed	
AT2G4572(	ARM repeat superfamily protein			LOC_Os10	armadillo	putative	expressed	
AT2G4572(	ARM repeat superfamily protein			LOC_Os10	armadillo	alpha/beta	putative	expressed
23				LOC_Os10	hydrolase	expressed		
AT5G0761(	F-box family protein			LOC_Os07	OsFBX238	expressed		
AT5G0761(	F-box family protein			LOC_Os07	OsFBX238 - F-box domain containing protein			
						putative	expressed	
15		Cre17.g73	(1 of 8) PF0	LOC_Os03	BRASSINO	expressed		
AT2G1461(	pathogene	Cre02.g08	Predicted e	LOC_Os10	SCP-like extracellular protein			
44 GO:0006886 GO:00		Cre10.g45	Mitochondrial inner membrane tra			putative	expressed	
AT1G6710(	LOB domain-containing protein 4(			LOC_Os01	DUF260 dc	expressed		
AT2G4431(	Calcium-binding EF-hand family p			LOC_Os03	EF hand fa	expressed		
AT1G2307(	Protein of unknown function (DUF			LOC_Os03	domain of u	expressed		
AT1G7829(	Protein kin	Cre10.g46(	conserved	LOC_Os03	CAMK_CAN	expressed		
AT1G7829(	Protein kin	Cre10.g46(	conserved	LOC_Os03	CAMK_CAMK_like.19 - CAMK includes calcium/calmoduli			
						putative	expressed	
24 GO:0005992 GO:00(		Cre12.g49	Trehalose 6	LOC_Os03	uncharacte	putative	expressed	
24 GO:0005992		Cre12.g49	Trehalose 6	LOC_Os03	uncharacte	putative	expressed	
AT1G7807(	Transducin	Cre09.g39	(1 of 1) PTH	LOC_Os03	WD-40 rep	putative	expressed	
AT1G7807(	Transducin	Cre09.g39	(1 of 1) PTH	LOC_Os03	WD-40 rep	putative	expressed	
AT1G7807(	Transducin	Cre09.g39	(1 of 1) PTH	LOC_Os03	WD-40 rep	putative	expressed	
AT1G0772(	3-ketoacyl-CoA synthase 3			LOC_Os03	3-ketoacyl-CoA synthase			
				LOC_Os03	expressed protein			
24		Cre05.g23	AAA ATPase			putative	expressed	

97 GO:0005506 GO:0016705 GO:0020037 GO				LOC_Os02	cytochrome P450				
AT2G4125(	Haloacid d	Cre09.g40:	Halo-acid dehalogenase-like hydr			putative	expressed		
AT3G6273(	(1 of 2) PF1	Cre01.g03:	(1 of 1) PF1	LOC_Os03	desiccatior	expressed			
54 GO:0006694 GO:00		Cre03.g16!	UDP-glucu	LOC_Os07	NAD deper	expressed			
54 GO:0006694 GO:00		Cre03.g16!	UDP-glucu	LOC_Os07	NAD deper	expressed			
15				LOC_Os03	DUF623 dc	expressed			
AT2G2849(	RmlC-like cupins superfamily prot			LOC_Os03	cupin domain containing protein				
				LOC_Os03	expressed	LOC_Os03	2	3-bisphosp	putative
24 GO:0004619 GO:00(		Cre06.g27:	Phosphogly	2	3-bisphosp	C3HC4 typ	expressed		
15 GO:0008270		Cre13.g58(	(1 of 1) K16	LOC_Os03	zinc finger	C3HC4 typ	expressed		
15 GO:0008270		Cre13.g58(	(1 of 1) K16	LOC_Os03	zinc finger	putative	expressed		
				LOC_Os03	chaperone	putative	expressed		
				LOC_Os03	chaperone	putative	expressed		
AT2G3664(	embryonic	Cre03.g208497		LOC_Os03	late embry	expressed			
				LOC_Os03	cytochrom	putative	expressed		
				LOC_Os03	VQ domain	putative	expressed		
AT2G4718(	galactinol synthase 1			LOC_Os03	glycosyl transferase 8 domain containing protein				
						expressed			
AT2G4739(	Prolyl oligo	Cre07.g31:	(1 of 2) 3.4	LOC_Os03	OsPOP7 - F	expressed			
AT2G4739(	Prolyl oligo	Cre07.g31:	(1 of 2) 3.4	LOC_Os03	OsPOP7 - F	putative	expressed		
AT4G1616(	Mitochondrial import inner memb			LOC_Os03	mitochond	putative	expressed		
AT4G1614(	cAMP-regu	Cre06.g30	(1 of 1) PTH	LOC_Os12	Lg106	putative	expressed		
AT1G5630(	Chaperone DnaJ-domain superfar			LOC_Os03	heat shock	putative	expressed		
AT1G5630(	Chaperone DnaJ-domain superfar			LOC_Os03	heat shock	putative	expressed		
AT1G5630(	Chaperone DnaJ-domain superfar			LOC_Os03	heat shock protein DnaJ				
						putative	expressed		
66 GO:0006529		Cre01.g00	Asparagine	LOC_Os03	asparagine	putative	expressed		
66 GO:0006529		Cre01.g00	Asparagine	LOC_Os03	asparagine	putative	expressed		
66 GO:0006529		Cre01.g00	Asparagine	LOC_Os03	asparagine	putative	expressed		
66 GO:0006529		Cre01.g00	Asparagine	LOC_Os03	asparagine	putative	expressed		
66 GO:0006529		Cre01.g00	Asparagine	LOC_Os03	asparagine synthetase				
				LOC_Os03	expressed	putative	expressed		
AT5G0279(	Glutathione S-transferase family p			LOC_Os03	IN2-1 prote	putative	expressed		
AT2G3947(	PsbP-like protein 2			LOC_Os03	PsbP	putative	expressed		
		Cre08.g37:	Heat shock	LOC_Os03	DnaK family protein				
38		Cre09.g39	(1 of 1) K09	LOC_Os03	expressed protein				
AT5G0584(	Protein of unknown function (DUF			LOC_Os03	expressed protein				
AT3G4623(	heat shock	Cre07.g31	Heat shock protein 22H						
AT4G3730(	maternal effect embryo arrest 59			LOC_Os03	expressed	expressed			
AT1G0735(	RNA-binding (RRM/RBD/RNP moti			LOC_Os03	RNA recogr	expressed			
AT1G0735(	RNA-binding (RRM/RBD/RNP moti			LOC_Os03	RNA recogr	expressed			

AT1G0735(	RNA-binding (RRM/RBD/RNP moti			LOC_Os03	RNA recogr	putative	expressed	
55				LOC_Os03	plastocyan	putative	expressed	
AT1G7562(	glyoxal oxic	Cre11.g46	Glyoxal oxi	LOC_Os03	glyoxal oxic	putative	expressed	
AT4G3881(	Calcium-binding EF-hand family p			LOC_Os03	calcium-bi	putative	expressed	
AT4G3881(	Calcium-binding EF-hand family p			LOC_Os03	calcium-bi	putative	expressed	
AT4G3881(	Calcium-binding EF-hand family p			LOC_Os03	calcium-bi	putative	expressed	
AT4G3881(	Calcium-binding EF-hand family p			LOC_Os03	calcium-bi	putative	expressed	
AT4G3881(	Calcium-binding EF-hand family p			LOC_Os03	calcium-bi	glycosyl hy	putative	expressed
AT5G4210(	beta-1	3-glucanase_putative			LOC_Os03	putative	expressed	
AT4G2767(	heat shock protein 21			LOC_Os03	hsp20/alph	putative	expressed	
AT2G2105(	like AUXIN RESISTANT 2			LOC_Os03	transmembrane amino acid transporter protein			
AT3G5582(	Fasciclin-like arabinogalactan fam			LOC_Os03	expressed protein			
AT2G41950				LOC_Os03	expressed protein			
				LOC_Os03	expressed protein			
				LOC_Os03	expressed protein			
				LOC_Os03	expressed	putative	expressed	
50				LOC_Os03	ATP8	putative	expressed	
50				LOC_Os03	ATP8	putative	expressed	
AT2G2379(	Protein of u	Cre17.g72	(1 of 1) PF(	LOC_Os03	E2F-related protein			
21 GO:0055085								
21 GO:0055085						putative	expressed	
AT2G3212(	heat-shock protein 70T-2			LOC_Os03	DnaK famil	expressed		
AT1G2656(	beta glucos	Cre07.g32(	(1 of 2) 3.2	LOC_Os03	Os3bglu6 -	expressed		
AT1G2656(	beta glucos	Cre07.g32(	(1 of 2) 3.2	LOC_Os03	Os3bglu6 -	expressed		
AT4G3674(	homeobox protein 40			LOC_Os03	homeobox	expressed		
AT2G1854(	RmlC-like cupins superfamily prot			LOC_Os03	cupin doma	auxin-resp(	putative	expressed
AT2G0485(	Auxin-resp	Cre03.g20(	(1 of 1) PTH	ISOFORM /	LOC_Os03g09850			
AT5G1370(	polyamine oxidase 1							
AT5G1370(	polyamine oxidase 1					putative	expressed	
AT2G2224(	myo-inosit	Cre03.g18(	Myo-inosit	LOC_Os03	inositol-3-p	putative	expressed	
AT2G2224(	myo-inosit	Cre03.g18(	Myo-inosit	LOC_Os03	inositol-3-p	putative	expressed	
AT2G2224(	myo-inosit	Cre03.g18(	Myo-inosit	LOC_Os03	inositol-3-p	putative	expressed	
AT2G2224(	myo-inosit	Cre03.g18(	Myo-inosit	LOC_Os03	inositol-3-p	putative	expressed	
				LOC_Os03	hydrolase p	putative	expressed	
				LOC_Os03	hydrolase protein			
77 GO:0003700 GO:00(		Cre01.g00	basal body protein			putative	expressed	
AT1G7282(	Mitochondrial substrate carrier fan			LOC_Os03	mitochond	putative	expressed	
AT1G7282(	Mitochondrial substrate carrier fan			LOC_Os03	mitochond	putative	expressed	
				LOC_Os03	embryonic	expressed		
15				LOC_Os03	tetratricopeptide repeat domain containing protein			
AT4G1910(	Protein of unknown function (DUF			LOC_Os06	expressed	putative	expressed	
				LOC_Os03	late embry	putative	expressed	
				LOC_Os03	late embryogenesis abundant protein D-34			

AT2G2172(	Plant protein of unknown function			LOC_Os03	expressed protein				
AT2G2172(	Plant protein of unknown function			LOC_Os03	expressed protein				
AT2G2172(	Plant protein of unknown function			LOC_Os03	expressed	putative	expressed		
AT2G1680(	high-affinit	Cre14.g62!	nickel trans	LOC_Os03	high-affinit	putative	expressed		
AT2G1680(	high-affinit	Cre14.g62!	nickel trans	LOC_Os03	high-affinity nickel-transport family protein				
AT1G0857(	atypical CY	Cre06.g26!	Thioredoxir	chloroplastic					
AT1G0857(	atypical CY	Cre06.g26!	Thioredoxir	chloroplastic		putative	expressed		
20 GO:0016021 GO:0022857				LOC_Os03	nodulin	putative	expressed		
AT2G3683(	gamma tor	Cre12.g54!	Aquaporin	LOC_Os03	aquaporin protein				
AT5G2292(	CHY-type/(	Cre17.g71!	(1 of 1) K1C	PIRH2)					
AT1G0453(	Tetratricopeptide repeat (TPR)-like			LOC_Os03	expressed	Cytochrom	CYP197 su	LOC_Os03	cytochrom
AT4G3949(	cytochrom	family 96	subfamily	polypeptid	Cre01.g003850				
				LOC_Os03	expressed	putative	expressed		
15		Cre13.g58	(1 of 15) 2.	LOC_Os03	glutathione	expressed			
58 GO:0005975 GO:0006032 GO:0016998				LOC_Os03	CHIT16 - Chitinase family protein precursor				
21 GO:0055085		Cre16.g68(	(1 of 1) PTHR31419:SF1 - AUXIN E			putative	expressed		
AT1G3299(	plastid ribc	Cre10.g42:	Chloroplas	LOC_Os03	L11 domain	putative	expressed		
AT5G5535(	MBOAT (m	Cre07.g35(	(1 of 8) 2.3	LOC_Os02	O-acyltrans	putative	expressed		
01 GO:0006979 GO:0020037 GO:0055114				LOC_Os03	peroxidase precursor				
15 GO:0006810 GO:00:		Cre12.g54!	Aquaporin			putative	expressed		
AT3G1768(	Kinase interacting (KIP1-like) fami			LOC_Os03	KIP1	putative	expressed		
39 GO:0012511 GO:0016021				LOC_Os09	oleosin	expressed			
72 GO:0004713 GO:00(		Cre02.g09!	(1 of 1) PTH	LOC_Os03	protein kina	expressed			
72 GO:0004713 GO:00(		Cre02.g09!	(1 of 1) PTH	LOC_Os03	protein kina	expressed			
72 GO:0004713 GO:00(		Cre02.g09!	(1 of 1) PTH	LOC_Os03	protein kina	expressed			
72 GO:0004713 GO:00(		Cre02.g09!	(1 of 1) PTH	LOC_Os03	protein kina	expressed			
AT1G6912(	K-box region and MADS-box transc			LOC_Os07	OsMADS15	casein kina	putative	expressed	
AT3G5000(	casein kina	alpha chai	Cre16.g66!	Casein kina	LOC_Os07	putative	expressed		
AT5G4963(	amino acid permease 6			LOC_Os07	amino acid	putative	expressed		
AT1G3013!	jasmonate-zim-domain protein 8			LOC_Os07	zinc-finger	putative	expressed		
AT5G0657(	alpha/beta-Hydrolases superfami			LOC_Os07	gibberellin receptor GID1L2				
77 GO:0008270									
77 GO:0008270									
				LOC_Os07	expressed protein				
				LOC_Os07	expressed	putative	expressed		
99 GO:0050660 GO:00!		Cre03.g16!	Flavin-cont	LOC_Os04	flavin-cont	early light-i	chloroplas	putative	expressed
AT3G2284(	Chlorophyl	Cre08.g38	(1 of 6) PTH	CHLOROP	LOC_Os01g14410				
AT5G2877(	bZIP transcription factor family protein					putative	expressed		
53 GO:0005975				LOC_Os06	glycosyl hy	putative	expressed		
AT5G0379!	Exostosin family protein			LOC_Os07	exostosin family protein				
10 GO:0016021 GO:00:		Cre04.g22!	R-SNARE	VAMP72-family					
10 GO:0016021 GO:00:		Cre04.g22!	R-SNARE	VAMP72-family					
10 GO:0016021 GO:00:		Cre04.g22!	R-SNARE	VAMP72-family					

10 GO:0016021 GO:00:		Cre04.g22!	R-SNARE	VAMP72-family				
21 GO:0016192		Cre04.g22!	R-SNARE	VAMP72-family		putative	expressed	
77 GO:0006355				LOC_Os07	NAC doma	putative	expressed	
AT3G0772(	Galactose (	Cre03.g14(	(1 of 1) PTH	LOC_Os09	kelch repeat protein			
				LOC_Os10	expressed protein			
48 GO:0030598								
48 GO:0030598						putative	expressed	
				LOC_Os12	Jacalin-like lectin domain containing protein			
						putative	expressed	
AT5G2096(	aldehyde oxidase 1			LOC_Os07	aldehyde o	putative	expressed	
AT5G2096(	aldehyde oxidase 1			LOC_Os07	aldehyde o	putative	expressed	
AT5G2096(	aldehyde oxidase 1			LOC_Os07	aldehyde o	putative	expressed	
AT5G2096(	aldehyde oxidase 1			LOC_Os07	aldehyde o	putative	expressed	
AT5G2096(	aldehyde oxidase 1			LOC_Os07	aldehyde o	putative	expressed	
AT5G2096(	aldehyde oxidase 1			LOC_Os07	aldehyde oxidase			
AT4G2289(	PGR5-LIKE	Cre07.g34(	Proton-gra(	LOC_Os08	expressed protein			
AT4G2289(	PGR5-LIKE	Cre07.g34(	Proton-gra(	LOC_Os08	expressed	putative	expressed	
AT1G0456(	AWPM-19-like family protein			LOC_Os07	AWPM-19-l	putative	expressed	
AT1G0456(	AWPM-19-like family protein			LOC_Os07	AWPM-19-l	putative	expressed	
AT1G0456(	AWPM-19-like family protein			LOC_Os07	AWPM-19-l	putative	expressed	
AT1G0456(	AWPM-19-like family protein			LOC_Os07	AWPM-19-l	putative	expressed	
AT1G0457(	Major facilitator superfamily prote			LOC_Os07	integral membrane transporter family protein			
						putative	expressed	
97 GO:0005506 GO:0016705 GO:0020037 GO				LOC_Os05	cytochrome P450			
				LOC_Os09	expressed	putative	expressed	
AT1G0817(	Histone superfamily protein			LOC_Os09	Core histor	putative	expressed	
AT5G2395(	Calcium-dependent lipid-binding (			LOC_Os08	C2 domain	putative	expressed	
AT4G3992(	C-CAP/cofa	Cre03.g20(	(1 of 1) PTH	LOC_Os02	tubulin binding cofactor C			
AT4G31830				LOC_Os09	expressed protein			
						chlorophyll	putative	expressed
AT1G7657(	Chlorophyl	Cre02.g11(	Light-harve	chloroplas	LOC_Os09	putative	expressed	
72 GO:0004713 GO:0005515 GO:0005524 GO				LOC_Os09	senescenc	putative	expressed	
72 GO:0004713 GO:0005515 GO:0005524 GO				LOC_Os09	senescence-induced receptor-like serine/threonine-prote			
35 GO:0005515 GO:0005840 GO:0006412						putative	expressed	
24 GO:0006633 GO:0008152 GO:0008610 GO				LOC_Os09	3-ketoacyl-	putative	expressed	
AT5G6514(	Haloacid d	Cre12.g49	Trehalose 6	LOC_Os09	uncharacterized glycosyl hydrolase Rv2006/MT2062			
				LOC_Os09	expressed protein			
				LOC_Os09	expressed protein			
				LOC_Os09	expressed	expressed		
AT5G6516!	succinate (	Cre06.g26	Iron-sulfur	LOC_Os09	succinate dehydrogenase and fumarate reductase iron-su			
AT1G6001(	(1 of 28) PF14009 - Domain of unk			LOC_Os09	expressed	putative	expressed	
AT5G1694(	carbon-sulfur lyases			LOC_Os09	proline-rich	expressed		
AT4G3180(	WRKY DNA-binding protein 18			LOC_Os09	WRKY76	expressed		
AT4G3180(	WRKY DNA-binding protein 18			LOC_Os09	WRKY76	classes I a	domain co	expressed

24 GO:0009058 GO:00:		Cre06.g28	Alanine am	LOC_Os09	aminotrans	classes I a	domain co	expressed
24 GO:0009058 GO:00:		Cre06.g28	Alanine am	LOC_Os09	aminotransferase			
35						expressed		
AT1G5391(	related to AP2 12			LOC_Os09	AP2 domai	expressed		
AT1G5391(	related to AP2 12			LOC_Os09	AP2 domai	expressed		
AT1G5391(	related to AP2 12			LOC_Os09	AP2 domai	putative	expressed	
97 GO:0005506 GO:0016705 GO:0020037 GO				LOC_Os09	cytochrome P450			
				LOC_Os02	expressed	putative	expressed	
AT2G0191(	Microtubul	Cre14.g61	(1 of 1) K16	LOC_Os09	microtubul	putative	expressed	
AT2G0191(	Microtubul	Cre14.g61	(1 of 1) K16	LOC_Os09	microtubul	putative	expressed	
AT2G0191(	Microtubul	Cre14.g61	(1 of 1) K16	LOC_Os09	microtubul	expressed		
00 GO:0005634 GO:00(		Cre09.g38	Heat shock	LOC_Os09	HSF-type D	putative	expressed	
				LOC_Os09	gibberellin	putative	expressed	
				LOC_Os09	gibberellin	putative	expressed	
AT1G4965(	alpha/beta-Hydrolases superfami			LOC_Os09	gibberellin	RNA recogr	putative	expressed
AT1G4749(	RNA-bindir	Cre16.g65	(1 of 16) K(	subunit 4	LOC_Os09	RNA recogr	putative	expressed
AT1G4749(	RNA-bindir	Cre16.g65	(1 of 16) K(	subunit 4	LOC_Os09	expressed		
AT4G2466(	homeobox protein 22			LOC_Os09	ZF-HD prot	putative	unclassifie	expressed
AT5G2122	protein kinase family protein			LOC_Os01	retrotransp	putative	unclassifie	expressed
AT5G2122	protein kinase family protein			LOC_Os01	retrotransp	putative	unclassifie	expressed
AT5G2122	protein kinase family protein			LOC_Os01	retrotransp	putative	expressed	
02 GO:0055114				LOC_Os09	2-aminoeth	putative	expressed	
AT2G2242(	Peroxidase superfamily protein			LOC_Os09	peroxidase	putative	expressed	
AT2G2242(	Peroxidase superfamily protein			LOC_Os09	peroxidase	putative	expressed	
AT2G2242(	Peroxidase superfamily protein			LOC_Os09	peroxidase	putative	expressed	
AT2G2242(	Peroxidase superfamily protein			LOC_Os09	peroxidase	putative	expressed	
AT2G2242(	Peroxidase superfamily protein			LOC_Os09	peroxidase	putative	expressed	
24 GO:0016020 GO:00:		Cre02.g09	(1 of 13) 3.	LOC_Os09	white-brow	putative	expressed	
19				LOC_Os07	male sterili	putative	expressed	
AT4G2654(	Leucine-ric	Cre12.g50	(1 of 6) PF(	LOC_Os09	receptor-like protein kinase 2 precursor			
AT2G0424(	RING/U-box superfamily protein					putative	expressed	
71				LOC_Os09	caffeoyl-CoA O-methyltransferase			
				LOC_Os09	expressed	splicing fac	putative	expressed
66 GO:0003676 GO:00(		Cre06.g25	U2 snRNP a	small subu	LOC_Os09	splicing fac	putative	expressed
66 GO:0003676 GO:00(		Cre06.g25	U2 snRNP a	small subu	LOC_Os09	splicing fac	putative	expressed
66 GO:0003676 GO:00(		Cre06.g25	U2 snRNP a	small subu	LOC_Os09	splicing fac	putative	expressed
66 GO:0003676 GO:00(		Cre06.g25	U2 snRNP a	small subu	LOC_Os09	splicing fac	putative	expressed
66 GO:0003676 GO:00(		Cre06.g25	U2 snRNP a	small subu	LOC_Os09	splicing fac	putative	expressed
66 GO:0003676 GO:00(		Cre06.g25	U2 snRNP a	small subu	LOC_Os09	expressed		
AT1G0585(	Chitinase family protein			LOC_Os09	CHIT13 - C	expressed		
AT1G0585(	Chitinase family protein			LOC_Os09	CHIT13 - Chitinase family protein precursor			
				LOC_Os09	expressed	expressed protein		
AT4G3133(	Protein of u	DUF599			LOC_Os09	expressed protein		
AT4G3133(	Protein of u	DUF599			LOC_Os09g32100			

				LOC_Os09	expressed	3-beta-gluc	putative	expressed
AT3G0732(	O-Glycosyl hydrolases family 17 p			LOC_Os09	glucan end	putative	expressed	
				LOC_Os09	formin	putative	expressed	
60 GO:0005975				LOC_Os09	glycosyl hy	expressed		
55 GO:0015238 GO:0015297 GO:0016020 GO				LOC_Os09	MATE doma	expressed		
AT4G1166(	winged-helix DNA-binding transcri			LOC_Os09	HSF-type D	putative	expressed	
AT3G6188(	cytochrome p450 78a9			LOC_Os09	cytochrome P450			
				LOC_Os09	expressed	putative	expressed	
24 GO:0006596		Cre16.g68	Ornithine d	LOC_Os09	pyridoxal-dependent decarboxylase protein			
				LOC_Os09	expressed	putative	expressed	
AT2G2422(	purine permease 5			LOC_Os09	purine perr	putative	expressed	
AT2G3687(	xyloglucan endotransglucosylase/			LOC_Os07	glycosyl hy	putative	expressed	
AT3G5494(	Papain family cysteine protease			LOC_Os07	cysteine proteinase A494 precursor			
AT3G2180(	UDP-gluco	Cre07.g32	(1 of 1) PTHR11926//PTHR11926:SF16 - GLUCOSYL/GLUCURONOSYL TRANSFE					
52		Cre01.g04	Aconitate hydratase					
52		Cre01.g04	Aconitate hydratase			putative	expressed	
15 GO:0006810 GO:00		Cre16.g66	Conserved	LOC_Os07	transporter family protein			
60 GO:0005515								
60 GO:0005515						3-beta-gluc	putative	expressed
53 GO:0005975				LOC_Os07	glucan end	3-beta-gluc	putative	expressed
53 GO:0005975				LOC_Os07	glucan endo-1			
AT2G3228(	Protein of unknown function (DUF			LOC_Os07	expressed protein			
AT1G11120				LOC_Os07	expressed	expressed		
52 GO:0006508		Cre05.g24	Subtilisin-l	LOC_Os07	OsSub53 - Putative Subtilisin homologue			
AT1G27461				LOC_Os07	expressed protein			
01 GO:0006979 GO:0020037 GO:0055114								
				LOC_Os07	expressed	putative	expressed	
AT5G6742(	LOB domain-containing protein 37			LOC_Os07	DUF260 dc	putative	expressed	
57 GO:0019290 GO:0055114				LOC_Os07	sex determination protein tasselseed-2			
AT5G4307(	WPP domain protein 1					expressed		
AT1G1865(	plasmodesmata callose-binding p			LOC_Os07	X8 domain	putative	expressed	
				LOC_Os07	gibberellin	putative	expressed	
01 GO:0046872				LOC_Os07	proline-rich	putative	expressed	
AT5G5559(	Pectin lyase-like superfamily prote			LOC_Os07	pectinester	putative	expressed	
AT5G5559(	Pectin lyase-like superfamily prote			LOC_Os07	pectinester	putative	expressed	
72 GO:0004713 GO:00(		Cre12.g51	(1 of 1) K04	LOC_Os07	tyrosine protein kinase domain containing protein			
AT5G2128(	hydroxyproline-rich glycoprotein family protein					putative	expressed	
AT3G1413(	Aldolase-ty	Cre03.g17	Glycolate c	LOC_Os07	hydroxyaci	putative	expressed	
AT3G1413(	Aldolase-ty	Cre03.g17	Glycolate c	LOC_Os07	hydroxyaci	putative	expressed	
AT3G1413(	Aldolase-ty	Cre03.g17	Glycolate c	LOC_Os07	hydroxyaci	putative		
09				LOC_Os03	EF hand fai	putative	expressed	
AT4G3995	alpha/beta-Hydrolases superfami			LOC_Os03	hydrolase protein			
AT1G3578(	(1 of 4) PTHR31132:SF2 - EXPRES			LOC_Os07	expressed protein			

AT1G3578(	(1 of 4) PTHR31132:SF2 - EXPRES:			LOC_Os07	expressed protein		
AT1G3578(	(1 of 4) PTHR31132:SF2 - EXPRES:			LOC_Os07	expressed protein		
AT1G1677(	(1 of 1) PTH	Cre07.g32!	(1 of 1) PTH	LOC_Os02	expressed protein		
				LOC_Os07	expressed protein		
				LOC_Os07	expressed protein		
				LOC_Os07	expressed	putative	expressed
AT3G4760(	myb domai	Cre06.g28!	Hua Enhan	LOC_Os07	MYB family	C3HC4 typ	expressed
15 GO:0008270				LOC_Os07	zinc finger	putative	expressed
AT1G4813(	1-cysteine peroxiredoxin 1			LOC_Os07	peroxiredo:	putative	expressed
AT1G4813(	1-cysteine peroxiredoxin 1			LOC_Os07	peroxiredo:	putative	expressed
AT1G4813(	1-cysteine peroxiredoxin 1			LOC_Os07	peroxiredo:	putative	expressed
AT1G4813(	1-cysteine peroxiredoxin 1			LOC_Os07	peroxiredo:	putative	expressed
AT5G5060(	hydroxysteroid dehydrogenase 1			LOC_Os12	dehydroge	expressed	
)0 GO:0006355 GO:0043565				LOC_Os07	bZIP transc	putative	expressed
AT2G2842(	Lactoylglutathione lyase / glyoxala			LOC_Os07	glyoxalase	putative	expressed
22 GO:0008152 GO:0008667 GO:0016491 GO				LOC_Os07	sex determ	putative	expressed
				LOC_Os07	stress-rela	putative	expressed
52 GO:0016758		Cre07.g32:	(1 of 1) PTH	LOC_Os07	glucosyltransferase		
AT1G02700				LOC_Os07	expressed	expressed	
				LOC_Os07	DUF623 dc	putative	expressed
77 GO:0006355				LOC_Os07	no apical m	putative	expressed
AT5G1827(	Arabidopsis NAC domain containi			LOC_Os07	no apical m	expressed	
AT3G0366(	WUSCHEL related homeobox 11			LOC_Os07	homeobox	putative	expressed
58 GO:0051716				LOC_Os01	salt stress	putative	expressed
58 GO:0051716				LOC_Os01	salt stress	putative	expressed
58 GO:0051716				LOC_Os01	salt stress root protein RS1		
AT3G4828(	cytochrom	family 71	subfamily ,	polypeptide 25			
AT1G64080				LOC_Os01	expressed	putative	expressed
59				LOC_Os01	late embryo	putative	expressed
38		Cre14.g61!	(1 of 5) 2.3	LOC_Os01	GDSL-like l	expressed	
				LOC_Os01	OsGH3.3 -	expressed	
				LOC_Os01	OsGH3.3 -	putative	expressed
				LOC_Os01	DJ-1 family	putative	expressed
AT3G1492(	Peptide-N4-(N-acetyl-beta-glucos			LOC_Os01	peptide-N4	putative	expressed
)1 GO:0006979 GO:0020037 GO:0055114				LOC_Os01	peroxidase	expressed	
AT5G1193(	Thioredoxin superfamily protein			LOC_Os01	OsGrx_A2 -	putative	expressed
AT2G1981(	CCCH-type	Cre13.g56(	possible tra	LOC_Os01	zinc finger/	putative	expressed
AT4G3778(	myb domai	Cre06.g28!	Hua Enhan	LOC_Os01	myb-like DNA-binding domain containing protein		
AT3G2136(	2-oxoglutarate (2OG) and Fe(II)-de			LOC_Os01	expressed	putative	expressed
AT5G0361(	GDSL-like l	Cre14.g61!	(1 of 5) 2.3	LOC_Os09	GDSL-like lipase/acylhydrolase		
29		Cre09.g39:	(1 of 1) 3.1.1.32 - Phospholipase A			putative	expressed
AT5G1202(	17.6 kDa class II heat shock protei			LOC_Os01	hsp20/alpha crystallin family protein		

46						putative	expressed		
AT5G4039(	Raffinose synthase family protein			LOC_Os01	uncharacte	expressed			
35 GO:0006508		Cre09.g41	Serine carb	LOC_Os01	OsSCP1 - F	putative	expressed		
AT2G3711(	PLAC8 family protein			LOC_Os01	uncharacterized Cys-rich domain containing protein				
						putative	expressed		
50		Cre16.g64	Universal s	LOC_Os07	universal s	putative	expressed		
50		Cre16.g64	Universal s	LOC_Os07	universal s	putative	expressed		
50		Cre16.g64	Universal s	LOC_Os07	universal s	putative	expressed		
50		Cre16.g64	Universal s	LOC_Os07	universal stress protein domain containing protein				
				LOC_Os01	expressed	putative	expressed		
AT3G4623(	heat shock	Cre07.g31	Heat shock	LOC_Os01	hsp20/alph	putative	expressed		
AT3G4623(	heat shock	Cre07.g31	Heat shock	LOC_Os01	hsp20/alph	putative	expressed		
		Cre07.g31	Heat shock	LOC_Os01	hsp20/alph	putative	expressed		
		Cre07.g31	Heat shock	LOC_Os01	hsp20/alpha crystallin family protein				
				LOC_Os01	expressed protein				
						expressed			
AT1G1810(	PEBP (phosphatidylethanolamine-			LOC_Os01	osMFT2 MF	putative	expressed		
AT1G6878(	RNI-like superfamily protein			LOC_Os01	TOO MANY	putative	expressed		
AT5G1504(	Paired amphipathic helix (PAH2) s			LOC_Os08	paired amp	putative	expressed		
AT4G3923(	NmrA-like	Cre11.g47	Putative 2'-	LOC_Os01	isoflavone	putative	expressed		
AT2G3727(	ribosomal	Cre06.g29(	Cytosolic 8	LOC_Os01	40S ribosomal protein S5				
AT5G5639(	F-box/RNI-like/FBD-like domains-containing protein								
AT5G5639(	F-box/RNI-like/FBD-like domains-containing protein					putative	expressed		
AT3G1249(	cystatin B	Cre04.g22	(1 of 1) PTH	LOC_Os01	cysteine proteinase inhibitor precursor protein				
01 GO:0006979 GO:0020037 GO:0055114									
						putative	expressed		
				LOC_Os01	embryonic	putative	expressed		
AT5G21070				LOC_Os01	glycosyl hydrolases family 17 protein				
						expressed			
AT3G1761	RHOMBOID	Cre03.g14	Rhomboid-	LOC_Os01	OsRhmbd3	LOC_Os01	MDR-like A	putative	expressed
AT3G6215(	P-glycopro	Cre17.g72	(1 of 2) K05	subfamily	member 1	LOC_Os01	MDR-like A	putative	expressed
AT3G6215(	P-glycopro	Cre17.g72	(1 of 2) K05	subfamily	member 1	LOC_Os01	MDR-like A	putative	expressed
AT3G6215(	P-glycopro	Cre17.g72	(1 of 2) K05	subfamily	member 1	LOC_Os01	MDR-like A	putative	expressed
AT3G6215(	P-glycopro	Cre17.g72	(1 of 2) K05	subfamily	member 1	LOC_Os01	MDR-like A	putative	expressed
AT3G6215(	P-glycopro	Cre17.g72	(1 of 2) K05	subfamily	member 1	LOC_Os01	MDR-like A	putative	expressed
AT3G6215(	P-glycopro	Cre17.g72	(1 of 2) K05	subfamily	member 1	LOC_Os01	MDR-like A	putative	expressed
AT3G6215(	P-glycopro	Cre17.g72	(1 of 2) K05	subfamily	member 1	LOC_Os01	MDR-like A	putative	expressed
AT3G6215(	P-glycopro	Cre17.g72	(1 of 2) K05	subfamily	member 1	putative	expressed		
				LOC_Os01	mitochond	putative	expressed		
				LOC_Os01	mitochondrial import inner membrane translocase subun				
AT4G1614(	cAMP-regu	Cre06.g30	(1 of 1) PTH	LOC_Os01	expressed protein				
AT4G1614(	cAMP-regu	Cre06.g30	(1 of 1) PTH	LOC_Os01	expressed	putative	expressed		
AT5G4933(	myb domai	Cre06.g28	Hua Enhan	LOC_Os01	MYB family	putative	expressed		

				LOC_Os01	anther-spe	expressed			
AT3G1677(	ethylene-responsive element bind			LOC_Os01	AP2 domain containing protein				
56 GO:0003676		Cre01.g02(	Serine/arginine-rich pre-mRNA splicing factor						
56 GO:0003676		Cre01.g02(	Serine/arginine-rich pre-mRNA splicing factor						
56 GO:0003676		Cre01.g02(	Serine/arginine-rich pre-mRNA splicing factor						
56 GO:0003676		Cre01.g02(	Serine/arginine-rich pre-mRNA splicing factor						
56 GO:0003676		Cre01.g02(	Serine/arginine-rich pre-mRNA spl			putative	expressed		
01 GO:0006979 GO:0020037 GO:0055114				LOC_Os01	peroxidase precursor				
15						putative	expressed		
				LOC_Os01	stripe rust resistance protein Yr10				
AT2G25920						putative	CACTA	En/Spm su	expressed
08 GO:0008234				LOC_Os04	transposor	putative	CACTA	En/Spm su	expressed
				LOC_Os04	transposor	expressed			
AT4G1542(	Ubiquitin fusion degradation UFD1			LOC_Os01	ZOS1-09 - C2H2 zinc finger protein				
				LOC_Os01	expressed	expressed			
00 GO:0005634 GO:0006355 GO:0043565				LOC_Os01	HSF-type D	putative	expressed		
AT1G0130(	Eukaryotic aspartyl protease famil			LOC_Os01	aspartic proteinase				
				LOC_Os01	expressed protein		LOC_Os01	cytochrom	putative
AT3G1469(	cytochrom	family 72	subfamily	polypeptide 15					
AT5G66440						putative	expressed		
97 GO:0005506 GO:0016705 GO:0020037 GO				LOC_Os02	cytochrom	putative	expressed		
97 GO:0005506 GO:0016705 GO:0020037 GO				LOC_Os02	cytochrom	alpha/beta	expressed		
AT2G1836(	alpha/beta-Hydrolases superfami			LOC_Os01	hydrolase	putative	expressed		
11 GO:0016021				LOC_Os01	oleosin	putative	expressed		
AT1G1550(	TLC ATP/AI	Cre08.g35	Plastidic AI	LOC_Os01	plastidic AT	putative	expressed		
24 GO:0050662		Cre11.g47	Putative 2'-	LOC_Os01	isoflavone	dehydroge	putative	expressed	
AT1G6593(	cytosolic N	Cre04.g21	Isocitrate d	NADP-dep	LOC_Os01	dehydroge	putative	expressed	
AT1G6593(	cytosolic N	Cre04.g21	Isocitrate d	NADP-dep	LOC_Os01	expressed			
AT5G6646(	Glycosyl hy	Cre01.g04	conserved	LOC_Os01	OsMan01 -	putative	expressed		
24 GO:0005525 GO:0005622 GO:0006886 GO				LOC_Os01	ras-related	auxin-resp	putative	expressed	
AT3G6175(	Cytochrom	Cre03.g20(	(1 of 1) PTH	ISOFORM	LOC_Os01	putative	expressed		
AT1G1036(	glutathione	Cre11.g46	(1 of 15) 2.	LOC_Os01	glutathione S-transferase				
AT2G2149(	dehydrin LEA					group 3	putative	expressed	
AT3G1567(	Late embryogenesis abundant pro			LOC_Os01	late embryo	group 3	putative	expressed	
AT3G1567(	Late embryogenesis abundant pro			LOC_Os01	late embryo	putative	expressed		
AT1G3404(	Pyridoxal phosphate (PLP)-depend			LOC_Os01	alliin lyase	expressed			
				LOC_Os01	DUF581 dc	expressed			
				LOC_Os01	DUF581 dc	putative	unclassifie	expressed	
AT2G4284(	protodermal factor 1			LOC_Os01	retrotransp	putative	expressed		
AT5G4569(	Protein of unknown function (DUF			LOC_Os01	DUF1264 c	putative	expressed		
AT5G4569(	Protein of unknown function (DUF			LOC_Os01	DUF1264 domain containing protein				
24				LOC_Os01	expressed	expressed			

AT3G2452(	heat shock transcription factor C1			LOC_Os01	HSF-type D	putative	expressed		
70 GO:0004471 GO:00(		Cre06.g25:	NADP-depe	LOC_Os01	NADP-depe	putative	expressed		
				LOC_Os01	DUF584 dc	putative	expressed		
AT5G4569(	Protein of unknown function (DUF			LOC_Os01	DUF1264 c	putative	expressed		
77 GO:0003700 GO:0006355				LOC_Os01	ethylene-re	putative	expressed		
AT5G4602(	(1 of 1) PTHR22055 - 28 KDA HEAT			LOC_Os01	heat- and acid-stable phosphoprotein				
AT4G1869(	(1 of 4) PTHR22952:SF210 - DOG1 ALPHA SPLICE VARIAN					expressed			
72 GO:0004713 GO:0005524 GO:0006468 GO				LOC_Os01	CAMK_KIN1/SNF1/Nim1_like.1 - CAMK includes calcium/				
AT5G64510				LOC_Os01	expressed	putative	expressed		
AT5G5339(	O-acyltransferase (WSD1-like) far			LOC_Os01	wax syntha	putative	expressed		
37 GO:0008270 GO:00(		Cre10.g45!	(1 of 1) 2.1	LOC_Os01	homocyste	putative	expressed		
		Cre10.g45!	(1 of 1) 2.1	LOC_Os01	homocyste	chloroplas	putative	expressed	
AT2G3057(	photosystem II reaction center W			LOC_Os01	photosyste	chloroplas	putative	expressed	
AT2G3057(	photosystem II reaction center W			LOC_Os01	photosyste	expressed			
AT5G4363(	zinc knuckle (CCHC-type) family p			LOC_Os01	plus-3 dom	expressed			
AT5G4363(	zinc knuckle (CCHC-type) family p			LOC_Os01	plus-3 dom	putative	expressed		
55		Cre14.g62!	(1 of 1) PF(	LOC_Os01	early nodul	putative	expressed		
AT1G6174(	Sulfite exporter TauE/SafE family p			LOC_Os01	membrane	putative	expressed		
AT1G6174(	Sulfite exporter TauE/SafE family p			LOC_Os01	membrane protein				
22 GO:0006810		Cre07.g31(	Similar to Nuclear Transport Facto			KH domain	putative	expressed	
AT3G1323(	RNA-bindir	Cre12.g54!	(1 of 1) K11	DIM2)	LOC_Os03	expressed			
AT1G2837(	ERF domain protein 11			LOC_Os01	AP2 domain containing protein				
AT5G5390(	Serine/threonine-protein kinase WNK (With No Lysine)-re					putative	expressed		
AT3G6306(	EID1-like 3			LOC_Os01	circadian c	putative	expressed		
AT1G6953(	expansin A	Cre17.g72:	(1 of 11) PT	LOC_Os01	expansin p	putative	expressed		
AT3G5620(	Transmeml	Cre02.g14!	Amino acid	LOC_Os01	transmeml	putative	expressed		
AT3G5620(	Transmeml	Cre02.g14!	Amino acid	LOC_Os01	transmeml	putative	expressed		
AT3G5620(	Transmeml	Cre02.g14!	Amino acid	LOC_Os01	transmembrane amino acid transporter protein				
AT4G08330						putative	expressed		
AT2G2879(	Pathogene:	Cre02.g10:	(1 of 1) PTH	LOC_Os01	thaumatin				
AT3G1258(	heat shock	Cre08.g37:	Heat shock protein 70A						
AT3G1258(	heat shock	Cre08.g37:	Heat shock protein 70A						
AT3G1258(	heat shock	Cre08.g37:	Heat shock protein 70A			putative	expressed		
AT2G2938(	highly ABA-induced PP2C gene 3			LOC_Os01	protein phc	putative	expressed		
AT2G2938(	highly ABA-induced PP2C gene 3			LOC_Os01	protein phc	putative	expressed		
AT2G2938(	highly ABA-induced PP2C gene 3			LOC_Os01	protein phc	putative	expressed		
AT2G2938(	highly ABA-induced PP2C gene 3			LOC_Os01	protein phosphatase 2C				
AT3G55646				LOC_Os01	expressed protein				
AT3G55646				LOC_Os01	expressed	putative	expressed		
AT2G3797(	SOUL hem	Cre13.g56(	SOUL hem	LOC_Os01	SOUL heme-binding protein				
AT1G0730(	josephin protein-related			LOC_Os01	expressed	putative	expressed		
AT1G0823(	Transmembrane amino acid trans			LOC_Os01	transmeml	Cytochrom	CYP197 su	LOC_Os01	cytochrom

AT2G2769(	cytochrom	family 94	subfamily	polypeptid	Cre01.g00:	putative	expressed	
AT2G3627(	Basic-leucine zipper (bZIP) transc			LOC_Os01	bZIP transc	expressed		
55 GO:0022900 GO:0051536 GO:0051537				LOC_Os01	2Fe-2S iror	expressed		
AT5G6475(	Integrase-t	Cre16.g67	AP2-domai	LOC_Os01	AP2 domai	expressed		
AT1G2254(	Major facilitator superfamily prote			LOC_Os01	POT family protein			
AT1G3343(	Galactosyltransferase family protein							
AT1G3343(	Galactosyltransferase family protein					5-DOPA dic	putative	expressed
AT4G1509:	catalytic Li	Cre09.g40	(1 of 1) 1.1:	LOC_Os01	4			
				LOC_Os01	expressed	chloroplas	putative	expressed
AT1G4380(	Plant stear	Cre17.g70	plastid acy	LOC_Os01	acyl-desat	putative	expressed	
)6 GO:0016491 GO:0055114				LOC_Os01	gibberellin	putative	expressed	
				LOC_Os01	ARGOS			
AT1G5502(	lipoxygena	Cre12.g51	(1 of 1) 1.13.11.12//1.13.11.58 - Linoleate 13S-lipoxygenase / Lipoxidase // Linol					
AT1G5502(	lipoxygena	Cre12.g51	(1 of 1) 1.13.11.12//1.13.11.58 - L			auxin-resp	putative	expressed
AT3G0757(	Cytochrom	Cre03.g20	(1 of 1) PTH	ISOFORM	LOC_Os01	expressed		
)0 GO:0005739 GO:0006355				LOC_Os02	mTERF fam	RING-type	putative	expressed
AT1G2444(	RING/U-box superfamily protein			LOC_Os01	zinc finger			
AT2G3646(	Aldolase superfamily protein					putative	expressed	
)8 GO:0008234				LOC_Os01	cysteine proteinase EP-B 1 precursor			
15						expressed		
				LOC_Os01	LTPL38 - Protease inhibitor/seed storage/LTP family prote			
AT4G1297(	stomagen			LOC_Os01	expressed	putative	expressed	
AT4G1403(	selenium-b	Cre03.g16	Selenium b	LOC_Os01	selenium-b	putative	expressed	
AT4G1403(	selenium-b	Cre03.g16	Selenium b	LOC_Os01	selenium-b	C3HC4 typ	expressed	
AT5G4865!	RING/U-box superfamily protein			LOC_Os01	zinc finger	C3HC4 typ	expressed	
AT5G4865!	RING/U-box superfamily protein			LOC_Os01	zinc finger			
AT4G0289(	Ubiquitin family protein							
AT4G0289(	Ubiquitin family protein							
AT4G0289(	Ubiquitin family protein							
AT4G0289(	Ubiquitin family protein							
AT4G0289(	Ubiquitin family protein							
AT4G0289(	Ubiquitin family protein					putative	expressed	
				LOC_Os01	antifreeze glycoprotein			
AT5G5061(	(1 of 11) PF14364 - Domain of unk			LOC_Os01	expressed	putative	expressed	
77 GO:0006355				LOC_Os01	No apical meristem protein			
47						putative	expressed	
AT5G1329(	Protein kinase superfamily protein			LOC_Os01	receptor-lil	putative	expressed	
)0 GO:0006508				LOC_Os01	xylanase in	putative	expressed	
)0 GO:0006508				LOC_Os01	xylanase in	putative	expressed	
				LOC_Os06	DUF640 domain containing protein			
15		Cre11.g46	(1 of 15) 2.5.1.18 - Glutathione tra			putative	expressed	
AT1G0831!	ARM repeat superfamily protein			LOC_Os01	spotted lea	putative	expressed	
AT1G7883(	Curculin-like (mannose-binding) l			LOC_Os01	secreted glycoprotein			
AT5G45310				LOC_Os01	expressed	putative	expressed	

AT1G7169	Peroxidase superfamily protein			LOC_Os01	peroxidase	putative	expressed	
				LOC_Os10	AWPM-19-l	expressed		
00 GO:0006355 GO:0043565				LOC_Os01	WRKY17	putative	expressed	
77		Cre02.g11	(1 of 1) PTH	LOC_Os01	MYB family transcription factor			
				LOC_Os10	expressed	expressed		
AT1G2058	Small nucl	Cre10.g45	Small nucl	LOC_Os02	LSM domain containing protein			
						putative	expressed	
09				LOC_Os02	EF hand fa	putative	expressed	
39				LOC_Os02	membrane	putative	expressed	
39				LOC_Os02	membrane	putative	expressed	
AT5G4712	BAX inhibit	Cre01.g06	(1 of 1) PTH	LOC_Os02	transmeml	putative	expressed	
AT3G4957	response to low sulfur 3			LOC_Os02	UP-9A	putative	expressed	
AT1G2346	Pectin lyase-like superfamily prote			LOC_Os02	polygalacturonase			
				LOC_Os02	expressed protein			
				LOC_Os02	expressed protein			
AT5G6310	S-adenosyl	Cre07.g31	conserved	LOC_Os02	expressed	putative	expressed	
24 GO:0016866		Cre01.g01	Terpenoid	LOC_Os11	cycloarten	putative	expressed	
24 GO:0016866		Cre01.g01	Terpenoid	LOC_Os11	cycloarten	putative	expressed	
24 GO:0016866		Cre01.g01	Terpenoid	LOC_Os11	cycloarten	putative	expressed	
77 GO:0005634 GO:0006355 GO:0009725				LOC_Os02	auxin resp	putative	expressed	
77 GO:0005634 GO:0006355 GO:0009725				LOC_Os02	auxin resp	putative	expressed	
AT1G1533	Cystathionine beta-synthase (CBS			LOC_Os02	CBS domai	C3HC4 typ	expressed	
AT2G1867	RING/U-box superfamily protein			LOC_Os02	zinc finger	chaperone	putative	expressed
AT2G2514	casein lytic	Cre02.g09	ClpB chape	Hsp100 fa	LOC_Os02g08490			
				LOC_Os02	expressed protein			
AT4G37090				LOC_Os02	expressed	putative	expressed	
AT3G2834	ABC transporter family protein			LOC_Os02	multidrug r	putative	expressed	
AT1G5120	A20/AN1-like zinc finger family pro			LOC_Os02	zinc finger	putative	expressed	
AT1G5120	A20/AN1-like zinc finger family pro			LOC_Os02	zinc finger	putative	expressed	
AT1G5256	HSP20-like chaperones superfami			LOC_Os02	hsp20/alph	putative	expressed	
AT5G0420	metacaspa	Cre03.g18	(1 of 1) PTH	LOC_Os11	ICE-like pro	putative	expressed	
AT5G0420	metacaspa	Cre03.g18	(1 of 1) PTH	LOC_Os11	ICE-like protease p20 domain containing protein			
AT2G2664	3-ketoacyl-	Cre17.g72	Type III polyketide synthase					
AT2G2664	3-ketoacyl-	Cre17.g72	Type III polyketide synthase					
15						putative	expressed	
				LOC_Os02	hsp20/alph	putative	expressed	
				LOC_Os02	hsp20/alph	putative	expressed	
				LOC_Os02	hsp20/alph	putative	expressed	
AT5G2593	Protein kin	Cre12.g50	(1 of 6) PFC	LOC_Os02	receptor-lil	expressed		
00 GO:0005634 GO:0006355 GO:0043565				LOC_Os02	HSF-type DNA-binding domain containing protein			
AT2G4274	ribosomal	Cre01.g02	Cytosolic 80S ribosomal protein L11					
15 GO:0008270						putative	expressed	
AT2G4256	late embryogenesis abundant don			LOC_Os02	late embry	putative	expressed	
AT2G4256	late embryogenesis abundant don			LOC_Os02	late embry	putative	expressed	

AT5G5316(	regulatory components of ABA rec			LOC_Os02	CAPIP1	putative	expressed		
AT5G5316(	regulatory components of ABA rec			LOC_Os02	CAPIP1	putative	expressed		
AT5G5316(	regulatory components of ABA rec			LOC_Os02	CAPIP1	putative	expressed		
AT5G5316(	regulatory components of ABA rec			LOC_Os02	CAPIP1	putative	expressed		
AT5G5316(	regulatory components of ABA rec			LOC_Os02	CAPIP1				
AT5G24130				LOC_Os02	expressed	putative	expressed		
97 GO:0005506 GO:0016705 GO:0020037 GO				LOC_Os02	cytochrom	putative	expressed		
				LOC_Os06	late embry	putative	expressed		
24 GO:0004722 GO:00(		Cre01.g03(	Flagellar As	LOC_Os02	protein phc	LOC_Os04	importin-8	putative	expressed
AT2G3166(	ARM repea	Cre13.g56	Importin be	RAN-bindi	8				
AT1G27300				LOC_Os02	expressed protein				
AT1G27300				LOC_Os02	expressed	glycerophо	putative	expressed	
AT5G4108(	PLC-like ph	Cre03.g20	Glycerophc	probably a	LOC_Os02	glycerophо	putative	expressed	
AT5G4108(	PLC-like ph	Cre03.g20	Glycerophc	probably a	LOC_Os02	glycerophо	putative	expressed	
AT5G4108(	PLC-like ph	Cre03.g20	Glycerophc	probably a	LOC_Os02g31030				
						4-alpha-glu	chloroplas	putative	expressed
AT5G0365(	starch brar	Cre10.g44	Starch Brar	LOC_Os02	1	putative	expressed		
AT5G2247(	NAD+ ADP-ribosyltransferases;NA			LOC_Os02	poly synthe	putative	expressed		
AT5G2247(	NAD+ ADP-ribosyltransferases;NA			LOC_Os02	poly synthe	putative	expressed		
57				LOC_Os02	pectinester	putative	expressed		
57				LOC_Os02	pectinesterase inhibitor domain containing protein				
38		Cre14.g61	(1 of 5) 2.3.1.43 - Phosphatidylcholine--sterol O-acyltransferase / Phospholipid--						
38		Cre14.g61	(1 of 5) 2.3.1.43 - Phosphatidylchc			putative	expressed		
AT1G7699(	ACT domai	Cre02.g11	(1 of 2) 2.7	LOC_Os02	ACT domai	putative	expressed		
AT1G7699(	ACT domai	Cre02.g11	(1 of 2) 2.7	LOC_Os02	ACT domai	putative	expressed		
AT1G7699(	ACT domai	Cre02.g11	(1 of 2) 2.7	LOC_Os02	ACT domain containing protein				
AT4G13530				LOC_Os02	expressed	putative	expressed		
AT5G0420(	metacaspase 9			LOC_Os11	ICE-like protease p20 domain containing protein				
21 GO:0022857 GO:0055085				LOC_Os02	expressed protein				
21 GO:0022857 GO:0055085				LOC_Os02	expressed protein				
21 GO:0022857 GO:0055085				LOC_Os02	expressed protein				
21 GO:0022857 GO:0055085				LOC_Os02	expressed protein				
21 GO:0022857 GO:0055085				LOC_Os02	expressed	putative	expressed		
AT1G1880(	NAP1-relat	Cre06.g30	Nucleosom	LOC_Os02	SET	putative	expressed		
AT3G2167(	Major facilitator superfamily prote			LOC_Os04	peptide tra	putative	expressed		
AT3G2167(	Major facilitator superfamily prote			LOC_Os04	peptide transporter PTR2				
						putative	expressed		
				LOC_Os12	late embryogenesis abundant protein D-34				
						putative	expressed		
AT4G2669(	PLC-like phosphodiesterase famil			LOC_Os02	glycerophо	putative	expressed		
AT4G2669(	PLC-like phosphodiesterase famil			LOC_Os02	glycerophosphoryl diester phosphodiesterase family prot				
AT2G31410				LOC_Os02	expressed	expressed protein			
AT3G2525(	AGC (cAMF	cGMP-dependent and protein kin			LOC_Os02	putative	expressed		
AT4G3227	Nucleotide	Cre16.g66(	UDP-N-ace	LOC_Os02	transporter	LOC_Os02	RNA recogr	putative	expressed

6 GO:0003676		Cre02.g08	(1 of 90) PF	RBD	or RNP dor	putative	expressed	
AT2G3704(	PHE ammonia lyase 1			LOC_Os02	phenylalan	putative	expressed	
AT2G2831	Nucleotide/sugar transporter fami			LOC_Os02	transporter	expressed		
AT4G0898(	F-BOX WITH WD-40 2			LOC_Os02	OsFBX61 -	expressed		
AT4G0898(	F-BOX WITH WD-40 2			LOC_Os02	OsFBX61 -	expressed		
AT4G0898(	F-BOX WITH WD-40 2			LOC_Os02	OsFBX61 -	expressed		
AT4G0898(	F-BOX WITH WD-40 2			LOC_Os02	OsFBX61 -	expressed		
AT4G0898(	F-BOX WITH WD-40 2			LOC_Os02	OsFBX61 -	wound/stre	putative	expressed
AT4G3973(	Lipase/lipo	PLAT/LH2 family protein			LOC_Os02	putative	expressed	
AT3G5707(	Glutaredoxin family protein			LOC_Os02	glutaredoxi	putative	expressed	
AT1G2677(	expansin A	Cre17.g72	(1 of 11) PT	LOC_Os02	expansin precursor			
						putative	expressed	
AT1G1479(	RNA-dependent RNA polymerase			LOC_Os02	RNA-deper	putative	expressed	
AT1G1479(	RNA-dependent RNA polymerase			LOC_Os02	RNA-deper	putative	expressed	
AT1G1479(	RNA-dependent RNA polymerase			LOC_Os02	RNA-deper	putative	expressed	
AT1G1479(	RNA-dependent RNA polymerase			LOC_Os02	RNA-deper	putative	expressed	
AT1G1479(	RNA-dependent RNA polymerase			LOC_Os02	RNA-deper	putative	expressed	
AT1G1479(	RNA-dependent RNA polymerase			LOC_Os02	RNA-deper	putative	expressed	
77		Cre06.g28	Hua Enhan	LOC_Os02	MYB family	putative	expressed	
AT5G3768(	ADP-ribosy	Cre17.g70	ARF-like G	LOC_Os02	ADP-ribosy	putative	expressed	
77 GO:0006355				LOC_Os02	dof zinc fin	G-beta rep	expressed	
AT3G1330(	Transducin	Cre12.g55	(1 of 1) PTH	LOC_Os02	WD domair	putative	expressed	
AT5G5614(	RNA-binding KH domain-containir			LOC_Os02	KH domain	expressed		
AT1G1203(	Protein of u	Cre03.g14	PWR motif	LOC_Os02	plant-spec	putative	expressed	
AT5G3570(	fimbrin-like protein 2			LOC_Os02	fimbrin-like	putative	expressed	
AT5G3570(	fimbrin-like protein 2			LOC_Os02	fimbrin-like	putative	expressed	
AT5G3570(	fimbrin-like protein 2			LOC_Os02	fimbrin-like	protein 2		
						putative	expressed	
AT5G1307(	MSF1-like family protein			LOC_Os02	slowmo ho	fatty acid d	putative	expressed
AT3G1212(	fatty acid d	Cre17.g71	Fatty acid c	delta-12	LOC_Os02	fatty acid d	putative	expressed
AT3G1212(	fatty acid d	Cre17.g71	Fatty acid c	delta-12	LOC_Os02	fatty acid d	putative	expressed
AT3G1212(	fatty acid d	Cre17.g71	Fatty acid c	delta-12	LOC_Os02	putative	expressed	
				LOC_Os02	hsp20/alpha crystallin family protein			
AT5G1478(	formate de	Cre01.g01	(1 of 1) PTHR10996:SF20 - D-3-PH			putative	expressed	
AT1G3188(	DZC (Disease resistance/zinc fing			LOC_Os02	Disease re	putative	expressed	
AT1G3188(	DZC (Disease resistance/zinc fing			LOC_Os02	Disease re	putative	expressed	
33 GO:0006810 GO:00		Cre06.g29	Amino acid	LOC_Os02	amino acid	putative	expressed	
33 GO:0006810 GO:00		Cre06.g29	Amino acid	LOC_Os02	amino acid	putative	expressed	
33 GO:0006810 GO:00		Cre06.g29	Amino acid	LOC_Os02	amino acid	putative	unclassifie	expressed
AT3G2237(	alternative	Cre09.g39	Alternative	LOC_Os02	transposor	putative	unclassifie	expressed
AT3G2237(	alternative	Cre09.g39	Alternative	LOC_Os02	transposon protein			
AT1G32690				LOC_Os02	expressed	putative	expressed	
AT1G4475(	purine permease 11			LOC_Os02	purine perr	RING-H2 fi	putative	expressed

AT1G7641(	RING/U-bo	Cre12.g50:	RING finge	SDIR1 orth	LOC_Os02g46100			
AT1G5334!	(1 of 1) PTHR13734//PTHR13734:!			LOC_Os02	expressed protein			
AT1G5334!	(1 of 1) PTHR13734//PTHR13734:!			LOC_Os02	expressed protein			
AT1G5334!	(1 of 1) PTHR13734//PTHR13734:!			LOC_Os02	expressed	putative	expressed	
AT5G5251(	SCARECROW-like 8			LOC_Os02	chitin-indu	putative	expressed	
AT1G0551(	Protein of u	Cre03.g15:	(1 of 1) PF(	LOC_Os05	DUF1264 d	expressed		
AT2G3602(	Ovate family protein			LOC_Os02	DUF623 dc	expressed		
AT3G6106(	phloem protein 2-A13			LOC_Os02	OsFBX58 - F-box domain containing protein			
AT5G2529(	F-box family protein with a domair			LOC_Os02	expressed	putative	expressed	
38		Cre14.g61!	(1 of 5) 2.3	LOC_Os02	GDSL-like l	rho-GTPase	putative	expressed
AT4G3575(	SEC14 cyto	Cre16.g69:	(1 of 1) K18	CDC42GA	LOC_Os02g44820			
l5 GO:0006810 GO:0016020								
l5 GO:0006810 GO:0016020						expressed		
AT1G0203(	C2H2-like zinc finger protein			LOC_Os02	ZOS2-14 - (	putative	expressed	
AT2G4511(	expansin B	Cre03.g17:	(1 of 11) PT	LOC_Os02	expansin precursor			
AT2G4511(	expansin B	Cre03.g17:	(1 of 11) PTHR31867//PTHR31867:SF1 - FAMILY NOT NAMED // EXPANSIN-A1-RE					
AT4G1667(	Plant protein of unknown function			LOC_Os02	expressed	expressed		
AT2G4494(	Integrase-type DNA-binding super			LOC_Os02	AP2 domai	expressed		
77 GO:0003700 GO:0006355				LOC_Os02	AP2 domain containing protein			
						zinc finger	C3HC4 typ	expressed
l5 GO:0008270		Cre12.g50:	RING finge	SDIR1 orth	LOC_Os02	putative	expressed	
AT1G6271(	beta vacuo	Cre12.g48:	Vacuolar p	LOC_Os02	vacuolar-processing enzyme precursor			
AT1G0180(	NAD(P)-binding Rossmann-fold superfamily protein					putative	expressed	
AT5G0864(	flavonol synthase 1			LOC_Os02	flavonol synthase/flavanone 3-hydroxylase			
AT1G6814(	Protein of unknown function (DUF			LOC_Os02	expressed protein			
AT1G6814(	Protein of unknown function (DUF			LOC_Os02	expressed	3-beta-gluc	putative	expressed
AT2G0579(	O-Glycosyl hydrolases family 17 p			LOC_Os02	glucan end	3-beta-gluc	putative	expressed
AT2G0579(	O-Glycosyl hydrolases family 17 p			LOC_Os02	glucan end	expressed		
AT2G4194(	zinc finger protein 8			LOC_Os02	ZOS2-17 - C2H2 zinc finger protein			
				LOC_Os02	expressed protein			
				LOC_Os02	expressed	putative	expressed	
AT1G5405(	HSP20-like chaperones superfami			LOC_Os02	hsp20/alpl	putative	expressed	
AT4G3315(	lysine-ketoglutarate reductase/sa			LOC_Os02	saccharopi	putative	expressed	
AT4G3315(	lysine-ketoglutarate reductase/sa			LOC_Os02	saccharopi	putative	expressed	
AT4G3315(	lysine-ketoglutarate reductase/sa			LOC_Os02	saccharopi	putative	expressed	
AT4G3315(	lysine-ketoglutarate reductase/sa			LOC_Os02	saccharopi	putative	expressed	
AT4G3315(	lysine-ketoglutarate reductase/sa			LOC_Os02	saccharopi	expressed		
		Cre06.g27!	(1 of 4) PTH	LOC_Os02	SCP-like ex	putative	expressed	
AT4G3044(	UDP-D-glu	Cre06.g27!	UDP-D-glu	LOC_Os02	UDP-glucu	putative	expressed	
AT4G3044(	UDP-D-glu	Cre06.g27!	UDP-D-glu	LOC_Os02	UDP-glucu	putative	expressed	
AT4G3044(	UDP-D-glu	Cre06.g27!	UDP-D-glu	LOC_Os02	UDP-glucu	NUDIX fam	domain co	expressed
AT1G7969(	nudix hydrolase homolog 3			LOC_Os02	hydrolase	CAF1 famil	putative	expressed
AT2G3207(	Polynucleo	ribonuclea	Cre03.g15:	CCR4-assc	LOC_Os02	expressed		
AT2G0223(	phloem protein 2-B1			LOC_Os02	OsFBX70 -	putative	expressed	

AT3G0709(	PPPDE put	Cre12.g55!	(1 of 1) KO(	LOC_Os02	thioredoxir	putative	expressed	
				LOC_Os02	CBS domai	putative	expressed	
				LOC_Os02	CBS domain containing membrane protein			
55 GO:0015238 GO:0015297 GO:0016020 GO:0055085						putative	expressed	
21 GO:0055085		Cre09.g39!	(1 of 2) PTH	LOC_Os02	Citrate trar	putative	expressed	
21 GO:0055085		Cre09.g39!	(1 of 2) PTH	LOC_Os02	Citrate transporter protein			
15		Cre16.g671500				putative	expressed	
29 GO:0016717 GO:00!		Cre01.g03	Chloroplas	LOC_Os12	fatty acid desaturase			
				LOC_Os12	expressed	Tramtrack	Broad Con	expressed
AT2G3052(	Phototropic-responsive NPH3 fam			LOC_Os11	BTBN20 - B	Tramtrack	Broad Con	expressed
AT2G3052(	Phototropic-responsive NPH3 fam			LOC_Os11	BTBN20 - B	Tramtrack	Broad Con	expressed
AT2G3052(	Phototropic-responsive NPH3 fam			LOC_Os11	BTBN20 - B	Tramtrack	Broad Con	expressed
AT2G3052(	Phototropic-responsive NPH3 fam			LOC_Os11	BTBN20 - B	Tramtrack	Broad Con	expressed
AT2G3052(	Phototropic-responsive NPH3 fam			LOC_Os11	BTBN20 - B	Tramtrack	Broad Con	expressed
AT2G3052(	Phototropic-responsive NPH3 fam			LOC_Os11	BTBN20 - B	Tramtrack	Broad Con	expressed
AT2G3052(	Phototropic-responsive NPH3 fam			LOC_Os11	BTBN20 - B	Tramtrack	Broad Con	expressed
AT2G3052(	Phototropic-responsive NPH3 fam			LOC_Os11	BTBN20 - B	Tramtrack	Broad Con	expressed
AT2G3052(	Phototropic-responsive NPH3 fam			LOC_Os11	BTBN20 - B	expressed		
AT3G5432(	Integrase-t	Cre13.g602750		LOC_Os11	AP2 domai	plasma me	putative	expressed
AT3G5733(	autoinhibit	Cre09.g38	Flagellar As	LOC_Os12	calcium-tra	plasma me	putative	expressed
AT3G5733(	autoinhibit	Cre09.g38	Flagellar As	LOC_Os12	calcium-transporting ATPase			
AT5G1914(	Aluminium	Cre10.g44	(1 of 4) PTHR11772//PTHR11772:			expressed		
AT5G1646(	Putative adipose-regulatory protei			LOC_Os12	protein of u	major facil	putative	expressed
21 GO:0055085		Cre16.g65(	(1 of 1) PTH	LOC_Os11	transporter			
50								
52 GO:0008667 GO:00		Cre16.g68	Tropine dehydrogenase/reductase					
52 GO:0016491		Cre16.g68	Tropine dehydrogenase/reductase			putative	expressed	
				LOC_Os11	amino acid transporter			
AT1G58070				LOC_Os11	expressed	putative	expressed	
		Cre07.g31	Heat shock	LOC_Os11	hsp20/alph	putative	expressed	
24 GO:0016866		Cre01.g01	Terpenoid (	LOC_Os11	cycloartenol synthase			
31								
31								
70 GO:0016491 GO:0055114						putative	expressed	
15 GO:0051087				LOC_Os11	IQ calmodu	putative	expressed	
37 GO:0008152 GO:0010333 GO:0016829				LOC_Os03	terpene syr	putative	expressed	
37 GO:0008152 GO:0010333 GO:0016829				LOC_Os03	terpene syr	putative	expressed	
52 GO:0010333 GO:0016829				LOC_Os03	terpene syr	putative	expressed	
37 GO:0008152 GO:0010333 GO:0016829				LOC_Os03	terpene syr	putative	expressed	
AT5G1398(	Glycosyl hydrolase family 38 prote			LOC_Os11	lysosomal alpha-mannosidase precursor			
AT5G07330				LOC_Os11	expressed protein			
15 GO:0008270				LOC_Os11	expressed	putative	expressed	
04		Cre12.g55	14-3-3 prot	LOC_Os11	14-3-3 prot	putative	expressed	

04		Cre12.g55!	14-3-3 prot	LOC_Os11	14-3-3 protein			
						putative	expressed	
76	GO:0008083 GO:0008283			LOC_Os11	phytosulfo	putative	expressed	
AT2G3588(	TPX2 (targeting protein for Xklp2) p			LOC_Os11	lymphoid o	transmemb	putative	expressed
		Cre01.g03!	(1 of 1) PTH	ISOFORM	LOC_Os11	putative	expressed	
AT3G0353(	non-specific phospholipase C4			LOC_Os11	phosphoes	putative	expressed	
29	GO:0008152			LOC_Os11	patatin	putative	expressed	
29	GO:0008152			LOC_Os11	patatin	putative	expressed	
29	GO:0008152			LOC_Os11	patatin	putative	expressed	
29	GO:0008152			LOC_Os11	patatin	putative	expressed	
51	GO:0005634 GO:0007050			LOC_Os11	cyclin-dependent kinase inhibitor			
35								
35						expressed		
AT1G5353(	Peptidase S	Cre11.g46	Mitochond	LOC_Os11	OsSigP7 - F	expressed		
AT1G5353(	Peptidase S	Cre11.g46	Mitochond	LOC_Os11	OsSigP7 - F	putative	expressed	
AT1G1737(	oligouridylate binding protein 1B			LOC_Os11	RNA recognition motif containing protein			
						putative	expressed	
20	GO:0016021 GO:0022857 GO:0022891 GO			LOC_Os11	transporter	putative	expressed	
20	GO:0016021 GO:0022857 GO:0022891 GO			LOC_Os11	transporter	putative	expressed	
20	GO:0016021 GO:0022857 GO:0022891 GO			LOC_Os11	transporter family protein			
52	GO:0016758							
35								
35								
35								
52	GO:0016627 GO:0045735 GO:0050660 GO:0055114							
35								
35						putative	expressed	
AT2G2266(	Protein of u	Cre13.g56!	(1 of 10) PF	LOC_Os11	DUF1399 c	putative	expressed	
AT2G2266(	Protein of u	Cre13.g56!	(1 of 10) PF	LOC_Os11	DUF1399 c	putative	expressed	
AT2G2266(	Protein of u	Cre13.g56!	(1 of 10) PF	LOC_Os11	DUF1399 c	putative	expressed	
AT2G2266(	Protein of u	Cre13.g56!	(1 of 10) PF	LOC_Os11	DUF1399 c	putative	expressed	
AT2G2266(	Protein of u	Cre13.g56!	(1 of 10) PF	LOC_Os11	DUF1399 c	putative	expressed	
AT2G2266(	Protein of u	Cre13.g56!	(1 of 10) PF	LOC_Os11	DUF1399 c	putative	expressed	
AT2G2266(	Protein of u	Cre13.g56!	(1 of 10) PF	LOC_Os11	DUF1399 containing protein			
				LOC_Os11	expressed protein			
				LOC_Os11	expressed	putative	expressed	
				LOC_Os11	NBS-LRR d	putative	expressed	
				LOC_Os11	NBS-LRR disease resistance protein			
				LOC_Os01	expressed protein			
31								
31								
31								

72 GO:0004674 GO:00(		Cre01.g01:	(1 of 18) KOG0581 - Mitogen-activated protei				LOC_Os04	cytochrom	putative
AT5G0690(	cytochrom	family 93	subfamily	polypeptide 1		putative	expressed		
AT5G5264(	heat shock	Cre09.g38(	Heat shock	LOC_Os04	heat shock	putative	expressed		
AT5G5264(	heat shock	Cre09.g38(	Heat shock	LOC_Os04	heat shock	putative	expressed		
				LOC_Os03	zinc finger	expressed			
				LOC_Os03	WD40-like Beta Propeller Repeat family protein				
		Cre02.g10:	(1 of 1) PTHR31013:SF2 - PROTEIN			putative	expressed		
AT5G6090(	receptor-lil	Cre01.g01:	(1 of 18) K(	LOC_Os04	receptor-like protein kinase				
AT1G27990				LOC_Os04	expressed	LOC_Os04	2-oxoglutaı	mitochonc	putative
AT5G6575(	2-oxoglutaı	E1 compoı	Cre12.g53:	2-oxoglutaı	E1 subunit	LOC_Os04	2-oxoglutaı	mitochonc	putative
AT5G6575(	2-oxoglutaı	E1 compoı	Cre12.g53:	2-oxoglutaı	E1 subunit	LOC_Os04	2-oxoglutaı	mitochonc	putative
AT5G6575(	2-oxoglutaı	E1 compoı	Cre12.g53:	2-oxoglutaı	E1 subunit	LOC_Os04	2-oxoglutaı	mitochonc	putative
AT5G6575(	2-oxoglutaı	E1 compoı	Cre12.g53:	2-oxoglutaı	E1 subunit				
						putative	expressed		
52 GO:0016491				LOC_Os04	dehydrogeı	putative	expressed		
52 GO:0016491				LOC_Os04	11-beta-hy	expressed			
AT3G0200(	Thioredoxin superfamily protein			LOC_Os02	OsGrx_C8 -	glutaredoxin subgroup III			
AT4G2082(	FAD-bindin	Cre09.g39:	(1 of 1) PTHR11748//PTHR11748:SF75 - D-LACTATE DEHYDROGENASE // SUBFA						
				LOC_Os04	expressed protein				
				LOC_Os04	expressed	putative	expressed		
01 GO:0046872				LOC_Os04	basic proliı	putative	expressed		
				LOC_Os04	basic proliı	putative	expressed		
				LOC_Os04	basic proliı	putative	expressed		
AT1G4798(	(1 of 2) PF1	Cre01.g03:	(1 of 1) PF1	LOC_Os04	desiccatioı	putative	expressed		
93 GO:0016787 GO:0046872				LOC_Os04	Ser/Thr protein phosphatase family protein				
AT5G06270				LOC_Os04	expressed	putative	expressed		
50				LOC_Os04	abscisic stı	putative	expressed		
				LOC_Os04	abscisic stı	putative	expressed		
				LOC_Os04	abscisic stı	putative	expressed		
AT1G4974(	PLC-like phosphodiesterases sup(			LOC_Os04	phospholip	putative	expressed		
74 GO:0006457 GO:0042803 GO:0051087				LOC_Os04	co-chaperone GrpE protein				
AT1G7210(	late embryogenesis abundant domain-containing protein / LEA domain-containing protein								
24 GO:0010181 GO:00:		Cre03.g20(	(1 of 2) K05894 - 12-oxophytodienoic acid reductase (OPR)						
24 GO:0010181 GO:00:		Cre03.g20(	(1 of 2) K05894 - 12-oxophytodienoic acid reductase (OPR)						
24 GO:0010181 GO:00:		Cre03.g20(	(1 of 2) K05894 - 12-oxophytodien			putative	expressed		
AT4G1025(	HSP20-like	Cre07.g31	Heat shock	LOC_Os04	hsp20/alph	putative	expressed		
AT3G1722(	pectin methylesterase inhibitor 2			LOC_Os04	PME/invert	expressed			
				LOC_Os04	DUF623 dc	RNA recogı	putative	expressed	
AT1G4749(	RNA-bindir	Cre16.g65:	(1 of 16) K(	subunit 4	LOC_Os04	putative	expressed		

AT2G0285(	plantacyan	Cre14.g62(	(1 of 1) PFC	LOC_Os03	plastocyan	wound/stre	putative	expressed
AT4G3973(	Lipase/lipo	PLAT/LH2 family protein			LOC_Os04	putative	expressed	
)0 GO:0005634				LOC_Os04	ethylene-ir	putative	expressed	
)0 GO:0005634				LOC_Os04	ethylene-ir	putative	expressed	
AT3G4780(	Galactose	Cre06.g26:	Aldose-1-e	LOC_Os04	aldose 1-ep	putative	expressed	
AT3G0252(	general reg	Cre12.g55(	14-3-3 prot	LOC_Os04	14-3-3 prot	putative	expressed	
AT3G0252(	general reg	Cre12.g55(	14-3-3 prot	LOC_Os04	14-3-3 prot	putative	expressed	
AT3G0252(	general reg	Cre12.g55(	14-3-3 prot	LOC_Os04	14-3-3 prot	putative	expressed	
AT5G4077(	prohibitin 3	Cre12.g51(	Prohibitin	LOC_Os04	prohibitin	putative	expressed	
AT3G4807(	RING/U-box superfamily protein			LOC_Os04	protein bin	expressed		
				LOC_Os04	GASR4 - Gibberellin-regulated GASA/GAST/Snakin family			
AT3G0760(	Heavy metal transport/detoxification superfamily protein							
AT3G0760(	Heavy metal transport/detoxification superfamily protein							
AT3G0760(	Heavy metal transport/detoxification superfamily protein					putative	expressed	
AT1G1313(	Cellulase (glycosyl hydrolase fami			LOC_Os04	glycosyl hy	putative	expressed	
AT1G1313(	Cellulase (glycosyl hydrolase fami			LOC_Os04	glycosyl hy	expressed		
AT3G0166(	S-adenosyl	Cre12.g54:	putative m	LOC_Os04	methyltran	G-beta rep	expressed	
AT1G2453(	Transducin/WD40 repeat-like sup			LOC_Os04	WD domain	putative	expressed	
'79 GO:0008113 GO:00:		Cre01.g01:	Peptide me	LOC_Os04	peptide methionine sulfoxide reductase			
AT3G2180(	UDP-gluco	Cre07.g32:	(1 of 1) PTHR11926//PTHR11926:S			protein kina	putative	expressed
AT3G2525(	AGC (cAMP	cGMP-dependent and protein kina			LOC_Os04	putative	expressed	
AT2G3173(	basic helix-loop-helix (bHLH) DNA			LOC_Os04	ethylene-re	putative	expressed	
AT2G3173(	basic helix-loop-helix (bHLH) DNA			LOC_Os04	ethylene-re	putative	expressed	
AT2G3173(	basic helix-loop-helix (bHLH) DNA			LOC_Os04	ethylene-responsive protein related			
'68 GO:0005975 GO:0006032 GO:0016998								
AT2G4359(	Chitinase family protein							
						putative	unclassifie	expressed
AT2G2480(	Peroxidase superfamily protein			LOC_Os04	retrotransp	expressed		
AT3G5012(	Plant protein of unknown function			LOC_Os04	plant prote	putative	expressed	
AT4G2674(	seed gene	Cre06.g28:	(1 of 1) PTH	LOC_Os04	caleosin re	putative	expressed	
AT4G2674(	seed gene	Cre06.g28:	(1 of 1) PTH	LOC_Os04	caleosin re	putative	expressed	
AT4G2674(	seed gene	Cre06.g28:	(1 of 1) PTH	LOC_Os04	caleosin related protein			
)9 GO:0009523 GO:0009654 GO:0015979 GO				LOC_Os04	expressed	putative	expressed	
l5 GO:0006810 GO:00:		Cre12.g54(	Aquaporin	LOC_Os04	aquaporin	putative	expressed	
l5 GO:0006810 GO:00:		Cre12.g54(	Aquaporin	LOC_Os04	aquaporin	putative	expressed	
AT4G0493(	fatty acid desaturase family protei			LOC_Os02	sphingolipi	putative	expressed	
AT4G2557(	Cytochrom	Cre13.g58(	(1 of 1) K08	LOC_Os04	cytochrome b561			
36 GO:0003677 GO:0005634 GO:0006334								
						putative	expressed	
AT4G2514(	oleosin 1			LOC_Os04	oleosin	putative	expressed	
AT4G2514(	oleosin 1			LOC_Os04	oleosin	putative	expressed	
AT4G2514(	oleosin 1			LOC_Os04	oleosin			
AT4G1749(	ethylene responsive element binding factor 6					expressed		
AT2G4494(	Integrase-type DNA-binding super			LOC_Os04	AP2 domain containing protein			

76 GO:0003677 GO:0046983						putative	expressed		
AT2G4508(	cyclin p3;1			LOC_Os04	cyclin	putative	expressed		
AT4G3835(	Patched fa	Cre12.g55(	Sterol sens	LOC_Os04	niemann-P	putative	expressed		
00 GO:0005634 GO:0006355 GO:0043565				LOC_Os04	heat stress	putative	expressed		
AT4G2306(	IQ-domain 22			LOC_Os04	IQ calmodulin-binding motif family protein				
						C3HC4 typ	expressed		
15 GO:0008270				LOC_Os04	zinc finger	putative	expressed		
AT3G6016(	multidrug resistance-associated p			LOC_Os04	multidrug r	putative	expressed		
AT3G6016(	multidrug resistance-associated p			LOC_Os04	multidrug resistance-associated protein				
AT1G7889(	(1 of 3) PTHR33972:SF2 - EXPRESSED PROTEIN					putative	expressed		
AT4G2005(	Pectin lyase-like superfamily prote			LOC_Os04	QRT3	putative	expressed		
AT4G2005(	Pectin lyase-like superfamily prote			LOC_Os04	QRT3	expressed			
AT5G6057(	Galactose oxidase/kelch repeat su			LOC_Os04	OsFBK15 -	putative	expressed		
AT2G3466(	multidrug r	Cre04.g22(	(1 of 13) 3.(	LOC_Os04	ABC transp	putative	expressed		
AT2G3466(	multidrug r	Cre04.g22(	(1 of 13) 3.(	LOC_Os04	ABC transp	putative	expressed		
AT2G3466(	multidrug r	Cre04.g22(	(1 of 13) 3.(	LOC_Os04	ABC transp	putative	expressed		
AT2G3466(	multidrug r	Cre04.g22(	(1 of 13) 3.(	LOC_Os04	ABC transp	putative	expressed		
AT2G3466(	multidrug r	Cre04.g22(	(1 of 13) 3.(	LOC_Os04	ABC transp	expressed			
AT3G4786(	chloroplastic lipocalin			LOC_Os04	OsCHL Chl	expressed			
AT3G4786(	chloroplastic lipocalin			LOC_Os04	OsCHL Chloroplastic lipocalin				
AT4G0321(	xyloglucan endotransglucosylase/hydrolase 9								
				LOC_Os04	expressed	putative	expressed		
91 GO:0055114				LOC_Os04	gibberellin	putative	expressed		
AT1G8045(	VQ motif-containing protein			LOC_Os04	VQ domain containing protein				
						expressed			
AT5G6721(	Protein of unknown function (DUF			LOC_Os04	plant-spec	putative	expressed		
AT1G7426(	purine bios	Cre08.g36(	(1 of 1) 6.3	LOC_Os01	phosphorib	glycosyl hy	putative	expressed	
53 GO:0005975		Cre12.g50(	(1 of 2) K01	sacA)	LOC_Os09	glycosyl hy	putative	expressed	
53 GO:0005975		Cre12.g50(	(1 of 2) K01	sacA)	LOC_Os09	putative	expressed		
01 GO:0046872				LOC_Os04	heavy meta	putative	expressed		
01 GO:0046872				LOC_Os04	heavy metal transport/		LOC_Os01	cytochrom	putative
AT1G6490(	cytochrom	family 89	subfamily	polypeptide 2		putative	expressed		
AT5G5375(	CBS domain-containing protein			LOC_Os04	CBS domain-containing protein				
AT4G21720				LOC_Os04	expressed	putative	expressed		
				LOC_Os04	ATOFP18/(	putative	expressed		
01 GO:0006979 GO:0020037 GO:0055114				LOC_Os04	peroxidase	putative	expressed		
				LOC_Os08	membrane associated DUF588 domain containing protei				
AT3G12960				LOC_Os08	expressed protein				
76 GO:0009664		Cre17.g72(	(1 of 11) PTHR31867//PTHR31867			RNA recogn	putative	expressed	
AT3G1140(	eukaryotic	Cre06.g26(	Eukaryotic	subunit G	LOC_Os02	expressed			
AT5G6319(	MA3 domai	Cre09.g41(	(1 of 1) K16	LOC_Os08	MA3 domai	expressed			
AT5G6319(	MA3 domai	Cre09.g41(	(1 of 1) K16	LOC_Os08	MA3 domai	putative	expressed		
AT1G1344(	glyceraldeh	Cre12.g48(	Glyceralde	LOC_Os08	glyceraldeh	putative	expressed		

AT1G1344(	glyceraldeh	Cre12.g48!	Glyceralde	LOC_Os08	glyceraldeh	putative	expressed	
AT5G4078(	lysine histidine transporter 1			LOC_Os08	amino acid	putative	expressed	
AT5G0856(	transducin	Cre08.g38:	(1 of 1) PTH	LOC_Os02	WD repeat-	putative	expressed	
AT5G0856(	transducin	Cre08.g38:	(1 of 1) PTH	LOC_Os02	WD repeat-	putative	expressed	
AT5G0856(	transducin	Cre08.g38:	(1 of 1) PTH	LOC_Os02	WD repeat-	expressed		
AT1G0605(	Protein of u	Cre13.g57:	(1 of 4) PFC	LOC_Os08	DUF1336 d	expressed		
AT1G0605(	Protein of u	Cre13.g57:	(1 of 4) PFC	LOC_Os08	DUF1336 d	expressed		
AT1G0605(	Protein of u	Cre13.g57:	(1 of 4) PFC	LOC_Os08	DUF1336 d	putative	expressed	
AT1G0106(	Homeodomain-like superfamily pr			LOC_Os08	MYB family	putative	expressed	
AT1G0106(	Homeodomain-like superfamily pr			LOC_Os08	MYB family	putative	expressed	
AT1G0106(	Homeodomain-like superfamily pr			LOC_Os08	MYB family	putative	expressed	
AT1G0106(	Homeodomain-like superfamily pr			LOC_Os08	MYB family	putative	expressed	
AT1G0106(	Homeodomain-like superfamily pr			LOC_Os08	MYB family	putative	expressed	
AT1G0106(	Homeodomain-like superfamily pr			LOC_Os08	MYB family	putative	expressed	
AT1G0106(	Homeodomain-like superfamily pr			LOC_Os08	MYB family	putative	expressed	
AT1G0106(	Homeodomain-like superfamily pr			LOC_Os08	MYB family	putative	expressed	
AT1G0106(	Homeodomain-like superfamily pr			LOC_Os08	MYB family	putative	expressed	
AT1G0106(	Homeodomain-like superfamily pr			LOC_Os08	MYB family	putative	expressed	
AT1G0106(	Homeodomain-like superfamily pr			LOC_Os08	MYB family	putative	expressed	
AT1G0106(	Homeodomain-like superfamily pr			LOC_Os08	MYB family	putative	expressed	
AT1G0106(	Homeodomain-like superfamily pr			LOC_Os08	MYB family	putative	expressed	
AT1G0106(	Homeodomain-like superfamily pr			LOC_Os08	MYB family	putative	expressed	
AT1G0106(	Homeodomain-like superfamily pr			LOC_Os08	MYB family	putative	expressed	
AT1G0106(	Homeodomain-like superfamily pr			LOC_Os08	MYB family	putative	expressed	
AT1G0106(	Homeodomain-like superfamily pr			LOC_Os08	MYB family	putative	expressed	
AT1G0106(	Homeodomain-like superfamily pr			LOC_Os08	MYB family	putative	expressed	
AT1G0106(	Homeodomain-like superfamily pr			LOC_Os08	MYB family	putative	expressed	
21				LOC_Os08	tetraspanir	putative	expressed	
AT1G3183(	Amino acid	Cre06.g29:	Amino acid	LOC_Os03	amino acid	putative	expressed	
AT1G3183(	Amino acid	Cre06.g29:	Amino acid	LOC_Os03	amino acid	putative	expressed	
AT1G3183(	Amino acid	Cre06.g29:	Amino acid	LOC_Os03	amino acid	putative	expressed	
AT1G3183(	Amino acid	Cre06.g29:	Amino acid	LOC_Os03	amino acid	putative	expressed	
AT3G2378(	nuclear RNA polymerase D2A			LOC_Os08	DNA-direct	putative	expressed	
15		Cre10.g43(	(1 of 1) PTH	LOC_Os08	octicosape	putative	expressed	
15		Cre10.g43(	(1 of 1) PTH	LOC_Os08	octicosapeptide/Phox/Bem1p			
72								
97 GO:0008152 GO:0016491 GO:0055114						expressed		
15				LOC_Os08	OsFBX265	expressed		
15				LOC_Os08	OsFBX265	expressed		
15				LOC_Os08	OsFBX265	expressed		
15				LOC_Os08	OsFBX265	chloroplas	putative	expressed
31 GO:0016491 GO:0045300 GO:0055114				LOC_Os08	acyl-desatu	chloroplas	putative	expressed
31 GO:0016491 GO:0045300 GO:0055114				LOC_Os08	acyl-desaturase			

52						putative	expressed
AT4G3798(	elicitor-act	Cre03.g20:	Alcohol del	LOC_Os10	dehydrogenase		
77 GO:0003700 GO:0006355						putative	expressed
AT2G2436(	Protein kinase superfamily protein			LOC_Os08	serine/thre	putative	expressed
AT5G0676(	Late Embryogenesis Abundant 4-5			LOC_Os08	late embry(	putative	expressed
AT5G0676(	Late Embryogenesis Abundant 4-5			LOC_Os08	late embry(	putative	expressed
AT2G2476:	glutamine dumper 4			LOC_Os08	GDU1		
53 GO:0005618 GO:0005975 GO:0006073 GO:0010411 GO:0016762 GO:0042546 GO:0048046							
						putative	expressed
AT4G2982(	homolog of CFIM-25			LOC_Os08	cleavage ar	putative	expressed
AT1G2387(	trehalose-p	Cre06.g27!	Trehalose-(	LOC_Os08	trehalose-6	putative	expressed
AT1G2387(	trehalose-p	Cre06.g27!	Trehalose-(	LOC_Os08	trehalose-6	putative	expressed
AT1G2387(	trehalose-p	Cre06.g27!	Trehalose-(	LOC_Os08	trehalose-6	putative	expressed
AT1G2387(	trehalose-p	Cre06.g27!	Trehalose-(	LOC_Os08	trehalose-6-phosphate synthase		
77 GO:0003700 GO:0006355 GO:0043565				LOC_Os08	expressed	putative	expressed
52 GO:0016758		Cre07.g32:	(1 of 1) PTH	LOC_Os04	cytokinin-C	expressed	
AT2G0299(	ribonuclease 1			LOC_Os08	ribonuclea:	expressed	
				LOC_Os08	OsTIL-2 Te	putative	expressed
AT1G1595(	cinnamoyl	Cre12.g49:	Cinnamoyl	LOC_Os08	cinnamoyl-CoA reductase		
47							
47							
47							
21							
24 GO:0004351 GO:0006536 GO:0016831 GO:0019752 GO:0030170							
24 GO:0004351 GO:0006536 GO:0016831 GO:0019752 GO:0030170							
24 GO:0004351 GO:0006536 GO:0016831 GO:0019752 GO:003017(						putative	expressed
				LOC_Os08	HVA22	Cytochrom	CYP120 superfamily
AT3G1927(	cytochrom	family 707	subfamily ,	polypeptid	Cre07.g325000		
AT4G2500(	alpha-amyl	Cre08.g38!	Alpha-amylase			putative	expressed
AT1G4965(	alpha/beta-Hydrolases superfami			LOC_Os08	gibberellin	expressed	
77 GO:0003700 GO:0006355				LOC_Os08	AP2 domain containing protein		
AT4G00530						putative	expressed
AT1G0989(	Rhamnogalacturonate lyase famil'			LOC_Os08	rhamnogal	putative	expressed
AT1G0989(	Rhamnogalacturonate lyase famil'			LOC_Os08	rhamnogal	putative	expressed
AT1G0989(	Rhamnogalacturonate lyase famil'			LOC_Os08	rhamnogalacturonate lyase		
						C3HC4 typ	expressed
AT3G4718(	RING/U-bo	Cre10.g44:	(1 of 1) K1!	LOC_Os08	zinc finger	putative	expressed
AT3G2006(	ubiquitin-conjugating enzyme19			LOC_Os01	ubiquitin-conjugating enzyme		
77 GO:0003700 GO:0006355							
77							

						expressed		
AT3G2601(	Galactose oxidase/kelch repeat su			LOC_Os08	OsFBX303	C3HC4 typ	expressed	
15 GO:0008270				LOC_Os08	zinc finger	putative	expressed	
AT5G4856(	basic helix-loop-helix (bHLH) DNA			LOC_Os08	BHLH transcription factor			
				LOC_Os08	expressed	Alpha amyl	catalytic d	expressed
AT2G3993(	isoamylase	Cre03.g15!	isoamylase	starch deb	LOC_Os08g40930			
						putative	expressed	
AT5G4756(	tonoplast c	Cre06.g25:	Low-affinity	LOC_Os08	citrate tran	putative	expressed	
AT5G4756(	tonoplast c	Cre06.g25:	Low-affinity	LOC_Os08	citrate tran	putative	expressed	
AT2G1336(	alanine:gly	Cre01.g00!	Serine glyo	LOC_Os08	aminotrans	putative	LINE subcl	expressed
				LOC_Os05	retrotransp	putative	expressed	
71				LOC_Os08	caffeoyl-Co	putative	expressed	
71				LOC_Os08	caffeoyl-Co	C3HC4 typ	expressed	
AT2G0424(	RING/U-box superfamily protein			LOC_Os08	zinc finger	expressed		
				LOC_Os08	fasciclin do	putative	expressed	
72 GO:0004713 GO:0005524 GO:0006468				LOC_Os08	receptor pr	putative	expressed	
37		Cre06.g27:	Apyrase or	LOC_Os12	nucleoside-triphosphatase			
72 GO:0004713 GO:0005524 GO:0006468								
72 GO:0004713 GO:0005524 GO:0006468								
						expressed		
				LOC_Os11	RALFL23 - Rapid ALkalinization Factor RALF family proteir			
59 GO:0008289								
59 GO:0008289								
AT5G1909(	Heavy metal transport/detoxification superfamily protein							
AT5G1909(	Heavy metal transport/detoxification superfamily protein							
AT5G1909(	Heavy metal transport/detoxification superfamily protein							
AT3G1002(	(1 of 1) PTHR12626//PTHR12626:S			LOC_Os11	expressed	putative	expressed	
76 GO:0008083 GO:0008283				LOC_Os11	phytosulfol	putative	expressed	
AT3G5278(	Purple acid phosphatases superfa			LOC_Os11	Ser/Thr pro	putative	expressed	
AT3G5278(	Purple acid phosphatases superfa			LOC_Os11	Ser/Thr pro	putative	expressed	
97 GO:0005506 GO:0016705 GO:0020037 GO				LOC_Os09	cytochrom	putative	expressed	
				LOC_Os12	citrate-bin	putative	expressed	
AT2G3281(	beta galactosidase 9			LOC_Os12	beta-galact	expressed		
AT3G5640(	WRKY DNA-binding protein 70			LOC_Os05	WRKY45			
AT1G71910				LOC_Os12	expressed protein			
AT4G33467				LOC_Os12	expressed	putative	expressed	
15 GO:0016020 GO:0016192				LOC_Os12	syntaxin-related protein KNOLLE			
38		Cre14.g61!	(1 of 5) 2.3.1.43 - Phosphatidylcholine--sterol O-acyltransferase / Phospholipid--					
38		Cre14.g61!	(1 of 5) 2.3.1.43 - Phosphatidylcho			putative	expressed	
AT3G0308(	Zinc-binding dehydrogenase famil			LOC_Os12	NADP-dependent oxidoreductase			
AT3G6276(	Glutathion	Cre13.g58	(1 of 15) 2.5.1.18 - Glutathione transferase / S-(hydroxyalkyl)glutathione lyase					
24 GO:0010181 GO:00:		Cre03.g20!	(1 of 2) K05894 - 12-oxophytodienoic acid reductase (OPR)					
						putative	expressed	
24 GO:0006457 GO:00!		Cre06.g25(	Heat shock	LOC_Os12	DnaK family protein			

AT3G1181(	(1 of 2) PTHR33133:SF7 - F26K24.			LOC_Os12	expressed	putative	expressed	
AT3G4874(	Nodulin Mt	Cre07.g34(	paralog of I	LOC_Os12	nodulin MtI	putative	expressed	
AT1G7423(	glycine-ricl	Cre09.g39:	glycine-ricl	LOC_Os12	glycine-ricl	putative	expressed	
AT1G7423(	glycine-ricl	Cre09.g39:	glycine-ricl	LOC_Os12	glycine-rich RNA-binding protein 7			
50				LOC_Os12	expressed protein			
50				LOC_Os12	expressed protein			
				LOC_Os12	expressed	putative	expressed	
AT3G5539(	Uncharacterised protein family (U			LOC_Os12	membrane associated DUF588 domain containing protei			
24				LOC_Os12	expressed protein			
				LOC_Os12	expressed protein			
				LOC_Os12	expressed protein			
				LOC_Os12	expressed	expressed		
76 GO:0046872				LOC_Os12	ZOS12-08 -	putative	expressed	
18 GO:0005515		Cre16.g68	(1 of 1) K08	LOC_Os01	DNA-binding protein DSP1			
				LOC_Os12	expressed protein			
48 GO:0030598								
57				LOC_Os12	expressed	expressed		
AT1G0424(	AUX/IAA transcriptional regulator f			LOC_Os12	OsIAA31 - Auxin-responsive Aux/IAA gene family member			
53 GO:0005975								
AT1G2915(	non-ATPas	Cre02.g09	26S proteasome regulatory subunit					
AT1G2915(	non-ATPas	Cre02.g09	26S proteasome regulatory subunit					
AT2G35850				LOC_Os12	expressed	expressed		
AT4G3461(	BEL1-like h	Cre08.g37	Gamete-sp	LOC_Os12	homeobox	expressed		
AT4G3461(	BEL1-like h	Cre08.g37	Gamete-sp	LOC_Os12	homeobox	putative	expressed	
AT5G4001(	AAA-ATPas	Cre05.g23	AAA ATPas	LOC_Os12	ATPase 3	putative	expressed	
AT5G4001(	AAA-ATPas	Cre05.g23	AAA ATPas	LOC_Os12	ATPase 3			
				LOC_Os03	expressed protein			
11 GO:0016021						putative	expressed	
AT1G0963(	RAB GTPase 11C			LOC_Os05	ras-related	putative	expressed	
57 GO:0009611				LOC_Os12	inhibitor I f	putative	expressed	
57 GO:0009611				LOC_Os12	inhibitor I f	putative	expressed	
57 GO:0009611				LOC_Os12	inhibitor I family protein			
AT5G0624(	embryo defective 2735			LOC_Os05	expressed protein			
AT4G1855(	alpha/beta	Cre09.g39	(1 of 1) 3.1.1.32 - Phospholipase A(1) / Phospholipase A1					
				LOC_Os05	expressed protein			
				LOC_Os05	expressed protein			
AT5G02640				LOC_Os05	expressed	putative	expressed	
				LOC_Os05	glycine-ricl	putative	expressed	
				LOC_Os05	glycine-ricl	membrane	putative	expressed
AT2G3733(	aluminum sensitive 3			LOC_Os05	ABC transp	chloroplas	putative	expressed
22 GO:0042254		Cre06.g27	Chloroplas	LOC_Os05	50S ribosomal protein L10			

				LOC_Os05	expressed	putative	expressed	
15 GO:0046872		Cre07.g326150		LOC_Os05	zinc finger	putative	expressed	
01 GO:0006979 GO:0020037 GO:0055114				LOC_Os05	peroxidase	short chair	putative	expressed
AT1G5487	NAD(P)-bir	Cre09.g39	Short-chair	LOC_Os05	oxidoreductase			
				LOC_Os05	expressed	putative	expressed	
AT2G4777	TSPO(outer membrane tryptophar			LOC_Os05	peripheral-	putative	expressed	
29				LOC_Os05	lipase	RNA polym	N-terminal	expressed
AT4G1466	RNA polym	N-terminal domain			LOC_Os05	RNA polym	N-terminal	expressed
AT4G1466	RNA polym	N-terminal domain			LOC_Os05	putative	expressed	
01 GO:0006979 GO:0020037 GO:0055114				LOC_Os05	peroxidase precursor			
						HAD super	putative	expressed
93		Cre16.g68	(1 of 1) PF	subfamily	LOC_Os05	HAD super	putative	expressed
93		Cre16.g68	(1 of 1) PF	subfamily	LOC_Os05	putative	expressed	
AT2G1981	CCCH-type	Cre13.g56	possible tr	LOC_Os05	zinc finger	putative	expressed	
AT2G1981	CCCH-type	Cre13.g56	possible tr	LOC_Os05	zinc finger	putative	expressed	
76 GO:0003677 GO:0008270				LOC_Os05	Myb transcription factor			
45 GO:0045735						putative	expressed	
35 GO:0016020 GO:00		Cre07.g35	Zinc-nutriti	LOC_Os05	metal cation transporter			
06 GO:0016491 GO:0055114								
91 GO:0055114								
06 GO:0016491 GO:0055114								
AT2G31090				LOC_Os05	expressed	putative	expressed	
		Cre07.g34	paralog of I	LOC_Os05	nodulin Mtl	putative	expressed	
AT3G1707	Peroxidase family protein			LOC_Os05	peroxidase precursor			
		Cre03.g21	(1 of 1) PTHR10174:SF127 - PHOSPHATIDYLINOSITOL TRANSFER PROTEIN PDR1					
		Cre03.g21	(1 of 1) PTHR10174:SF127 - PHOSPHATIDYLINOSITOL TRANSFER PROTEIN PDR1					
		Cre03.g21	(1 of 1) PTHR10174:SF127 - PHOS			expressed		
33				LOC_Os05	basic helix-loop-helix domain containing protein			
AT3G1098	senescence associated gene 20					putative	expressed	
AT2G4017	Stress induced protein			LOC_Os05	small hydr	putative	expressed	
AT2G4017	Stress induced protein			LOC_Os05	small hydr	putative	expressed	
				LOC_Os05	leaf senes	expressed		
77 GO:0003700 GO:0006355				LOC_Os05	AP2 domai	putative	expressed	
AT2G3713	Peroxidase superfamily protein			LOC_Os07	peroxidase	expressed		
AT4G0141	Late embryogenesis abundant (LE			LOC_Os05	harpin-ind	putative	expressed	
AT2G2362	methyl esterase 1			LOC_Os05	esterase	putative	expressed	
AT2G2362	methyl esterase 1			LOC_Os05	esterase	putative	expressed	
AT5G6640	Dehydrin family protein			LOC_Os11	dehydrin	glycosyl hy	putative	expressed
AT3G5724	beta-1	3-glucanase 3			LOC_Os05	transmemb	putative	expressed
AT4G1547	Bax inhibit	Cre01.g03	(1 of 1) PTH	ISOFORM	LOC_Os05	putative	expressed	
AT1G0322	Eukaryotic aspartyl protease famil			LOC_Os05	basic 7S gl	putative	expressed	
				LOC_Os05	alpha/beta	putative	expressed	
15		Cre11.g46	(1 of 15) 2.	LOC_Os05	glutathione	putative	expressed	
AT5G4838	BAK1-inter	Cre10.g45	(1 of 546) 2	LOC_Os05	inactive rec	putative	expressed	

AT5G4838(	BAK1-inter	Cre10.g45	(1 of 546) 2	LOC_Os05	inactive rec	putative	expressed	
AT5G4838(	BAK1-inter	Cre10.g45	(1 of 546) 2	LOC_Os05	inactive rec	putative	expressed	
AT1G7738(	amino acid permease 3			LOC_Os05	amino acid	putative	expressed	
AT1G7738(	amino acid permease 3			LOC_Os05	amino acid	putative	expressed	
AT1G7738(	amino acid permease 3			LOC_Os05	amino acid transporter			
AT1G09812				LOC_Os05	expressed	putative	expressed	
AT1G7215(	PATELLIN 1	Cre02.g10	(1 of 1) PTH	LOC_Os05	patellin pro	putative	expressed	
AT1G7215(	PATELLIN 1	Cre02.g10	(1 of 1) PTH	LOC_Os05	patellin pro	putative	expressed	
AT1G7215(	PATELLIN 1	Cre02.g10	(1 of 1) PTH	LOC_Os05	patellin pro	putative	expressed	
AT4G0946(	myb domai	Cre06.g28	Hua Enhan	LOC_Os05	MYB family	expressed		
AT5G6475(	Integrase-t	Cre16.g67	AP2-domai	LOC_Os05	AP2 domai	putative	expressed	
77				LOC_Os05	MYB family	putative	expressed	
77				LOC_Os05	MYB family	putative	expressed	
AT2G3812(	Transmembrane amino acid trans			LOC_Os05	transmembrane amino acid transporter protein			
AT2G2967(	Tetratricopeptide repeat (TPR)-like			LOC_Os05	expressed protein			
AT2G2967(	Tetratricopeptide repeat (TPR)-like			LOC_Os05	expressed protein			
AT4G2110!	cytochrome-c oxidases;electron carriers					putative	expressed	
AT2G2938(	highly ABA-induced PP2C gene 3			LOC_Os05	protein pho	putative	expressed	
AT2G2938(	highly ABA-induced PP2C gene 3			LOC_Os05	protein pho	putative	expressed	
AT3G1258(	heat shock	Cre08.g37	Heat shock	LOC_Os01	DnaK famil	putative	expressed	
AT5G0130(	PEBP (phosphatidylethanolamine-			LOC_Os05	phosphatic	putative	expressed	
AT5G0130(	PEBP (phosphatidylethanolamine-			LOC_Os05	phosphatic	expressed		
77 GO:0003700 GO:0006355				LOC_Os05	AP2 domai	aldo/keto r	putative	expressed
AT5G0167(	NAD(P)-linked oxidoreductase sup			LOC_Os05	oxidoreductase			
AT5G0184(	ovate family protein 1					expressed		
AT3G4372(	Bifunctional inhibitor/lipid-transfe			LOC_Os05	LTPL67 - Pr	putative	expressed	
AT3G6306(	EID1-like 3			LOC_Os05	circadian c	expressed		
AT3G1521(	ethylene responsive element bind			LOC_Os05	AP2 domai	putative	expressed	
AT1G1136(	Adenine nu	Cre16.g64	Universal s	LOC_Os05	universal s	expressed		
AT5G4885(	Tetratricopeptide repeat (TPR)-like			LOC_Os05	tetratricopeptide repeat domain containing protein			
24 GO:0005525 GO:00(		Cre13.g56	54kDa Subunit of the Signal Recognition Particle					
24 GO:0005525 GO:00(		Cre13.g56	54kDa Subunit of the Signal Recognition Particle					
						putative	expressed	
AT1G8012(	Protein of unknown function (DUF			LOC_Os05	DUF567 domain containing protein			
AT3G0106(	(1 of 1) PF1	Cre13.g56	Conserved	LOC_Os05	expressed protein			
AT3G0106(	(1 of 1) PF1	Cre13.g56	Conserved	LOC_Os05	expressed	3-beta-gluc	putative	expressed
				LOC_Os05	glucan end	3-beta-gluc	putative	expressed
				LOC_Os05	glucan endo-1			
AT1G2924(	Protein of unknown function (DUF			LOC_Os05	expressed protein			
AT1G6710(	LOB domain-containing protein 40					cyclic phos	putative	expressed
AT4G1893(	RNA ligase,	Cre10.g41	(1 of 2) PTH	3'-CYCLIC-	LOC_Os05	putative	expressed	
AT5G4567(	GDSL-like I	Cre14.g61	(1 of 5) 2.3	LOC_Os05	GDSL-like I	heat shock	putative	expressed
AT1G7431(	heat shock	Cre11.g46	ClpB chape	Hsp100 fa	LOC_Os05	putative	expressed	

72				LOC_Os05	zinc finger/	putative	expressed	
72				LOC_Os05	zinc finger/	3-O-glucos	putative	expressed
52 GO:0016758		Cre07.g32	(1 of 1) PTH	LOC_Os05	anthocyani	putative	expressed	
34 GO:0006334		Cre09.g41	Nucleosom	LOC_Os05	NAP domai	putative	expressed	
34 GO:0006334		Cre09.g41	Nucleosom	LOC_Os05	NAP domai	expressed		
AT5G1184	Protein of unknown function (DUF			LOC_Os05	DUF1230 d	expressed		
AT5G1195	Putative lys	Cre07.g34	(1 of 1) PTH	LOC_Os05	possible ly	expressed		
AT5G1195	Putative lys	Cre07.g34	(1 of 1) PTH	LOC_Os05	possible ly	group 3	putative	expressed
				LOC_Os05	late embry	group 3	putative	expressed
				LOC_Os05	late embry	group 3	putative	expressed
				LOC_Os05	late embry	group 3	putative	expressed
				LOC_Os05	late embry	group 3	putative	expressed
				LOC_Os05	late embry	group 3	putative	expressed
				LOC_Os05	late embry	putative	expressed	
15 GO:0005622 GO:00		Cre12.g52	(1 of 7) PTH	LOC_Os05	phosphatic	putative	expressed	
15 GO:0005622 GO:00		Cre12.g52	(1 of 7) PTH	LOC_Os05	phosphatic	putative	expressed	
15 GO:0005622 GO:00		Cre12.g52	(1 of 7) PTH	LOC_Os05	phosphatic	putative	expressed	
15 GO:0005622 GO:00		Cre12.g52	(1 of 7) PTH	LOC_Os05	phosphatic	putative	expressed	
15 GO:0005622 GO:00		Cre12.g52	(1 of 7) PTH	LOC_Os05	phosphatic	expressed		
AT1G6884	related to ABI3/VP1 2			LOC_Os05	B3 DNA binding domain containing protein			
AT1G6834	Protein of unknown function (DUF			LOC_Os05	expressed protein			
AT1G6834	Protein of unknown function (DUF			LOC_Os05	expressed	putative	expressed	
AT3G1488	(1 of 1) PTHR22952:SF195 - DNA-I			LOC_Os05	transcriptio	expressed		
AT2G2056	DNAJ heat	Cre10.g42	DnaJ-like p	LOC_Os05	dnaJ domain containing protein			
AT5G4609	Protein of u	Cre07.g31	(1 of 1) PTH	LOC_Os05	expressed	putative	expressed	
AT5G6086	RAB GTPas	Cre03.g18	Small Rab-	LOC_Os05	ras-related protein			
				LOC_Os05	expressed	putative	expressed	
24 GO:0004722 GO:0006470				LOC_Os05	protein pho	putative	expressed	
AT3G1857	Oleosin family protein			LOC_Os05	oleosin	putative	expressed	
AT1G2371	Protein of unknown function (DUF			LOC_Os05	DUF1645 domain containing protein			
AT2G2165	Homeodomain-like superfamily protein							
				LOC_Os05	expressed	putative	expressed	
AT1G0130	Eukaryotic aspartyl protease famil			LOC_Os05	aspartic pr	putative	expressed	
				LOC_Os04	reticulon d	putative	expressed	
AT4G2787	Vacuolar iron transporter (VIT) fam			LOC_Os06	integral me	putative	expressed	
AT4G2787	Vacuolar iron transporter (VIT) fam			LOC_Os06	integral me	putative	expressed	
AT4G2787	Vacuolar iron transporter (VIT) fam			LOC_Os06	integral me	putative	expressed	
27 GO:0005524 GO:00		Cre10.g43	Adenylate I	LOC_Os06	adenylate I	late embry	putative	expressed
AT1G3256	Late embry	group 1 protein			LOC_Os06g02040			
77 GO:0005634						putative	expressed	
				LOC_Os06	ATOZI1			
AT4G28310				LOC_Os06	expressed	putative	expressed	
36 GO:0003677 GO:0005634 GO:0006334				LOC_Os06	histone H1	putative	expressed	

				LOC_Os11	von Willebı	eukaryotic	putative	expressed
23 GO:0003743 GO:00(		Cre02.g10:	Eukaryotic	eIF-1A	LOC_Os06	putative	expressed	
AT3G1637(	GDSL-like l	Cre14.g61!	(1 of 5) 2.3	LOC_Os06	GDSL-like l	putative	expressed	
				LOC_Os06	pollen-spe	putative	expressed	
50 GO:0009415				LOC_Os11	dehydrin	expressed		
AT2G4262(	RNI-like superfamily protein			LOC_Os06	OsFBL27 -	putative	expressed	
38		Cre14.g61!	(1 of 5) 2.3	LOC_Os06	GDSL-like l	putative	expressed	
38		Cre14.g61!	(1 of 5) 2.3	LOC_Os06	GDSL-like l	putative	expressed	
				LOC_Os06	harpin-indu	putative	expressed	
				LOC_Os06	harpin-indu	expressed		
				LOC_Os06	LTPL128 - F	putative	expressed	
AT1G1569(	Inorganic H	Cre09.g39	(1 of 1) PF(	LOC_Os06	inorganic H	putative	expressed	
AT1G1569(	Inorganic H	Cre09.g39	(1 of 1) PF(	LOC_Os06	inorganic H	putative	expressed	
AT1G1569(	Inorganic H	Cre09.g39	(1 of 1) PF(	LOC_Os06	inorganic H	putative	expressed	
AT1G1569(	Inorganic H	Cre09.g39	(1 of 1) PF(	LOC_Os06	inorganic H	putative	expressed	
AT1G1569(	Inorganic H	Cre09.g39	(1 of 1) PF(	LOC_Os06	inorganic H	putative	expressed	
AT1G1569(	Inorganic H	Cre09.g39	(1 of 1) PF(	LOC_Os06	inorganic H	putative	expressed	
AT1G8052(	Sterile alpha motif (SAM) domain-			LOC_Os06	jp18	putative	expressed	
AT2G2517(	chromatin	Cre08.g37:	SNF2-relat	LOC_Os06	CHD3-type	putative	expressed	
AT3G1420(	Chaperone DnaJ-domain superfar			LOC_Os06	heat shock protein DnaJ			
				LOC_Os06	expressed protein			
91 GO:0055114						putative	expressed	
52 GO:0016758		Cre07.g32:	(1 of 1) PTH	LOC_Os03	cytokinin-C	putative	expressed	
07 GO:0016020 GO:0042546				LOC_Os06	xyloglucan	putative	expressed	
49		Cre08.g38(	Small prote	LOC_Os01	calvin cycle	putative	expressed	
49		Cre08.g38(	Small prote	LOC_Os01	calvin cycle protein CP12			
AT1G4965(	alpha/beta-Hydrolases superfamily protein							
				LOC_Os06	expressed protein			
						expressed		
				LOC_Os06	phosphate-	LOC_Os06	dehydroge	expressed
AT1G2418(	Thiamin dip	Cre07.g33:	Mitochond	E1 compor	alpha subu	putative	expressed	
AT5G3767(	HSP20-like	Cre07.g31(	heat shock	LOC_Os06	hsp20/alph	putative	expressed	
77		Cre06.g28!	Hua Enhan	LOC_Os06	ODORANT1			
24 GO:0006633 GO:0008152 GO:0008610 GO:0016020 GO:0016747								
						ligand-bin(	putative	expressed
AT4G1268(	(1 of 1) PTHR31133:SF2 - EXPRES!			LOC_Os06	steroid nuc	ligand-bin(	putative	expressed
AT4G1268(	(1 of 1) PTHR31133:SF2 - EXPRES!			LOC_Os06	steroid nuc	putative	expressed	
90				LOC_Os06	late embryogenesis abundant group 1			
						peptidyl-pr	putative	expressed
15 GO:0006457		Cre09.g39(	Peptidyl-pr	FKBP-type	LOC_Os04g28420			
AT1G2090(	Predicted AT-hook DNA-binding family protein							
						expressed		

AT5G1478(	formate de	Cre01.g01!	(1 of 1) PTH	LOC_Os06	erythronate	putative	expressed	
		Cre06.g30!	(1 of 2) KO(	LOC_Os06	reticulon domain containing protein			
AT5G4176:	(1 of 3) PTHR33513:SF4 - GB			LOC_Os06	expressed protein			
						putative	expressed	
AT2G2049!	(1 of 1) PTH	Cre07.g34!	(1 of 1) PTH	LOC_Os07	serine/thre	putative	expressed	
AT2G2049!	(1 of 1) PTH	Cre07.g34!	(1 of 1) PTH	LOC_Os07	serine/threonine-protein kinase 19			
)0 GO:0005634 GO:0006355 GO:0043565								
)0 GO:0005634 GO:0006355 GO:0043565								
;3 GO:0005975		Cre07.g32(	(1 of 2) 3.2.1.21 - Beta-glucosidas			putative	expressed	
AT1G1097(	zinc transp	Cre07.g35!	Zinc-nutriti	LOC_Os06	metal catic	putative	expressed	
;3 GO:0005975				LOC_Os06	beta-galact	putative	expressed	
;3 GO:0005975				LOC_Os06	beta-galact	putative	expressed	
AT1G1718(	glutathione	Cre11.g46:	(1 of 15) 2.:	LOC_Os07	glutathione S-transferase			
;2 GO:0016491 GO:0016620 GO:0055114								
;2 GO:0016758								
;2 GO:0016758								
15 GO:0008270						peroxiredo:	putative	expressed
AT3G5296(	Thioredoxir	Cre01.g01	Peroxiredo:	type II	LOC_Os06	expressed		
AT3G2212(	cell wall-plasma membrane linker			LOC_Os06	LTPL129 - F	expressed		
AT3G2212(	cell wall-plasma membrane linker			LOC_Os06	LTPL129 - F	expressed		
AT3G2212(	cell wall-plasma membrane linker			LOC_Os06	LTPL129 - F	putative	expressed	
AT3G5258(	Ribosomal	Cre11.g48(	Cytosolic 8	LOC_Os02	ribosomal protein			
						putative	expressed	
AT2G2415(	heptahelic	Cre06.g30:	(1 of 1) PTH	LOC_Os06	haemolysir	putative	expressed	
21				LOC_Os06	tetraspanir	phosphogly	putative	expressed
AT1G7955(	phosphogly	Cre11.g46:	Phosphogly	chloroplas	LOC_Os06	phosphogly	putative	expressed
AT1G7955(	phosphogly	Cre11.g46:	Phosphogly	chloroplas	LOC_Os06	phosphogly	putative	expressed
AT1G7955(	phosphogly	Cre11.g46:	Phosphogly	chloroplas	LOC_Os06	putative	expressed	
AT5G2062(	ubiquitin 4			LOC_Os05	ubiquitin fa	putative	expressed	
AT5G2062(	ubiquitin 4			LOC_Os05	ubiquitin fa	putative	expressed	
AT5G2062(	ubiquitin 4			LOC_Os05	ubiquitin family protein			
15								
15						putative	expressed	
AT5G5387(	early nodul	Cre14.g62!	(1 of 1) PF(	LOC_Os06	early nodul	putative	expressed	
AT4G2132(	Aldolase-type TIM barrel family pr(			LOC_Os06	phosphosulfolactate synthase-related protein			
						expressed		
)9				LOC_Os06	OsCML29 -	ATP-bindir	putative	expressed
AT1G6794(	non-intrins	Cre06.g27:	Chloroplas	LOC_Os06	ABC transp	ATP-bindir	putative	expressed

AT1G6794(	non-intrins	Cre06.g27:	Chloroplas	LOC_Os06	ABC transp	putative	expressed
AT5G5755(	xyloglucan endotransglucosylase/			LOC_Os06	glycosyl hy	putative	expressed
AT4G3175(	HOPW1-1-	Cre12.g52!	Flagellar As	LOC_Os06	protein phc	putative	expressed
AT4G3175(	HOPW1-1-	Cre12.g52!	Flagellar As	LOC_Os06	protein phosphatase 2C		
AT5G4069(	(1 of 1) PTHR34278:SF1 - GB			LOC_Os05	expressed	expressed	
AT3G1302(	hAT transposon superfamily prote			LOC_Os06	hAT dimeri:	expressed	
AT3G1302(	hAT transposon superfamily prote			LOC_Os06	hAT dimeri:	expressed	
AT3G1302(	hAT transposon superfamily prote			LOC_Os06	hAT dimeri:	putative	expressed
AT4G1239(	pectin methylesterase inhibitor 1			LOC_Os06	invertase/p	putative	expressed
AT4G1239(	pectin methylesterase inhibitor 1			LOC_Os06	invertase/p	expressed	
56	GO:0003676			LOC_Os06	RNA recogr	expressed	
56	GO:0003676			LOC_Os06	RNA recogr	expressed	
56	GO:0003676			LOC_Os06	RNA recognition motif containing protein		
AT1G7624(	Arabidopsis protein of unknown fu			LOC_Os06	expressed protein		

in depedent protein kinases

expressed

putative expressed

ein kinase precursor

ulfur protein

RASES // PROTEIN UGT-61-RELATED

ıit Tim17

expressed

'calmodulin depedent protein kinases

putative expressed

leate 9S-lipoxygenase / Linoleate 9-lipoxygenase

ein precursor

-cholesterol acyltransferase

ein

ELATED

expressed

expressed
expressed
expressed
expressed

AMILY NOT NAMED

protein precursor

expressed

n

ı precursor

-cholesterol acyltransferase

n

16-RELATED
16-RELATED
