# Supplementary figures and images for: Large scale deletion and rebalancing within the k1C kafirin family in sorghum
Source: Front Plant Sci. 2025 Oct 23;16:1686027. doi: 10.3389/fpls.2025.1686027 (PMC12588965; doi:10.3389/fpls.2025.1686027)

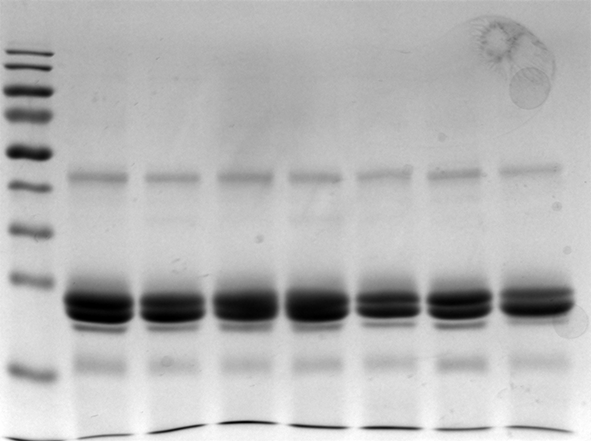

Supplement: Supplementary file 3 [file Image1.tif]

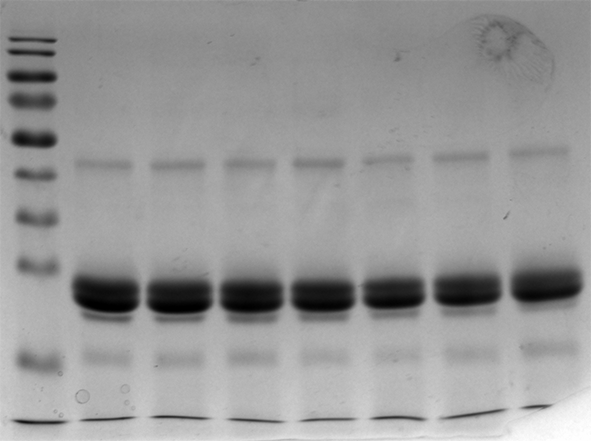

Supplement: Supplementary file 4 [file Image2.tif]

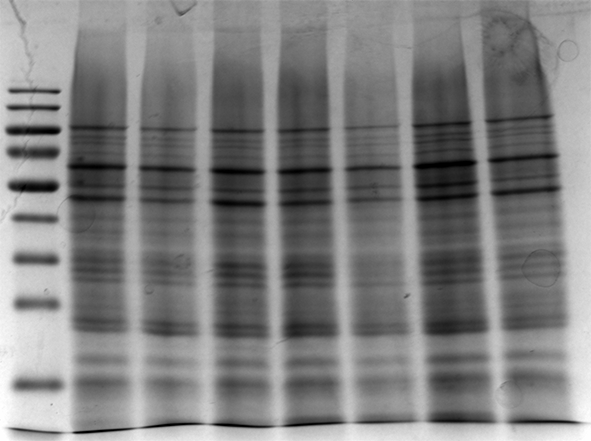

Supplement: Supplementary file 5 [file Image3.tif]

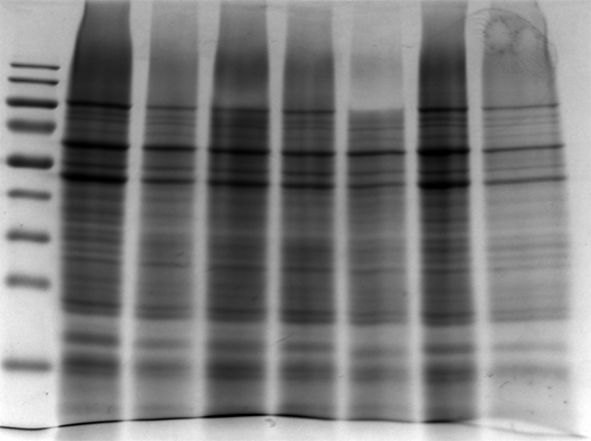

Supplement: Supplementary file 6 [file Image4.tif]

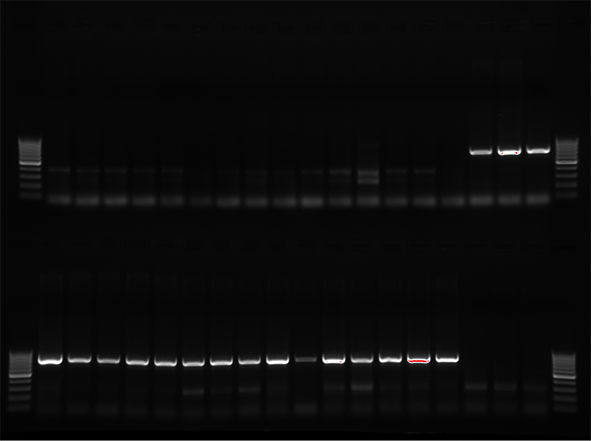

Supplement: Supplementary file 7 [file Image5.tif]

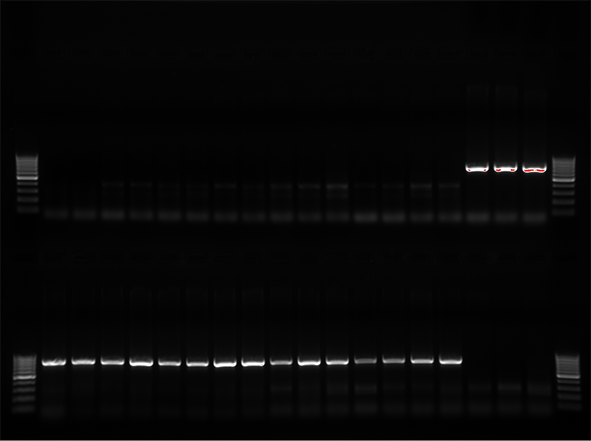

Supplement: Supplementary file 8 [file Image6.tif]
